# Tris(2,4,6‐trifluorophenyl)borane: An Efficient Hydroboration Catalyst

**DOI:** 10.1002/chem.201703109

**Published:** 2017-07-27

**Authors:** James R. Lawson, Lewis C. Wilkins, Rebecca L. Melen

**Affiliations:** ^1^ School of Chemistry Cardiff University, Main Building Park Place Cardiff Cymru/Wales CF10 3AT UK

**Keywords:** boron, catalysis, hydroboration, Lewis acids, metal-free

## Abstract

The metal‐free catalyst *tris*(2,4,6‐trifluorophenyl)borane has demonstrated its extensive applications in the 1,2‐hydroboration of numerous unsaturated reagents, namely alkynes, aldehydes and imines, consisting of a wide array of electron‐withdrawing and donating functionalities. A range of over 50 borylated products are reported, with many reactions proceeding with low catalyst loading under ambient conditions. These pinacol boronate esters, in the case of aldehydes and imines, can be readily hydrolyzed to leave the respective alcohol and amine, whereas alkynyl substrates result in vinyl boranes. This is of great synthetic use to the organic chemist.

The hydroboration reaction has been rigorously explored, with many historic examples utilizing transition‐metal catalysts such as rhodium, palladium and platinum.^1^ More recently, the use of metal‐free catalysts, often derived from p‐block elements,^2^ has been developed. However, cases of alkaline earth metal centered catalysts have also been documented with good success,^3^ allowing access to a wider variety of borylated substrates without the necessity of removing trace‐metal impurities.^4^ Indeed, many hydroboration reactions are often atom‐efficient, utilizing hydroboranes such as pinacol borane (HBPin),^5^ Piers’ borane (HB(C_6_F_5_)_2_)^6^ or 9‐BBN.^7^ The resulting borylation process more often yields the *syn*‐hydroboration product, however, the *trans*‐hydroboration has been reported in the literature (Scheme 1, top).^8^ The hydroboration of carbon–carbon double and triple bonds provides access to synthetically useful borylated molecules that can be readily functionalized further through cross‐coupling reactions such as the Suzuki reaction.^9^ In other work, the hydroboration of alkenes and imines with HBPin was found to be catalyzed by the functionalized triarylborane *tris*[3,5‐*bis*(trifluoromethyl)phenyl]borane (BArF_3_), which was found to be a superior catalyst to the archetypical Lewis acid B(C_6_F_5_)_3_ (Scheme 1, middle).^10^ It has also been shown that the catalytic hydroboration of alkynes is achievable using Piers’ borane [HB(C_6_F_5_)_2_] as a catalyst.^11^ Although metal catalyzed hydroborations of aldehydes^12^ and imines^13^ have been described, metal‐free alternatives are seldom reported.^14^ In addition to those reactions described above, the hydroboration of C=O and C=N bonds results in borylated alcohols and amines, respectively, which can in turn undergo hydrolysis to generate the free alcohol and amine, thus providing a simple synthetically accessible pathway for heteroatom double‐bond reduction.

**Scheme 1:**
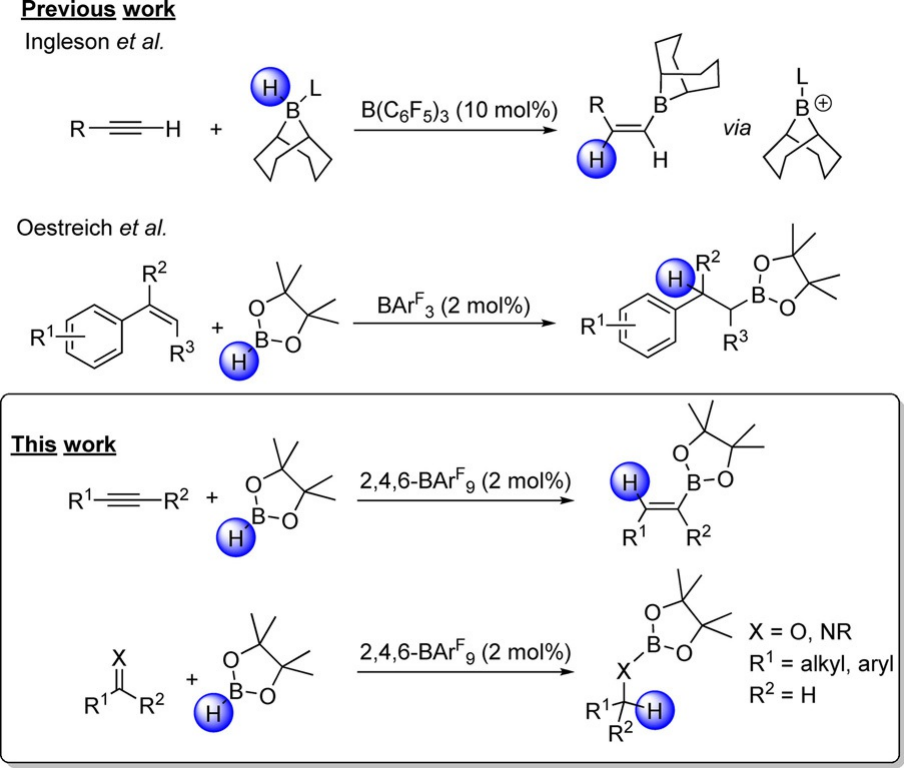
Previous catalytic hydroboration reactions and this work.

In this work, we sought a new metal‐free catalytic protocol for the hydroboration of a wide variety of C−X (X=C, N, O) multiple bonds. Our goal was to identify a highly Lewis acidic boron‐based catalyst that presented no competing reactivity when exposed to a broad array of substrates featuring various functional groups. Early in our studies, *tris*(2,4,6‐trifluorophenyl)borane (2,4,6‐BAr^F^_9_) showed great potential for this task.^15^ When combined with phenylacetylene in a 1:1 molar ratio, NMR spectroscopic studies showed no 1,1‐carboboration of the alkyne, as is observed with other Lewis acidic triaryl boranes such as B(C_6_F_5_)_3_.^16^ Heating at 60 °C for 120 hours still did not induce any reactivity, showcasing this catalysts lack of side‐reaction. Additionally, there was no evidence of ligand redistribution between the HBPin reagent and the catalyst as has been identified previously, presumably due to the presence of the *o*‐fluorine atoms on the phenyl rings.10a Thus, 2,4,6‐BAr^F^_9_ was selected for screening to probe its effectiveness as a catalyst for hydroboration.

Initially, the hydroboration of phenylacetylene was studied due to its long‐standing use as a model reagent for this transformation,^17^ with the conversion being measured by in situ multinuclear NMR spectroscopy. To offer contrast to our chosen catalyst, it was compared against other fluorinated triaryl boranes, including B(C_6_F_5_)_3_, *tris*(2,6‐difluorophenyl)borane (2,6‐BAr^F^_6_), as well as the non‐fluorinated triphenylborane (entry 1–3, Table 1). Initial hydroborations were carried out at 5 mol % catalyst loading, and it was discovered that 2,4,6‐BAr^F^_9_ facilitated hydroboration of phenylacetylene within 5 hours (entry 4, Table 1). This borane showed superior reactivity when compared to that of the archetypal Lewis acid, B(C_6_F_5_)_3_, which failed to reach completion after 18 hours, giving just 59 % conversion (entry 1, Table 1). The less Lewis acidic borane, 2,6‐BAr^F^_6_, showed only slight improvements over B(C_6_F_5_)_3_, with 62 % conversion (entry 2, Table 1), and BPh_3_ demonstrated just 31 % conversion (entry 3, Table 1). Despite the molecular structure of 2,4,6‐BAr^F^_9_ and 2,6‐BAr^F^_6_ differing only by the substitution of a *p*‐F atom, work by Alcarazo has elucidated their relative Lewis acidities in the series: B(C_6_F_5_)_3_ (100 %) >2,4,6‐BAr^F^_9_ (70 %) >2,6‐BAr^F^_9_ (56 %), which perhaps sheds light on the observed conversions for entries 2 and 4 (Table 1).^15^ Following this, solvent effects were probed with THF, Et_2_O and toluene, used in addition to CH_2_Cl_2_ (entry 4–7, Table 1). Comparable results were garnered in toluene (entry 5, Table 1) as in CH_2_Cl_2_, however no reaction was observed with coordinating solvents, most likely due to the sequestration of the borane catalyst (entry 6–7, Table 1). CH_2_Cl_2_ was chosen over toluene to facilitate more convenient purification of the product. Finally, catalyst and HBPin loading was explored (entry 8–13, Table 1). It was noted that the catalyst could be lowered to 1 mol % without significant deleterious impact on the conversion; equally, increasing to 10 mol % only had slight positive impact on the rate of reaction, leading to full conversion after 4 hours. Although a stoichiometric amount of HBPin yielded quantitative conversion in this system, 1.2 equivalents of HBPin were used going forward to facilitate maximum conversion in subsequent reactions; any unreacted excess HBPin was readily removed in vacuo. Additionally, catecholborane (HBCat) was trialled as an alternative borylation reagent using the established catalytic procedure, which garnered the vinylboronate ester in slightly lower conversions of 85 % after 6 hours (entry 14, Table 1).

**Table 1:** Reaction condition optimization.

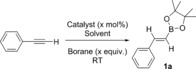						
Entry	Catalyst	Loading [mol %]	Borane (equiv.)	Solvent	*t* [h]	Conversion^[a]^ [%]
1	B(C_6_F_5_)_3_	5	HBPin (1)	CH_2_Cl_2_	18	59
2	2,6‐BAr^F^_6_	5	HBPin (1)	CH_2_Cl_2_	18	62
3	BPh_3_	5	HBPin (1)	CH_2_Cl_2_	18	31
4	2,4,6‐BAr^F^_9_	5	HBPin (1)	CH_2_Cl_2_	5	99
5	2,4,6‐BAr^F^_9_	5	HBPin (1)	Toluene	5	99
6	2,4,6‐BAr^F^_9_	5	HBPin (1)	THF	18	0
7	2,4,6‐BAr^F^_9_	5	HBPin (1)	Et_2_O	18	0
8	2,4,6‐BAr^F^_9_	1	HBPin (1)	CH_2_Cl_2_	18	99
9	2,4,6‐BAr^F^_9_	2	HBPin (1)	CH_2_Cl_2_	6	99
10	2,4,6‐BAr^F^_9_	10	HBPin (1)	CH_2_Cl_2_	4	99
11	2,4,6‐BAr^F^_9_	2	HBPin (1.2)	CH_2_Cl_2_	6	99
12	2,4,6‐BAr^F^_9_	2	HBPin (2)	CH_2_Cl_2_	6	99
13	2,4,6‐BAr^F^_9_	2	HBPin (5)	CH_2_Cl_2_	5	99
14	2,4,6‐BAr^F^_9_	2	HBCat (1.2)	CH_2_Cl_2_	6	85

[a] Conversion measured using in situ ^1^H NMR spectroscopy.

With optimized reaction conditions for the hydroboration of phenylacetylene in hand, using HBPin as the borylation reagent, the reaction scope was expanded to a range of terminal alkynes. Simple aryl‐ and alkyl‐substituted terminal alkynes proceeded rapidly to the hydroboration products **1** **a**–**d** at ambient temperature, giving good‐to‐excellent isolated yields of 71–99 %. Propargyl esters were found to react exclusively with the alkyne functionality in good yields (55–77 %), with no observable reduction of the ester moiety (**1** **e**–**h**). Furthermore, propargyl acrylate was reacted to give **1** **i** with exclusive hydroboration of the alkyne over the alkene in 87 % yield. The generation of **1** **l** from the diyne featuring both terminal and internal alkynes displayed selective hydroboration of the terminal triple bond over the internal unit. In a bid to expand the scope of this reaction, reagents exclusively featuring internal alkynes were targeted next. Combinations of alkynes featuring aryl and alkyl termini were successfully hydroborated to give **1** **m**–**p** with some of the best isolated yields of 80–96 %. Of particular note, the use of asymmetric internal alkynes led to a single regioisomer predominating in products **1** **m** and **1** **o** (Scheme 2). In situ NMR spectroscopic studies indicate that the two regioisomers were formed in a roughly 10:1 ratio, preferring a geminal methyl/borane configuration. Storing saturated CH_2_Cl_2_ solutions of **1** **k** and **1** **p** gave croppings of colorless crystals, which could be measured by X‐ray crystallography to determine the *trans*‐alkene molecular structure as a result of the *syn*‐addition reaction, (Figure 1). Alkenyl substrates were also attempted, however they were met with more limited success than their alkynyl counterparts in contrast to the work of Oestreich et al.10a

**Scheme 2:**
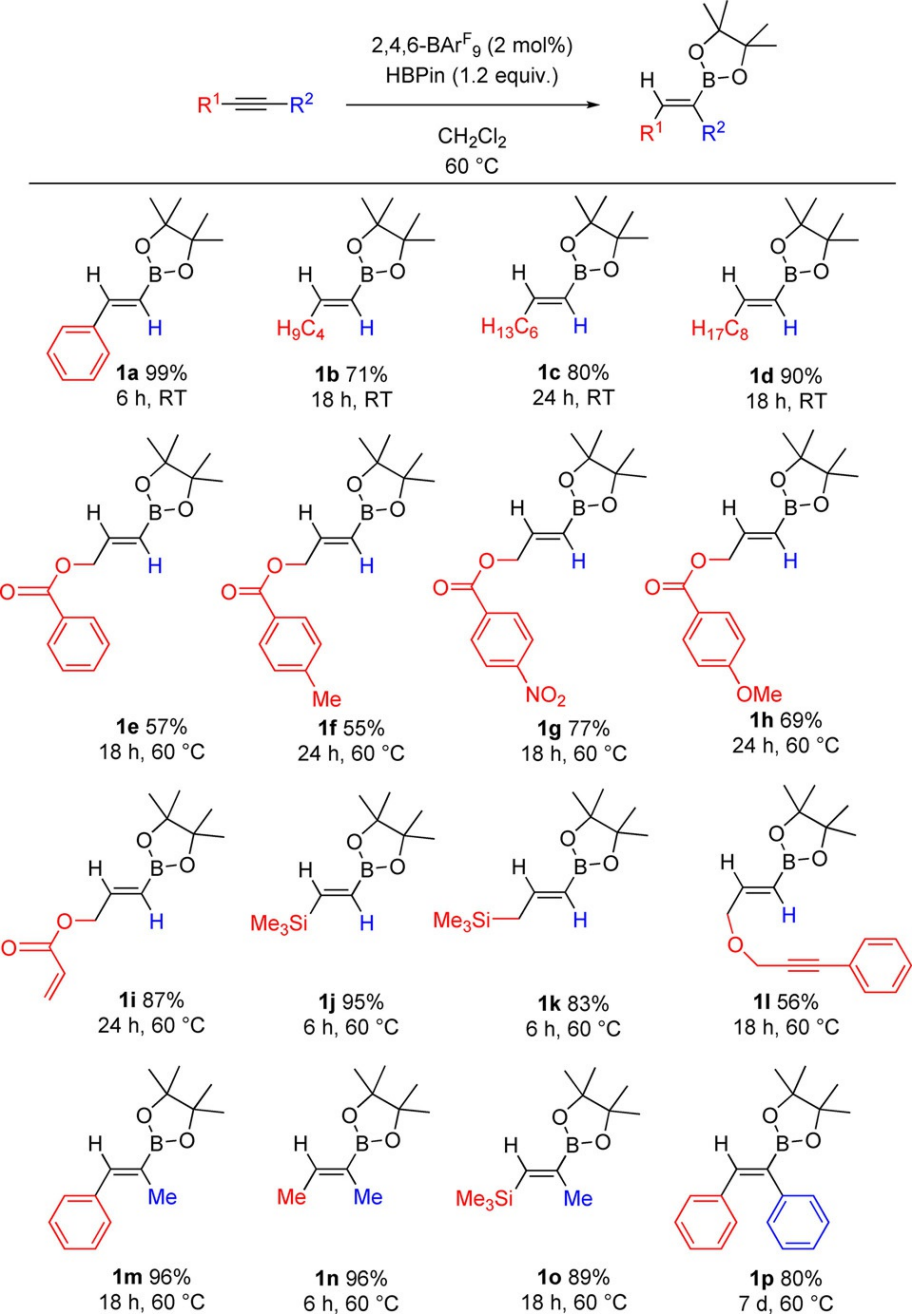
Hydroboration of various internal and terminal alkynes. Conditions for given isolated yield noted.

**Figure 1:**
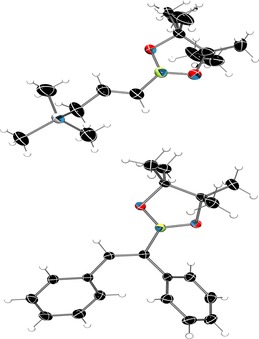
Solid‐state structure of **1** **k** and **1** **p**. Thermal ellipsoids shown at 50 % probability. C: black, H: white, O: red, B: yellow green, Si: grey. Disordered pinacol unit of **1 k** modelled over multiple sites with solvent molecules omitted for clarity.

The 1,2‐hydroboration of aldehydes was then examined, beginning with benzaldehyde (Scheme 3). Using the optimized reaction conditions established previously, it was observed using multinuclear NMR spectroscopy that the aldehyde was completely consumed within 1 hour at room temperature. Removal of volatiles in vacuo and redissolution in CDCl_3_ gave multinuclear NMR data confirming the hydroborated benzaldehyde (**2** **a**) as the sole product. Following this, the hydroboration reaction was extended to several other aldehydes to explore the functional group tolerance (Scheme 3). Beginning with substituted benzaldehydes, it was observed that electron‐withdrawing groups (including *p*‐NO_2_, *o*‐CN, *p*‐F and *p*‐CF_3_) and electron‐donating groups such as OMe could be included in both the *ortho*‐ and *para*‐positions with little effect on reactivity (**2** **a**–**m**). Heteroarenes (**2** **o**–**p**) were tolerated under comparable conditions to other substituents, indicating that potential sequestration of the borane or catalyst by the coordinating heteroatom is a reversibly facile process. Fused aryl systems (**2** **n**), alkyl substituents (**2** **q**–**s**) as well as cyclic aliphatics (**2** **t**) were all tolerated, explicating the versatility of this synthetic methodology. It was, however, found that elevated temperatures were required to achieve full conversion for most substrates, notably *ortho*‐substituted benzaldehydes and some electron‐withdrawing functionalities, with slightly longer reaction times being noted.

**Scheme 3:**
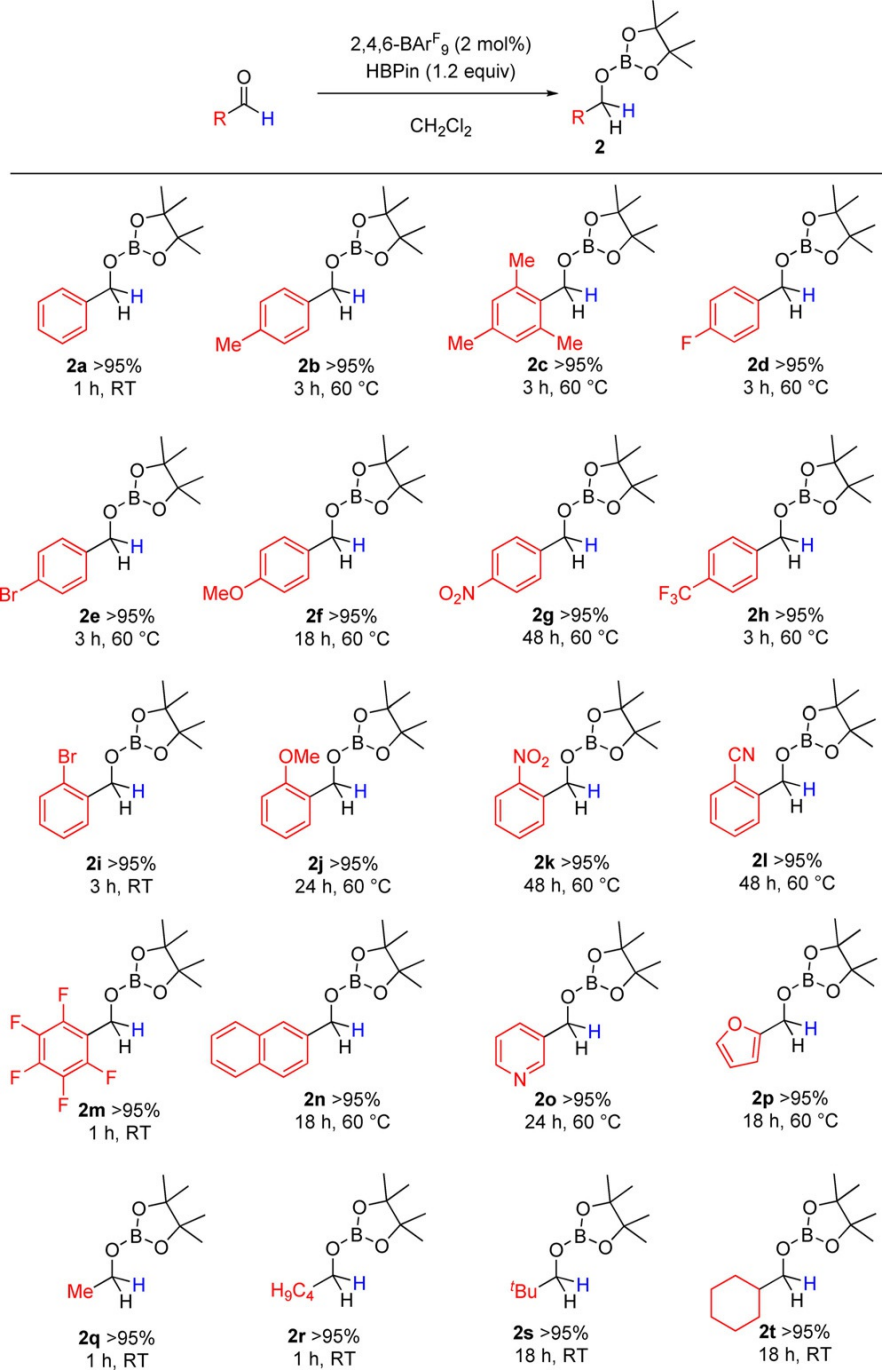
Hydroboration of aldehydes. Conditions indicated to reach quantitative conversion by in situ ^1^H NMR spectroscopy.

Following aldehydes, C=N bond hydroboration was investigated, beginning with *N*‐benzylideneaniline. It was once again observed that hydroboration occurred rapidly using the same reaction conditions, with exclusive product formation as observed by multinuclear NMR spectroscopy, showing full conversion of the imine to the borolanamine **3** **a** within 4 hours. Indeed, other recent studies have shown similar results when using *tris*(3,5‐bis(trifluoromethyl)phenyl)borane in imine reductions with good yields being reported.10b Expansion of the substrate scope required a library of imines, which were readily synthesized using literature procedures.^18^ Hydroboration of these various imines was readily achieved, featuring aryl groups substituted with alkyl, fused aryl, electron withdrawing‐ and donating‐groups, as well as variance on the nitrogen atom (Scheme 4). It was noted that electron‐rich R^1^ aryl groups gave the corresponding aminoborane again in quantitative yields (**3** **b**–**e**) with the analogous electron‐poor moieties performing equally as well (**3** **f**–**g**). Moreover, functionalization of the R^2^ unit had little impact on reactivity whereby aliphatic groups were tolerated well, generating the borylated products **3** **i**–**l** quantitatively in as little as 4 hours at 60 °C. Sterically encumbered 2,6‐diethylphenyl and 2,4,6‐trimethylphenyl substituted amines (**3** **m**–**n**) performed well with other functionalities such as *p*‐CF_3_ and *o*‐F (**3** **o**–**p**) posing no obstacle. Some borylated amine products were found to be sensitive to protodeboration upon work‐up, and as such were fully hydrolyzed to the secondary amine for the purpose of NMR analysis (**3** **d**–**g**, **3** **p**).

**Scheme 4:**
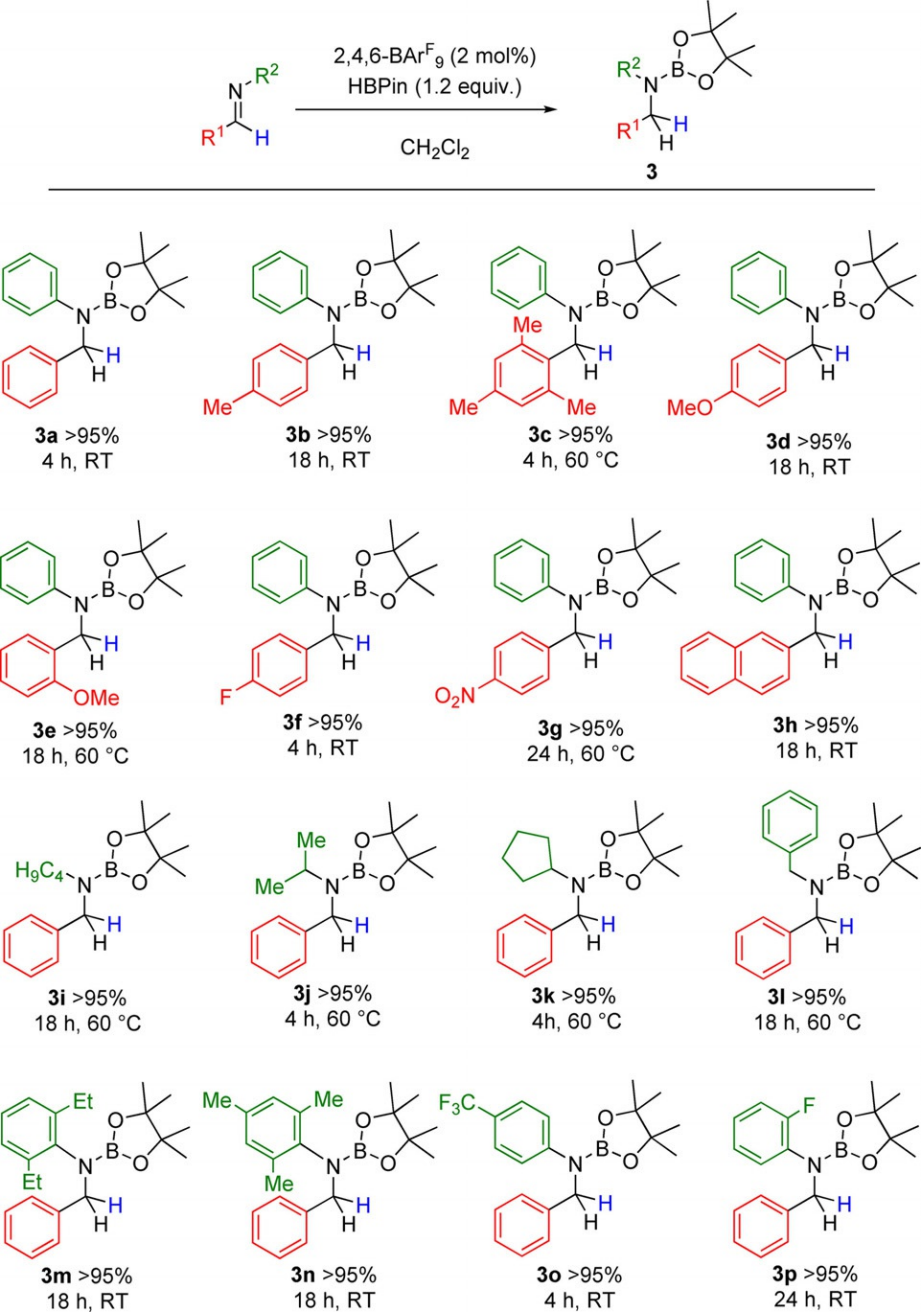
Hydroboration of imines. Conditions indicated to reach quantitative conversion by in situ ^1^H NMR spectroscopy.

Within this work, we have demonstrated that *tris*(2,4,6‐trifluorophenyl)borane, 2,4,6‐BAr^F^_9_, is an extremely versatile reagent for the hydroboration of a wide variety of substrates. This catalyst is particularly well‐suited for this transformation as it precludes any reactivity with the unsaturated frameworks, as is observed with other Lewis acid boranes, while still remaining catalytically active. Alkynes, aldehydes and aldimines of various steric and electronic character are indeed compatible with most reactions requiring low catalyst loading and relatively mild reaction conditions, whereby the products are simply purified in vacuo or by passing through a short silica gel plug. Future investigations will look at mechanistic aspects of this transformation to ascertain further information on potentially reactive intermediates and expand this methodology further.

## Conflict of interest

The authors declare no conflict of interest.

## Supporting information

As a service to our authors and readers, this journal provides supporting information supplied by the authors. Such materials are peer reviewed and may be re‐organized for online delivery, but are not copy‐edited or typeset. Technical support issues arising from supporting information (other than missing files) should be addressed to the authors.

Supplementary
