# Supplementary material for: Tris(2,4,6‐trifluorophenyl)borane: An Efficient Hydroboration Catalyst
Source: Chemistry. 2017 Jul 27;23(46):10997–1000. doi: 10.1002/chem.201703109 (PMC5577513; doi:10.1002/chem.201703109)
Supplement: Supplementary file 1 — Supplementary [file CHEM-23-10997-s001.pdf]

CHEMISTRY
A European Journal

Supporting Information

Tris(2,4,6-trifluorophenyl)borane: An Efficient Hydroboration Catalyst

James R. Lawson+, Lewis C. Wilkins+, and Rebecca L. Melen*[a]

chem_201703109_sm_miscellaneous_information.pdf

Contents

1. Experimental 2

1.1 General experimental 2

1.2 Starting material synthesis 3

1.3 General hydroboration 8

1.4 Hydroboration of alkynes 8

1.5 Hydroboration of aldehydes. 13

1.6 Hydroboration of imines. 18

2. Experimental: NMR spectra 23

2.1 NMR spectra of starting materials. 23

2.2 NMR spectra of products 48

3. Crystallographic studies. 207

3.1 General information and refinement data. 207

3.2 Thermal ellipsoid plots. 208

4. References 210

1. Experimental

1.1 General experimental

With the exception of the starting materials, all reactions and manipulations were carried out under an atmosphere of dry, O_2-free nitrogen using standard double-manifold techniques with a rotary oil pump. A nitrogen-filled glove box (MBraun) was used to manipulate solids including the storage of starting materials, room temperature reactions, product recovery and sample preparation for analysis. All starting materials other than those synthesized as outlined below were purchased from commercial sources (Sigma-Aldrich, Alfa Aesar, Acros, Fluorochem, TCI) and were used as received without subsequent purification. All solvents (toluene, CH_2Cl_2, hexane) were dried by employing a solvent purification system MB SPS-800 and stored under a nitrogen atmosphere. Deuterated solvents were distilled and/or dried over molecular sieves before use. Chemicals were purchased from commercial suppliers and used as received. ^{1}H, ^{13}C, ^{11}B, ^{19}F and ^{29}Si NMR spectra were recorded on a Bruker Avance II 400 or Bruker Avance 500 spectrometers. Chemical shifts are expressed as parts per million (ppm, δ) downfield of tetramethylsilane (TMS) and are referenced to $CDCl_3$ (7.26/77.16 ppm) as internal standards. NMR spectra were referenced to $BF_3{\cdot}Et_2O/CDCl_3$ (^{11}B), $CFCl_3$ (^{19}F) and Me_4Si (^{29}Si). The description of signals include: s = singlet, d = doublet, t = triplet, q = quartet, sep = septet, m = multiplet and br. = broad. All coupling constants are absolute values and are expressed in Hertz (Hz). ^{13}C NMR was measured as ^{1}H decoupled. Yields are given as isolated yields. All spectra were analyzed assuming a first order approximation. Mass spectra were measured in house on a Waters LCT Premier/XE or a Waters GCT Premier spectrometer.

1.2 Starting material synthesis

General procedure a: According to the literature,[1] the corresponding di- or trifluoro-substituted bromobenzene (3 equiv.) was dissolved in freshly distilled THF (100 mL) and cooled to -20 °C. At this temperature ^{i}PrMgCl (3 equiv., 2.0 M in THF) was added dropwise. The reaction mixture was then allowed to reach 0 °C and after 1 h at this temperature cooled again to -50 °C. Subsequently, BF_3•Et_2O (1 equiv.) was added dropwise and after 1 h the cooling bath was removed and the reaction mixture warmed to room temperature within another hour. Removal of all volatiles and sublimation of the remaining solid (120 °C, 1 x 10^{-3} mbar) afforded the desired boranes as white solids.

Tris(2,6-difluorophenyl)borane

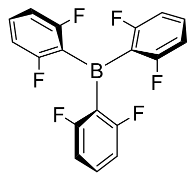

Synthesized in accordance with ***general procedure a*** using 1-bromo-2,6-difluorobenzene (3.39 mL, 30 mmol, 3 equiv.), ^{i}PrMgCl (15 mL, 30 mmol, 3 equiv.) and BF_3•Et_2O (1.23 mL, 10 mmol, 1 equiv.) to give the pure product (2.14 g, 6.1 mmol, 61%). Spectroscopic analyses agree with literature established values.[1] **^{1}H NMR** (500 MHz, $CDCl_3$, 298 K) δ/ppm: 7.59–7.20 (m, 3H, aryl), 6.87 (t, $^{3}J_{HF}$ = 7.8 Hz, 6H, aryl). **^{11}B NMR** (128 MHz, $CDCl_3$, 298 K) δ/ppm: 62.8 (br. s). **^{19}F NMR** (471 MHz, $CDCl_3$, 298 K) δ/ppm: -99.05.

Tris(2,4,6-trifluorophenyl)borane

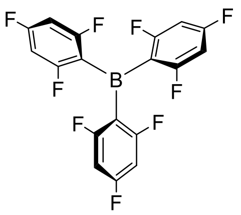

Synthesized in accordance with ***general procedure a*** using 1-bromo-2,4,6-trifluorobenzene (3.50 mL, 30 mmol, 3 equiv.), ^{i}PrMgCl (15 mL, 30 mmol, 3 equiv.) and BF_3•Et_2O (1.23 mL, 10 mmol, 1 equiv.) to give the pure product (3.35 g, 8.3 mmol, 83%). Spectroscopic analyses agree with literature established values.[1] **^{1}H NMR** (500 MHz, $CDCl_3$, 298 K) δ/ppm: 6.64 (t, $^{3}J_{HH}$ = 8.3 Hz, 6H, aryl). **^{11}B NMR** (128 MHz, $CDCl_3$, 298 K) δ/ppm: 58.4 (br. s). **^{19}F NMR** (471 MHz, $CDCl_3$, 298 K) δ/ppm: -95.75 (d, $^{3}J_{HF}$ = 10.4 Hz, 6F, *o*-F), -100.31 (t, $^{3}J_{HF}$ = 10.4 Hz, 3F, *p*-F).

General procedure b: Synthesized according to the literature[2] whereby propargyl alcohol (0.72 mL, 12.5 mmol, 1 equiv.) was dissolved in 100 mL CH_2Cl_2 and cooled to -78 °C. Tetramethylethylenediamine (TMEDA) (1.12 mL, 7.5 mmol, 0.6 equiv.) was added followed by dropwise addition of acyl chloride (13.8 mmol, 1.1 equiv.). After stirring at this temperature for 30 minutes the reaction was warmed to room temperature and quenched with saturated NH_4Cl solution. The aqueous phase was extracted with CH_2Cl_2 (3 × 30 mL) with the combined organic phases being washed with H_2O then dried over K_2CO_3 and Na_2SO_4. Removal of volatiles under reduced pressure gave the crude product as a pale yellow oil. This was purified by either recrystallisation or column chromatography.

General procedure c: According to the literature[3] whereby triethylamine (1.0 equiv.) and 4-dimethylaminopyridine (DMAP) (0.02 equiv.) were added to a solution of the propargyl alcohol (1.0 equiv.) in CH_2Cl_2 (ca. 20 mL) and was stirred for 5 min. The solution was cooled to 0 °C and the acyl chloride (1.0 equiv.) added dropwise. The resulting mixture was stirred at this temperature for 30 min and was then allowed to warm to room temperature and was stirred for a further 48 h. The reaction was quenched with water and the aqueous layer extracted twice with CH_2Cl_2. The collective organic phases were washed with brine, dried with $MgSO_4$, filtered, and the solvent was removed under vacuum and the product purified by column chromatography or recrystallisation.

Prop-2-yn-1-yl benzoate

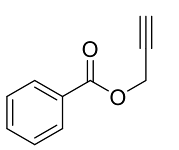

Synthesized according to ***general procedure b*** using benzoyl chloride (1.60 mL, 13.8 mmol, 1.1 equiv.). Purification *via* column chromatography (hexane/ethyl acetate, 20:1) gave the pure product as a colorless oil. Yield: 1.82 g, 11.4 mmol, 91%. Spectroscopic analyses agree with literature established values.[4] **^{1}H NMR** (500 MHz, $CDCl_3$, 298 K) δ/ppm: 8.09 – 8.07 (m, 2H, aryl), 7.58 (tt, $^3J_{HH}$ = 7.5 Hz, $^4J_{HH}$ = 1.3 Hz, 1H, aryl), 7.45 (t, $^3J_{HH}$ = 7.6 Hz, 2H, aryl), 4.93 (d, $^4J_{HH}$ = 2.5 Hz, 2H, CH_2), 2.52 (t, $^4J^{HH}$ = 2.5 Hz, 1H, ≡CH).

Prop-2-yn-1-yl-4-methylbenznoate

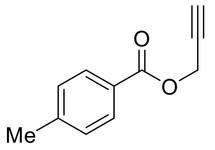

Synthesized according to ***general procedure b*** using *p*-toluoyl chloride (1.82 mL, 13.8 mmol, 1.1 equiv.). Recrystallisation from hexane by slow evaporation afforded the product as a white crystalline solid. Yield: 2.17 g, 12.5 mmol, 100%. Spectroscopic analyses agree with literature established values.[3] **^{1}H NMR** (500 MHz, $CDCl_3$, 298 K) δ/ppm: 7.96 (d, $^3J_{HH}$ = 8.1 Hz, 2H, aryl), 7.25 (d, $^3J_{HH}$ = 8.1 Hz, 2H, aryl), 4.91 (d, $^4J_{HH}$ = 2.5 Hz, 2H, CH_2), 2.51 (t, $^4J_{HH}$ = 2.5 Hz, 1H, ≡CH), 2.41 (s, 3H, CH_3).

Prop-2-yn-1-yl-4-methoxybenznoate

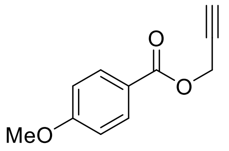

Synthesized according to ***general procedure c*** using *p*-anisoyl chloride (2.03 mL, 15 mmol, 1.2 equiv.). Purification *via* column chromatography (hexane/ethyl acetate, 5:1) gave the pure product as a colorless oil. Yield: 1.98 g, 10.4 mmol, 83%). Spectroscopic analyses agree with literature established values.[5] **^{1}H NMR** (500 MHz, $CDCl_3$, 298 K) δ/ppm: 8.02 (dt, $^3J_{HH}$ = 9.0 Hz, $^4J_{HH}$ = 2.2 Hz, 2H, aryl), 6.92 (dt, $^3J_{HH}$ = 9.0 Hz, $^4J_{HH}$ = 2.2 Hz, 2H, aryl), 4.89 (d, $^4J_{HH}$ = 2.4 Hz, 2H, CH_2), 3.86 (s, 3H, OCH_3), 2.50 (t, $^4J_{HH}$ = 2.4 Hz, 1H, ≡CH).

Prop-2-yn-1-yl-4-nitrobenznoate

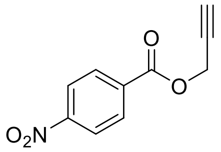

Synthesized according to ***general procedure c*** using *p*-nitrobenzoylchloride (2.77 g, 15 mmol, 1.2 equiv.). Recrystallisation *via* slow evaporation of CH_2Cl_2 gave the pure product as a yellow crystalline solid. Yield: 2.36 g, 11.5 mmol, 96%. Spectroscopic analyses agree with literature established values.[6] **^{1}H NMR** (500

MHz, $CDCl_3$, 298 K) δ/ppm: 8.30 (dt, $^3J_{HH}$ = 9.0 Hz, $^4J_{HH}$ = 2.2 Hz, 2H, aryl), 8.23 (dt, $^3J_{HH}$ = 9.0 Hz, $^4J_{HH}$ = 2.2 Hz, 2H, aryl), 4.97 (d, $^4J_{HH}$ = 2.5 Hz, 2H, CH_2), 2.56 (t, $^4J_{HH}$ = 2.5 Hz, 1H, ≡CH).

General procedure d: In accordance with the literature known procedure[7] the requisite aldehyde (10 mmol) was dissolved in CH_2Cl_2 (10 mL) along with 3 Å molecular sieves. To this the necessary amine (10 mmol) was added. The reaction was left at ambient temperature for 2 hours at which point $MgSO_4$ was added with subsequent filtration. Volatiles were removed *in vacuo* to leave the pure imine in quantitative yields.

(E)-N-phenyl-1-(p-tolyl)methanimine

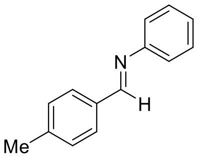

Synthesized in accordance with ***general procedure d*** using 4-tolualdehyde (1.18 mL, 10 mmol) and aniline (913 µL, 10 mmol). Spectroscopic analyses agree with literature values.[9] Yield: 1.8 g, 9.2 mmol, 92%. **^{1}H NMR** (500 MHz, $CDCl_3$, 298 K) δ/ppm: 8.42 (s, 1H, N=CH), 7.80 (d, $^3J_{HH}$ = 8.0 Hz, 2H, aryl), 7.39 (t, $^3J_{HH}$ = 7.8 Hz, 2H, aryl), 7.28 (d, $^3J_{HH}$ = 7.9 Hz, 2H, aryl), 7.22 (t, $^3J_{HH}$ = 9.1 Hz, 3H, aryl), 2.43 (s, 3H, CH_3).

(E)-1-mesityl-N-phenylmethanimine

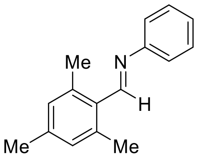

Synthesized in accordance with ***general procedure d*** using mesitaldehyde (1.47 mL, 10 mmol) and aniline (913 µL, 10 mmol). Spectroscopic analyses agree with literature values.[10] Yield: 2.0 g, 9.1 mmol, 91%. **^{1}H NMR** (500 MHz, $CDCl_3$, 298 K) δ/ppm: 8.80 (s, 1H, N=CH), 7.43 (t, $^3J_{HH}$ = 7.7 Hz, 2H, aryl), 7.23–7.06 (m, 3H, aryl), 6.95 (s, 2H, aryl), 2.56 (s, 6H, CH_3, mesityl), 2.35 (s, 3H, CH_3 mesityl).

(E)-1-(4-methoxyphenyl)-N-phenylmethanimine

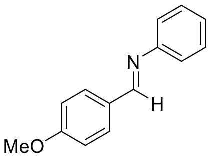

Synthesized in accordance with ***general procedure d*** using 4-anisaldehyde (1.22 mL, 10 mmol) and aniline (913 µL, 10 mmol). Spectroscopic analyses agree with literature values.[8] Yield: 2.0 mg, 9.4 mmol, 94%. **^{1}H NMR** (500 MHz, $CDCl_3$, 298 K) δ/ppm: 8.44 (s, 1H), 7.91 (d, $^3J_{HH}$ = 8.7 Hz, 2H, N=CH), 7.44 (t, $^3J_{HH}$ = 7.7 Hz, 2H, aryl), 7.30–7.14 (m, 3H, aryl), 7.04 (d, $^3J_{HH}$ = 8.7 Hz, 2H, aryl), 3.93 (s, 3H, OCH_3).

(E)-1-(2-methoxyphenyl)-N-phenylmethanimine

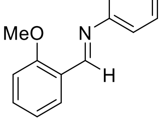

Synthesized in accordance with ***general procedure d*** using benzaldehyde (1.21 mL, 10 mmol) and aniline (913 µL, 10 mmol). Spectroscopic analyses agree with literature values.[11] Yield: 1.9 g, 9.0 mmol, 90%. **^{1}H NMR** (500 MHz, $CDCl_3$, 298 K) δ/ppm: 8.81 (s, 1H, N=CH), 8.05 (d, $^3J_{HH}$ = 5.3 Hz, 1H, aryl), 7.26 (s, 3H, aryl), 7.17–7.04 (m, 3H, aryl), 6.94–6.70 (m, 2H, aryl), 3.72 (s, 3H, OCH_3).

(E)-1-(4-fluorophenyl)-N-phenylmethanimine

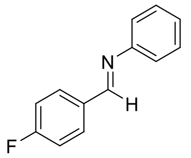

Synthesized in accordance with ***general procedure d*** using benzaldehyde (1.07 mL, 10 mmol) and aniline (913 µL, 10 mmol). Spectroscopic analyses agree with literature values.[11] Yield: 1.8 g, 9.1 mmol, 91%. **^{1}H NMR** (500 MHz, $CDCl_3$, 298 K) δ/ppm: 8.43 (s, 1H, N=CH), 7.91 (dd, $^3J_{HH}$ = 8.6, $^4J_{HH}$ = 5.6 Hz, 2H, aryl), 7.40 (t, $^3J_{HH}$ = 7.7 Hz, 2H, aryl), 7.30–7.05 (m, 6H, aryl).

(E)-1-(4-nitrophenyl)-N-phenylmethanimine

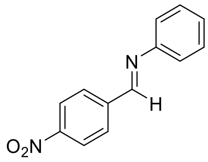

Synthesized in accordance with ***general procedure d*** using benzaldehyde (1.51 g, 10 mmol) and aniline (913 µL, 10 mmol). Spectroscopic analyses agree with literature values.[10] Yield: 2.1 g, 9.4 mmol, 94%. **^{1}H NMR** (500 MHz, $CDCl_3$, 298 K) δ/ppm: 8.49 (s, 1H, N=CH), 8.26 (d, $^3J_{HH}$ = 8.7 Hz, 2H, aryl), 8.01 (d, $^3J_{HH}$ = 8.7 Hz, 2H, aryl), 7.36 (t, $^3J_{HH}$ = 7.7 Hz, 2H, aryl), 7.28–7.17 (m, 3H, aryl).

(E)-1-(naphthalen-2-yl)-N-phenylmethanimine

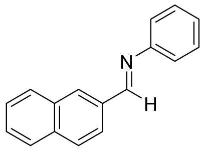

Synthesized in accordance with ***general procedure d*** using benzaldehyde (1.56 g, 10 mmol) and aniline (913 µL, 10 mmol). Spectroscopic analyses agree with literature values.[10] Yield: 2.1 g, 8.9 mmol, 89%. **^{1}H NMR** (500 MHz, $CDCl_3$, 298 K) δ/ppm: 8.52 (s, 1H, N=CH), 8.09 (dd, $^3J_{HH}$ = 10.4, $^4J_{HH}$ = 1.6 Hz, 2H, aryl), 7.81 (ddd, $^3J_{HH}$ = 8.9, $^3J_{HH}$ = 6.5 Hz, 3H, aryl), 7.49–7.41 (m, 2H, aryl), 7.38–7.28 (m, 2H, aryl), 7.21–7.13 (m, 3H, aryl).

(E)-N-butyl-1-phenylmethanimine

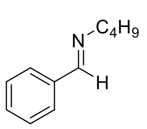

Synthesized in accordance with ***general procedure d*** using benzaldehyde (1.02 mL, 10 mmol) and *n*-butylamine (988 µL, 10 mmol). Spectroscopic analyses agree with literature values.[12] Yield: 1.5 g, 9.5 mmol, 95%. **^{1}H NMR** (500 MHz, $CDCl_3$, 298 K) δ/ppm: 8.19 (s, 1H, N=CH), 7.66–7.55 (m, 2H, aryl), 7.33–7.23 (m, 3H, aryl), 3.53 (t, $^3J_{HH}$ = 6.9 Hz, 2H, N–CH_2), 1.61 (p, $^3J_{HH}$ = 7.1 Hz, 2H, CH_2), 1.31 (h, $^3J_{HH}$ = 7.2 Hz, 2H, CH_2), 0.87 (t, $^3J_{HH}$ = 7.4 Hz, 3H, CH_3).

(E)-N-isopropyl-1-phenylmethanimine

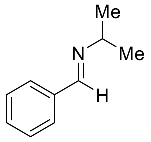

Synthesized in accordance with ***general procedure d*** using benzaldehyde (1.02 mL, 10 mmol) and isopropylamine (859 µL, 10 mmol). Spectroscopic analyses agree with literature values.[12] Yield: 1.3 g, 8.7 mmol, 87%. **^{1}H NMR** (500 MHz, $CDCl_3$, 298 K) δ/ppm: 8.31 (s, 1H, N=CH), 7.80–7.69 (m, 2H, aryl), 7.54–7.32 (m, 3H, aryl), 3.55 (sep, $^3J_{HH}$ = 5.2 Hz, 1H, *i*Pr H), 1.28 (d, $^3J_{HH}$ = 6.3 Hz, 6H, *i*Pr CH_3).

(E)-N-cyclopentyl-1-phenylmethanimine

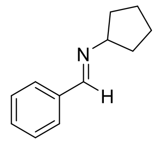

Synthesized in accordance with ***general procedure d*** using benzaldehyde (1.02 mL, 10 mmol) and cyclopentylamine (987 µL, 10 mmol). Spectroscopic analyses agree with literature values.[13] Yield: 1.5 g, 89 mmol, 89%. **^{1}H NMR** (500 MHz, $CDCl_3$, 298 K) δ/ppm: 8.29 (s, 1H, N=CH), 7.78–7.66 (m, 2H, aryl), 7.49–7.28 (m, 3H, aryl), 4.06–3.39 (m, 1H, N–CH), 1.88 (s, 4H, CH_2), 1.77–1.59 (m, 4H, CH_2).

(E)-N-benzyl-1-phenylmethanimine

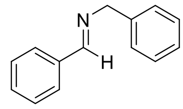

Synthesized in accordance with ***general procedure d*** using benzaldehyde (1.02 mL, 10 mmol) and benzylamine (1.09 mL, 10 mmol). Spectroscopic analyses agree with literature values.[8] Yield: 1.8 g, 9.2 mmol, 92%. **^{1}H NMR** (500 MHz, $CDCl_3$, 298 K) δ/ppm: 8.31 (s, 1H, N=CH), 7.77–7.60 (m, 2H, aryl), 7.33 (d, $^3J_{HH}$ = 4.9 Hz, 3H, aryl), 7.31–7.04 (m, 4H, aryl), 4.74 (s, 2H, CH_2Ph).

(E)-N-(2,6-diethylphenyl)-1-phenylmethanimine

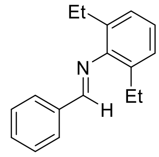

Synthesized in accordance with ***general procedure d*** using benzaldehyde (1.02 mL, 10 mmol) and 2,6-diethylaniline (1.65 mL, 10 mmol). Spectroscopic analyses agree with literature values.[14] Yield: 2.1 g, 8.7 µmol, 87%. **^{1}H NMR** (500 MHz, $CDCl_3$, 298 K) δ/ppm: 8.23 (s, 1H, N=CH), 7.92 (d, $^3J_{HH}$ = 6.9 Hz, 2H, aryl), 7.52 (d, $^3J_{HH}$ = 6.1 Hz, 3H, aryl), 7.11 (d, $^3J_{HH}$ = 7.4 Hz, 2H, aryl), 7.07–7.02 (m, 1H, aryl), 2.51 (q, $^3J_{HH}$ = 7.4 Hz, 4H, ethyl CH_2), 1.14 (t, $^3J_{HH}$ = 7.5 Hz, 6H, ethyl CH_3).

(E)-N-mesityl-1-phenylmethanimine

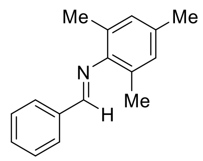

Synthesized in accordance with ***general procedure d*** using benzaldehyde (1.02 mL, 10 mmol) and 2,4,6-trimethylaniline (1.40 mL, 10 mmol). Spectroscopic analyses agree with literature values.[8] Yield: 2.1 g, 9.4 mmol, 94%. **^{1}H NMR** (500 MHz, $CDCl_3$, 298 K) δ/ppm: 8.16 (br. s, 1H, N=CH), 7.86 (br. s, 2H, aryl), 7.45 (br. s, 3H, aryl), 6.85 (br. s, 2H, aryl), 2.24 (s, 3H, *p*-CH_3), 2.08 (s, 6H, *o*-CH_3).

(E)-1-phenyl-N-(4-(trifluoromethyl)phenyl)methanimine

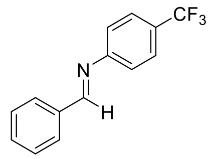

Synthesized in accordance with ***general procedure d*** using benzaldehyde (1.02 mL, 10 mmol) and 4-trifluoromethylaniline (1.26 mL, 10 mmol). Spectroscopic analyses agree with literature values.[15] Yield: 2.4 g, 9.1 mmol, 91%. **^{1}H NMR** (500 MHz, $CDCl_3$, 298 K) δ/ppm: 8.40 (s, 1H, N=CH), 7.88 (d, $^3J_{HH}$ = 7.4 Hz, 2H, aryl), 7.62 (d, $^3J_{HH}$ = 7.9 Hz, 2H, aryl), 7.47 (q, $^3J_{HH}$ = 7.8 Hz, 3H, aryl), 7.22 (d, $^3J_{HH}$ = 7.0 Hz, 2H, aryl).

(E)-N-(2-fluorophenyl)-1-phenylmethanimine

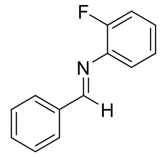

Synthesized in accordance with ***general procedure d*** using benzaldehyde (1.02 mL, 10 mmol) and 2-fluoroaniline (965 µL, 10 mmol). Spectroscopic analyses agree with literature values.[8] Yield: 1.8 g, 9.0 mmol, 90%. **^{1}H NMR** (500 MHz, $CDCl_3$, 298 K) δ/ppm: 8.53 (s, 1H, N=CH), 7.94 (dd, $^3J_{HH}$ = 7.4, $^4J_{HH}$ = 1.9 Hz, 2H, aryl), 7.52–7.45 (m, 3H, aryl), 7.19–7.13 (m, 4H, aryl).

1.3 General hydroboration

General procedure e: In an NMR tube, pinacol borane (31 mg, 0.22 mmol) and the substrate (aldehyde/imine) (0.2 mmol) were combined in CH_2Cl_2 (0.6 mL). To this, *tris*(2,4,6-trifluorophenyl)borane (2 mg, 2 mol%) was added, and the NMR tube sealed. Reactions occurred at 60 °C, while reaction times varied with substrate. Once conversion was complete, as measured by *in situ* ^{1}H NMR spectroscopy, the reaction mixtures were reduced under vacuum. The alkoxy/amino-pinacol boronates were redissolved in $CDCl_3$ for NMR spectroscopic analysis. Select examples were subsequently converted to alcohols/amines by hydrolysis as denoted in the text.

General procedure f: In an NMR tube, pinacol borane (31 mg, 0.22 mmol) and the substrate alkyne/alkene (0.2 mmol) were combined in CH_2Cl_2 (0.6 mL). To this, *tris*(2,4,6-trifluorophenyl)borane (2 mg, 2 mol%) was added, and the NMR tube sealed. Reactions occurred at 60 °C, while reaction times varied with substrate. Once the reaction was complete, as measured by *in situ* ^{1}H NMR spectroscopy, the reaction mixtures were concentrated under vacuum and subsequently passed through a short plug of silica in hexane. The pinacol boronates were redissolved in $CDCl_3$ for NMR spectroscopic analysis.

General procedure g: As per ***general procedure e*** and ***f***, at room temperature.

1.4 Hydroboration of alkynes

(E)-4,4,5,5-tetramethyl-2-styryl-1,3,2-dioxaborolane **1a**

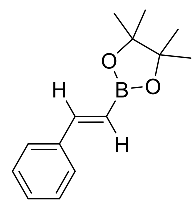

In accordance with ***general procedure f*** using phenylacetylene (20 mg, 0.2 mmol) as the substrate gave product **1a** as a colorless oil after 6 h. Yield: 47 mg, 198 µmol, 99%. **^{1}H NMR** (500 MHz, $CDCl_3$, 298 K) δ/ppm: 7.42 (d, $^3J_{HH}$ = 7.2 Hz, 2H, aryl), 7.33 (d, $^3J_{HH}$ = 18.5 Hz, 1H, =CH), 7.30–7.21 (m, 3H, aryl), 6.10 (d, $^3J_{HH}$ = 18.5 Hz, 1H, =CH), 1.24 (s, 12H, pinacol). **^{11}B NMR** (160 MHz, $CDCl_3$, 298 K) δ/ppm: 30.1 (s). **^{13}C NMR** (126 MHz, $CDCl_3$, 298 K) δ/ppm: 149.5 (s), 137.5 (s), 128.9 (s), 128.6 (s), 127.1 (s), 83.4 (s), 24.8 (s). **HRMS** $(ES)^+$ $[C_{14}H_{20}{}^{10}BO_2]^+$ $[M+H]^+$ *m/z* calculated: 230.1593, found: 230.1600.

(E)-2-(hex-1-en-1-yl)-4,4,5,5-tetramethyl-1,3,2-dioxaborolane **1b**

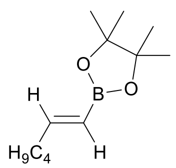

In accordance with ***general procedure g*** using 1-hexyne (16 mg, 0.2 mmol) as the substrate gave product **1b** as a colorless oil after 18 h. Yield: 30 mg, 142 µmol, 71%. Spectroscopic data agrees with literature values.[16] **^{1}H NMR** (500 MHz, $CDCl_3$, 298 K) δ/ppm: 6.63 (dt, $^3J_{HH}$ = 17.9 Hz, $^3J_{HH}$ = 6.4 Hz, 1H, =CH), 5.42 (dt, $^3J_{HH}$ = 17.9 Hz, $^4J_{HH}$ = 1.5 Hz, 1H, =CH), 2.17–2.12 (m, 2H, alkyl), 1.42–1.36 (m, 2H, alkyl), 1.35–1.29 (m, 2H, alkyl), 1.26 (s, 12H, pinacol), 0.88 (t, $^3J_{HH}$ = 7.2 Hz, 3H, alkyl). **^{11}B NMR** (160 MHz, $CDCl_3$, 298 K) δ/ppm: 29.7 (s). **^{13}C NMR** (126 MHz, $CDCl_3$, 298 K) δ/ppm: 154.8 (s), 83.0 (s), 35.5 (s), 30.4 (s), 24.8 (s), 22.3 (s), 13.9 (s).

(E)-4,4,5,5-tetramethyl-2-(oct-1-en-1-yl)-1,3,2-dioxaborolane **1c**

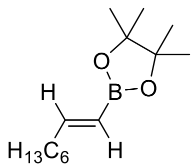

In accordance with ***general procedure g*** using 1-octyne (22 mg, 0.2 mmol) as the substrate gave product **1c** as a colorless oil after 24 h. Yield: 38 mg, 160 µmol, 80%. **^{1}H NMR** (500 MHz, $CDCl_3$, 298 K) δ/ppm: 6.63 (dt, $^3J_{HH}$ = 18.1 Hz, $^3J_{HH}$ = 6.4 Hz, 1H, =CH), 5.41 (d, $^3J_{HH}$ = 18.1 Hz, 1H, =CH), 2.14 (q, $^3J_{HH}$ = 6.9 Hz, 2H, CH_2), 1.40 (quintet, $^3J_{HH}$ = 7.2 Hz, 2H, CH_2), 1.32–1.20 (m, 6H, alkyl), 1.26 (s, 12H, pinacol), 0.87 (t, $^3J_{HH}$ = 6.6 Hz, 3H, CH_3). **^{11}B NMR** (160 MHz, $CDCl_3$, 298 K) δ/ppm: 29.8 (s). **^{13}C NMR** (126 MHz, $CDCl_3$, 298 K) δ/ppm: 154.9 (s), 83.0 (s), 35.9 (s), 31.7 (s), 28.9 (s), 28.2 (s), 24.8 (s), 22.6 (s), 14.1 (s). **HRMS** $(AP)^+$ $[C_{14}H_{28}{}^{10}BO_2]^+$ $[M+H]^+$ *m/z* calculated: 238.2219, found: 238.2226.

(E)-2-(dec-1-en-1-yl)-4,4,5,5-tetramethyl-1,3,2-dioxaborolane **1d**

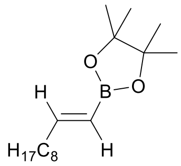

In accordance with ***general procedure g*** using 1-decyne (28 mg, 0.2 mmol) as the substrate gave product **1d** as a colorless oil after 18 h. Yield: 48 mg, 180 µmol, 90%. **^{1}H NMR** (500 MHz, $CDCl_3$, 298 K) δ/ppm: 6.62 (dt, $^3J_{HH}$ = 17.9 Hz, $^3J_{HH}$ = 6.4 Hz, 1H, =CH), 5.41 (dt, $^3J_{HH}$ = 17.9 Hz, $^4J_{HH}$ = 1.5 Hz, 1H, =CH), 2.15–2.11 (m, 2H, alkyl), 1.43–1.36 (m, 2H, alkyl), 1.29–1.22 (m, 10H, alkyl), 1.25 (s, 12H, pinacol), 0.87 (t, $^3J_{HH}$ = 7.0 Hz, 3H, alkyl). **^{11}B NMR** (160 MHz, $CDCl_3$, 298 K) δ/ppm: 29.7 (s). **^{13}C NMR** (126 MHz, $CDCl_3$, 298 K) δ/ppm: 153.8 (s), 82.0 (s), 34.8 (s), 30.9 (s), 28.4 (s), 28.2 (s), 27.2 (s), 23.8 (s), 21.7 (s), 13.1 (s). **HRMS** $(EI)^+$ $[C_{16}H_{31}{}^{10}BO_2]^+$ $[M]^+$ *m/z* calculated: 265.2453, found: 265.2448.

(E)-3-(4,4,5,5-tetramethyl-1,3,2-dioxaborolan-2-yl)allyl benzoate **1e**

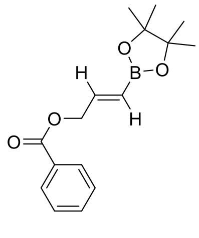

In accordance with ***general procedure f*** using prop-2-yn-1-yl benzoate (32 mg, 0.2 mmol) as the substrate gave product **1e** as a colorless oil after 18 h. Yield: 33 mg, 114 µmol, 57%. **^{1}H NMR** (500 MHz, $CDCl_3$, 298 K) δ/ppm: 8.03–7.96 (m, 2H, aryl), 7.48 (t, $^3J_{HH}$ = 7.4 Hz, 1H, aryl), 7.36 (q, $^3J_{HH}$ = 7.5 Hz, 2H, aryl), 6.66 (dt, $^3J_{HH}$ = 18.1, $^4J_{HH}$ = 4.4 Hz, 1H, =CH), 5.72 (dt, $^3J_{HH}$ = 18.1, $^4J_{HH}$ = 1.8 Hz, 1H, =CH),

4.87–4.81 (m, 1H, CH_2), 1.20 (s, 12H, pinacol). **^{11}B NMR** (160 MHz, $CDCl_3$, 298 K) δ/ppm: 29.6 (s). **^{13}C NMR** (126 MHz, $CDCl_3$, 298 K) δ/ppm: 166.1 (s), 146.0 (s), 133.1 (s), 129.8 (s), 129.7 (s), 128.5 (s), 128.4 (s), 83.5 (s), 75.0 (s), 65.7 (s), 52.5 (s), 24.8 (s). **HRMS** $(AP)^+$ $[C_{16}H_{22}{}^{11}BO_4]^+$ $[M+H]^+$ *m/z* calculated: 289.1611, found: 289.1597.

(E)-3-(4,4,5,5-tetramethyl-1,3,2-dioxaborolan-2-yl)allyl 4-methylbenzoate **1f**

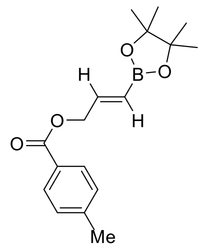

In accordance with ***general procedure f*** using prop-2-yn-1-yl 4-methylbenzoate (35 mg, 0.2 mmol) as the substrate gave product **1f** as a colorless oil after 24 h. Yield: 30 mg, 110 µmol, 55%. **^{1}H NMR** (500 MHz, $CDCl_3$, 298 K) δ/ppm: 7.89 (d, $^3J_{HH}$ = 8.2 Hz, 2H, aryl), 7.16 (d, $^3J_{HH}$ = 7.9 Hz, 2H, aryl), 6.66 (dt, $^3J_{HH}$ = 4.4 Hz, $^3J_{HH}$ = 18.2 Hz, 1H, =CH), 5.72 (d, $^3J_{HH}$ = 18.2 Hz, 1H, =CH), 4.83 (d, $^3J_{HH}$ = 6.0 Hz, 2H, CH_2), 2.34 (s, 3H, CH_3), 1.20 (s, 12H, pinacol). **^{11}B NMR** (160 MHz, $CDCl_3$, 298 K) δ/ppm: 29.7 (s). **^{13}C NMR** (126 MHz, $CDCl_3$, 298 K) δ/ppm: 166.2 (s), 146.2 (s), 143.8 (s), 129.8 (s), 129.1 (s), 127.2 (s), 83.5 (s), 74.9 (s), 65.6 (s), 52.3 (s), 24.8 (s), 21.7 (s). **HRMS** $(AP)^+$ $[C_{17}H_{24}{}^{10}BO_4]^+$ $[M+H]^+$ *m/z* calculated: 302.1804, found: 302.1792.

(E)-3-(4,4,5,5-tetramethyl-1,3,2-dioxaborolan-2-yl)allyl 4-nitrobenzoate **1g**

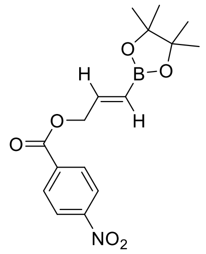

In accordance with ***general procedure f*** using prop-2-yn-1-yl 4-nitrobenzoate (41 mg, 0.2 mmol) as the substrate gave product **1g** as a colorless oil after 18 h. Yield: 51 mg, 154 µmol, 77%. **^{1}H NMR** (500 MHz, $CDCl_3$, 298 K) δ/ppm: 8.26–8.14 (m, 4H, aryl), 6.65 (dt, $^3J_{HH}$ = 18.1, $^3J_{HH}$ = 4.6 Hz, 1H, =CH), 5.72 (d, $^3J_{HH}$ = 18.2 Hz, 1H, =CH), 4.90 (dd, $^3J_{HH}$ = 8.2, $^4J_{HH}$ = 3.3 Hz, 2H, CH_2), 1.21 (s, 2H, pinacol). **^{11}B NMR** (160 MHz, $CDCl_3$, 298 K) δ/ppm: 29.6 (br. s). **^{13}C NMR** (126 MHz, $CDCl_3$, 298 K) δ/ppm: 164.1 (s), 145.1 (s), 134.9 (s), 131.1 (s), 123.8 (s), 83.7 (s), 75.9 (s), 66.8 (s), 53.4 (s), 24.9 (s).

(E)-3-(4,4,5,5-tetramethyl-1,3,2-dioxaborolan-2-yl)allyl 4-methoxybenzoate **1h**

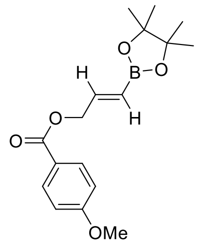

In accordance with ***general procedure f*** using prop-2-yn-1-yl 4-methoxybenzoate (38 mg, 0.2 mmol) as the substrate gave product **1h** as a colorless oil after 24 h. Yield: 44 mg, 138 µmol, 69%. **^{1}H NMR** (500 MHz, $CDCl_3$, 298 K) δ/ppm: 7.95 (dd, $^3J_{HH}$ = 8.7, $^4J_{HH}$ = 1.8 Hz, 2H, aryl), 6.84 (dd, $^3J_{HH}$ = 8.7, 2H, aryl), 6.65 (dt, $^3J_{HH}$ = 18.1, $^3J_{HH}$ = 4.3 Hz, 1H, =CH), 5.71 (d, $^3J_{HH}$ = 18.2 Hz, 1H, =CH), 4.81 (s, 2H, CH_2), 3.78 (s, 3H, OMe), 1.20 (s, 12H, pinacol). **^{11}B NMR** (160 MHz, $CDCl_3$, 298 K) δ/ppm: 29.6 (s). **^{13}C NMR** (126 MHz, $CDCl_3$, 298 K) δ/ppm:165.9 (s), 163.5 (s), 146.4 (s), 131.8 (s), 122.5 (s), 113.7 (s), 83.5 (s), 74.9 (s), 65.6 (s), 55.5 (s), 52.3 (s), 24.9 (s). **HRMS** $(AP)^+$ $[C_{17}H_{24}{}^{11}BO_5]^+$ $[M+H]^+$ *m/z* calculated: 319.1717, found: 319.1704.

(E)-3-(4,4,5,5-tetramethyl-1,3,2-dioxaborolan-2-yl)allyl acrylate **1i**

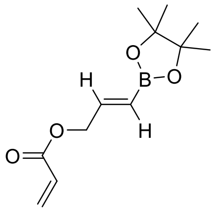

In accordance with ***general procedure f*** using prop-2-yn-1-yl acrylate (22 mg, 0.2 mmol) as the substrate gave product **1i** as a colorless oil after 24 h. Yield: 41 mg, 174 µmol, 87%. **^{1}H NMR** (400 MHz, $CDCl_3$, 298 K) δ/ppm: 6.64 (dt, $^{3}J_{HH}$ = 18.2 Hz, $^{4}J_{HH}$ = 4.6 Hz, 1H, =CH), 6.44 (dd, $^{3}J_{HH}$ = 17.3 Hz, $^{4}J_{HH}$ = 1.3 Hz, 1H, =CH), 6.14 (dd, $^{3}J_{HH}$ = 17.3 Hz, $^{3}J_{HH}$ = 10.4 Hz, 1H, =CH), 5.85 (dd, $^{3}J_{HH}$ = 10.5 Hz, $^{4}J_{HH}$ = 1.3 Hz, 1H, =CH), 5.70 (dt, $^{3}J_{HH}$ = 18.1 Hz, $^{4}J_{HH}$ = 1.7 Hz, 1H, =CH), 4.75 (dd, $^{3}J_{HH}$ = 4.6 Hz, $^{4}J_{HH}$ = 1.8 Hz, 2H, CH_2), 1.26 (s, 12H, pinacol). **^{11}B NMR** (128 MHz, $CDCl_3$, 298 K) δ/ppm: 29.6 (s). **^{13}C NMR** (101 MHz, $CDCl_3$, 298 K) δ/ppm: 164.6 (s), 144.8 (s), 130.1 (s), 127.2 (s), 82.4 (s), 64.4 (s), 23.8 (s).

(E)-trimethyl(2-(4,4,5,5-tetramethyl-1,3,2-dioxaborolan-2-yl)vinyl)silane **1j**

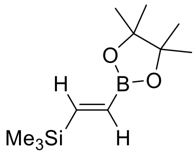

In accordance with ***general procedure f*** using ethynyltrimethylsilane (20 mg, 0.2 mmol) as the substrate gave product **1j** as a colorless oil after 6 h. Yield: 43 mg, 190 µmol, 95%. Spectroscopic data agrees with literature values.[17] **^{1}H NMR** (500 MHz, $CDCl_3$, 298 K) δ/ppm: 7.11 (d, $^{3}J_{HH}$ = 21.8 Hz, 2H, =CH), 6.23 (d, $^{3}J_{HH}$ = 21.8 Hz, 1H, =CH), 1.27 (s, 12H, pinacol), 0.06 (s, 9H, $SiMe_3$). **^{11}B NMR** (160 MHz, $CDCl_3$, 298 K) δ/ppm: 28.9 (s). **^{13}C NMR** (126 MHz, $CDCl_3$, 298 K) δ/ppm: 157.9 (s), 83.3 (s), 24.8 (s), -1.9 (s). **^{29}Si NMR** (80 MHz, $CDCl_3$, 298 K) δ/ppm: -6.7 (s).

(E)-trimethyl(3-(4,4,5,5-tetramethyl-1,3,2-dioxaborolan-2-yl)allyl)silane **1k**

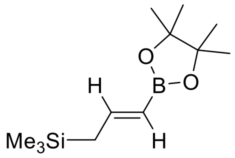

In accordance with ***general procedure f*** using 3-trimethylsilylpropyne (22 mg, 0.2 mmol) as the substrate gave product **1k** as colorless crystals after 6 h. Yield: 40 mg, 166 µmol, 83%. Spectroscopic data agrees with literature values.[18] **^{1}H NMR** (400 MHz, $CDCl_3$, 298 K) δ/ppm: 6.69–6.60 (m, 1H, =CH), 5.22 (d, $^{3}J_{HH}$ = 17.7 Hz, 1H, =CH), 1.67 (dd, $^{3}J_{HH}$ = 8.2 Hz, $^{4}J_{HH}$ = 1.0 Hz, 2H, CH_2), 1.24 (s, 12H, pinacol), 0.00 (s, 9H, $SiMe_3$). **^{11}B NMR** (128 MHz, $CDCl_3$, 298 K) δ/ppm: 29.6 (s). **^{13}C NMR** (101 MHz, $CDCl_3$, 298 K) δ/ppm: 154.0 (s), 84.6 (s), 30.1 (s), 26.6 (s), 0.00 (s). **^{29}Si NMR** (80 MHz, $CDCl_3$, 298 K) δ/ppm: 0.8 (s).

(E)-4,4,5,5-tetramethyl-2-(3-((3-phenylprop-2-yn-1-yl)oxy)prop-1-en-1-yl)-1,3,2-dioxaborolane **1l**

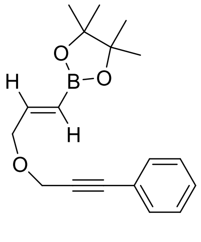

In accordance with ***general procedure f*** using (3-(prop-2-yn-1-yloxy)prop-1-yn-1-yl)benzene (34 mg, 0.2 mmol) as the substrate gave product **1l** as a colorless oil after 18 h. Yield: 33 mg, 112 µmol, 56%. **^{1}H NMR** (400 MHz, $CDCl_3$, 298 K) δ/ppm: 7.44 (dd, $^{3}J_{HH}$ = 6.6, $^{4}J_{HH}$ =3.2 Hz, 2H), 7.30 (dd, $^{3}J_{HH}$ = 5.1, $^{3}J_{HH}$ = 1.9 Hz, 2H), 6.67 (dt, $^{3}J_{HH}$ = 18.1, $^{4}J_{HH}$ =4.9 Hz, 1H), 5.76 (dt, $^{3}J_{HH}$ = 18.1, $^{4}J_{HH}$ =1.6 Hz, 1H), 4.38 (s, 1H), 4.22 (dd, $^{3}J_{HH}$ = 4.9, $^{4}J_{HH}$ = 1.6 Hz, 2H), 1.27 (s, 12H, pinacol). **^{11}B NMR** (128 MHz,

$CDCl_3$, 298 K) δ/ppm: 29.6 (s). **^{13}C NMR** (101 MHz, $CDCl_3$, 298 K) δ/ppm:148.5 (s), 131.9 (s), 128.5 (s), 128.4 (s), 122.8 (s), 86.5 (s), 85.1 (s), 83.4 (s), 71.4 (s), 58.4 (s), 24.9 (s). **HRMS** $(AP)^+$ $[C_{18}H_{24}{}^{10}BO_3]^+$ $[M+H]^+$ *m/z* calculated: 298.1855, found: 298.1849.

(Z)-4,4,5,5-tetramethyl-2-(1-phenylprop-1-en-2-yl)-1,3,2-dioxaborolane **1m**

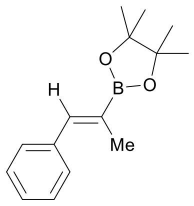

In accordance with ***general procedure f*** using 1-phenyl-1-propyne (23 mg, 0.2 mmol) as the substrate gave product **1m** as a colorless oil after 18 h. Yield: 47 mg, 192 µmol, 96%. **^{1}H NMR** (500 MHz, $CDCl_3$, 298 K) δ/ppm: 7.30 (d, $^3J_{HH}$ = 7.7 Hz, 2H, aryl), 7.25 (t, $^3J_{HH}$ = 7.4 Hz, 2H, aryl), 7.16 (s, 1H, =CH), 7.14 (t, $^3J_{HH}$ = 7.4 Hz, 1H, aryl), 1.91 (s, 3H, Me), 1.23 (s, 12H, pinacol). **^{11}B NMR** (160 MHz, $CDCl_3$, 298 K) δ/ppm: 30.6 (s). **^{13}C NMR** (126 MHz, $CDCl_3$, 298 K) δ/ppm: 141.4 (s), 136.9 (s), 128.4 (s), 127.0 (s), 126.1 (s), 82.5 (s), 23.8 (s), 14.9 (s). **HRMS** $(ES)^+$ $[C_{15}H_{22}{}^{10}BO_2]^+$ $[M+H]^+$ *m/z* calculated: 244.1749, found: 244.1739.

(Z)-2-(but-2-en-2-yl)-4,4,5,5-tetramethyl-1,3,2-dioxaborolane **1n**

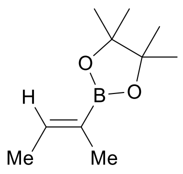

In accordance with ***general procedure f*** using 2-butyne (11 mg, 0.2 mmol) as the substrate gave product **1n** as a colorless oil after 6 h. Yield: 35 mg, 192 µmol, 96%. **^{1}H NMR** (500 MHz, $CDCl_3$, 298 K) δ/ppm: 6.42 (q, $^3J_{HH}$ = 6.5 Hz, 1H, =CH), 1.70 (d, $^3J_{HH}$ = 6.8 Hz, 3H, CH_3), 1.67 (s, 3H, CH_3), 1.25 (s, 12H, pinacol). **^{11}B NMR** (160 MHz, $CDCl_3$, 298 K) δ/ppm: 30.2 (s). **^{13}C NMR** (126 MHz, $CDCl_3$, 298 K) δ/ppm: 140.8 (s), 83.1 (s), 24.8 (s), 14.3 (s), 13.5 (s). **HRMS** $(EI)^+$ $[C_{10}H_{19}{}^{10}BO_2]^+$ $[M]^+$ *m/z* calculated: 181.1514, found: 181.1516.

(Z)-trimethyl(2-(4,4,5,5-tetramethyl-1,3,2-dioxaborolan-2-yl)prop-1-en-1-yl)silane **1o**

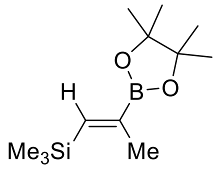

In accordance with ***general procedure f*** using 1-trimethylsilylpropyne (22 mg, 0.2 mmol) as the substrate gave product **1o** as a colorless oil after 18 h. Yield: 43 mg, 178 µmol, 89%. **^{1}H NMR** (400 MHz, $CDCl_3$, 298 K) δ/ppm: 7.09 (q, $^4J_{HH}$ = 6.7 Hz, 1H, =CH), 1.89 (d, $^4J_{HH}$ = 6.7 Hz, 3H, Me), 1.24 (s, 12H, pinacol), 0.16 (s, 9H, $SiMe_3$). **^{11}B NMR** (128 MHz, $CDCl_3$, 298 K) δ/ppm: 31.4 (s). **^{13}C NMR** (101 MHz, $CDCl_3$, 298 K) δ/ppm: 155.9 (s), 82.8 (s), 24.7 (s), 0.8 (s). **^{29}Si NMR** (80 MHz, $CDCl_3$, 298 K) δ/ppm: -8.44 (s).

(Z)-2-(1,2-diphenylvinyl)-4,4,5,5-tetramethyl-1,3,2-dioxaborolane **1p**

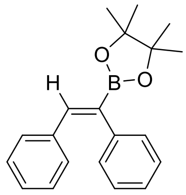

In accordance with ***general procedure f*** using diphenylacetylene (36 mg, 0.2 mmol) as the substrate gave product **1p** as white crystals after 168 h. Yield: 49 mg, 160 µmol, 80%. **^{1}H NMR** (500 MHz, $CDCl_3$, 298 K) δ/ppm: 7.37 (br. s, 1H, aryl), 7.29–7.21 (m, 3H, aryl), 7.20–7.14 (m, 2H, aryl), 7.14–7.07 (m, 3H, aryl and =CH), 7.09–7.02 (m, 2H, aryl), 1.30 (s, 12H, pinacol). **^{11}B NMR** (160 MHz, $CDCl_3$, 298 K) δ/ppm: 30.6 (s). **^{13}C**

NMR (126 MHz, $CDCl_3$, 298 K) δ/ppm: 143.2 (s), 140.5 (s), 137.0 (s), 131.6 (s), 130.0 (s), 128.9 (s), 128.3 (s), 127.9 (s), 126.3 (s), 83.8 (s), 24.8 (s). **HRMS** $(EI)^+$ $[C_{20}H_{23}{}^{10}BO_2]^+$ $[M]^+$ *m/z* calculated: 305.1827, found: 305.1831.

1.5 Hydroboration of aldehydes.

2-(benzyloxy)-4,4,5,5-tetramethyl-1,3,2-dioxaborolane **2a**

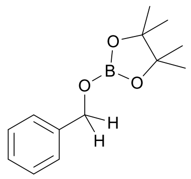

In accordance with ***general procedure g*** using benzaldehyde (21 mg, 0.2 mmol) as the substrate gave product **2a** as a colorless oil after 1 h. Conversion >99%. Spectroscopic data agrees with literature values.[19] ^{1}**H NMR** (500 MHz, $CDCl_3$, 298 K) δ/ppm: 7.28–7.17 (m, 5H, aryl), 4.85 (s, 2H, CH_2), 1.18 (s, 12H, pinacol). ^{11}**B NMR** (160 MHz, $CDCl_3$, 298 K) δ/ppm: 22.3 (s). ^{13}**C NMR** (126 MHz, $CDCl_3$, 298 K) δ/ppm: 139.2 (s), 128.3 (s), 127.4 (s), 126.7 (s), 83.0 (s), 66.7 (s), 24.6 (s).

4,4,5,5-tetramethyl-2-((4-methylbenzyl)oxy)-1,3,2-dioxaborolane **2b**

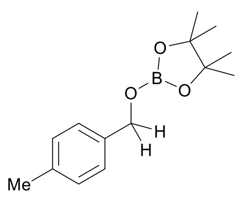

In accordance with ***general procedure e*** using 4-methylbenzaldehyde (24 mg, 0.2 mmol) as the substrate gave product **2b** as a colorless oil after 3 h. Conversion >99%. ^{1}**H NMR** (500 MHz, $CDCl_3$, 298 K) δ/ppm: 7.16 (d, $^3J_{HH}$ = 8.0 Hz, 2H, aryl), 7.06 (d, $^3J_{HH}$ = 8.0 Hz, 2H, aryl), 4.81 (s, 2H, CH_2), 2.25 (s, 3H, CH_3), 1.18 (s, 12H, pinacol). ^{11}**B NMR** (160 MHz, $CDCl_3$, 298 K) δ/ppm: 22.3 (s). ^{13}**C NMR** (126 MHz, $CDCl_3$, 298 K) δ/ppm: 137.0 (s), 136.3 (s), 129.0 (s), 126.9 (s), 82.9 (s), 66.6 (s), 24.6 (s), 21.2 (s). **HRMS** $(EI)^+$ $[C_{14}H_{21}{}^{10}BO_3]^+$ $[M]^+$ *m/z* calculated: 247.1620, found: 247.1614.

4,4,5,5-tetramethyl-2-((2,4,6-trimethylbenzyl)oxy)-1,3,2-dioxaborolane **2c**.

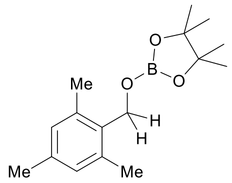

In accordance with ***general procedure e*** using mesitaldehyde (29 mg, 0.2 mmol) as the substrate gave product **2c** as a colorless oil after 3 h. Conversion >99%. Spectroscopic data agrees with literature values.[20] ^{1}**H NMR** (500 MHz, $CDCl_3$, 298 K) δ/ppm: 6.75 (s, 2H, aryl), 4.87 (s, 2H, CH_2), 2.30 (s, 6H, *o*-Me), 2.17 (s, 3H, *p*-Me) 1.18 (s, 12H, pinacol). ^{11}**B NMR** (160 MHz, $CDCl_3$, 298 K) δ/ppm: 22.3 (s). ^{13}**C NMR** (126 MHz, $CDCl_3$, 298 K) δ/ppm: 137.8 (s), 137.6 (s), 132.2 (s), 128.9 (s), 82.8 (s), 61.2 (s), 22.6 (s), 21.0 (s), 19.5 (s).

2-((4-fluorobenzyl)oxy)-4,4,5,5-tetramethyl-1,3,2-dioxaborolane **2d**

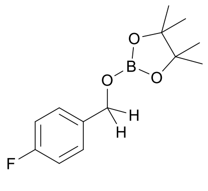

In accordance with ***general procedure e*** using 4-fluorobenzaldehyde (25 mg, 0.2 mmol) as the substrate gave product **2d** as a colorless oil after 3 h. Conversion >99%. ^{1}**H NMR** (500 MHz, $CDCl_3$, 298 K) δ/ppm: 7.26–7.22 (m, 2H, aryl), 6.96–6.91 (m, 2H, aryl), 4.80 (s, 2H, CH_2), 1.19 (s, 12H, pinacol). ^{11}**B NMR** (160 MHz,

$CDCl_3$, 298 K) δ/ppm: 22.3 (s). **^{13}C NMR** (126 MHz, $CDCl_3$, 298 K) δ/ppm: 162.2 (d, $^1J_{CF}$ = 245 Hz, C-F), 135.0 (d, $^4J_{CF}$ = 3.1 Hz), 128.6 (d, $^3J_{CF}$ = 8.1 Hz), 115.1 (d, $^2J_{CF}$ = 21.4 Hz), 83.1 (s), 66.1 (s), 24.6 (s). **^{19}F NMR** (565 MHz, $CDCl_3$, 298 K) δ/ppm: -115.3 (s, 1F). **HRMS** $(EI)^+$ $[C_{13}H_{18}{}^{10}BO_3F]^+$ $[M]^+$ *m/z* calculated: 251.1369, found: 251.1363.

2-((4-bromobenzyl)oxy)-4,4,5,5-tetramethyl-1,3,2-dioxaborolane **2e**

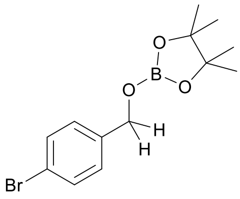

In accordance with ***general procedure e*** using 4-bromobenzaldehyde (37 mg, 0.2 mmol) as the substrate gave product **2e** as a colorless oil after 3 h. Conversion >99%. **^{1}H NMR** (500 MHz, $CDCl_3$, 298 K) δ/ppm: 7.38 (d, $^3J_{HH}$ = 7.6 Hz, 2H, aryl), 7.14 (d, $^3J_{HH}$ = 7.6 Hz, 2H, aryl), 4.79 (s, 2H, CH_2), 1.19 (s, 1H, pinacol). **^{11}B NMR** (160 MHz, $CDCl_3$, 298 K) δ/ppm: 22.3 (s). **^{13}C NMR** (126 MHz, $CDCl_3$, 298 K) δ/ppm: 138.2 (s), 131.4 (s), 128.4 (s), 121.2 (s), 83.2 (s), 66.0 (s), 24.6 (s). **HRMS** $(EI)^+$ $[C_{13}H_{18}O_3Br^{10}B]^+$ $[M]^+$ *m/z* calculated: 311.0569, found: 311.0573.

2-((4-methoxybenzyl)oxy)-4,4,5,5-tetramethyl-1,3,2-dioxaborolane **2f**

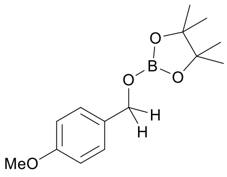

In accordance with ***general procedure e*** using 4-methoxybenzaldehyde (27 mg, 0.2 mmol) as the substrate gave product **2f** as a colorless oil after 18 h. Conversion >99%. **^{1}H NMR** (500 MHz, $CDCl_3$, 298 K) δ/ppm: 7.20 (d, $^3J_{HH}$ = 8.3 Hz, 2H, aryl), 6.79 (d, $^3J_{HH}$ = 8.3 Hz, 2H, aryl), 4.77 (s, 2H, CH_2), 3.72 (s, 3H, OMe), 1.18 (s, 12H, pinacol). **^{11}B NMR** (160 MHz, $CDCl_3$, 298 K) δ/ppm: 22.3 (s). **^{13}C NMR** (126 MHz, $CDCl_3$, 298 K) δ/ppm: 158.0 (s), 127.5 (s), 112.6 (s), 81.9 (s), 65.4 (s), 54.2 (s), 23.6 (s). **HRMS** $(EI)^+$ $[C_{14}H_{21}{}^{10}BO_4]^+$ $[M]^+$ *m/z* calculated: 263.1569, found: 263.1565.

4,4,5,5-tetramethyl-2-((4-nitrobenzyl)oxy)-1,3,2-dioxaborolane **2g**

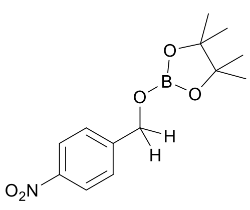

In accordance with ***general procedure e*** using 4-nitrobenzaldehyde (30 mg, 0.2 mmol) as the substrate gave product **2g** as a colorless oil after 48 h. Conversion >99%. Spectroscopic data agrees with literature values.[19] **^{1}H NMR** (500 MHz, $CDCl_3$, 298 K) δ/ppm: 8.12 (d, $^3J_{HH}$ = 8.8 Hz, 2H, aryl), 7.43 (d, $^3J_{HH}$ = 8.8 Hz, 2H, aryl), 4.95 (s, 2H, CH_2), 1.20 (s, 12H, pinacol). **^{11}B NMR** (160 MHz, $CDCl_3$, 298 K) δ/ppm: 22.4 (s). **^{13}C NMR** (126 MHz, $CDCl_3$, 298 K) δ/ppm: 146.2 (s), 145.6 (s), 125.8 (s), 122.6 (s), 82.4 (s), 64.5 (s), 23.6 (s).

4,4,5,5-tetramethyl-2-((4-(trifluoromethyl)benzyl)oxy)-1,3,2-dioxaborolane **2h**

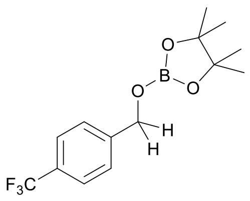

In accordance with ***general procedure e*** using 4-(trifluoromethyl)benzaldehyde (35 mg, 0.2 mmol) as the substrate gave product **2h** as a colorless oil after 3 h. Conversion >99%. Spectroscopic data agrees with literature values.[20] **^{1}H NMR** (500 MHz, $CDCl_3$, 298 K) δ/ppm: 7.51 (d, $^{3}J_{HH}$ = 8.0 Hz, 2H, aryl), 7.37 (d, $^{3}J_{HH}$ = 8.0 Hz, 2H, aryl), 4.90 (s, 2H, CH_2), 1.19 (s, 12H, pinacol). **^{11}B NMR** (160 MHz, $CDCl_3$, 298 K) δ/ppm: 22.4 (s). **^{13}C NMR** (126 MHz, $CDCl_3$, 298 K) δ/ppm: 143.2 (s), 129.6 (q, $^{2}J_{CF}$ = 32.3 Hz), 126.6 (s), 125.3 (q, $^{3}J_{CF}$ = 3.7 Hz), 83.2 (s), 65.9 (s), 24.6 (s). **^{19}F NMR** (471 MHz, $CDCl_3$, 298 K) δ/ppm: -62.5 (s, 3F, *p*-CF_3).

2-((2-bromobenzyl)oxy)-4,4,5,5-tetramethyl-1,3,2-dioxaborolane **2i**

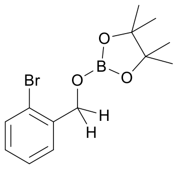

In accordance with ***general procedure g*** using 2-bromobenzaldehyde (37 mg, 0.2 mmol) as the substrate gave product **2i** as a colorless oil after 18 h. Conversion >99%. **^{1}H NMR** (500 MHz, $CDCl_3$, 298 K) δ/ppm: 7.43 (d, $^{3}J_{HH}$ = 7.9 Hz, 2H, aryl), 7.23 (t, $^{3}J_{HH}$ = 7.5 Hz, 1H, aryl), 7.05 (t, $^{3}J_{HH}$ = 7.7 Hz, 1H, aryl), 4.90 (s, 2H, CH_2), 1.20 (s, 12H, pinacol). **^{11}B NMR** (160 MHz, $CDCl_3$, 298 K) δ/ppm: 22.4 (s) **^{13}C NMR** (126 MHz, $CDCl_3$, 298 K) δ/ppm: 138.4 (s), 132.3 (s), 128.6 (s), 127.8 (s), 127.4 (s), 121.6 (s), 83.2 (s), 66.3 (s), 24.6 (s). **HRMS** $(EI)^+$ $[C_{13}H_{18}O_3Br^{10}B]^+$ $[M]^+$ *m/z* calculated: 311.0569, found: 311.0564.

2-((2-methoxybenzyl)oxy)-4,4,5,5-tetramethyl-1,3,2-dioxaborolane **2j**

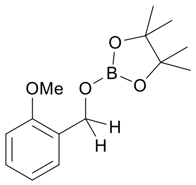

In accordance with ***general procedure e*** using 2-methoxybenzaldehyde (27 mg, 0.2 mmol) as the substrate gave product **2j** as a colorless oil after 24 h. Conversion >99%. **^{1}H NMR** (500 MHz, $CDCl_3$, 298 K) δ/ppm: 7.32 (d, $^{3}J_{HH}$ = 7.5 Hz, 1H, aryl), 7.15 (t, $^{3}J_{HH}$ = 7.9 Hz, 1H, aryl), 6.86 (t, $^{3}J_{HH}$ = 7.4 Hz, 1H, aryl), 6.74 (d, $^{3}J_{HH}$ = 8.2 Hz, 1H, aryl), 4.90 (s, 2H, CH_2), 3.71 (s, 3H, OMe), 1.18 (s, 12H, pinacol). **^{11}B NMR** (160 MHz, $CDCl_3$, 298 K) δ/ppm: 22.4 (s). **^{13}C NMR** (126 MHz, $CDCl_3$, 298 K) δ/ppm: 156.5 (s), 128.3 (s), 127.7 (s), 127.4 (s), 120.4 (s), 109.8 (s), 82.9 (s), 62.3 (s), 55.2 (s), 24.6 (s). **HRMS** $(EI)^+$ $[C_{14}H_{21}{}^{10}BO_4]^+$ $[M]^+$ *m/z* calculated: 263.1569, found: 263.1559.

4,4,5,5-tetramethyl-2-((2-nitrobenzyl)oxy)-1,3,2-dioxaborolane **2k**

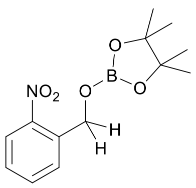

In accordance with ***general procedure e*** using 2-nitrobenzaldehyde (30 mg, 0.2 mmol) as the substrate gave product **2k** as a colorless oil after 48 h. Conversion >99%. Compound was allowed to hydrolyze, and subsequent filtration through silica afforded a clean NMR spectra of the hydrolyzed alcohol product. Spectroscopic data agrees with literature values.[21] **^{1}H NMR** (500 MHz, $CDCl_3$, 298 K) δ/ppm: 8.01 (d, $^{3}J_{HH}$ = 8.2 Hz, 1H, aryl), 7.68 (d, $^{3}J_{HH}$ = 7.6 Hz, 1H, aryl), 7.58 (t, $^{3}J_{HH}$ = 7.5 Hz, 1H, aryl), 7.39 (t, $^{3}J_{HH}$ = 7.8 Hz, 1H, aryl),

4.91 (d, $^3J_{HH}$ = 5.2 Hz, 1H, CH_2), 2.49 (br. s, 1H, OH). **^{13}C NMR** (126 MHz, $CDCl_3$, 298 K) δ/ppm: 136.8 (s), 134.2 (s), 130.0 (s), 128.5 (s), 125.1 (s), 62.6 (s).

2-(((4,4,5,5-tetramethyl-1,3,2-dioxaborolan-2-yl)oxy)methyl)benzonitrile **2l**

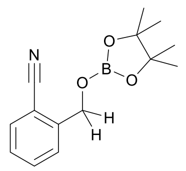

In accordance with ***general procedure e*** using 2-cyanobenzaldehyde (26 mg, 0.2 mmol) as the substrate gave product **2l** as a colorless oil after 4 h. Conversion >99%. **^{1}H NMR** (500 MHz, $CDCl_3$, 298 K) δ/ppm: 7.56–7.50 (m, 3H, aryl), 7.29 (t, $^3J_{HH}$ = 7.4 Hz, 1H, aryl), 5.60 (s, 2H, CH_2), 1.20 (s, 12H, pinacol). **^{11}B NMR** (160 MHz, $CDCl_3$, 298 K) δ/ppm: 22.4 (s). **^{13}C NMR** (126 MHz, $CDCl_3$, 298 K) δ/ppm: 142.8 (s), 132.9 (s), 132.6 (s), 127.8 (s), 127.4 (s), 117.0 (s), 83.4 (s), 64.4 (s), 24.6 (s). **HRMS** $(AP)^+$ $[C_8H_8NO]^+$ $[M+2H-(C_6H_{12}BO_2)]^+$ *m/z* calculated: 134.0606, found: 134.0600.

4,4,5,5-tetramethyl-2-((perfluorophenyl)methoxy)-1,3,2-dioxaborolane **2m**

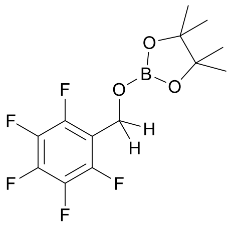

In accordance with ***general procedure g*** using 2,3,4,5,6-pentaflourobenzaldehyde (39 mg, 0.2 mmol) as the substrate gave product **2m** as a colorless oil after 1 h. Conversion >99%. Spectroscopic data agrees with literature values.[19] **^{1}H NMR** (500 MHz, $CDCl_3$, 298 K) δ/ppm: 5.00 (s, 2H, CH_2), 1.27 (s, 12H, pinacol). **^{11}B NMR** (160 MHz, $CDCl_3$, 298 K) δ/ppm: 22.3 (s). **^{13}C NMR** (126 MHz, $CDCl_3$, 298 K) δ/ppm: 146.6–144.4 (m), 142.4–140.2 (m), 138.5–136.3 (m), 112.1 (td, $^2J_{CF}$ = 17.7 Hz, $^3J_{CF}$ = 3.8 Hz), 83.5 (s), 54.2 (s), 24.5 (s). **^{19}F NMR** (471 MHz, $CDCl_3$, 298 K) δ/ppm: -143.1–-143.2 (m, 2F, *o*-F), -154.1 (t, 1F, *p*-F), -162.3–-162.4 (m, 2F, *m*-F).

4,4,5,5-tetramethyl-2-(naphthalen-2-ylmethoxy)-1,3,2-dioxaborolane **2n**

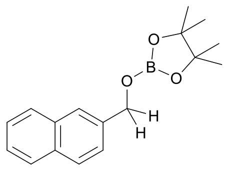

In accordance with ***general procedure e*** using 2-napthaldehyde (31 mg, 0.2 mmol) as the substrate gave product **2n** as a colorless oil after 18 h. Conversion >99%. **^{1}H NMR** (500 MHz, $CDCl_3$, 298 K) δ/ppm: 7.76–7.69 (m, 4H, aryl), 7.40–7.31 (m, 3H, aryl), 5.01 (s, 2H, CH_2), 1.19 (s, 12H, pinacol). **^{11}B NMR** (160 MHz, $CDCl_3$, 298 K) δ/ppm: 22.4 (s). **^{13}C NMR** (126 MHz, $CDCl_3$, 298 K) δ/ppm: 136.7 (s), 133.4 (s), 132.9 (s), 128.0 (s), 127.9 (s), 127.7 (s), 126.1 (s), 125.8 (s), 125.2 (s), 124.9 (s), 83.1 (s), 66.8 (s), 24.7 (s). **HRMS** $(EI)^+$ $[C_{17}H_{21}{}^{10}BO_3]^+$ $[M]^+$ *m/z* calculated: 283.1620, found: 283.1620.

3-(((4,4,5,5-tetramethyl-1,3,2-dioxaborolan-2-yl)oxy)methyl)pyridine **2o**

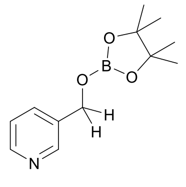

In accordance with ***general procedure e*** using 3-pyridinecarboxaldehyde (21 mg, 0.2 mmol) as the substrate gave product **2o** as a colorless oil after 24 h. Conversion >99%. **^{1}H NMR** (500 MHz, $CDCl_3$, 298 K) δ/ppm: 8.49 (s, 1H, aryl), 8.42 (d, $^3J_{HH}$ = 3.8 Hz, 1H, aryl), 7.65 (d, $^3J_{HH}$ = 7.8 Hz, 1H, aryl), 7.21 (t, $^3J_{HH}$ = 7.5 Hz, 1H, aryl), 4.85 (s, 2H, CH_2), 1.18 (s, 12H, pinacol). **^{11}B NMR** (160 MHz, $CDCl_3$, 298 K) δ/ppm: 22.3 (s). **^{13}C NMR** (126

MHz, $CDCl_3$, 298 K) δ/ppm: 148.5 (s), 148.1 (s), 135.1 (s), 123.5 (s), 83.3 (s), 82.7 (s), 64.3 (s), 24.6 (s). **HRMS** $(AP)^+$ $[C_6H_8NO]^+$ $[M+2H-(C_6H_{12}BO_2)]^+$ *m/z* calculated: 110.0606, found: 110.0602.

2-(furan-2-ylmethoxy)-4,4,5,5-tetramethyl-1,3,2-dioxaborolane **2p**

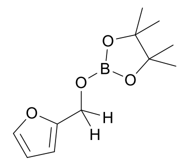

In accordance with ***general procedure e*** using 2-furanaldehyde (19 mg, 0.2 mmol) as the substrate gave product **2p** as a colorless oil after 18 h. Conversion >99%. Spectroscopic data agrees with literature values.[22] ^{1}**H NMR** (500 MHz, $CDCl_3$, 298 K) δ/ppm: 7.30–7.29 (m, 1H, furan), 6.25–6.21 (m, 2H, furan), 4.75 (s, 2H, CH_2), 1.19 (s, 12H, pinacol). ^{11}**B NMR** (160 MHz, $CDCl_3$, 298 K) δ/ppm: 22.3 (s). ^{13}**C NMR** (126 MHz, $CDCl_3$, 298 K) δ/ppm: 152.5 (s), 142.5 (s), 110.2 (s), 108.3 (s), 83.1 (s), 59.2 (s), 24.6 (s).

2-ethoxy-4,4,5,5-tetramethyl-1,3,2-dioxaborolane **2q**

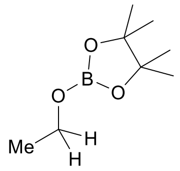

In accordance with ***general procedure g*** using acetaldehyde (9 mg, 0.2 mmol) as the substrate gave product **2q** as a colorless oil after 1 h. Conversion >99%. Spectroscopic data agrees with literature values.[23] ^{1}**H NMR** (400 MHz, $CDCl_3$, 298 K) δ/ppm: 3.89 (q, $^3J_{HH}$ = 7.0 Hz, 2H, CH_2), 1.24 (s, 12H, pinacol), 1.21 (t, $^3J_{HH}$ = 7.1 Hz, 3H, Me), ^{11}**B NMR** (128 MHz, $CDCl_3$, 298 K) δ/ppm: 22.1 (s). ^{13}**C NMR** (101 MHz, $CDCl_3$, 298 K) δ/ppm: 83.2 (s), 60.7 (s), 24.5 (s), 17.2 (s).

4,4,5,5-tetramethyl-2-(pentyloxy)-1,3,2-dioxaborolane **2r**

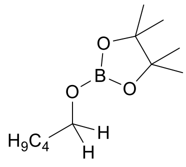

In accordance with ***general procedure g*** using 1-pentanal (17 mg, 0.2 mmol) as the substrate gave product **2r** as a colorless oil after 1 h. Conversion >99%. Spectroscopic data agrees with literature values.[24] ^{1}**H NMR** (500 MHz, $CDCl_3$, 298 K) δ/ppm: 3.76 (t, $^3J_{HH}$ = 6.6 Hz, 2H, CH_2), 1.50 (p, $^3J_{HH}$ = 6.5 Hz, 2H, CH_2), 1.30–1.23 (m, 4H, CH_2), 1.19 (d, $^3J_{HH}$ = 8.1 Hz, 12H), 0.83 (q, $^3J_{HH}$ = 7.3 Hz, 3H, CH_3). ^{11}**B NMR** (160 MHz, $CDCl_3$, 298 K) δ/ppm: 22.1 (s). ^{13}**C NMR** (126 MHz, $CDCl_3$, 298 K) δ/ppm: 82.6 (s), 65.0 (s), 31.1 (s), 27.8 (s), 24.6 (s), 22.4 (s), 14.0 (s).

4,4,5,5-tetramethyl-2-(neopentyloxy)-1,3,2-dioxaborolane **2s**

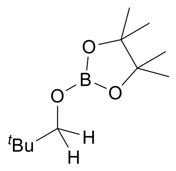

In accordance with ***general procedure g*** using trimethylacetaldehyde (17 mg, 0.2 mmol) as the substrate gave product **2s** as a colorless oil after 18 h. Conversion >99%. Spectroscopic data agrees with literature values.[20] ^{1}**H NMR** (400 MHz, $CDCl_3$, 298 K) δ/ppm: 3.51 (s, 2H, CH_2), 1.24 (s, 12H, pinacol), 0.88 (9H, s, *t*-butyl). ^{11}**B NMR** (128 MHz, $CDCl_3$, 298 K) δ/ppm: 22.1 (s). ^{13}**C NMR** (101 MHz, $CDCl_3$, 298 K) δ/ppm: 82.6 (s), 74.9 (s), 32.3 (s), 26.0 (s), 24.6 (s).

2-(cyclohexylmethoxy)-4,4,5,5-tetramethyl-1,3,2-dioxaborolane **2t**

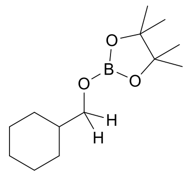

In accordance with ***general procedure g*** using cyclohexanaldehyde (22 mg, 0.2 mmol) as the substrate gave product **2t** as a colorless oil after 18 h. Conversion >99%. Spectroscopic data agrees with literature values.[23] **^{1}H NMR** (400 MHz, $CDCl_3$, 298 K) δ/ppm: 3.63 (d, $^{3}J_{HH}$ = 6.4 Hz, 2H, CH_2), 1.75–1.62 (m, 5H, alkyl), 1.54–1.45 (m, 1H, alkyl), 1.20 (s, 12H, pinacol), 1.19–1.10 (m, 3H, alkyl), 0.96–0.87 (m, 2H, alkyl). **^{11}B NMR** (128 MHz, $CDCl_3$, 298 K) δ/ppm: 22.1 (s). **^{13}C NMR** (101 MHz, $CDCl_3$, 298 K) δ/ppm: 82.6 (s), 70.4 (s), 39.3 (s), 29.3 (s), 26.5 (s), 25.8 (s), 24.6 (s).

1.6 Hydroboration of imines.

N-benzyl-4,4,5,5-tetramethyl-N-phenyl-1,3,2-dioxaborolan-2-amine **3a**

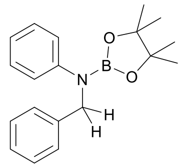

In accordance with ***general procedure e*** using (*E*)-*N*,1-diphenylmethanimine (36 mg, 0.2 mmol) as the substrate gave product **3a** as a colorless oil after 4 h. Conversion >99%. **^{1}H NMR** (500 MHz, $CDCl_3$, 298 K) δ/ppm: 7.27–7.23 (m, 4H, aryl), 7.18 (t, $^{3}J_{HH}$ = 6.7 Hz, 1H, aryl), 7.08 (t, $^{3}J_{HH}$ = 7.3 Hz, 2H, aryl), 6.65 (t, $^{3}J_{HH}$ = 7.2 Hz, 1H, aryl), 6.56 (d, $^{3}J_{HH}$ = 8.0 Hz, 2H, aryl), 4.24 (s, 2H, CH_2), 1.18 (s, 12H, pinacol). **^{11}B NMR** (160 MHz, $CDCl_3$, 298 K) δ/ppm: 21.1 (s). **^{13}C NMR** (126 MHz, $CDCl_3$, 298 K) δ/ppm: 129.3 (s), 128.7 (s), 127.6 (s), 127.3 (s), 83.2 (s), 48.7 (s), 24.6 (s). **HRMS** $(ES)^+$ $[C_{19}H_{25}{}^{10}BNO_2]^+$ $[M+H]^+$ *m/z* calculated: 309.2015, found: 309.2019.

4,4,5,5-tetramethyl-N-(4-methylbenzyl)-N-phenyl-1,3,2-dioxaborolan-2-amine **3b**

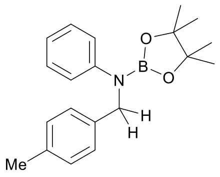

In accordance with ***general procedure e*** using (*E*)-*N*-phenyl-1-(p-tolyl)methanimine (39 mg, 0.2 mmol) as the substrate gave product **3b** as a colorless oil after 18 h. Conversion >99%. **^{1}H NMR** (500 MHz, $CDCl_3$, 298 K) δ/ppm: 7.17 (d, $^{3}J_{HH}$ = 7.9 Hz, 2H, aryl), 7.10–7.04 (m, 4H, aryl), 6.62 (t, $^{3}J_{HH}$ = 7.3 Hz, 1H, aryl), 6.54 (d, $^{3}J_{HH}$ = 7.8 Hz, 2H, aryl), 4.18 (s, 2H, CH2), 2.25 (s, 3H, *o*-Me), 1.18 (s, 12H, pinacol). **^{11}B NMR** (160 MHz, $CDCl_3$, 298 K) δ/ppm: 21.1 (s). **^{13}C NMR** (126 MHz, $CDCl_3$, 298 K) δ/ppm: 148.3 (s), 136.9 (s), 136.4 (s), 129.4 (s), 129.3 (s), 127.6 (s), 117.5 (s), 112.9 (s), 83.2 (s), 48.1 (s), 24.6 (s), 21.2 (s). **HRMS** $(EI)^+$ $[C_{20}H_{26}{}^{10}BNO_2]^+$ $[M]^+$ *m/z* calculated: 322.2093, found: 322.2094; $[C_{14}H_{15}N]^+$ $[M]^+$ *m/z* calculated: 197.1204, found: 197.1206.

4,4,5,5-tetramethyl-N-phenyl-N-(2,4,6-trimethylbenzyl)-1,3,2-dioxaborolan-2-amine **3c**

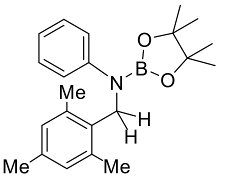

In accordance with ***general procedure e*** using (*E*)-1-mesityl-*N*-phenylmethanimine (44 mg, 0.2 mmol) as the substrate gave product **3c** as a colorless oil after 18 h. Conversion >99%. **^{1}H NMR** (500 MHz, $CDCl_3$, 298 K) δ/ppm: 7.12 (t, $^{3}J_{HH}$ = 7.3 Hz, 2H, aryl), 6.80 (s, 2H, aryl), 6.64 (t, $^{3}J_{HH}$ = 7.2 Hz, 1H, aryl), 6.57 (d, $^{3}J_{HH}$ = 7.7 Hz, 2H, aryl), 4.09 (s, 2H, CH_2), 2.25 (s, 6H, *o*-Me), 2.20 (s, 3H, *p*-Me),

1.18 (s, 12H, pinacol). **^{11}B NMR** (160 MHz, $CDCl_3$, 298 K) δ/ppm: 22.3 (s). **^{13}C NMR** (126 MHz, $CDCl_3$, 298 K) δ/ppm: 148.6 (s), 137.5 (s), 137.3 (s), 132.2 (s), 129.3 (s), 129.1 (s), 117.4 (s), 112.6 (s), 83.3 (s), 42.5, 24.5, 21.0, 19.5. **HRMS** $(EI)^+$ $[C_{16}H_{19}N]^+$ $[M+2H-(C_6H_{12}BO_2)]^+$ *m/z* calculated: 225.1517, found: 225.1517.

N-(4-methoxybenzyl)-4,4,5,5-tetramethyl-N-phenyl-1,3,2-dioxaborolan-2-amine **3d**

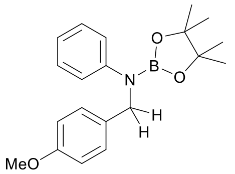

In accordance with ***general procedure e*** using (*E*)-1-(4-methoxyphenyl)-*N*-phenylmethanimine (42 mg, 0.2 mmol) as the substrate gave product **3d** as a colorless oil after 18 h. Conversion >99%. Compound was allowed to hydrolyze, and subsequent filtration through silica afforded clean NMR spectra of the secondary amine. Spectroscopic data agrees with literature values.[25] **^{1}H NMR** (500 MHz, $CDCl_3$, 298 K) δ/ppm: 7.30 (d, $^3J_{HH}$ = 8.5 Hz, 2H, aryl), 7.20–7.16 (m, 2H, aryl), 6.89 (d, $^3J_{HH}$ = 8.6 Hz, 2H, aryl), 6.72 (t, $^3J_{HH}$ = 7.3 Hz, 1H, aryl), 6.65 (d, $^3J_{HH}$ = 7.7 Hz, 2H, aryl), 4.26 (s, 2H, CH_2), 3.95 (br. s, 1H, NH), 3.81 (s, 3H, OMe). **^{13}C NMR** (126 MHz, $CDCl_3$, 298 K) δ/ppm: 158.9 (s), 148.2 (s), 131.4 (s), 129.3 (s), 128.8 (s), 117.5 (s), 114.0 (s), 112.9 (s), 55.3 (s), 47.8 (s).

N-(2-methoxybenzyl)-4,4,5,5-tetramethyl-N-phenyl-1,3,2-dioxaborolan-2-amine **3e**

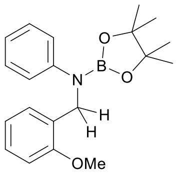

In accordance with ***general procedure e*** using (*E*)-1-(2-methoxyphenyl)-*N*-phenylmethanimine (42 mg, 0.2 mmol) as the substrate gave product **3e** as a colorless oil after 4 h. Conversion >99%. Compound was allowed to hydrolyze, and subsequent filtration through silica afforded clean NMR spectra of the secondary amine. **^{1}H NMR** (500 MHz, $CDCl_3$, 298 K) δ/ppm: 7.23 (d, $^3J_{HH}$ = 7.2 Hz, 1H, aryl), 7.20–7.13 (m, 1H, aryl), 7.13–7.05 (m, 2H, aryl), 6.87–6.76 (m, 2H, aryl), 6.67–6.53 (m, 3H, aryl), 4.26 (s, 2H, CH_2), 3.78 (s, 3H, OCH_3). **^{13}C NMR** (126 MHz, $CDCl_3$, 298 K) δ/ppm: 157.4 (s), 148.4 (s), 129.2 (s), 128.9 (s), 128.3 (s), 127.3 (s), 120.6 (s), 117.4 (s), 113.1 (s), 110.3 (s), 55.3 (s), 43.5 (s). **HRMS** $(ES)^+$ $[C_{14}H_{16}NO]^+$ $[M+H]^+$ *m/z* calculated: 214.1232, found: 214.1236.

N-(4-fluorobenzyl)-4,4,5,5-tetramethyl-N-phenyl-1,3,2-dioxaborolan-2-amine **3f**

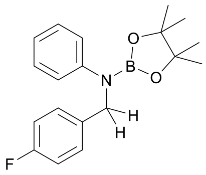

In accordance with ***general procedure g*** using (*E*)-1-(4-fluorophenyl)-*N*-phenylmethanimine (40 mg, 0.2 mmol) as the substrate gave product **3f** as a colorless oil after 18 h. Conversion >99%. Compound was allowed to hydrolyze, and subsequent filtration through silica afforded clean NMR spectra of the secondary amine. **^{1}H NMR** (500 MHz, $CDCl_3$, 298 K) δ/ppm: 7.34 (dd, $^3J_{HH}$ = 8.4 Hz, $^3J_{HH}$ = 5.5 Hz, 2H, aryl), 7.18 (t, $^3J_{HH}$ = 7.9 Hz, 2H, aryl), 7.03 (t, $^3J_{HH}$ = 8.7 Hz, 2H, aryl), 6.73 (t, $^3J_{HH}$ = 7.3 Hz, 1H, aryl), 6.63 (t, $^3J_{HH}$ = 7.8 Hz, 2H, aryl), 4.31 (s, 2H, CH2), 4.02 (br. s, 1H, NH). **^{13}C NMR** (126 MHz, $CDCl_3$, 298 K) δ/ppm: 162.2 (d, $^1J_{CF}$ = 245 Hz), 148.1 (s), 135.2 (d, $^4J_{CF}$ = 3.1 Hz), 129.4 (s), 129.1 (d,

$^3J_{CF}$ = 8.0 Hz), 117.9 (s), 115.6 (d, $^2J_{CF}$ = 21.4 Hz), 113.0 (s), 47.8 (s). **^{19}F NMR** (377 MHz, $CDCl_3$, 298 K) δ/ppm: -115.7 (s, 1F, *p*-F). **HRMS** $(ES)^+$ $[C_{13}H_{13}NF]^+$ $[M+H]^+$ *m/z* calculated: 202.1032, found: 202.1024.

4,4,5,5-tetramethyl-N-(4-nitrobenzyl)-N-phenyl-1,3,2-dioxaborolan-2-amine **3g**

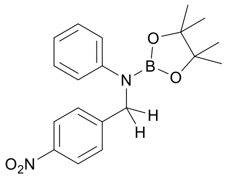

In accordance with ***general procedure g*** using (*E*)-1-(4-nitrophenyl)-N-phenylmethanimine (45 mg, 0.2 mmol) as the substrate gave product **3g** as a colorless oil after 24 h. Conversion >99%. Compound was allowed to hydrolyze, and subsequent filtration through silica afforded clean NMR spectra of the secondary amine. **^{1}H NMR** (500 MHz, $CDCl_3$, 298 K) δ/ppm: 8.19 (d, $^3J_{HH}$ = 8.7 Hz, 2H, aryl), 7.54 (d, $^3J_{HH}$ = 8.6 Hz, 2H, aryl), 7.18 (t, $^3J_{HH}$ = 7.9 Hz, 2H, aryl), 6.75 (t, $^3J_{HH}$ = 7.3 Hz, 1H, aryl), 6.59 (t, $^3J_{HH}$ = 8.4 Hz, 2H, aryl), 4.48 (s, 2H, CH_2), 4.26 (br. s, 1H, NH). **^{13}C NMR** (126 MHz, $CDCl_3$, 298 K) δ/ppm:147.5 (s), 147.3 (s), 129.4 (s), 127.7 (s), 123.9 (s), 121.0 (s), 118.2 (s), 112.9 (s), 47.6 (s). **HRMS** $(ES)^+$ $[C_{13}H_{13}N_2O_2]^+$ $[M+H]^+$ *m/z* calculated: 229.0977, found: 229.0966.

4,4,5,5-tetramethyl-N-(naphthalen-2-ylmethyl)-N-phenyl-1,3,2-dioxaborolan-2-amine **3h**

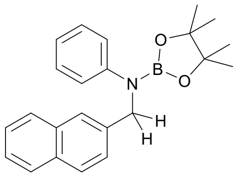

In accordance with ***general procedure e*** using (*E*)-1-(naphthalen-2-yl)-N-phenylmethanimine (46 mg, 0.2 mmol) as the substrate gave product **3h** as a colorless oil after 24 h. Conversion >99%. **^{1}H NMR** (500 MHz, $CDCl_3$, 298 K) δ/ppm: 7.73–7.68 (m, 4H, aryl), 7.37–7.35 (m, 3H, aryl), 7.08 (t, $^3J_{HH}$ = 7.9 Hz, 2H, aryl), 6.65 (t, $^3J_{HH}$ = 7.3 Hz, 1H, aryl), 6.58 (d, $^3J_{HH}$ = 8.0 Hz, 2H, aryl), 3.38 (s, 2H, CH_2), 1.17 (s, 12H, pinacol). **^{11}B NMR** (160 MHz, $CDCl_3$, 298 K) δ/ppm: 22.3 (s). **^{13}C NMR** (126 MHz, $CDCl_3$, 298 K) δ/ppm: 133.5 (s), 132.8 (s), 129.3 (s), 128.4 (s), 127.8 (s), 127.7 (s), 126.2 (s), 125.8 (s), 125.8 (s), 83.2 (s), 48.9 (s), 24.6 (s). **HRMS** $(EI)^+$ $[C_{17}H_{15}N]^+$ $[M+H-(C_6H_{12}BO_2)]^+$ *m/z* calculated: 233.1204, found: 233.1208.

N-benzyl-N-butyl-4,4,5,5-tetramethyl-1,3,2-dioxaborolan-2-amine **3i**

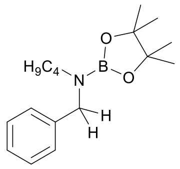

In accordance with ***general procedure e*** using (*E*)-*N*-butyl-1-phenylmethanimine (32 mg, 0.2 mmol) as the substrate gave product **3i** as a colorless oil after 18 h. Conversion >99%. **^{1}H NMR** (500 MHz, $CDCl_3$, 298 K) δ/ppm: 7.25–7.08 (m, 5H, aryl), 4.00 (s, 2H, CH_2), 2.70 (t, $^3J_{HH}$ = 6.9 Hz, 2H, alkyl CH_2), 1.34–1.23 (m, 4H, alkyl CH_2), 1.17 (s, 12H, pinacol), 0.79 (t, $^3J_{HH}$ = 7.3 Hz, 3H, CH_3). **^{11}B NMR** (160 MHz, $CDCl_3$, 298 K) δ/ppm: 24.6 (s). **^{13}C NMR** (126 MHz, $CDCl_3$, 298 K) δ/ppm: 141.0 (s), 128.3 (s), 127.8 (s), 126.6 (s), 82.2 (s), 49.1 (s), 44.4 (s), 30.8 (s), 24.7 (s), 19.8 (s), 14.1 (s). **HRMS** $(ES)^+$ $[C_{11}H_{18}N]^+$ $[M+2H-(C_6H_{12}BO_2)]^+$ *m/z* calculated: 164.1439, found: 164.1435.

N-benzyl-N-isopropyl-4,4,5,5-tetramethyl-1,3,2-dioxaborolan-2-amine **3j**

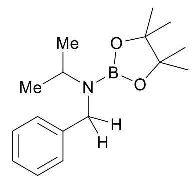

In accordance with ***general procedure e*** using (*E*)-*N*-isopropyl-1-phenylmethanimine (29 mg, 0.2 mmol) as the substrate gave product **3j** as a colorless oil after 18 h. Conversion >99%. **^{1}H NMR** (500 MHz, $CDCl_3$, 298 K) δ/ppm: 7.19 (br. s, 4H, aryl), 7.10 (br. s, 1H, aryl), 4.01 (s, 2H, CH_2), 3.27 (sept, $^3J_{HH}$ = 7.3 Hz, 1H, *i*Pr H), 1.17 (s, 12H, pinacol), 0.93 (d, $^3J_{HH}$ = 6.6 Hz, 6H, *i*Pr CH_3). **^{11}B NMR** (160 MHz, $CDCl_3$, 298 K) δ/ppm: 24.6 (s). **^{13}C NMR** (126 MHz, $CDCl_3$, 298 K) δ/ppm: 143.2 (s), 128.1 (s), 127.4 (s), 126.3 (s), 81.9 (s), 48.1 (s), 48.0 (s), 24.7 (s), 22.7 (s). **HRMS** (EI)$^+$ $[C_{17}H_{16}N]^+$ $[M+2H-(C_6H_{12}BO_2]^+$ *m/z* calculated: 150.1283, found: 150.1278.

N-benzyl-N-cyclopentyl-4,4,5,5-tetramethyl-1,3,2-dioxaborolan-2-amine **3k**

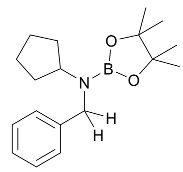

In accordance with ***general procedure e*** using (*E*)-*N*-cyclopentyl-1-phenylmethanimine (35 mg, 0.2 mmol) as the substrate gave product **3k** as a colorless oil after 6 h. Conversion >99%. **^{1}H NMR** (500 MHz, $CDCl_3$, 298 K) δ/ppm: 7.29–7.21 (m, 4H, aryl), 7.20–7.10 (m, 1H, aryl), 4.10 (s, 2H, CH_2), 3.53–3.36 (m, 1H, NCH cyclopentyl), 1.61–1.36 (m, 8H, cyclopentyl), 1.22 (s, 12H, pinacol). **^{11}B NMR** (160 MHz, $CDCl_3$, 298 K) δ/ppm: 24.6 (s). **^{13}C NMR** (126 MHz, $CDCl_3$, 298 K) δ/ppm: 143.0 (s), 128.1 (s) (s), 127.0 (s), 126.2 (s), 81.9 (s), 58.9 (s), 48.7 (s), 31.2 (s), 24.7 (s), 23.7 (s). **HRMS** (ES)$^+$ $[C_{12}H_{18}N]^+$ $[M+2H-(C_6H_{12}BO_2)]^+$ *m/z* calculated: 176.1439, found: 176.1446.

N,N-dibenzyl-4,4,5,5-tetramethyl-1,3,2-dioxaborolan-2-amine **3l**

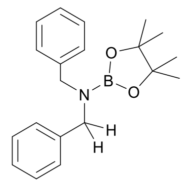

In accordance with ***general procedure e*** using (*E*)-*N*-benzyl-1-phenylmethanimine (39 mg, 0.2 mmol) as the substrate gave product **3l** as a colorless oil after 18 h. Conversion >99%. **^{1}H NMR** (500 MHz, $CDCl_3$, 298 K) δ/ppm: 7.22 (dd, $^3J_{HH}$ = 7.9, $^3J_{HH}$ = 6.4 Hz, 4H, aryl), 7.14 (td, $^3J_{HH}$ = 8.2, $^4J_{HH}$ = 4.1 Hz, 6H), 3.85 (s, 4H, CH_2), 1.22 (s, 12H, pinacol). **^{11}B NMR** (160 MHz, $CDCl_3$, 298 K) δ/ppm: 24.9 (s). **^{13}C NMR** (126 MHz, $CDCl_3$, 298 K) δ/ppm: 140.2, 128.4, 128.1, 126.8, 82.7, 48.5, 24.8. **HRMS** (ES)$^+$ $[C_{14}H_{16}N]^+$ $[M+2H-(C_6H_{12}BO_2)]^+$ *m/z* calculated: 198.1283, found: 198.1288.

N-benzyl-N-(2,6-diethylphenyl)-4,4,5,5-tetramethyl-1,3,2-dioxaborolan-2-amine **3m**

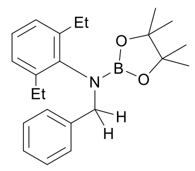

In accordance with ***general procedure g*** using (*E*)-*N*-(2,6-diethylphenyl)-1-phenylmethanimine (47 mg, 0.2 mmol) as the substrate gave product **3m** as a colorless oil after 18 h. Conversion >99%. **^{1}H NMR** (500 MHz, $CDCl_3$, 298 K) δ/ppm: 7.09 (s, 3H, aryl), 7.01–6.92 (m, 5H, aryl), 4.15 (s, 2H, CH_2), 2.35–2.31 (m, 2H, CH_2), 2.06–2.01 (m, 2H, CH_2), 1.21 (br. s, 6H, pinacol), 1.07 (br. s, 6H, pinacol), 0.91 (t, $^3J_{HH}$ = 7.1 Hz, 6H, CH_3). **^{11}B NMR** (160 MHz, $CDCl_3$, 298 K) δ/ppm: 24.1 (s). **^{13}C NMR** (126 MHz, $CDCl_3$, 298 K) δ/ppm: 142.6 (s), 140.8 (s), 140.0 (s), 129.6 (s), 127.9 (s), 126.9 (s), 126.2 (s), 126.1 (s), 82.6

(s), 54.9 (s), 24.6 (s), 23.9 (s), 14.8 (s). **HRMS** (EI)$^+$ [$C_{23}H_{32}{}^{10}BNO_2$]$^+$ [M]$^+$ *m/z* calculated: 364.2562, found: 364.2556.

N-benzyl-N-mesityl-4,4,5,5-tetramethyl-1,3,2-dioxaborolan-2-amine **3n**

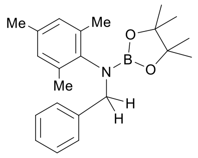

In accordance with ***general procedure g*** using (*E*)-*N*-mesityl-1-phenylmethanimine (45 mg, 0.2 mmol) as the substrate gave product **3n** as a colorless oil after 18 h. Conversion >99%. **^{1}H NMR** (500 MHz, $CDCl_3$, 298 K) δ/ppm: 7.11–7.03 (m, 3H, aryl), 7.02–7.01 (m, 2H, aryl), 6.68 (s, 2H, aryl), 4.12 (s, 2H, CH_2), 2.12 (s, 3H, *p*-Me) 1.78 (s, 6H, *o*-Me), 1.23 (br. s, 6H, pinacol), 1.09 (br. s, 6H, pinacol). **^{11}B NMR** (160 MHz, $CDCl_3$, 298 K) δ/ppm: 23.8 (s). **^{13}C NMR** (126 MHz, $CDCl_3$, 298 K) δ/ppm: 140.6 (s), 139.7 (s), 136.3 (s), 134.9 (s), 129.5 (s), 129.0 (s), 128.0 (s), 126.8 (s), 82.5 (s), 53.7 (s), 24.6 (s), 21.0 (s), 18.2 (s). **HRMS** (ES)$^+$ [$C_{22}H_{30}{}^{10}BNO_2$]$^+$ [M]$^+$ *m/z* calculated: 350.2406, found: 350.2411; **HRMS** (ES)$^+$ [$C_{16}H_{19}N$]$^+$ [M+H-($C_6H_{12}BO_2$)]$^+$ *m/z* calculated: 225.1517, found: 225.1517.

N-benzyl-4,4,5,5-tetramethyl-N-(4-(trifluoromethyl)phenyl)-1,3,2-dioxaborolan-2-amine **3o**

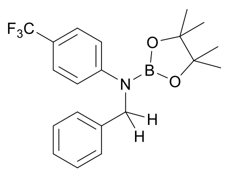

In accordance with ***general procedure g*** using (*E*)-1-phenyl-*N*-(4-(trifluoromethyl)phenyl)methanimine (50 mg, 0.2 mmol) as the substrate gave product **3o** as a colorless oil after 18 h. Conversion >99%. **^{1}H NMR** (400 MHz, $CDCl_3$, 298 K) δ/ppm: 7.30 (d, 2H, $^3J_{HH}$ = 8.5), 7.27–7.26 (m, 5H, aryl), 6.54 (d, $^3J_{HH}$ = 8.5 Hz, 2H, Aryl), 4.27 (s, 2H, CH_2), 1.19 (s, 12H, pinacol). **^{11}B NMR** (128 MHz, $CDCl_3$, 298 K) δ/ppm: 22.3 (s). **^{13}C NMR** (101 MHz, $CDCl_3$, 298 K) δ/ppm: 150.1 (s), 138.1 (s), 130.2 (s), 128.8 (s), 127.5 (s), 127.4 (s), 126.6 (q, $^4J_{CF}$ = 3.8 Hz), 125.1 (q, $^1J_{CF}$ = 270 Hz), 119.0 (q, $^2J_{CF}$ = 32.6 Hz), 83.3 (s), 47.8 (s), 24.5 (s). **^{19}F NMR** (377 MHz, $CDCl_3$, 298 K) δ/ppm: -61.0 (s, 3F, CF_3). **HRMS** (ES)$^+$ [$C_{14}H_{13}NF_3$]$^+$ [M+H-($C_6H_{12}BO_2$)]$^+$ *m/z* calculated: 252.1000, found: 252.1001.

N-benzyl-N-(2-fluorophenyl)-4,4,5,5-tetramethyl-1,3,2-dioxaborolan-2-amine **3p**

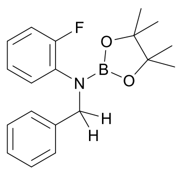

In accordance with ***general procedure g*** using (*E*)-*N*-(2-fluorophenyl)-1-phenylmethanimine (40 mg, 0.2 mmol) as the substrate gave product **3p** as a colorless oil after 24 h. Conversion >99%. Compound was allowed to hydrolyze, and subsequent filtration through silica afforded clean NMR spectra of the secondary amine. **^{1}H NMR** (500 MHz, $CDCl_3$, 298 K) δ/ppm: 7.31–7.15 (m, 5H, aryl), 6.92–6.85 (m, 2H, aryl), 6.61–6.52 (m, 2H, aryl), 4.28 (s, 2H, CH_2), 4.24 (br. s, 1H, NH). **^{13}C NMR** (126 MHz, $CDCl_3$, 298 K) δ/ppm: 151.6 (d, $^1J_{CF}$ = 238 Hz), 139.1 (s), 136.7 (d, $^3J_{CF}$ = 11.5 Hz), 128.8 (s), 127.5 (s), 124.7 (d, $^4J_{CF}$ = 3.5 Hz), 116.9 (d, $^3J_{CF}$ = 7.0 Hz), 114.5 (d, $^2J_{CF}$ = 18.4 Hz), 112.4 (s), 48.0 (s). **^{19}F NMR** (377 MHz, $CDCl_3$, 298 K) δ/ppm: -136.5 (s, 1F). **HRMS** (ES)$^+$ [$C_{13}H_{13}NF$]$^+$ [M+H-($C_6H_{12}BO_2$]$^+$ *m/z* calculated: 202.1032, found: 202.1026.

2. Experimental: NMR spectra

2.1 NMR spectra of starting materials.

S1 ^{1}H NMR (500 MHz, $CDCl_3$, 298 K) spectrum of *tris(2,6-difluorophenyl)borane.*

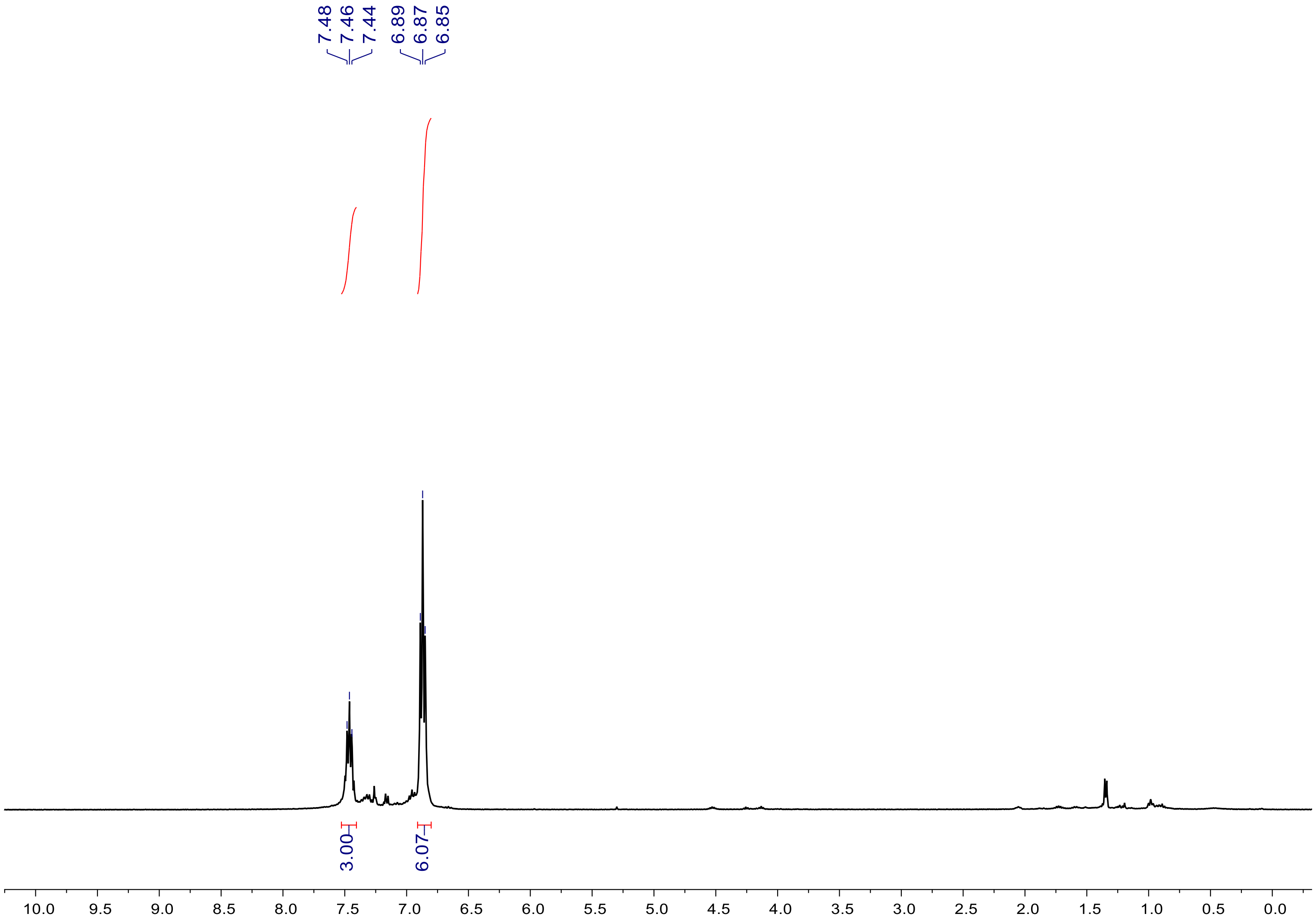

S2 ^{11}B NMR (160 MHz, $CDCl_3$, 298 K) spectrum of *tris(2,6-difluorophenyl)borane*.

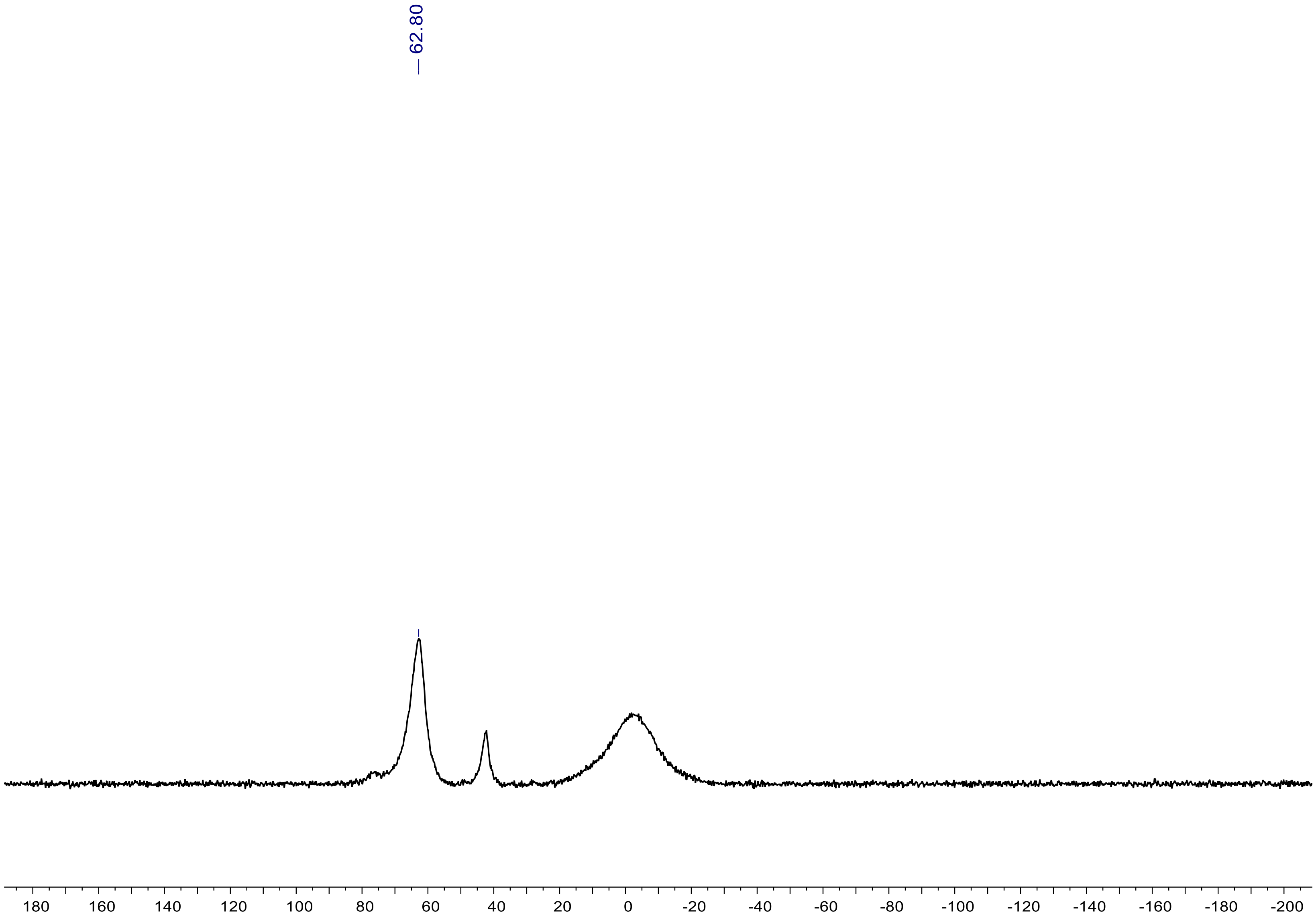

S3 ^{19}F NMR (471 MHz, $CDCl_3$, 298 K) spectrum of *tris(2,6-difluorophenyl)borane*.

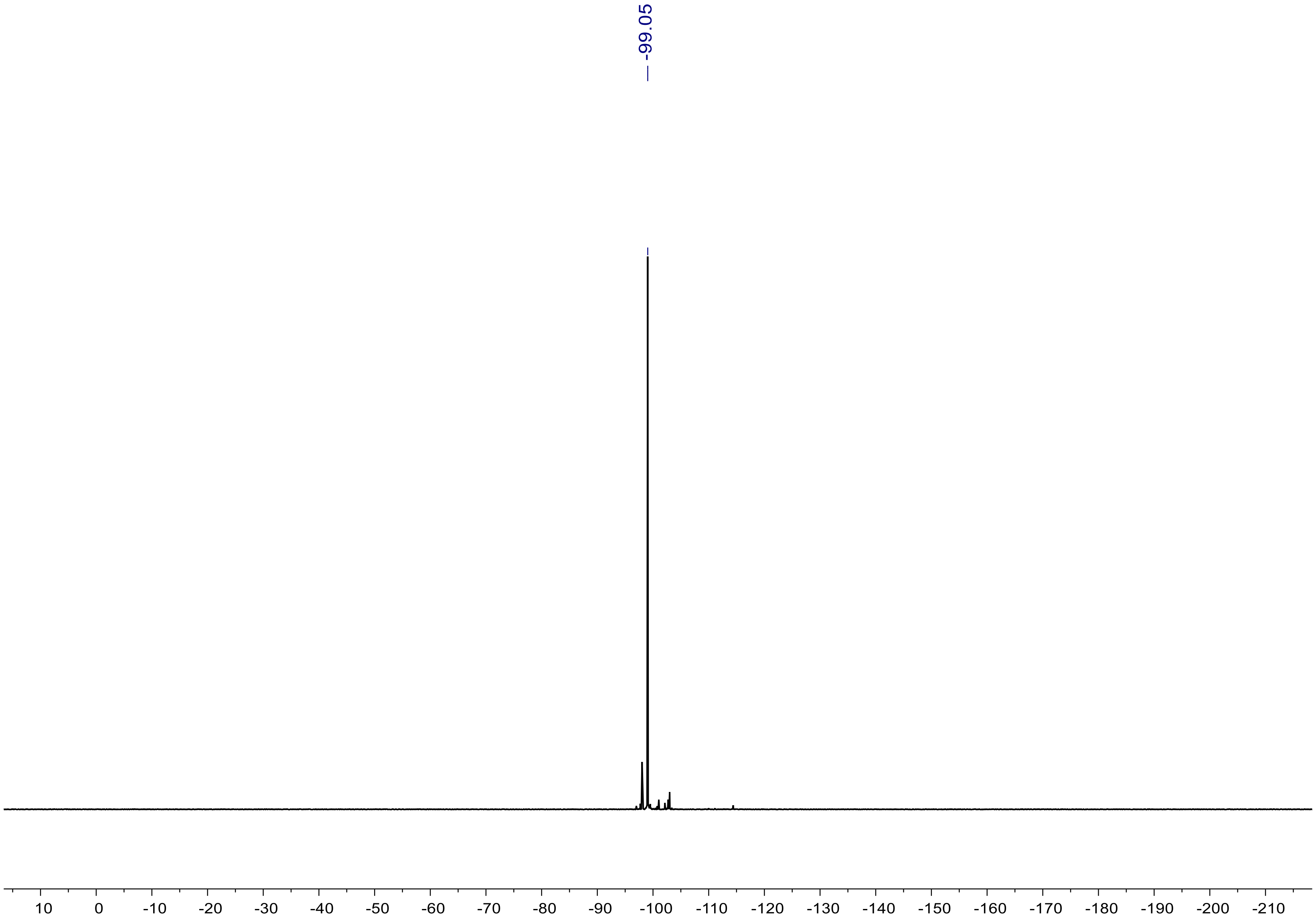

S4 ^{1}H NMR (500 MHz, $CDCl_3$, 298 K) spectrum of *tris(2,4,6-trifluorophenyl)borane.*

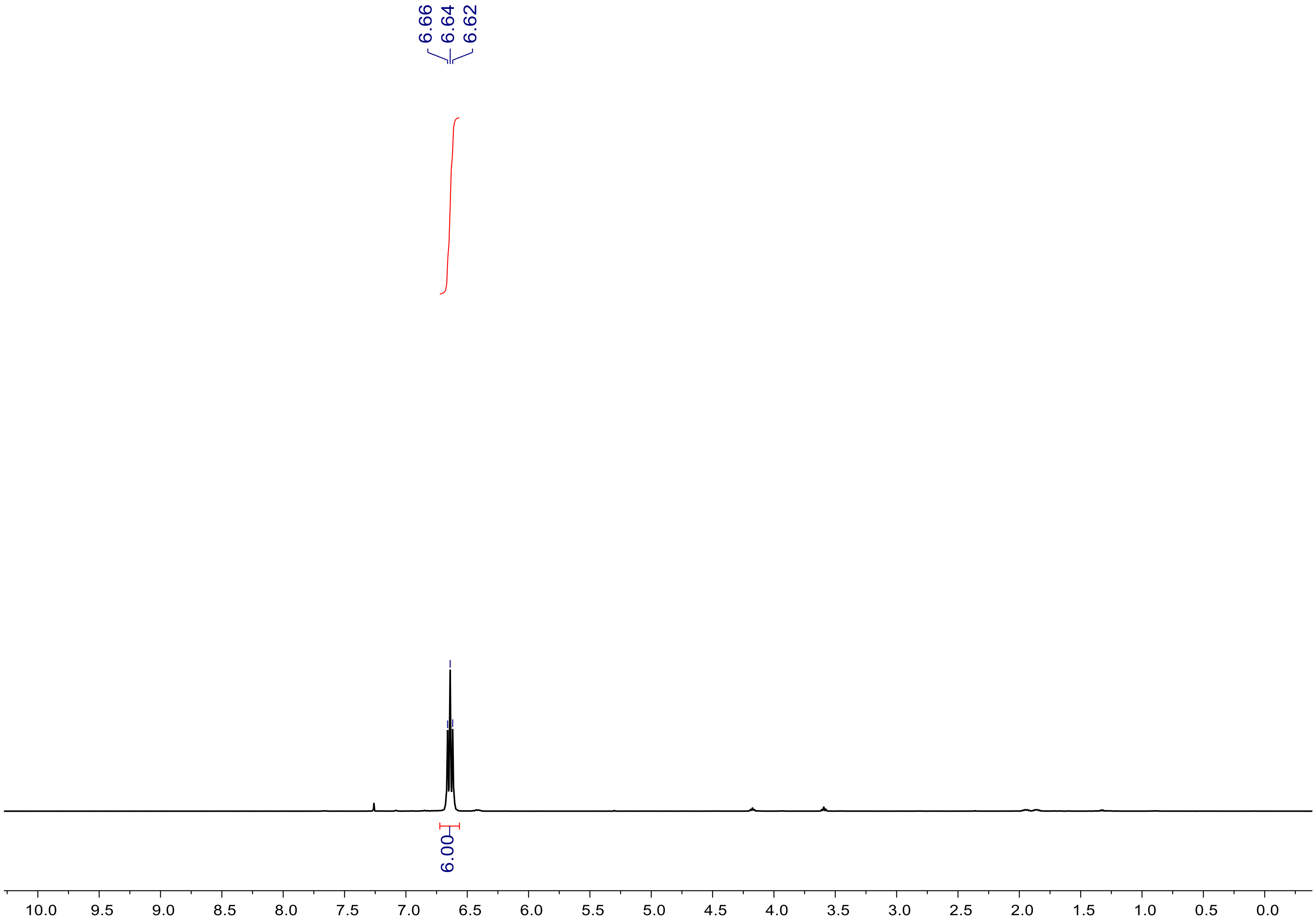

S5 ^{11}B NMR (160 MHz, $CDCl_3$, 298 K) spectrum of *tris(2,4,6-trifluorophenyl)borane*.

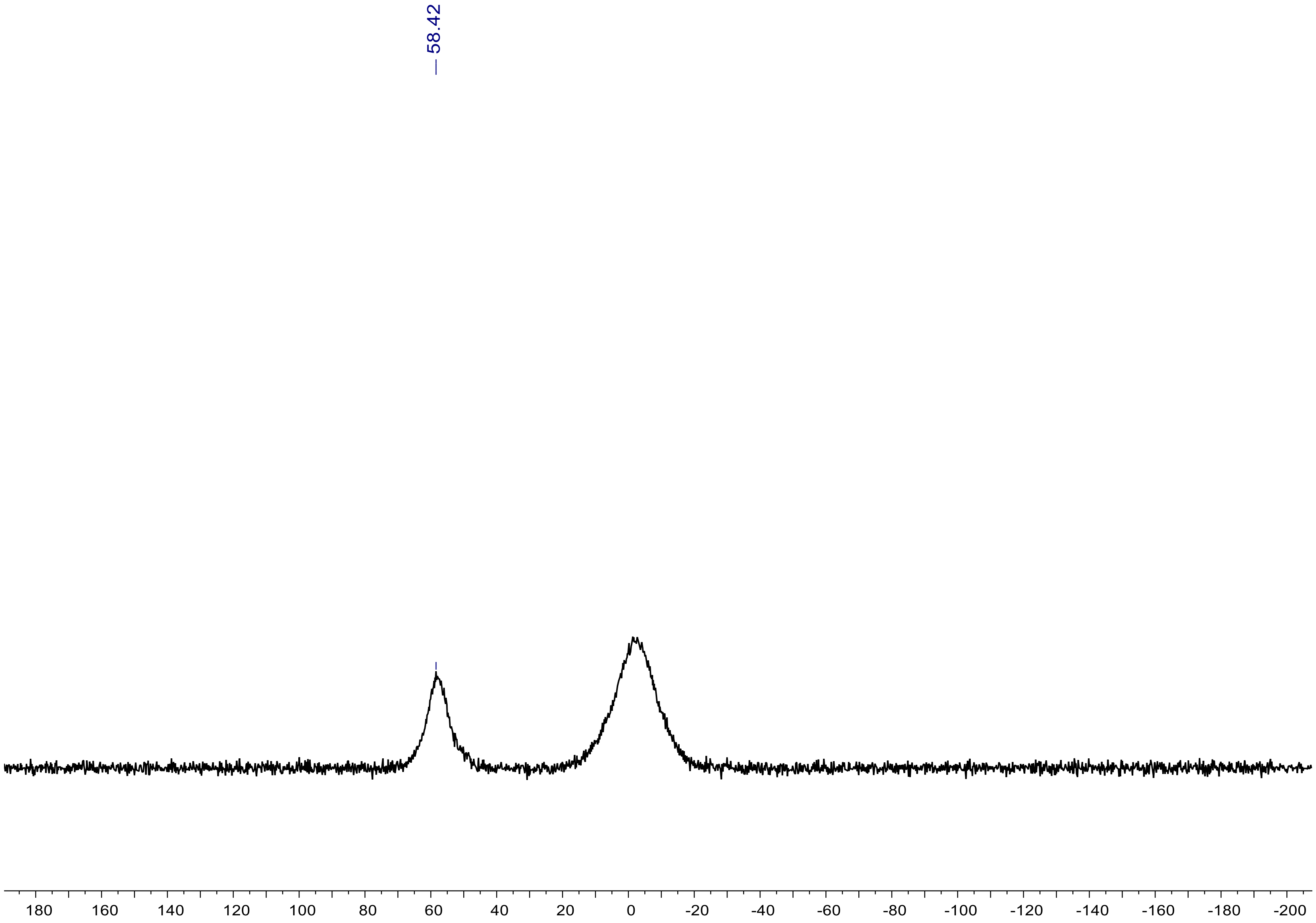

S6 ^{19}F NMR (471 MHz, $CDCl_3$, 298 K) spectrum of *tris(2,4,6-trifluorophenyl)borane*.

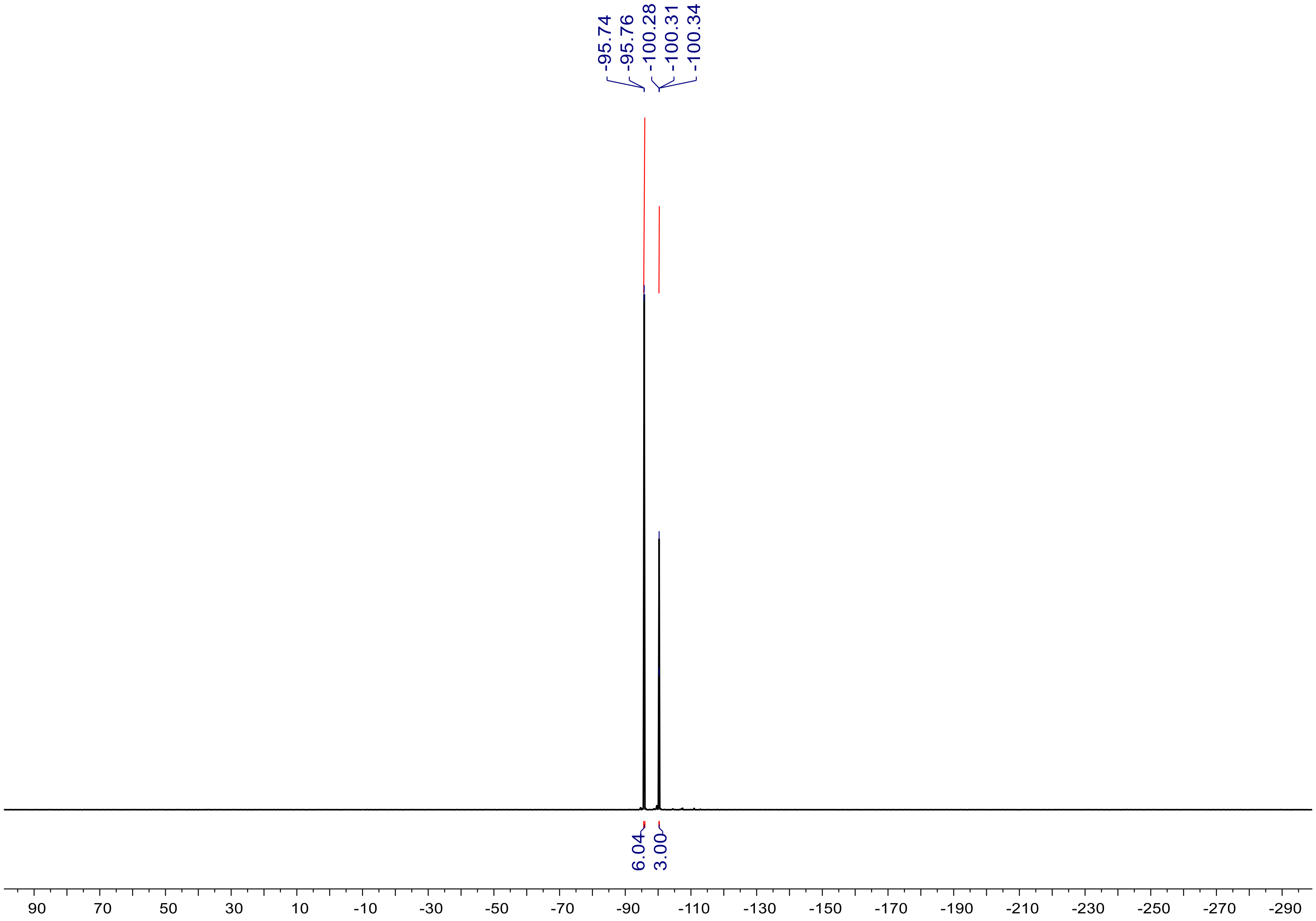

S7 ^{1}H NMR (500 MHz, $CDCl_3$, 298 K) spectrum of *prop-2-yn-1-yl benzoate*.

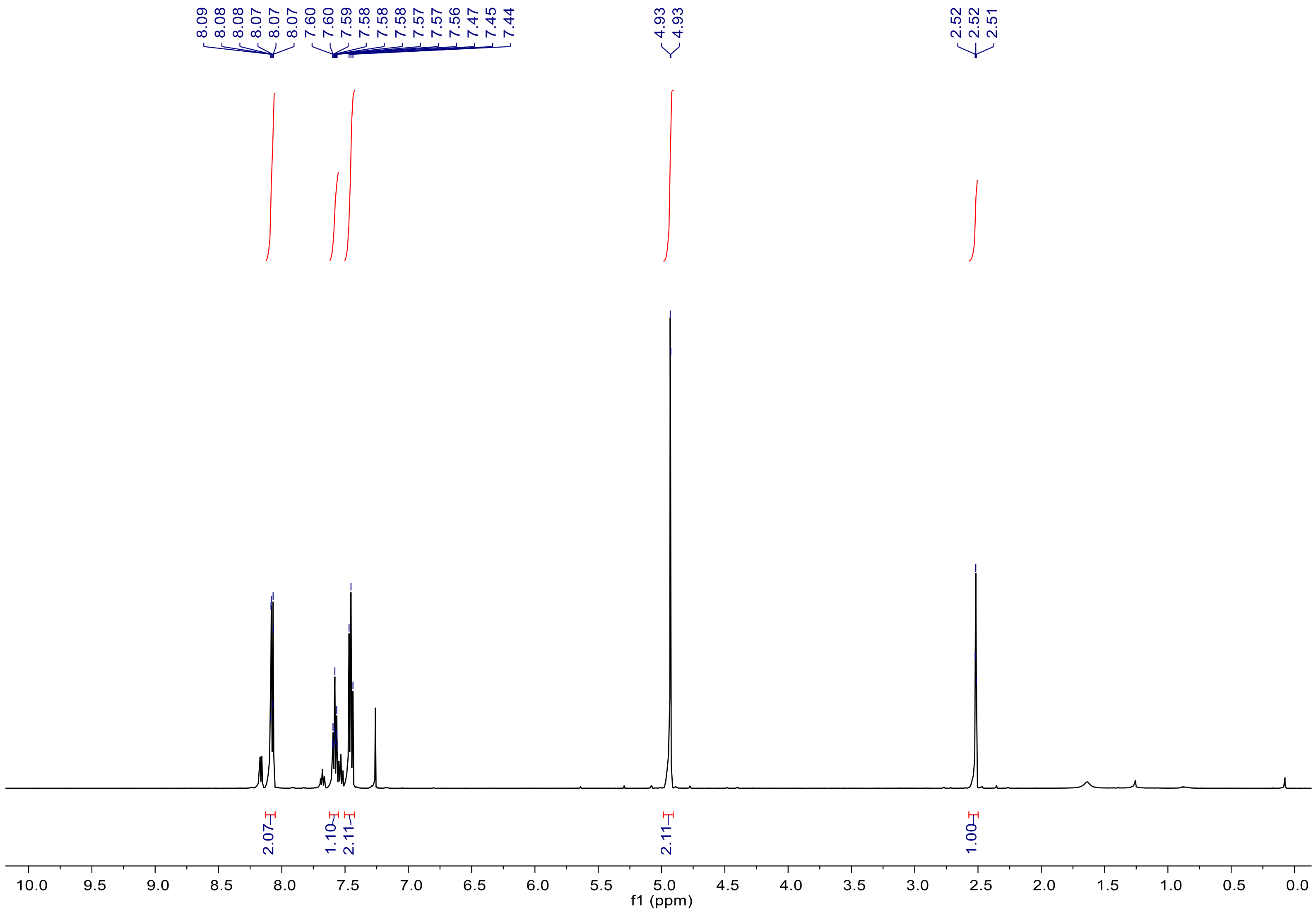

S8 ^{1}H NMR (500 MHz, $CDCl_3$, 298 K) spectrum of *prop-2-yn-1-yl-4-methylbenznoate*.

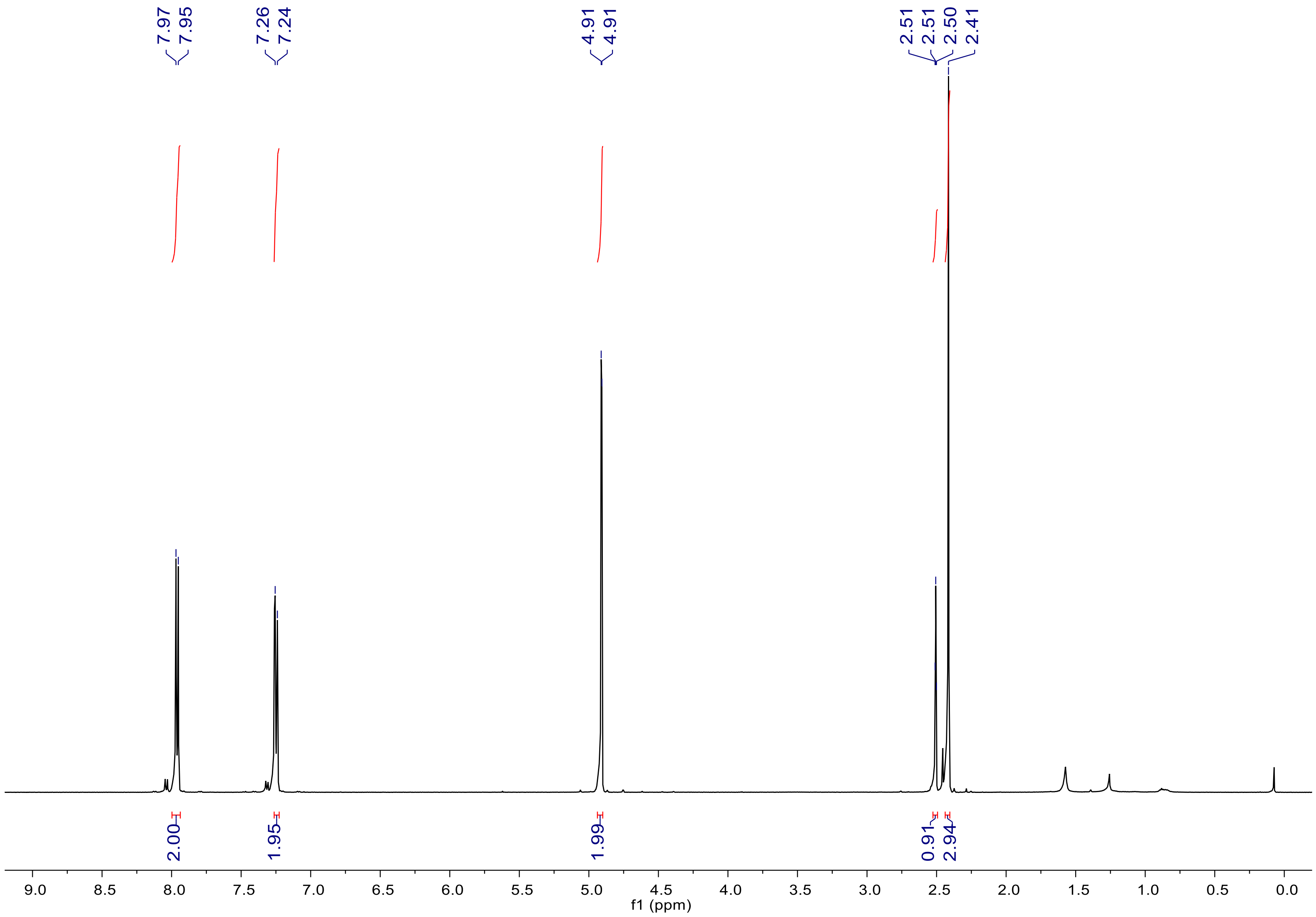

S9 ^{1}H NMR (500 MHz, $CDCl_3$, 298 K) spectrum of *prop-2-yn-1-yl-4-methoxybenznoate*

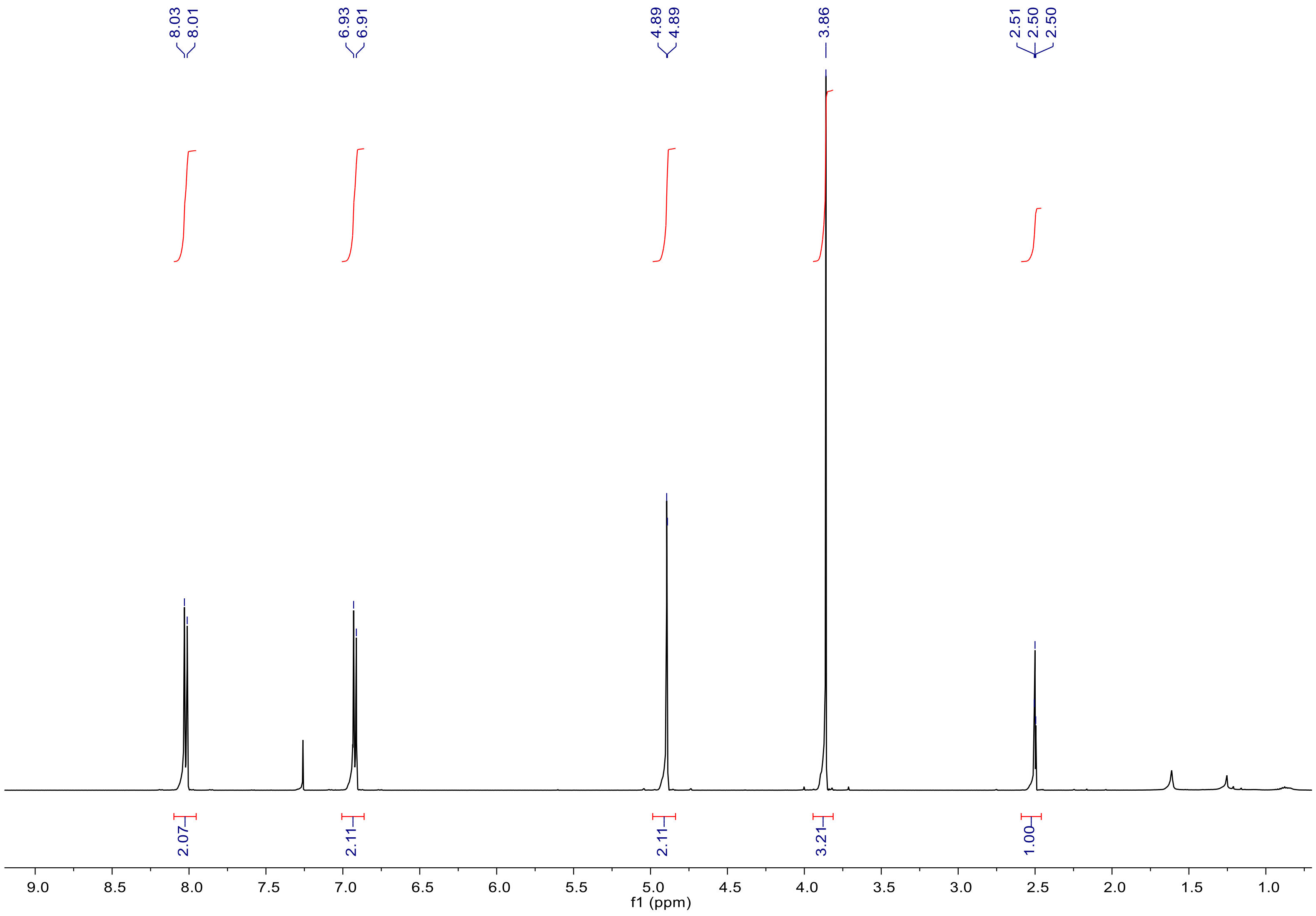

S10 ^{1}H NMR (500 MHz, $CDCl_3$, 298 K) spectrum of *prop-2-yn-1-yl-4-nitrobenznoate*.

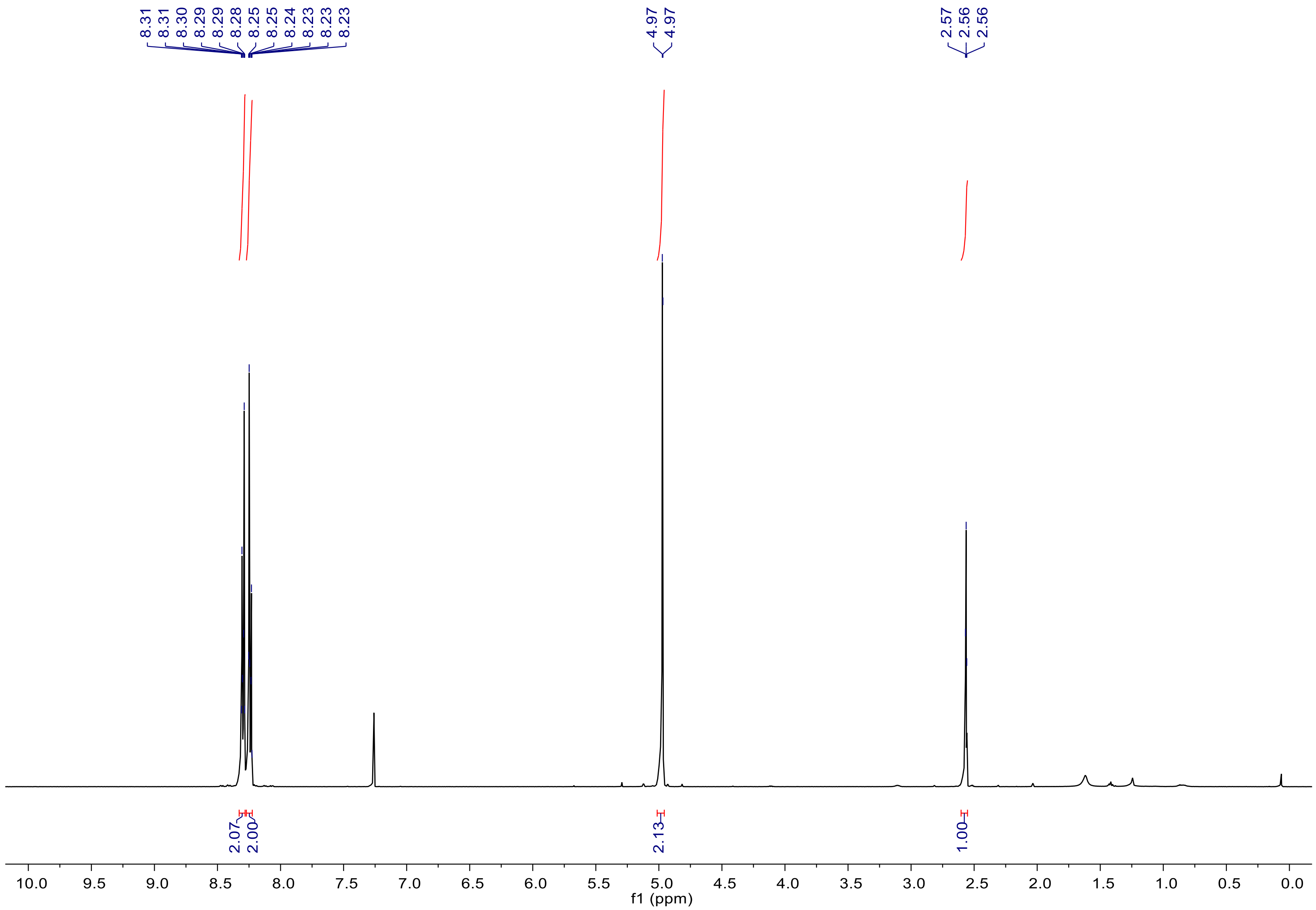

S11 ^{1}H NMR (500 MHz, $CDCl_3$, 298 K) spectrum of *(E)-N-phenyl-1-(p-tolyl)methanimine*.

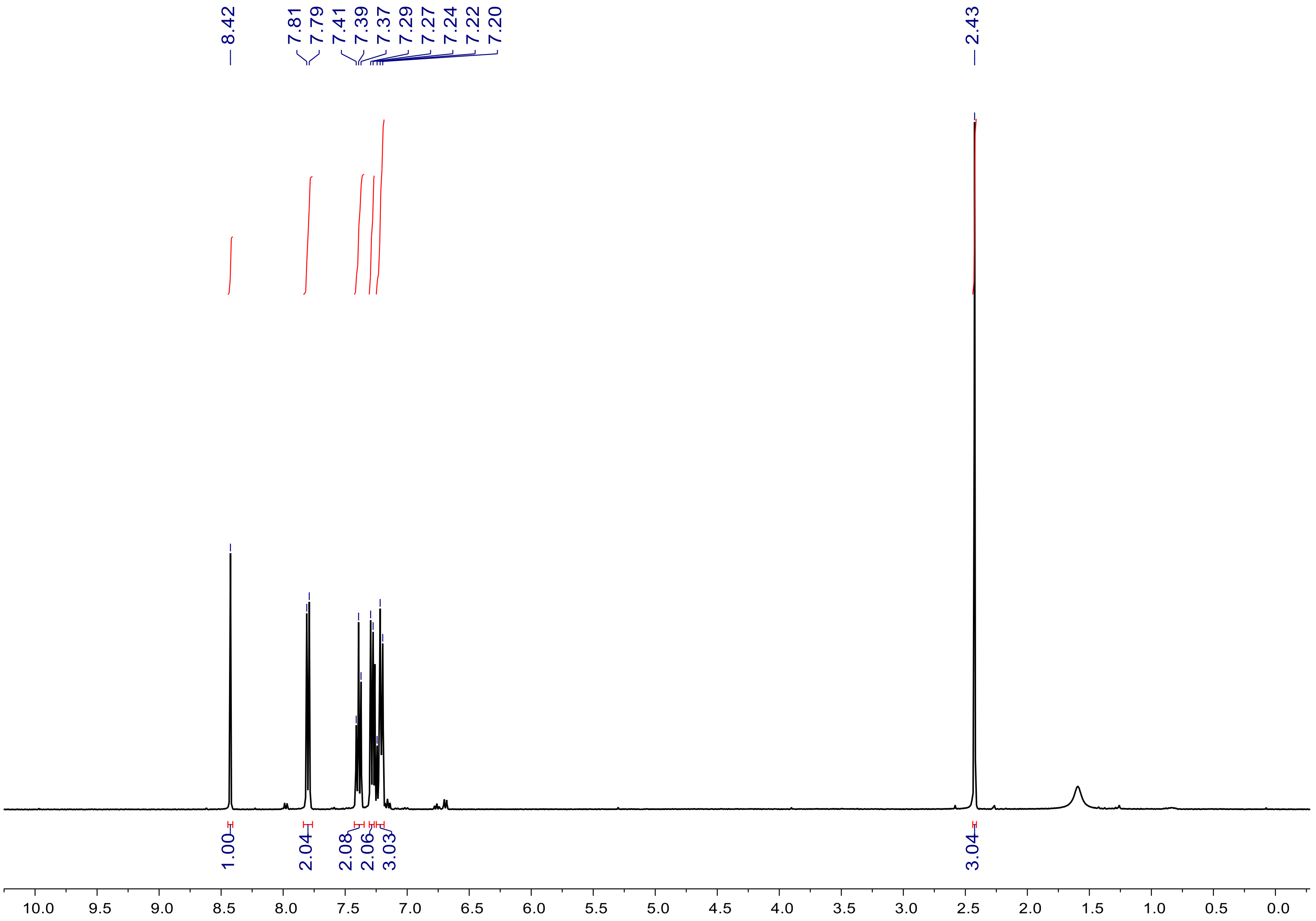

S12 ^{1}H NMR (500 MHz, $CDCl_3$, 298 K) spectrum of *(E)-1-mesityl-N-phenylmethanimine*.

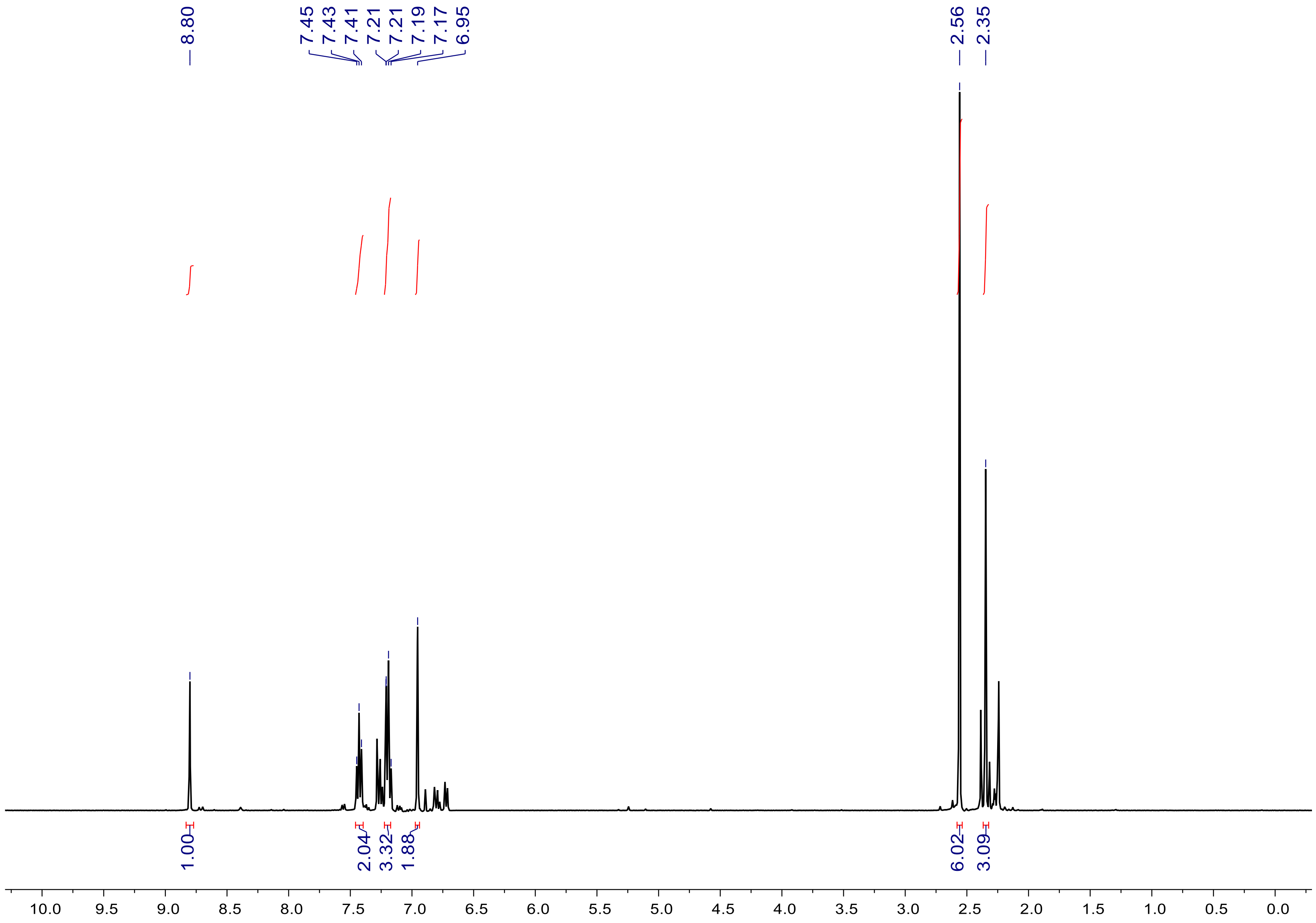

S13 ^{1}H NMR (500 MHz, $CDCl_3$, 298 K) spectrum of *(E)-1-(4-methoxyphenyl)-N-phenylmethanimine*.

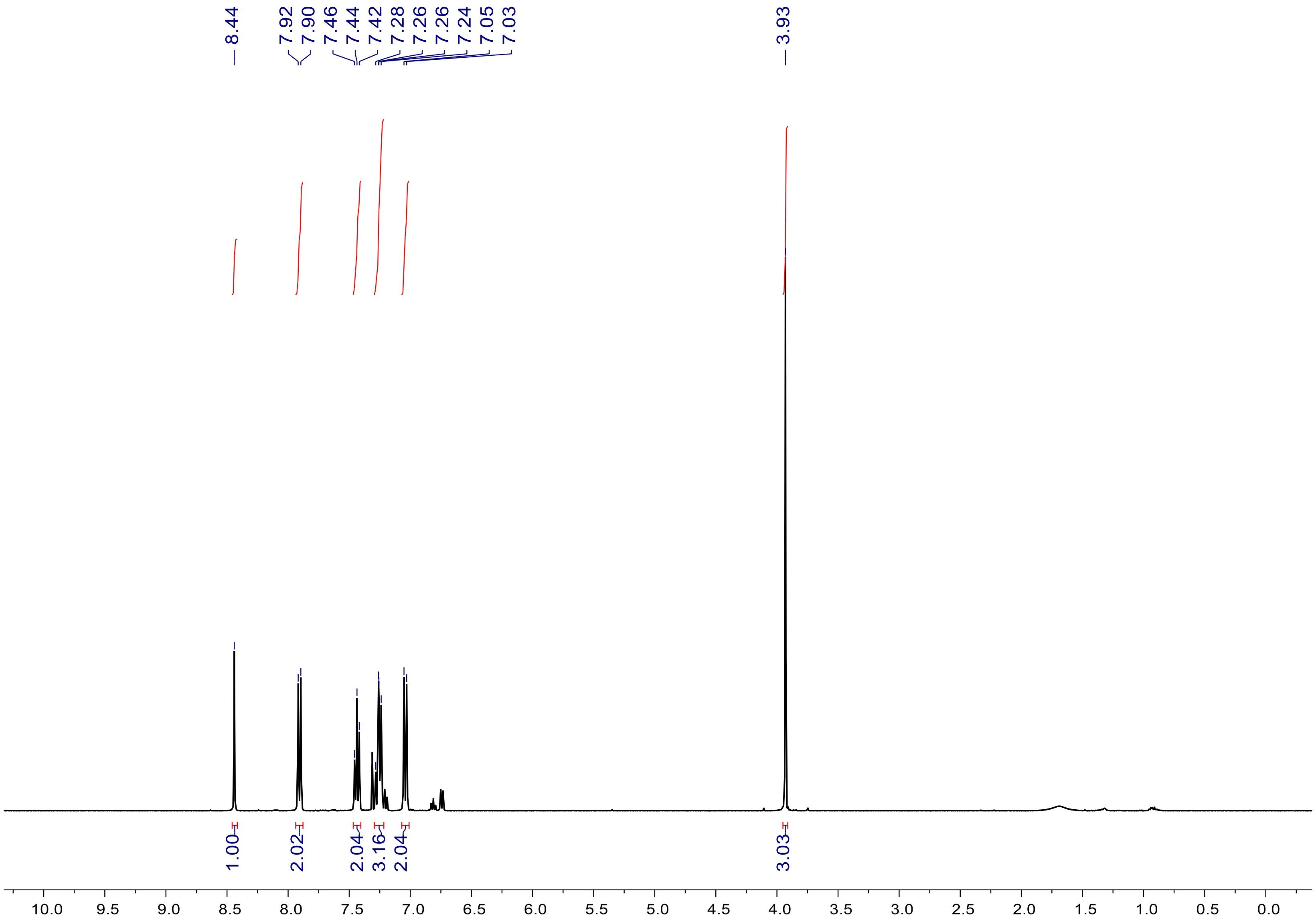

S14 ^{1}H NMR (500 MHz, $CDCl_3$, 298 K) spectrum of *(E)-1-(2-methoxyphenyl)-N-phenylmethanimine*.

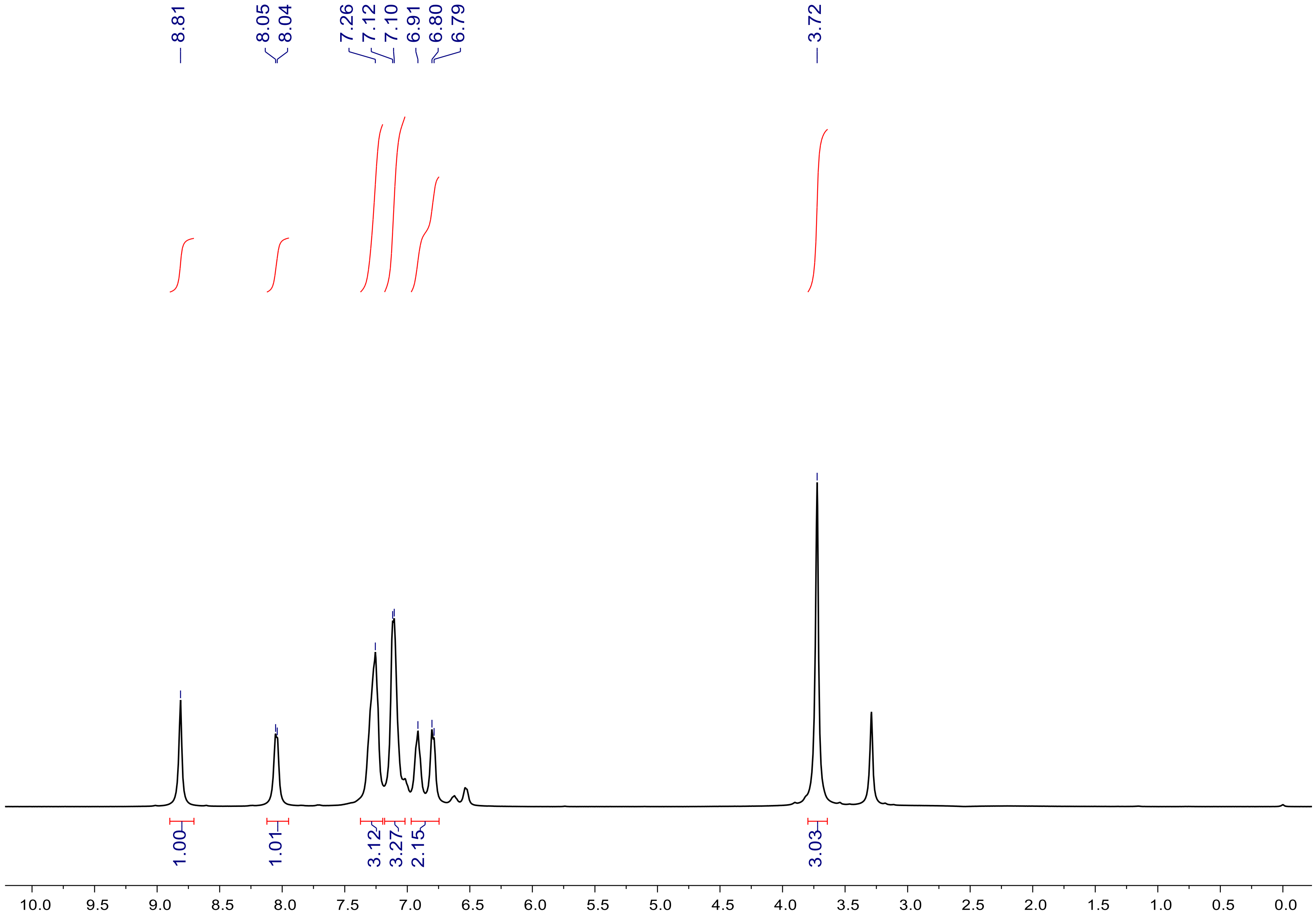

S15 ^{1}H NMR (500 MHz, $CDCl_3$, 298 K) spectrum of *(E)-1-(4-fluorophenyl)-N-phenylmethanimine.*

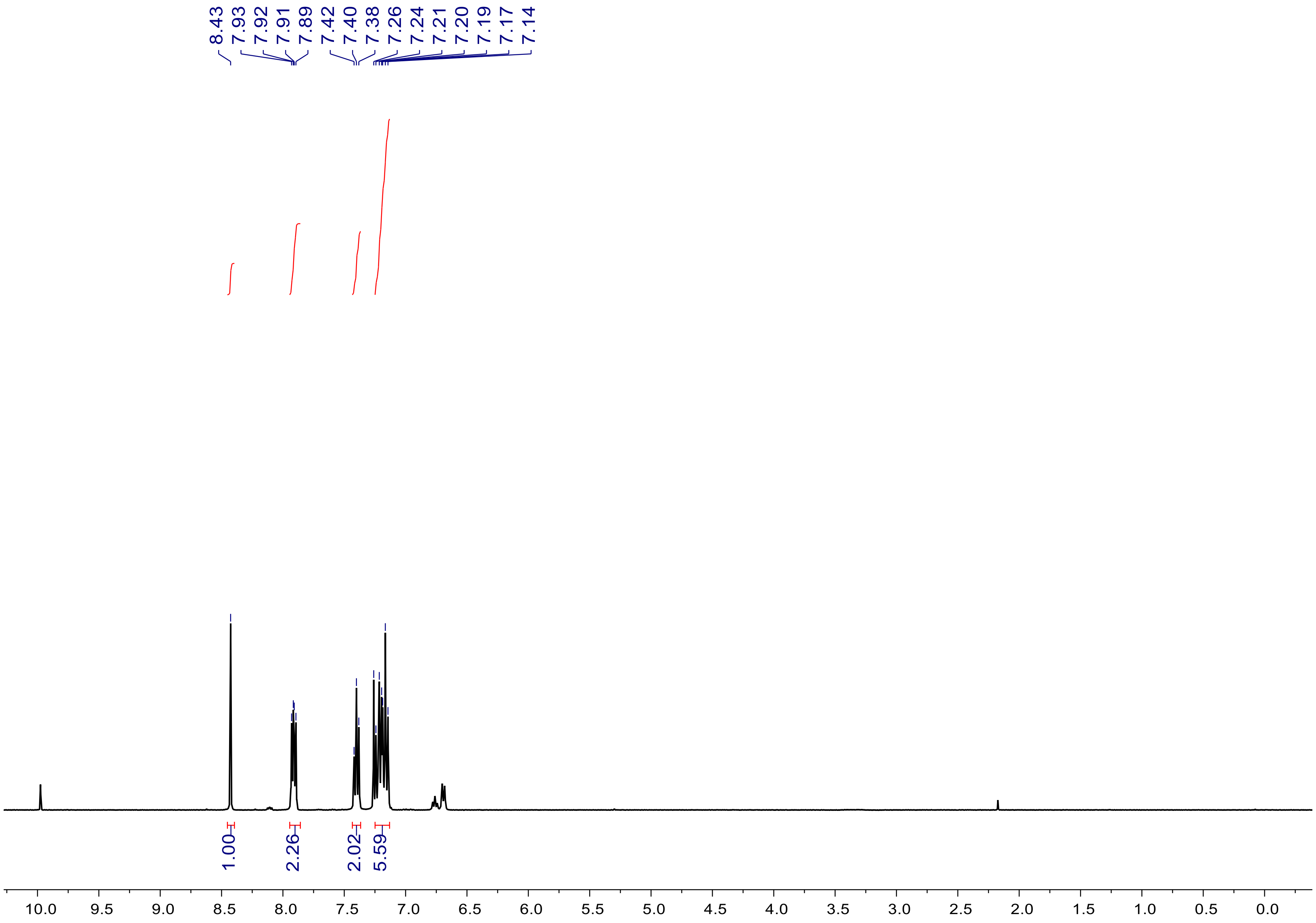

S16 ^{1}H NMR (500 MHz, $CDCl_3$, 298 K) spectrum of *(E)-1-(4-nitrophenyl)-N-phenylmethanimine*.

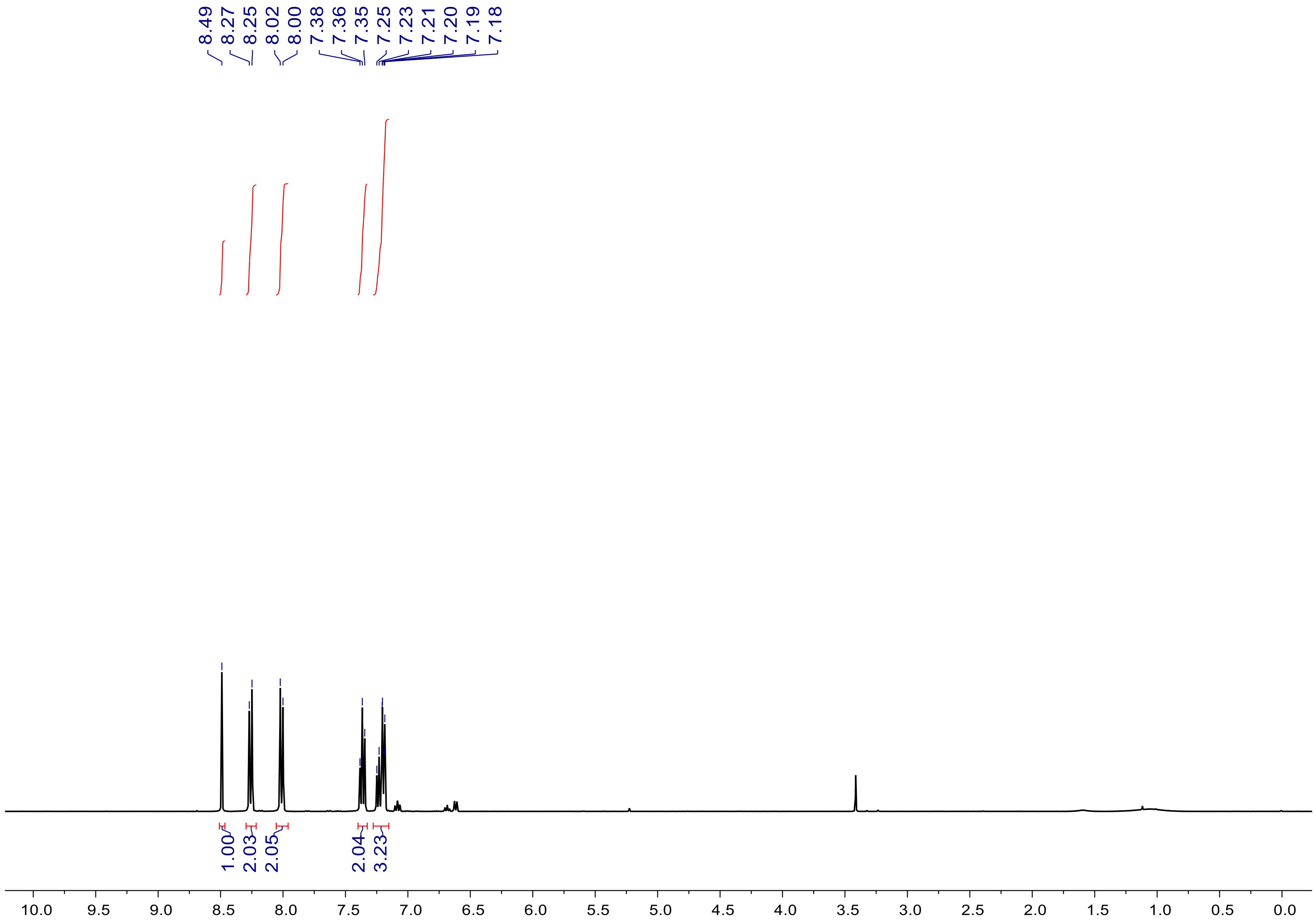

S17 ^{1}H NMR (500 MHz, $CDCl_3$, 298 K) spectrum of *(E)-1-(naphthalen-2-yl)-N-phenylmethanimine*.

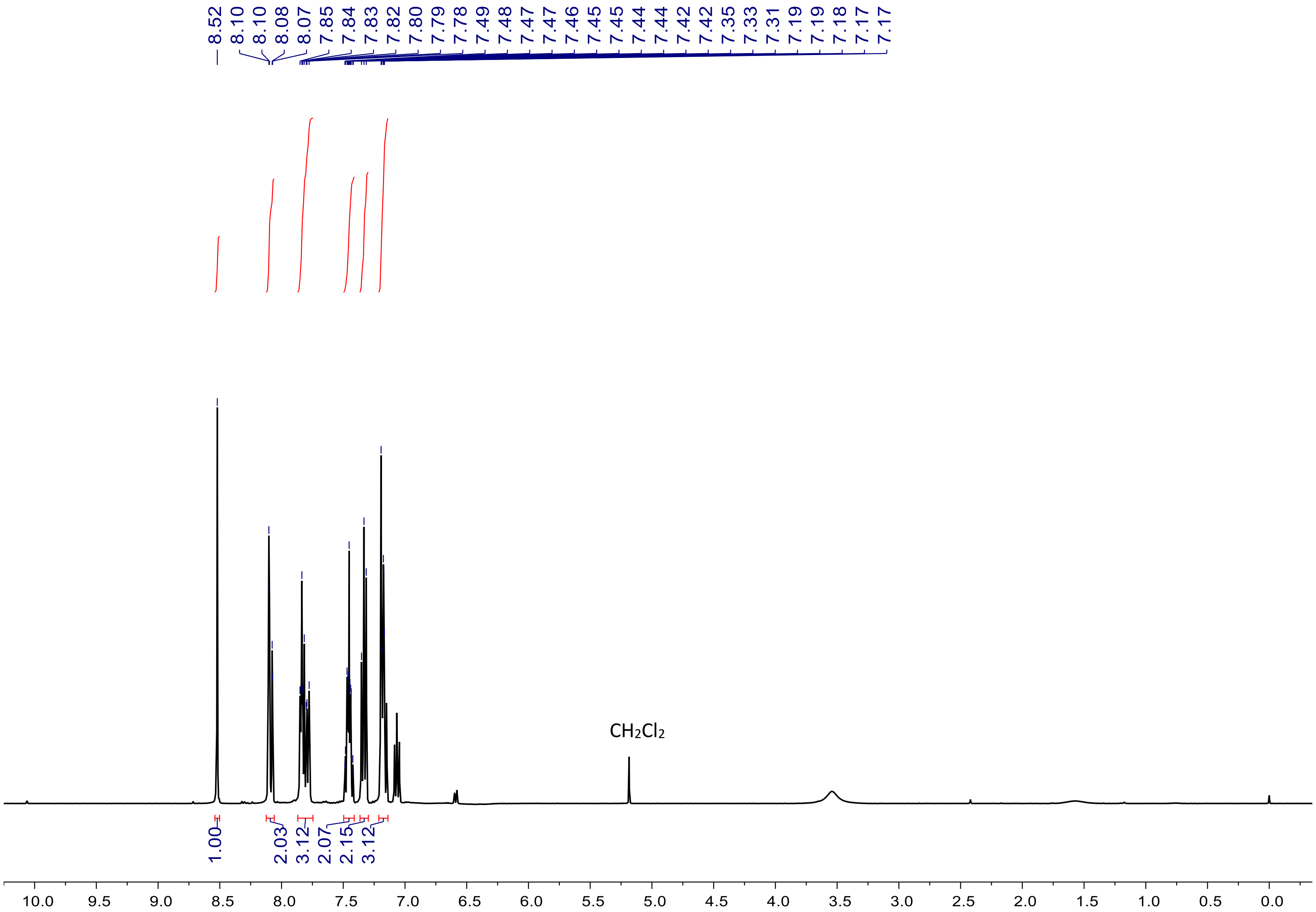

S18 ^{1}H NMR (500 MHz, $CDCl_3$, 298 K) spectrum of *(E)-N-butyl-1-phenylmethanimine*.

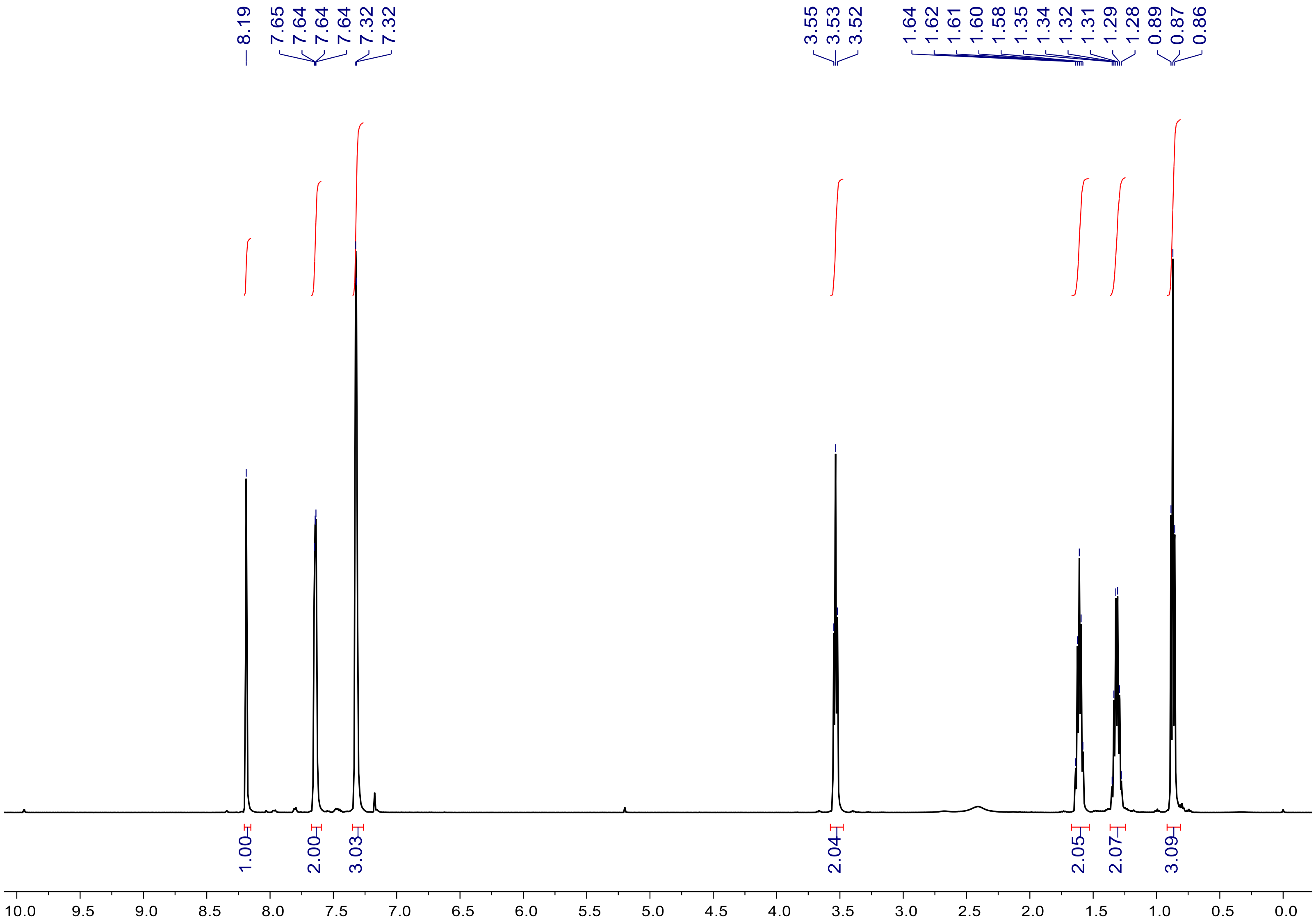

S19 ^{1}H NMR (500 MHz, $CDCl_3$, 298 K) spectrum of *(E)-N-isopropyl-1-phenylmethanimine*.

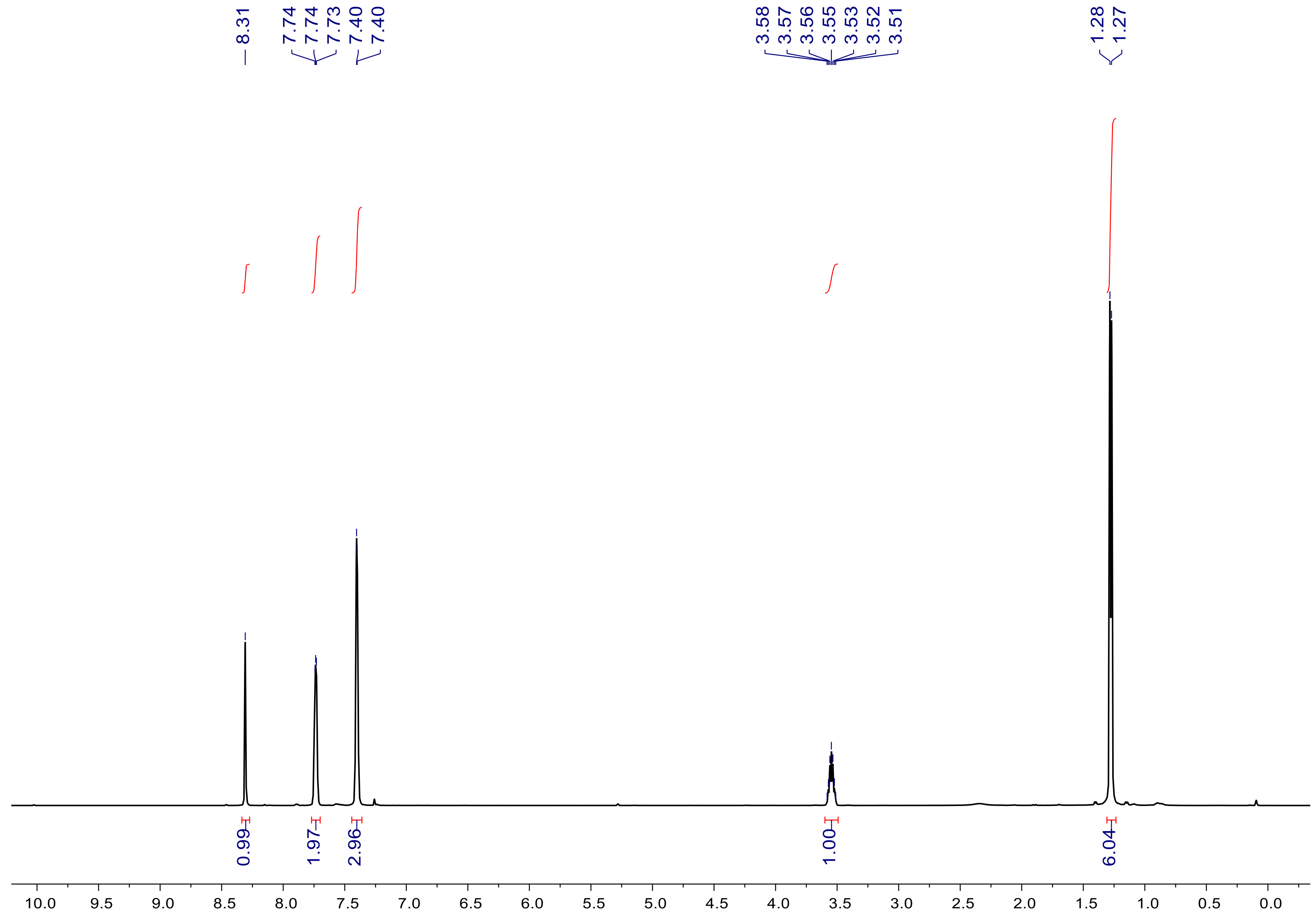

S20 ^{1}H NMR (500 MHz, $CDCl_3$, 298 K) spectrum of *(E)-N-cyclopentyl-1-phenylmethanimine*.

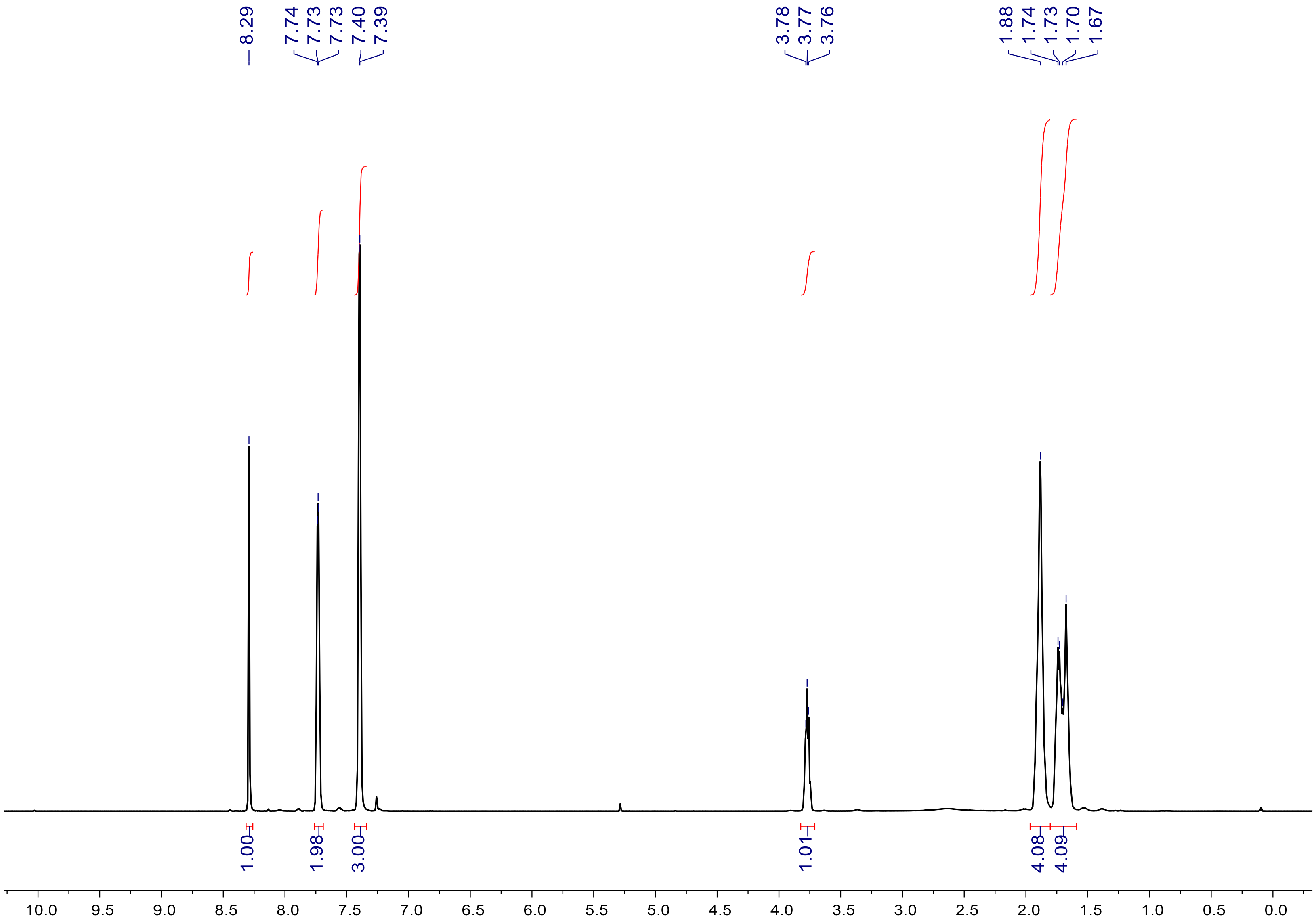

S21 ^{1}H NMR (500 MHz, $CDCl_3$, 298 K) spectrum of *(E)-N-benzyl-1-phenylmethanimine*.

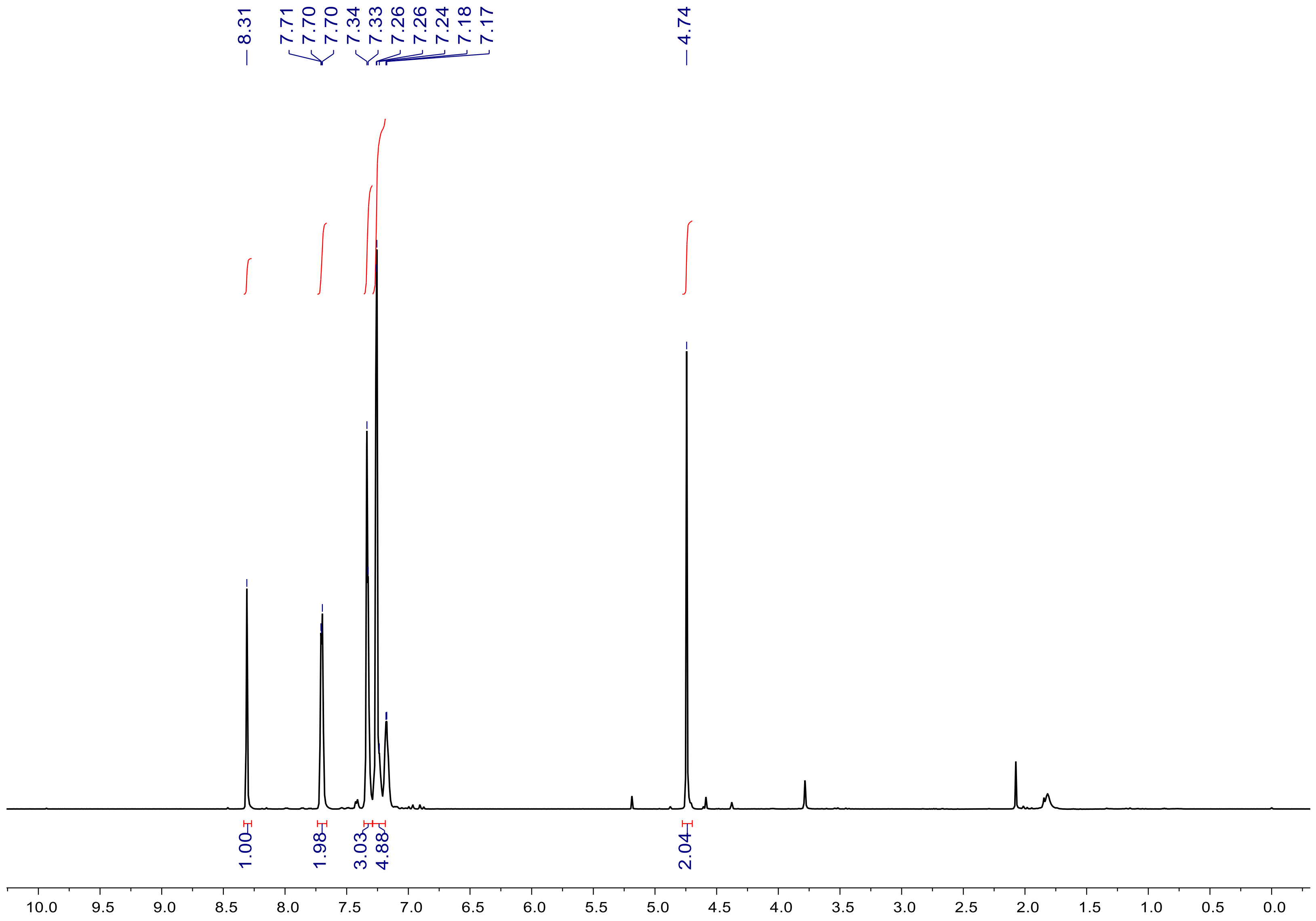

S22 ^{1}H NMR (500 MHz, $CDCl_3$, 298 K) spectrum of *(E)-N-(2,6-diethylphenyl)-1-phenylmethanimine*.

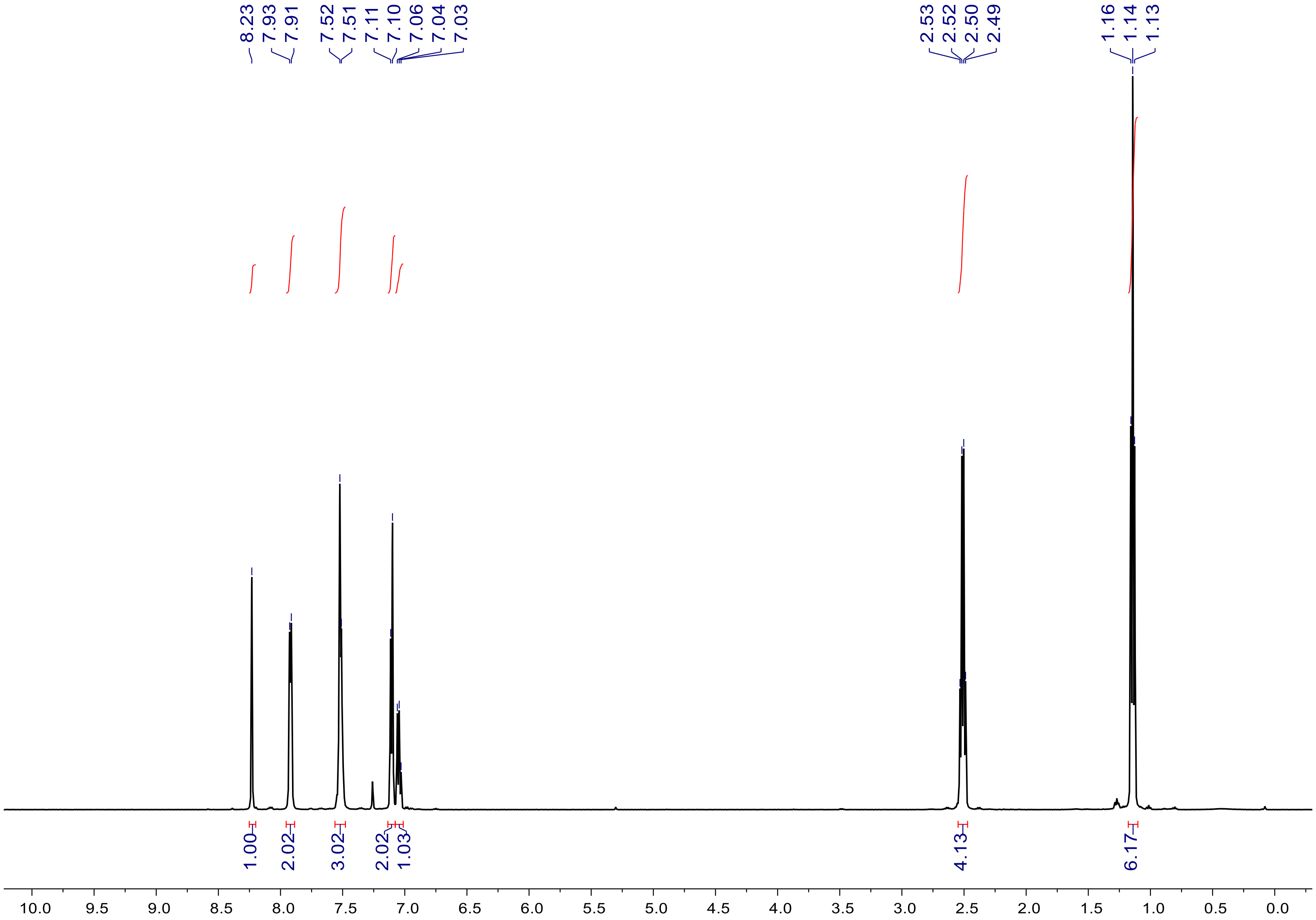

S23 ^{1}H NMR (500 MHz, $CDCl_3$, 298 K) spectrum of *(E)-N-mesityl-1-phenylmethanimine*.

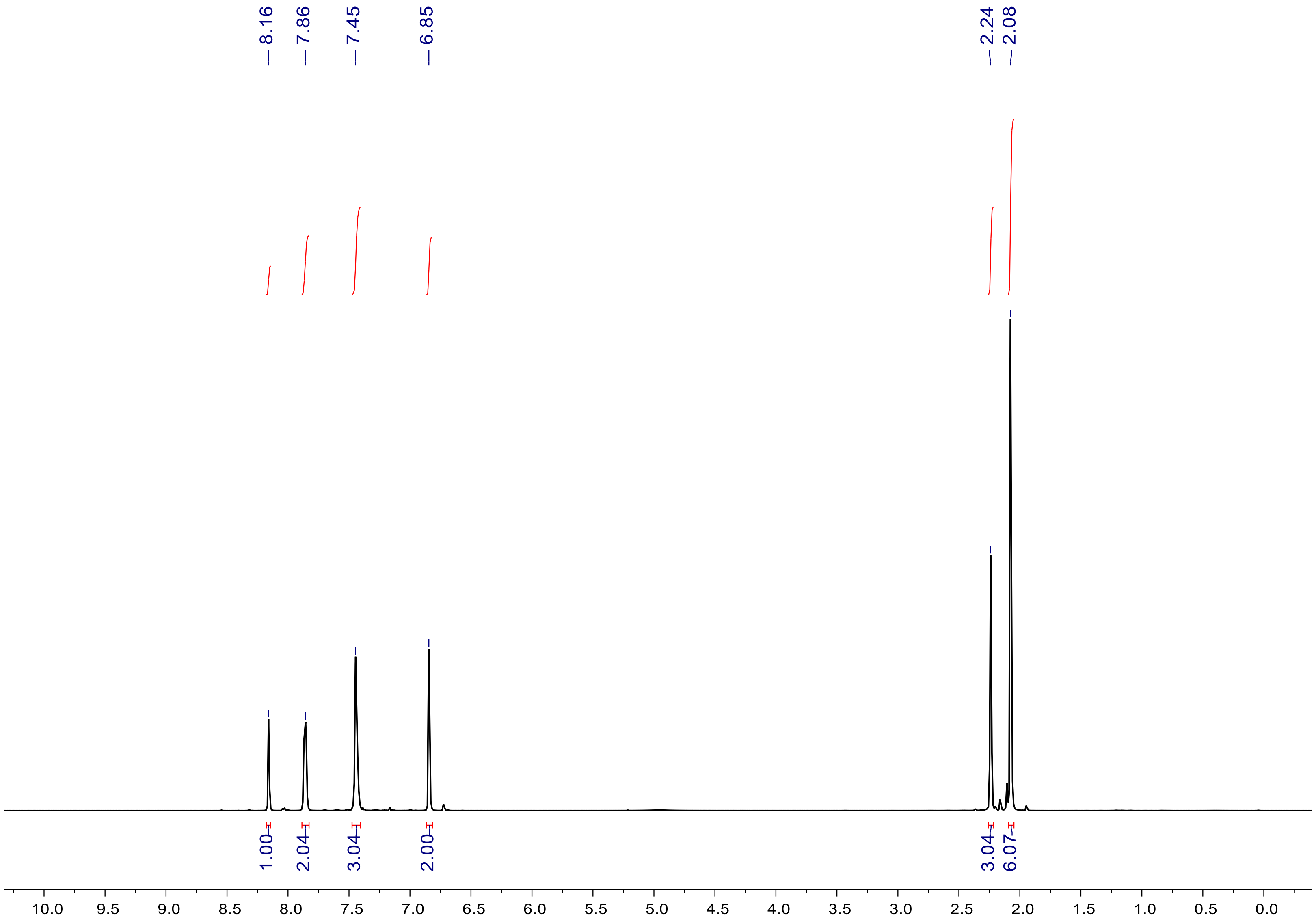

S24 ^{1}H NMR (500 MHz, $CDCl_3$, 298 K) spectrum of *(E)-1-phenyl-N-(4-(trifluoromethyl)phenyl)methanimine.*

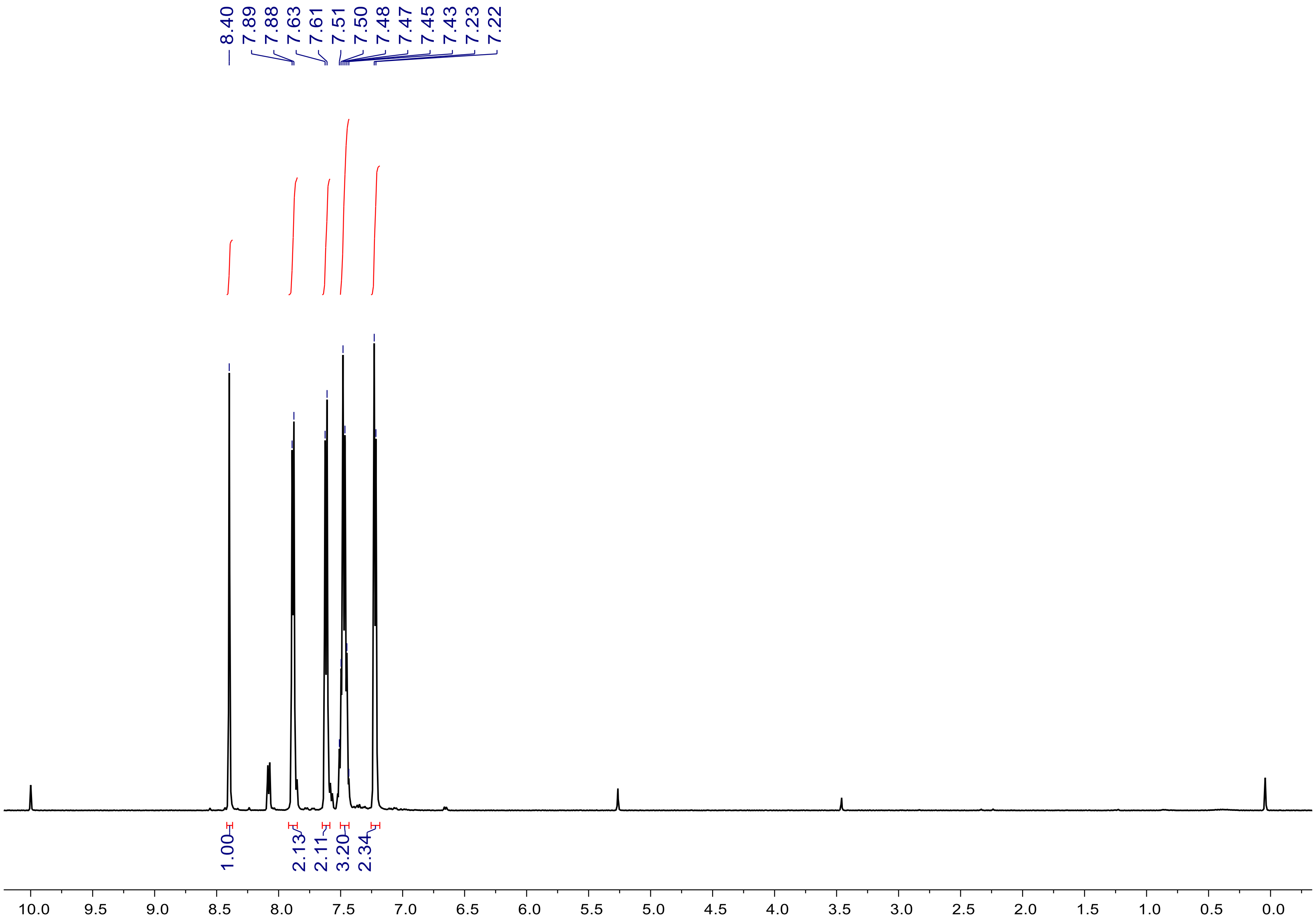

S25 ^{1}H NMR (500 MHz, $CDCl_3$, 298 K) spectrum of *(E)-N-(2-fluorophenyl)-1-phenylmethanimine*.

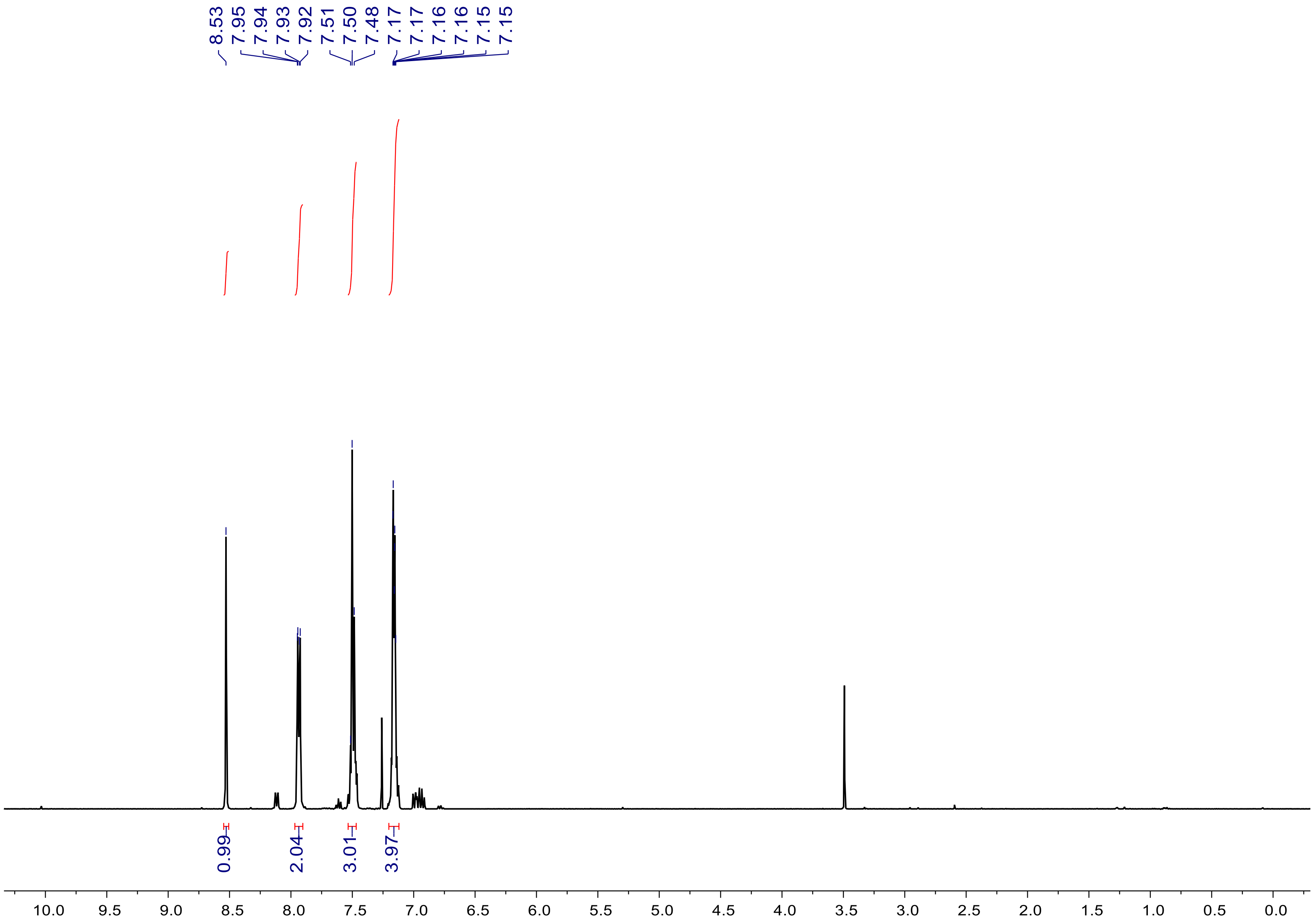

2.2 NMR spectra of products

S26 ^{1}H NMR (500 MHz, $CDCl_3$, 298 K) spectrum of *(E)-4,4,5,5-tetramethyl-2-styryl-1,3,2-dioxaborolane* **1a**.

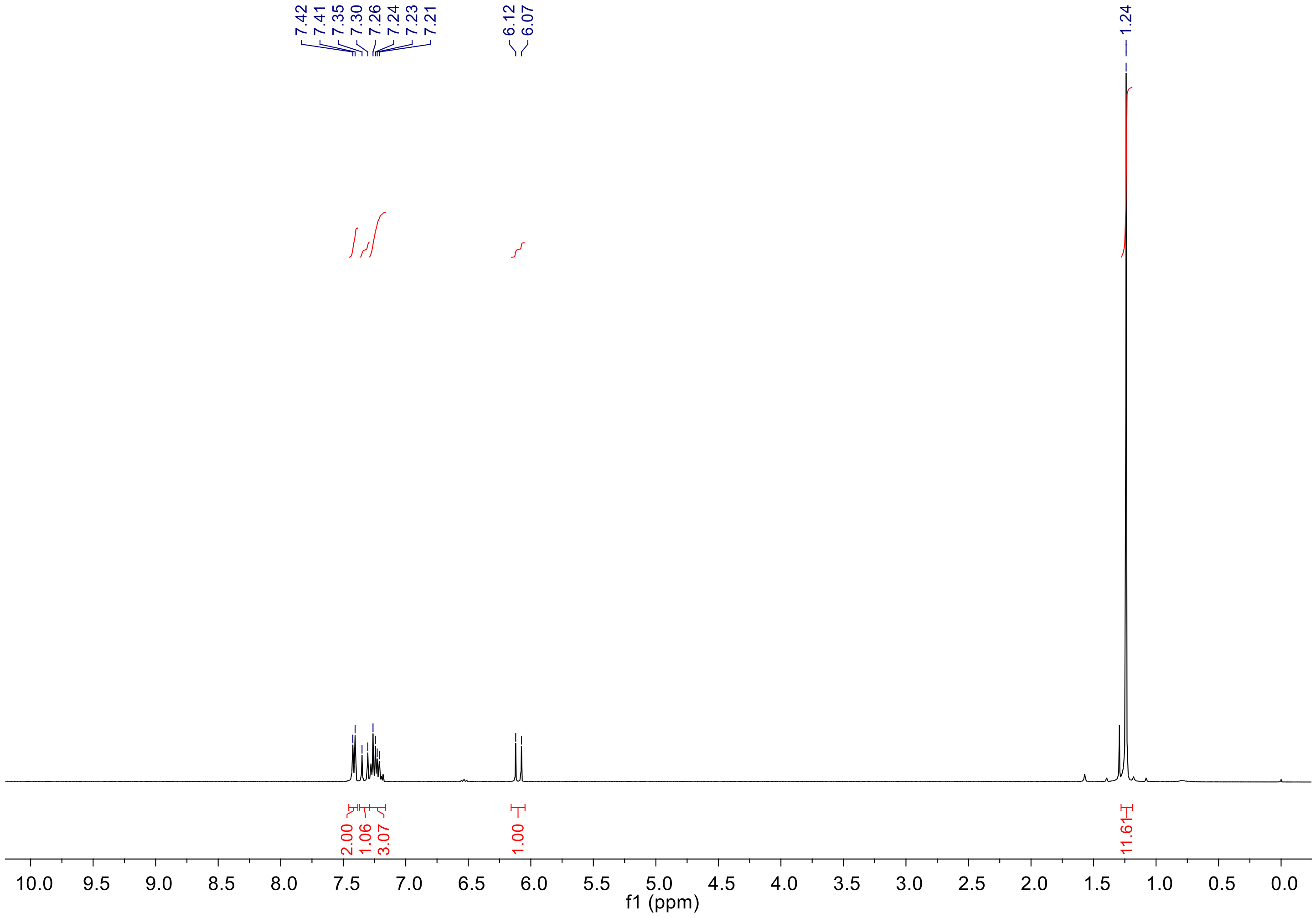

S27 ^{11}B NMR (160 MHz, $CDCl_3$, 298 K) spectrum of *(E)-4,4,5,5-tetramethyl-2-styryl-1,3,2-dioxaborolane* **1a**.

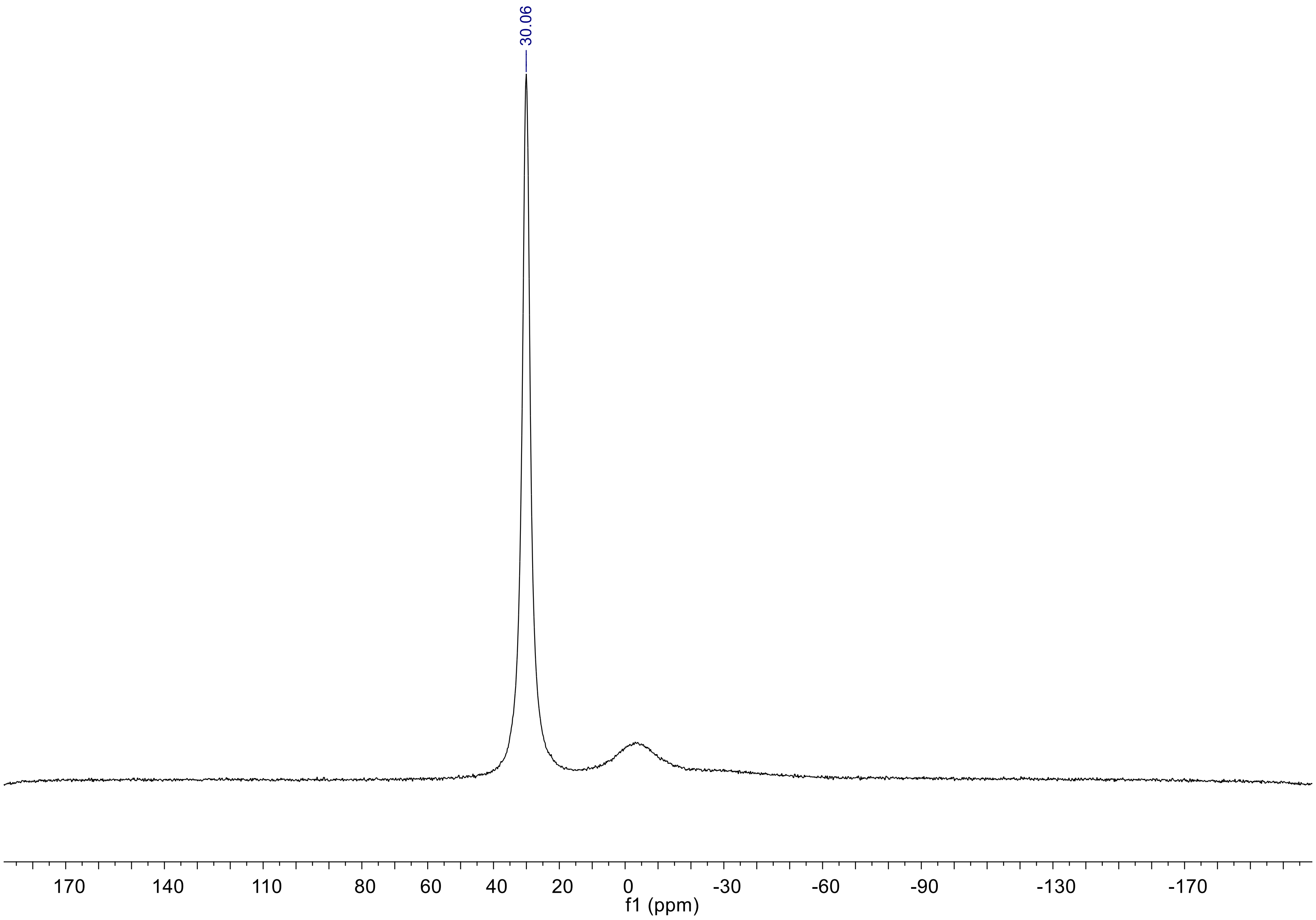

S28 ^{13}C NMR (126 MHz, $CDCl_3$, 298 K) spectrum of *(E)-4,4,5,5-tetramethyl-2-styryl-1,3,2-dioxaborolane* **1a**.

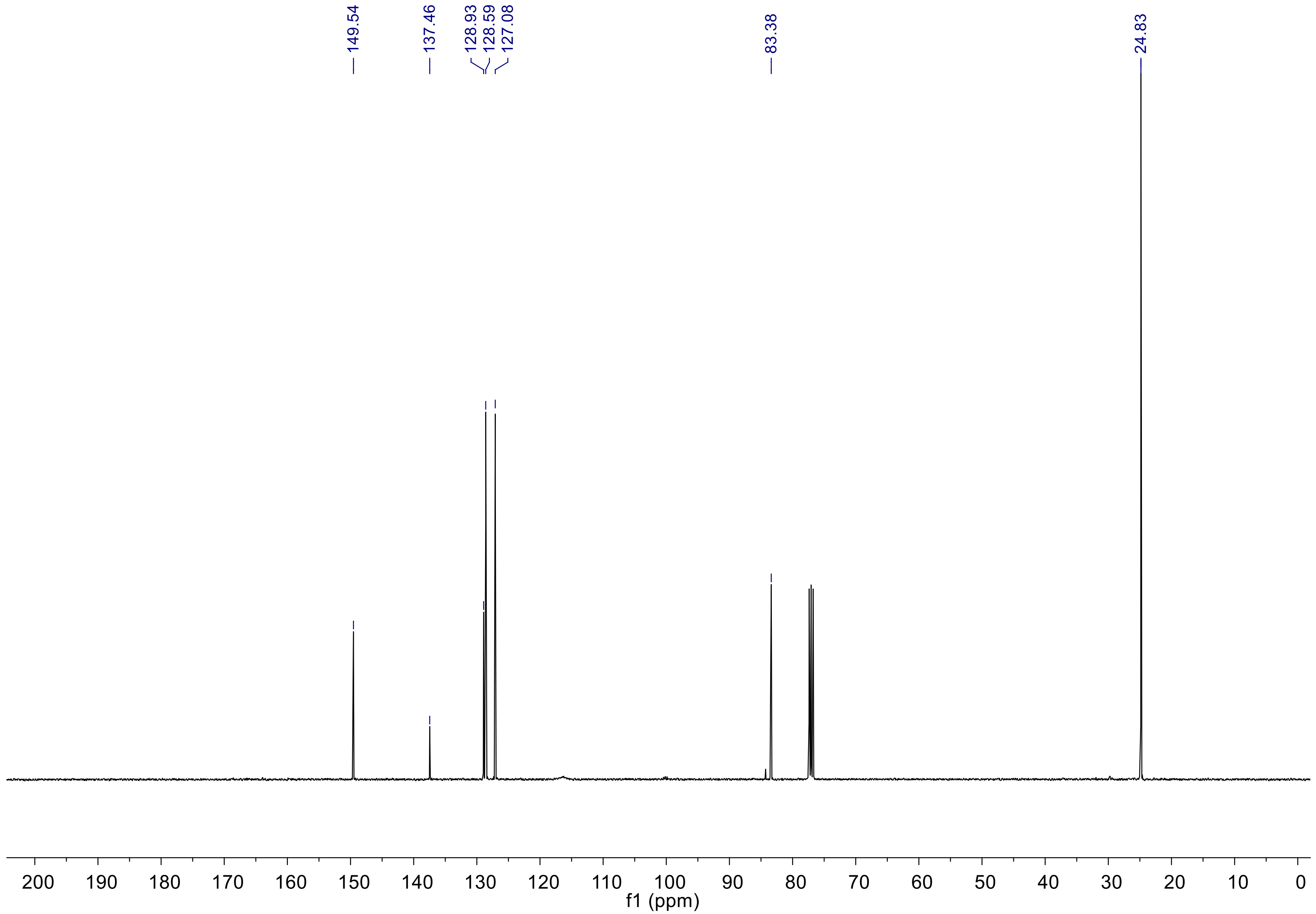

S29 ^{1}H NMR (500 MHz, $CDCl_3$, 298 K) spectrum of *(E)-2-(hex-1-en-1-yl)-4,4,5,5-tetramethyl-1,3,2-dioxaborolane* **1b**.

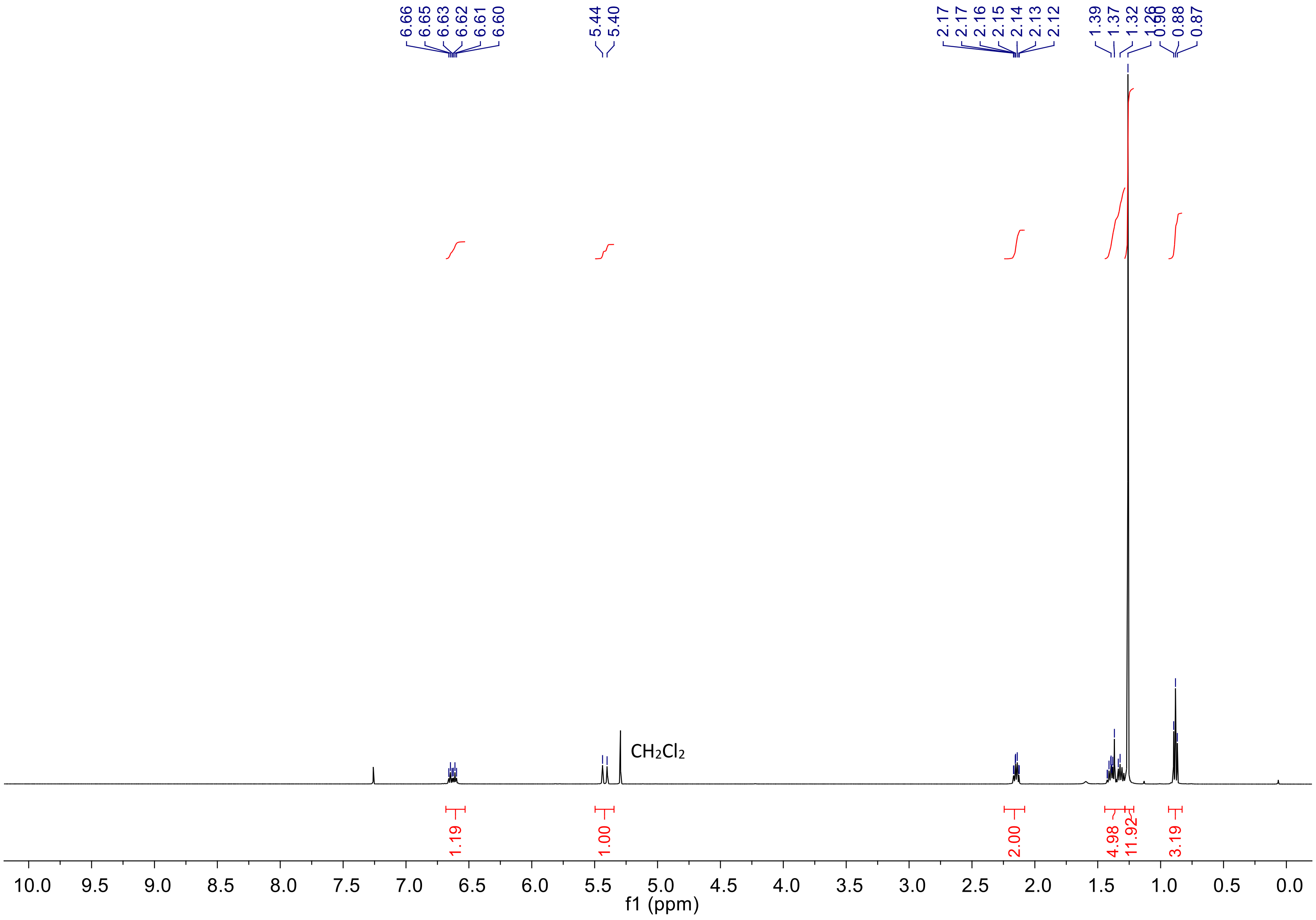

S30 ^{11}B NMR (160 MHz, $CDCl_3$, 298 K) spectrum of *(E)-2-(hex-1-en-1-yl)-4,4,5,5-tetramethyl-1,3,2-dioxaborolane* **1b**.

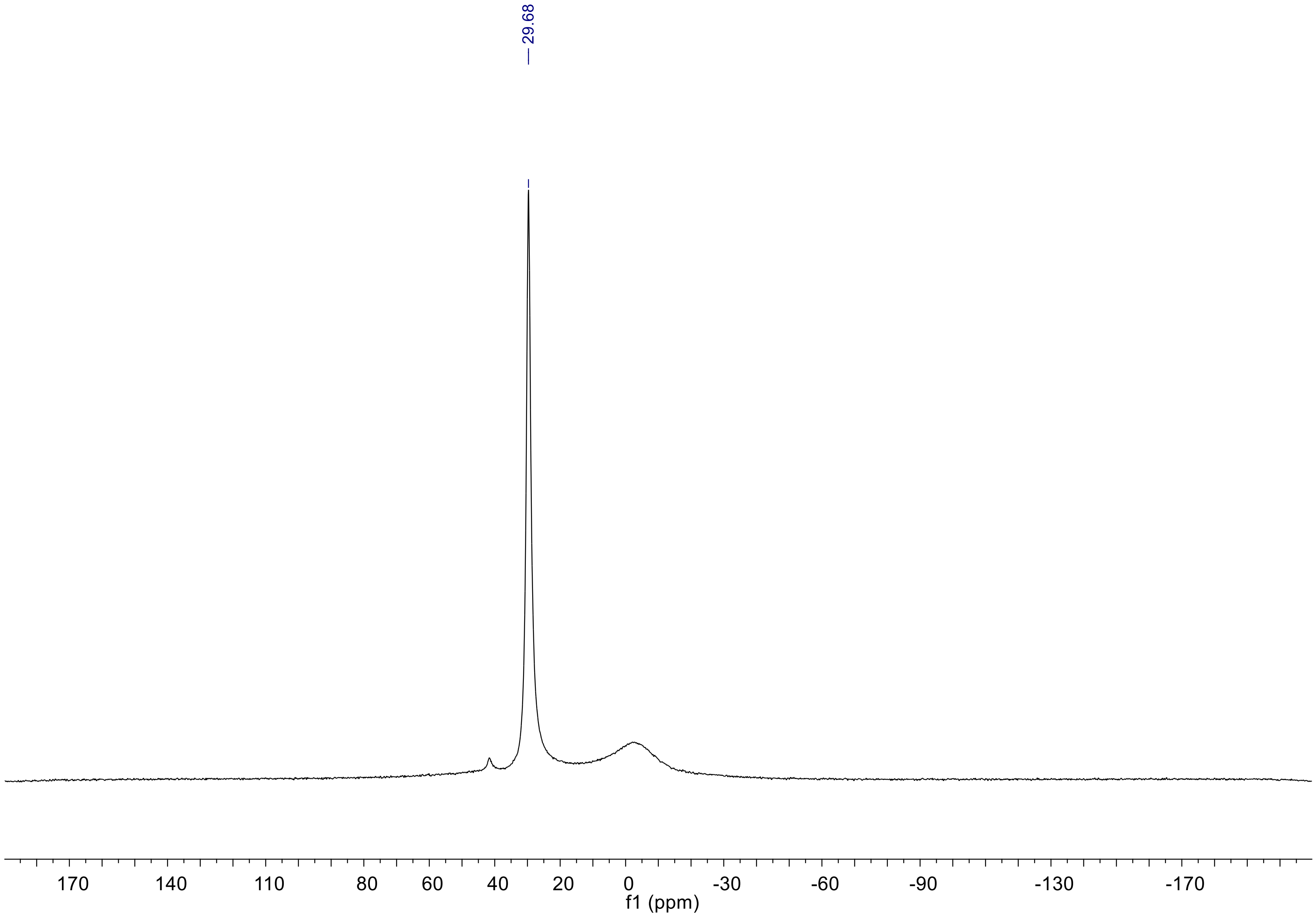

S31 ^{13}C NMR (126 MHz, $CDCl_3$, 298 K) spectrum of *(E)-2-(hex-1-en-1-yl)-4,4,5,5-tetramethyl-1,3,2-dioxaborolane* **1b**.

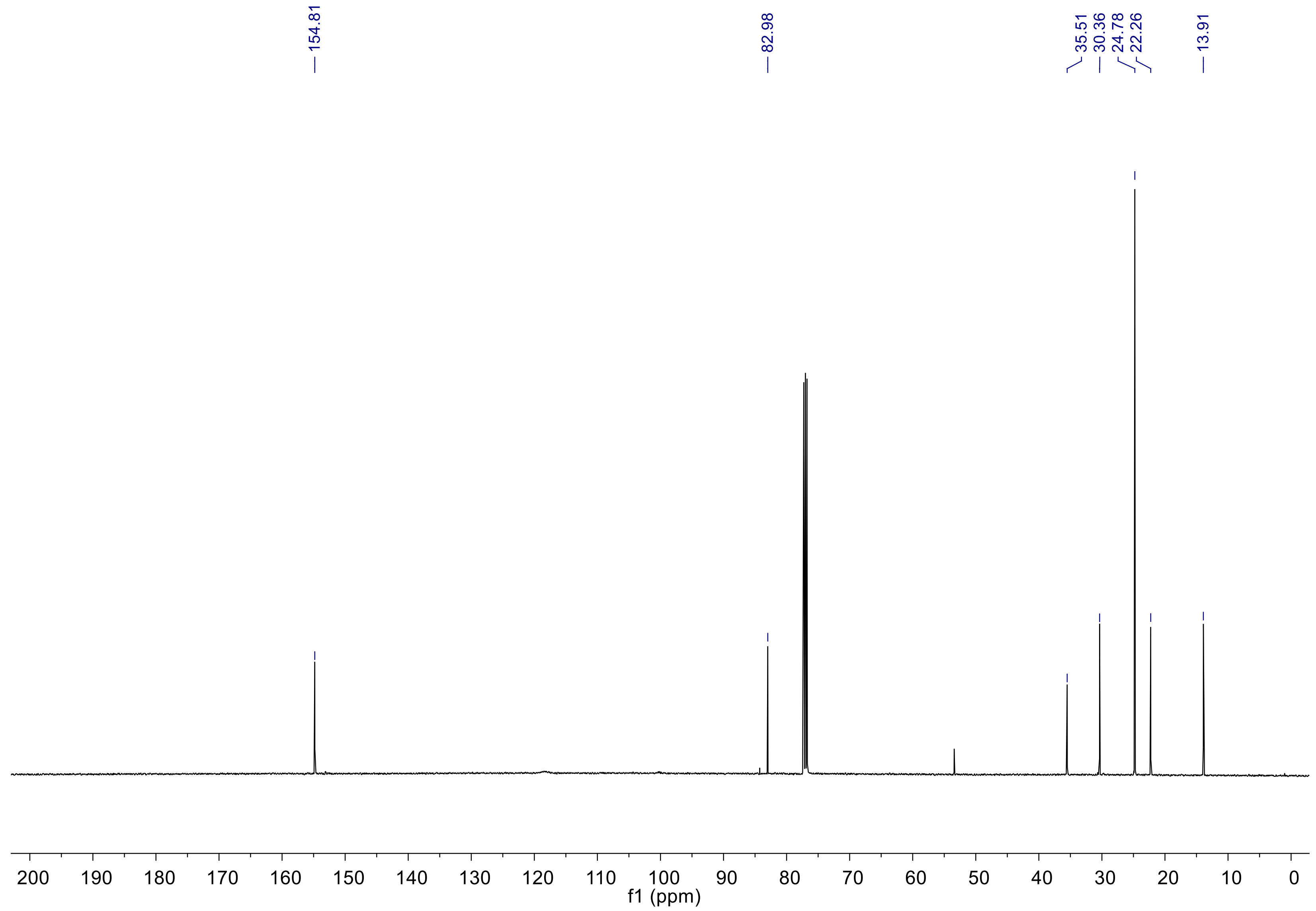

S32 ^{1}H NMR (400 MHz, $CDCl_3$, 298 K) spectrum of *(E)-4,4,5,5-tetramethyl-2-(oct-1-en-1-yl)-1,3,2-dioxaborolane* **1c**.

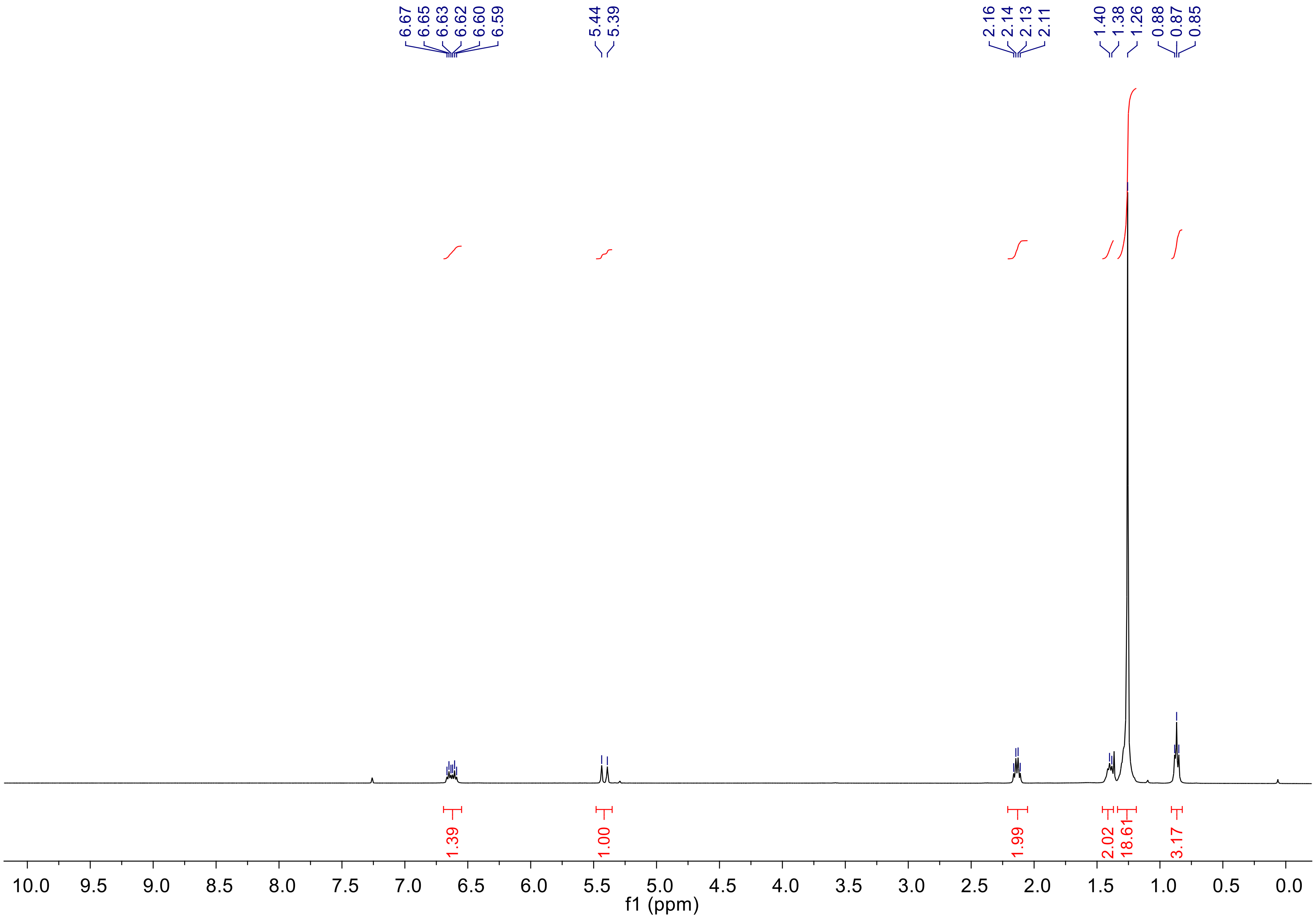

S33 ^{11}B NMR (128 MHz, $CDCl_3$, 298 K) spectrum of *(E)-4,4,5,5-tetramethyl-2-(oct-1-en-1-yl)-1,3,2-dioxaborolane* **1c**.

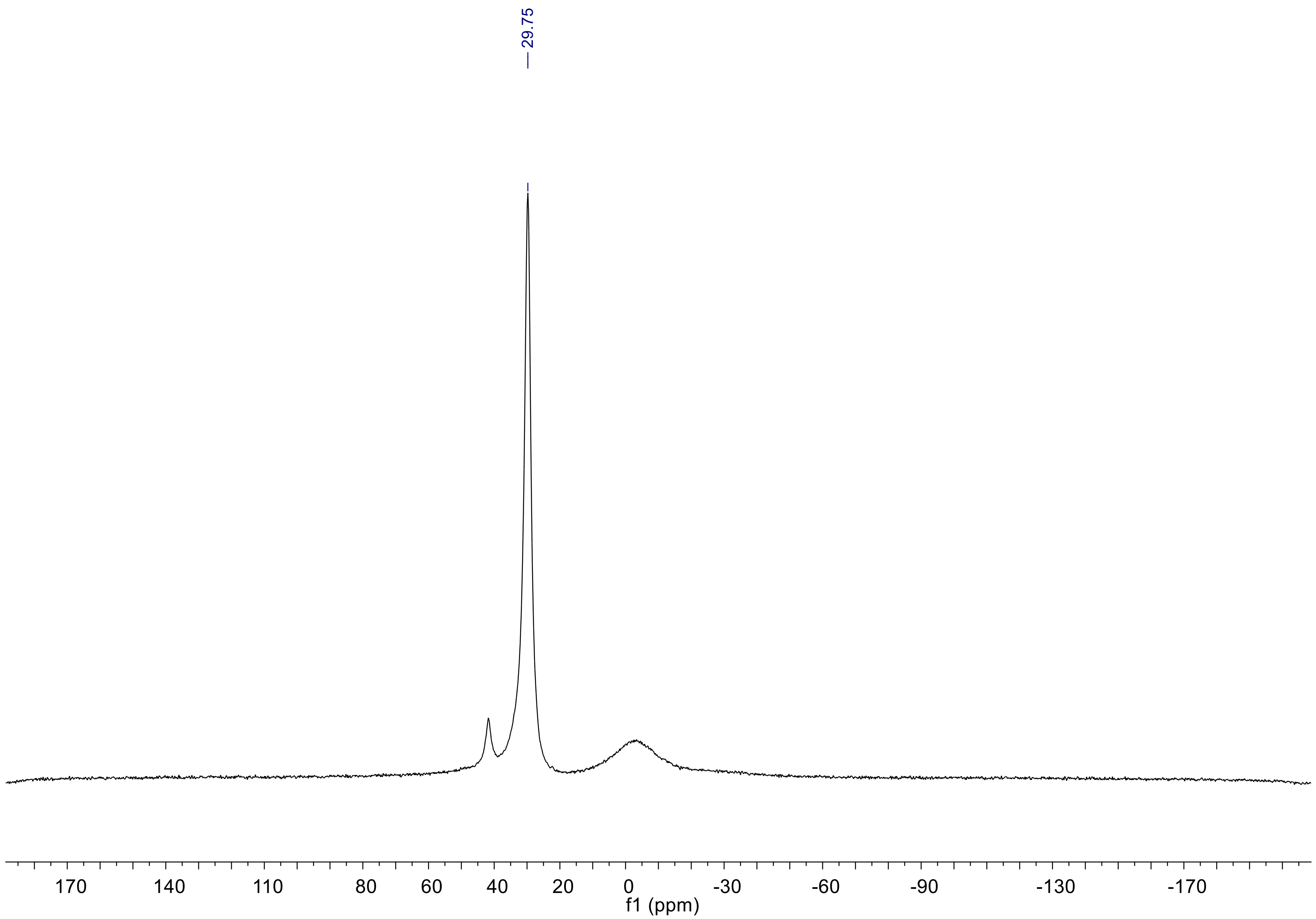

S34 ^{13}C NMR (101 MHz, $CDCl_3$, 298 K) spectrum of *(E)-4,4,5,5-tetramethyl-2-(oct-1-en-1-yl)-1,3,2-dioxaborolane* **1c**.

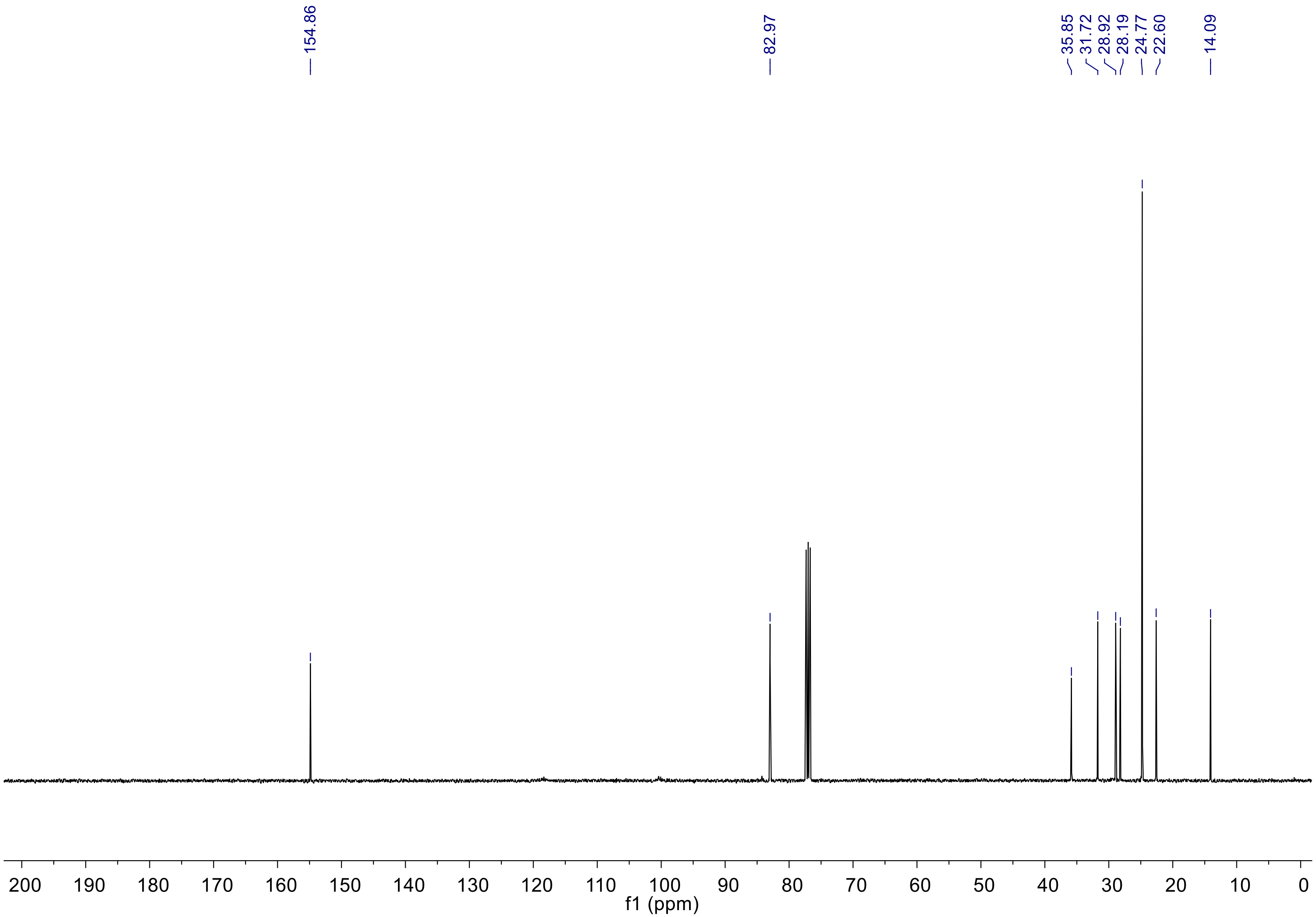

S35 ^{1}H NMR (500 MHz, $CDCl_3$, 298 K) spectrum of *(E)-2-(dec-1-en-1-yl)-4,4,5,5-tetramethyl-1,3,2-dioxaborolane* **1d**.

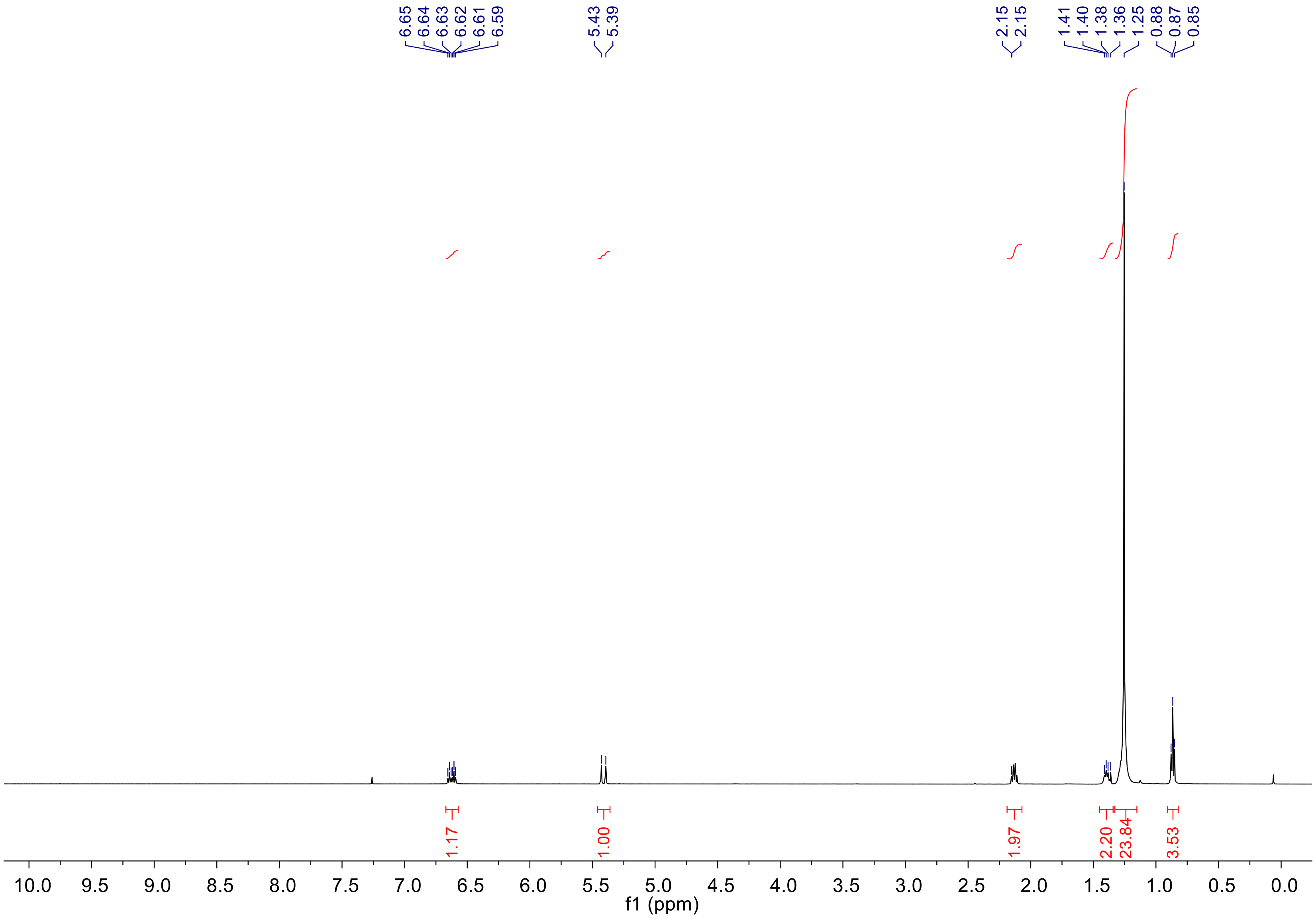

S36 ^{11}B NMR (160 MHz, $CDCl_3$, 298 K) spectrum of *(E)-2-(dec-1-en-1-yl)-4,4,5,5-tetramethyl-1,3,2-dioxaborolane* **1d**.

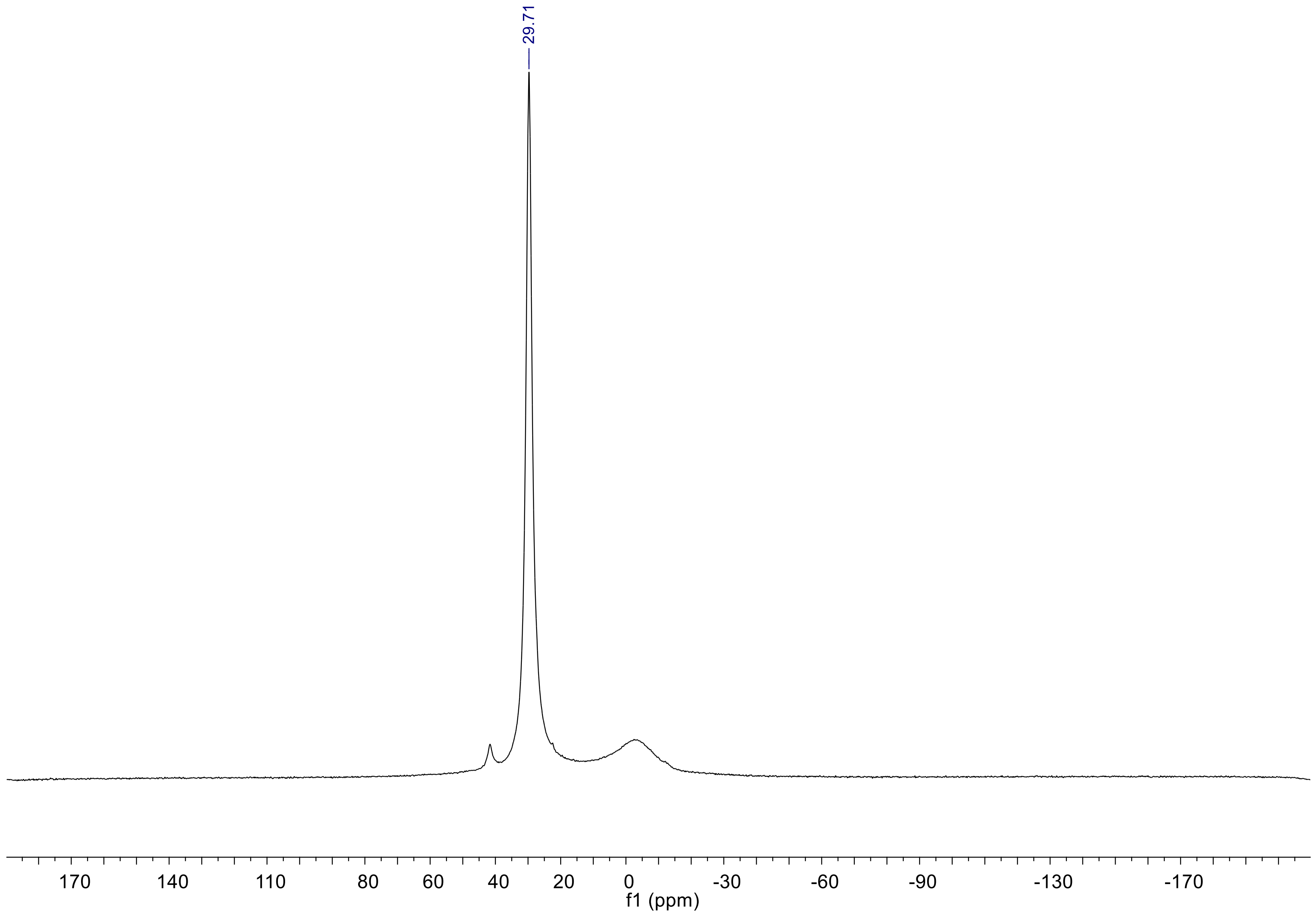

S37 ^{13}C NMR (126 MHz, $CDCl_3$, 298 K) spectrum of *(E)-2-(dec-1-en-1-yl)-4,4,5,5-tetramethyl-1,3,2-dioxaborolane* **1d**.

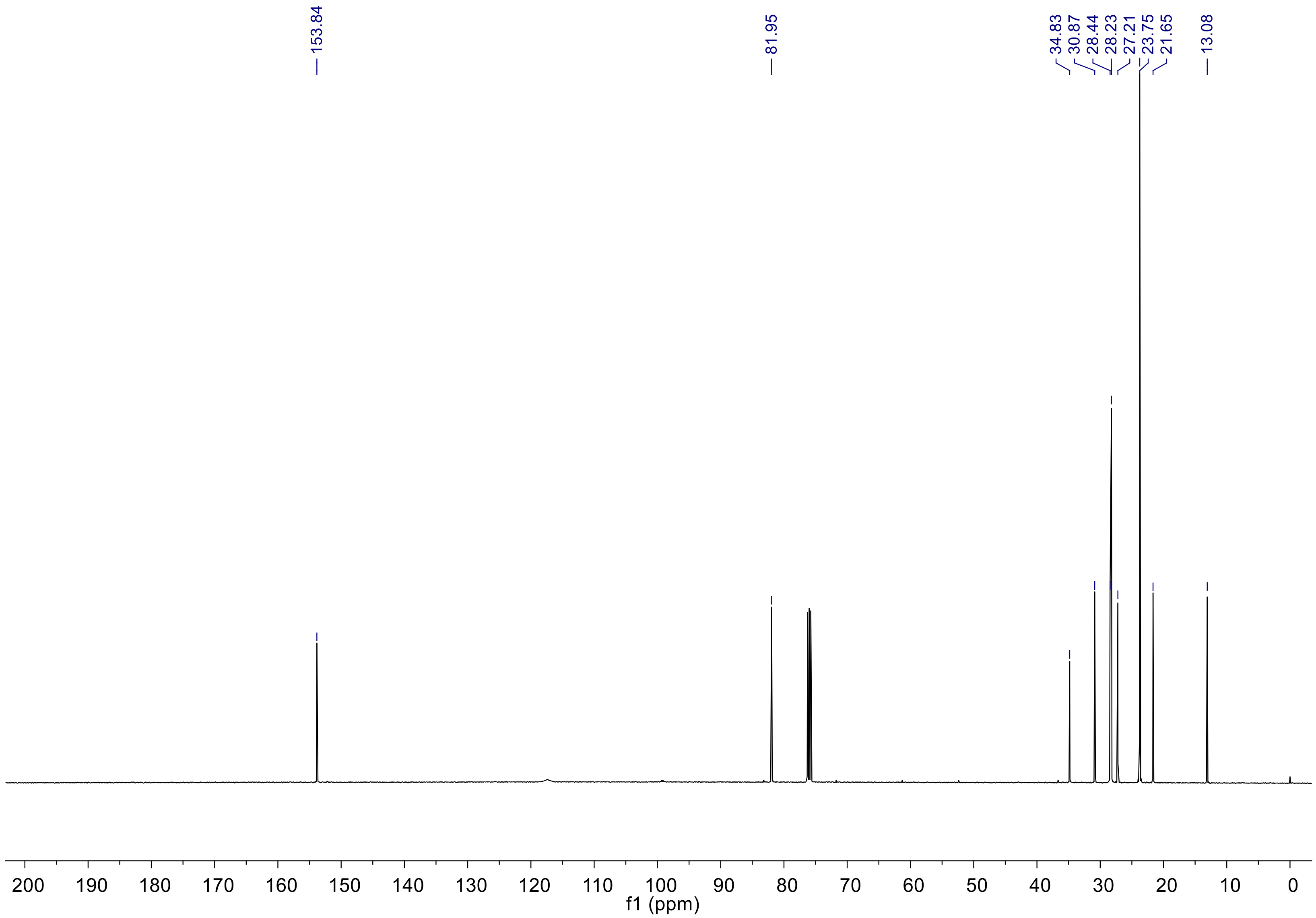

S38 ^{1}H NMR (400 MHz, $CDCl_3$, 298 K) spectrum of *(E)-3-(4,4,5,5-tetramethyl-1,3,2-dioxaborolan-2-yl)allyl benzoate* **1e**.

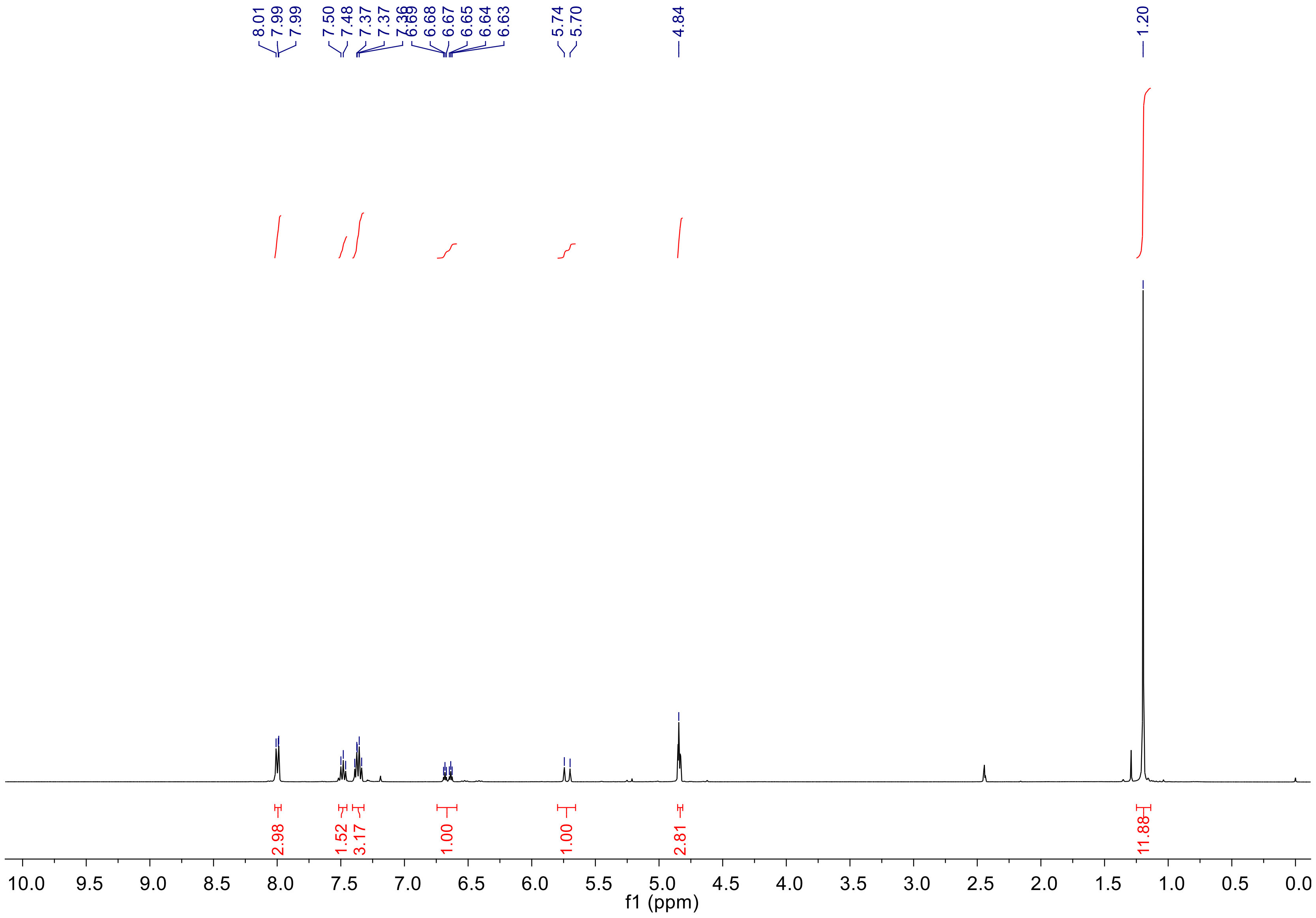

S39 ^{11}B NMR (128 MHz, $CDCl_3$, 298 K) spectrum of *(E)-3-(4,4,5,5-tetramethyl-1,3,2-dioxaborolan-2-yl)allyl benzoate* **1e**.

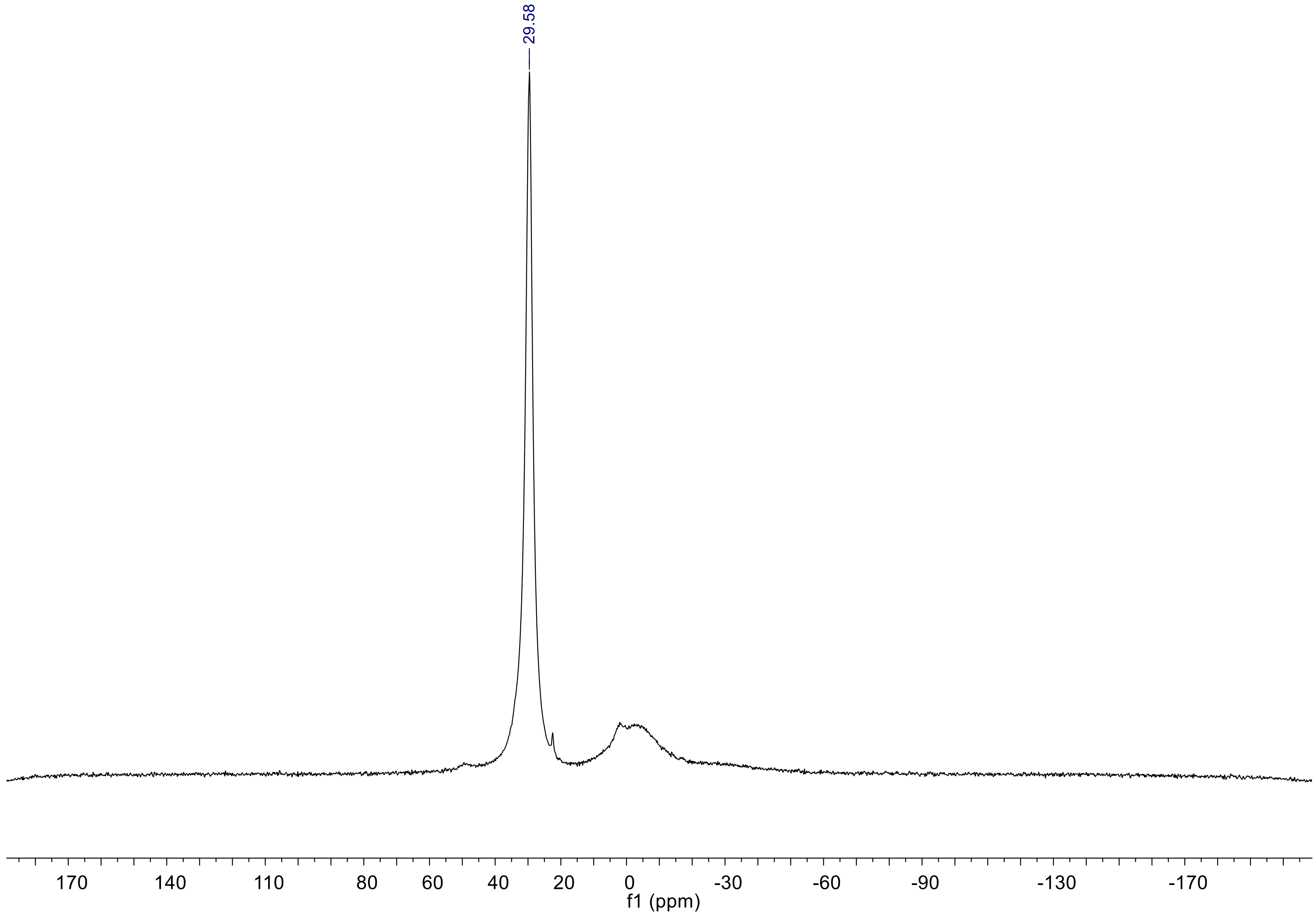

S40 ^{13}C NMR (101 MHz, $CDCl_3$, 298 K) spectrum of *(E)-3-(4,4,5,5-tetramethyl-1,3,2-dioxaborolan-2-yl)allyl benzoate* **1e**.

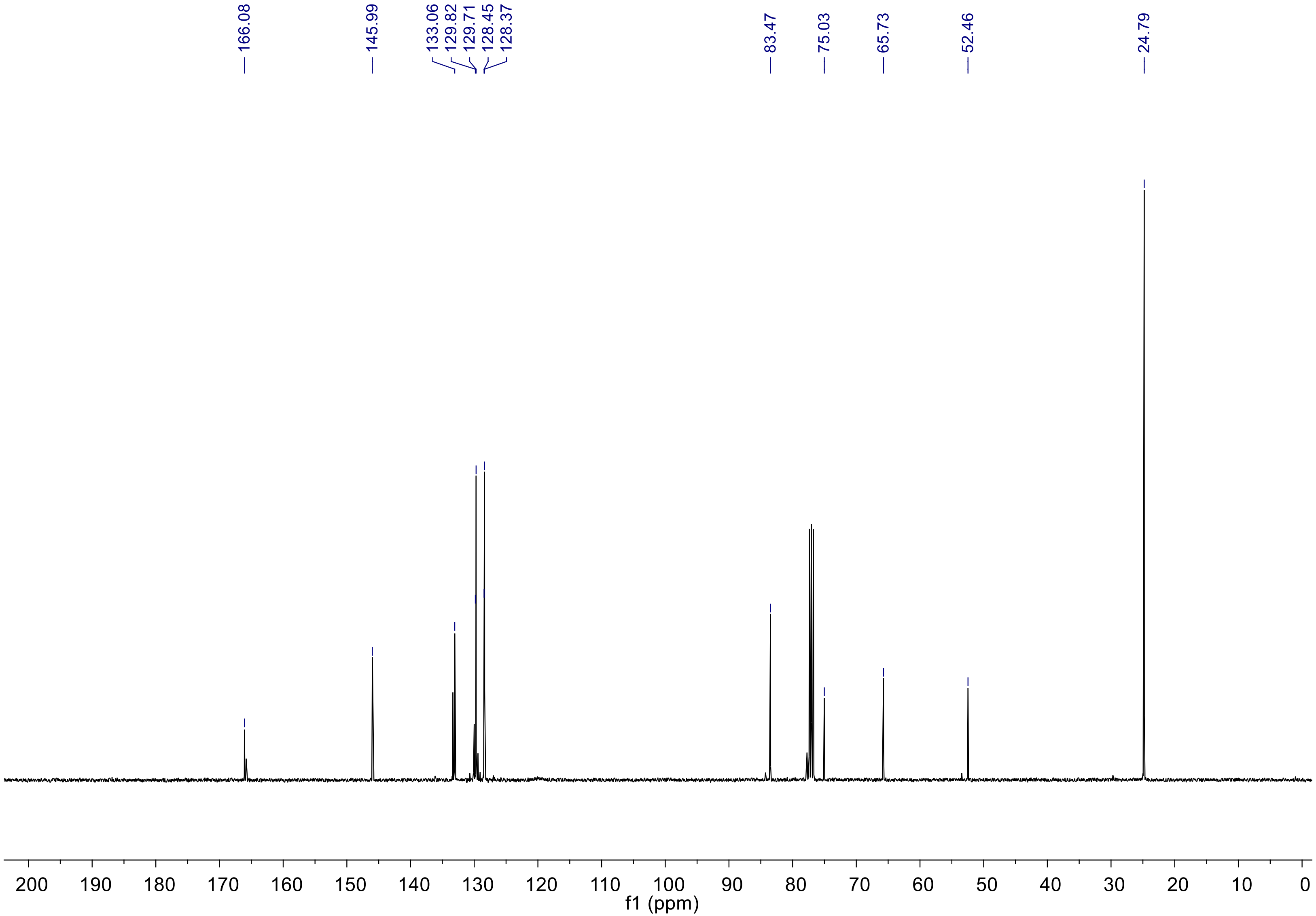

S41 ^{1}H NMR (500 MHz, $CDCl_3$, 298 K) spectrum of *(E)-3-(4,4,5,5-tetramethyl-1,3,2-dioxaborolan-2-yl)allyl 4-methylbenzoate* **1f**.

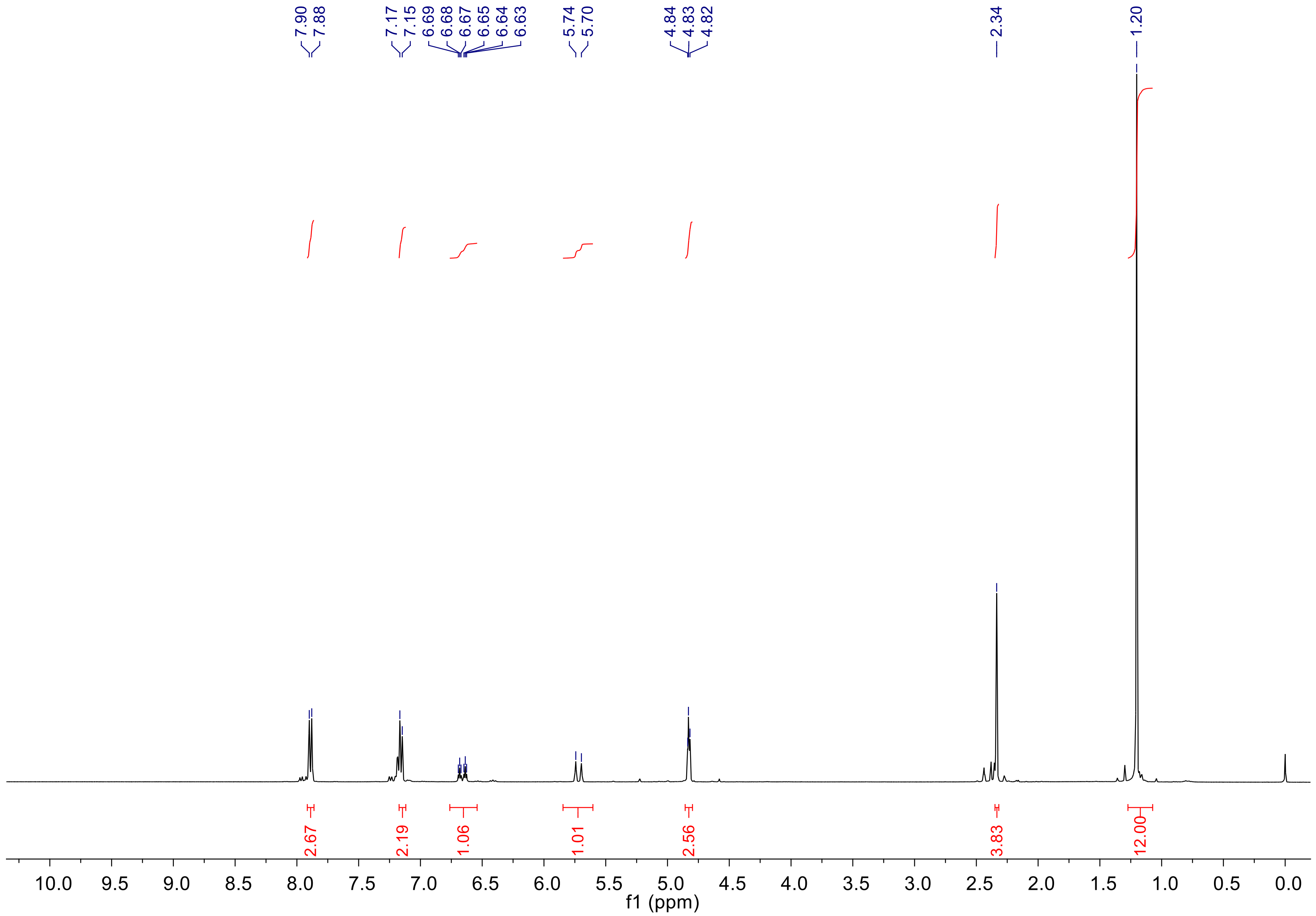

S42 ^{11}B NMR (160 MHz, $CDCl_3$, 298 K) spectrum of *(E)-3-(4,4,5,5-tetramethyl-1,3,2-dioxaborolan-2-yl)allyl 4-methylbenzoate* **1f**.

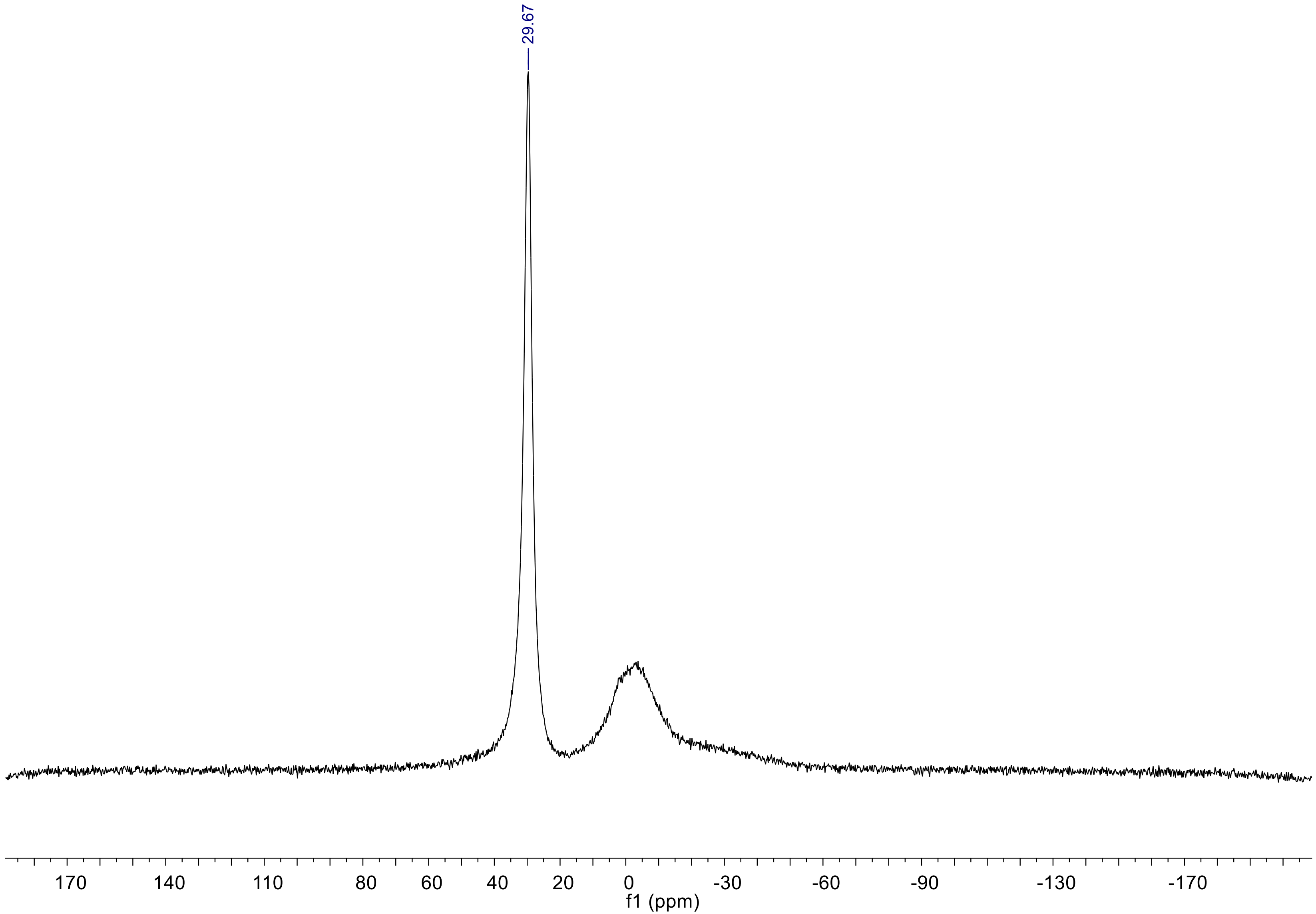

S43 ^{13}C NMR (126 MHz, $CDCl_3$, 298 K) spectrum of *(E)-3-(4,4,5,5-tetramethyl-1,3,2-dioxaborolan-2-yl)allyl 4-methylbenzoate* **1f**.

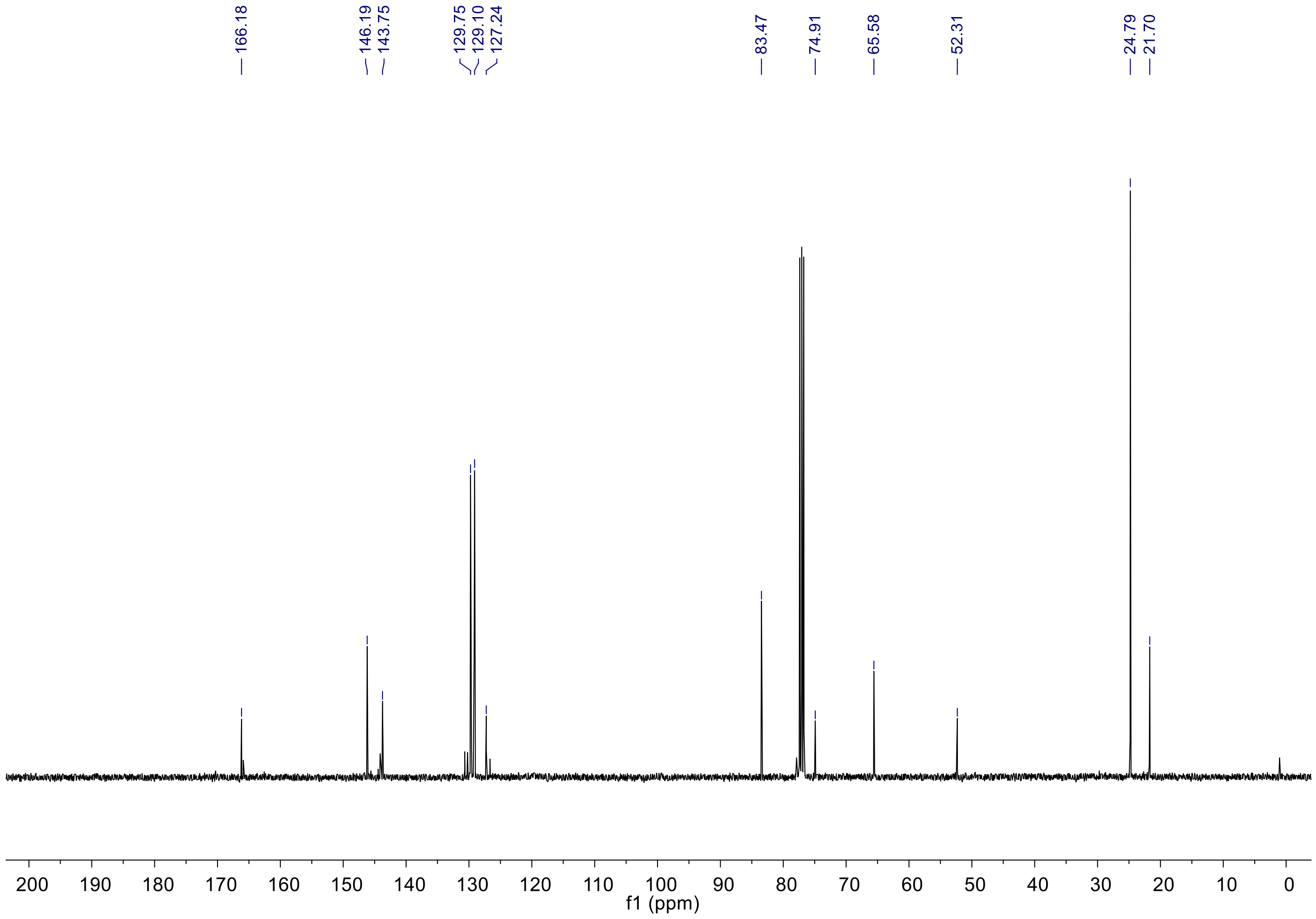

S44 ^{1}H NMR (400 MHz, $CDCl_3$, 298 K) spectrum of *(E)-3-(4,4,5,5-tetramethyl-1,3,2-dioxaborolan-2-yl)allyl 4-nitrobenzoate* **1g**.

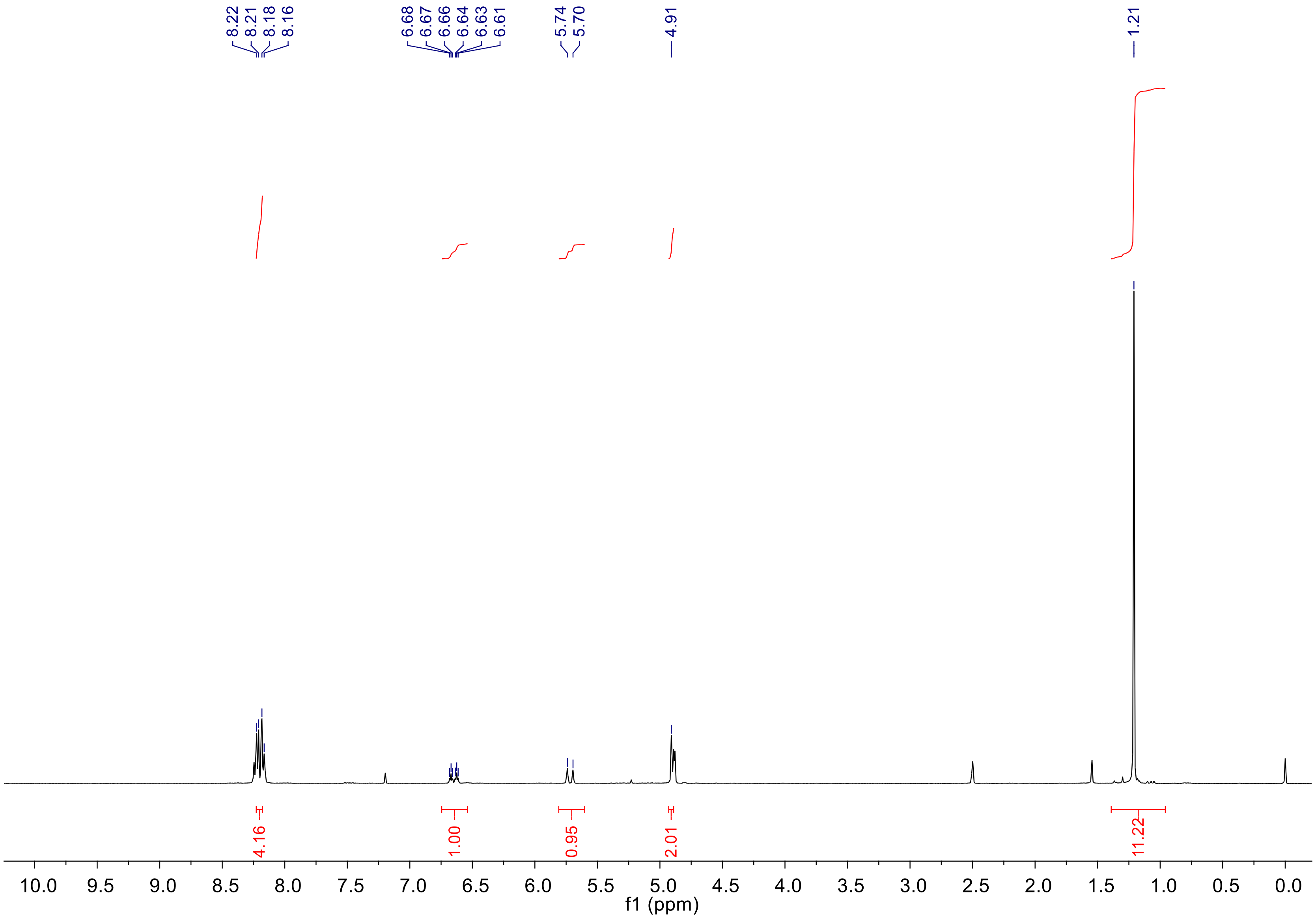

S45 ^{11}B NMR (128 MHz, $CDCl_3$, 298 K) spectrum of *(E)-3-(4,4,5,5-tetramethyl-1,3,2-dioxaborolan-2-yl)allyl 4-nitrobenzoate* **1g**.

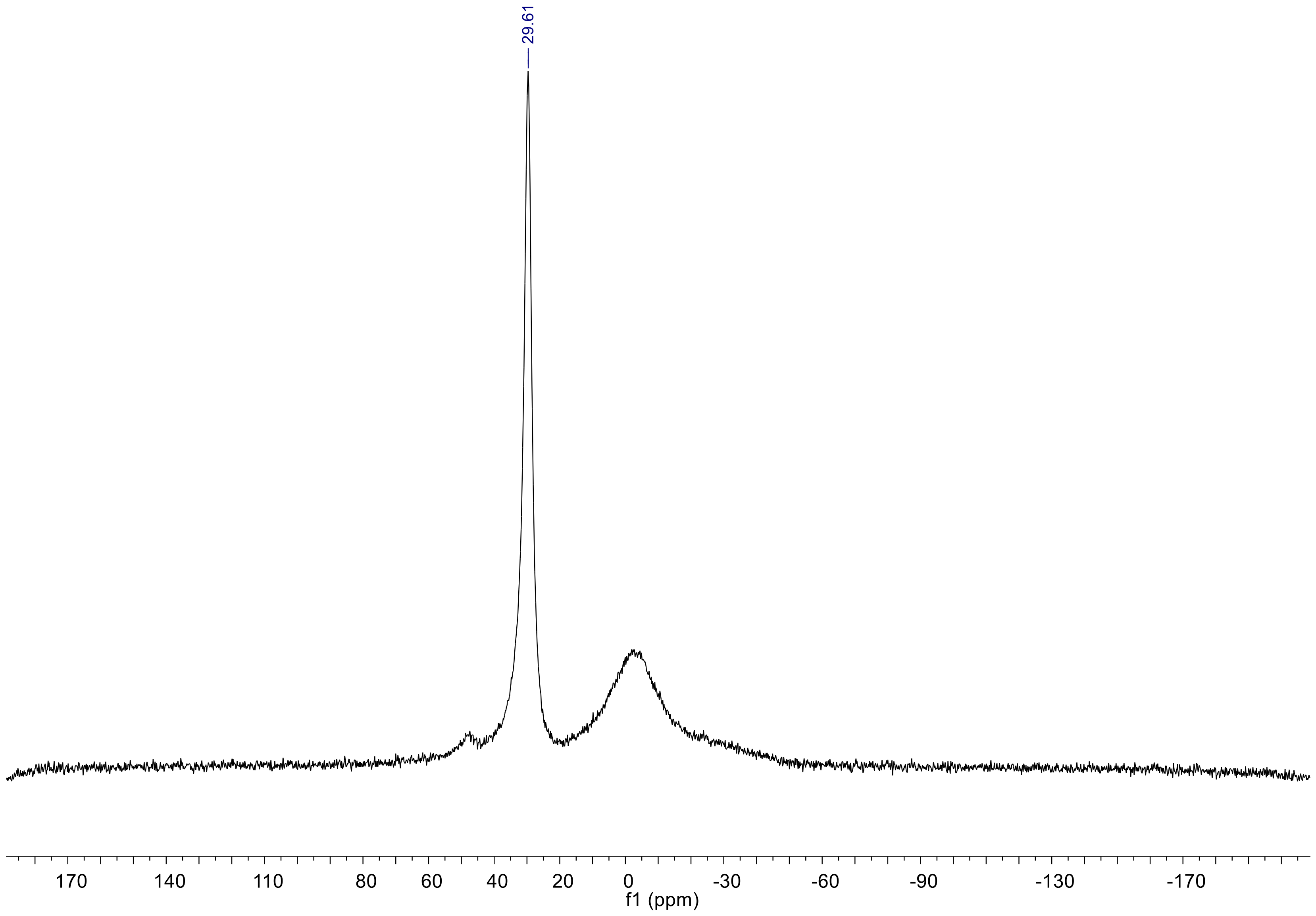

S46 ^{13}C NMR (101 MHz, $CDCl_3$, 298 K) spectrum of *(E)-3-(4,4,5,5-tetramethyl-1,3,2-dioxaborolan-2-yl)allyl 4-nitrobenzoate* **1g**.

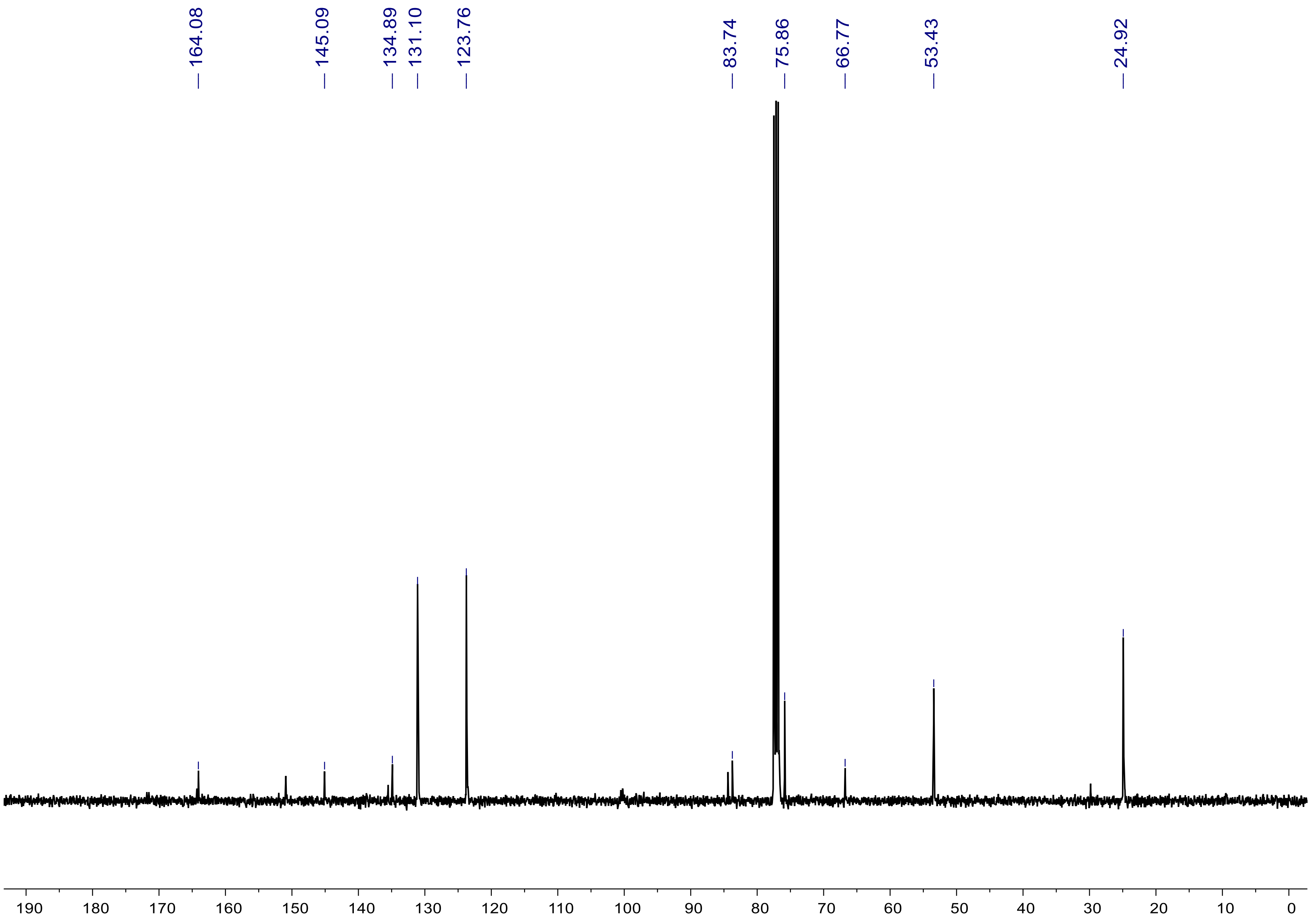

S47 ^1H NMR (400 MHz, $CDCl_3$, 298 K) spectrum of *(E)-3-(4,4,5,5-tetramethyl-1,3,2-dioxaborolan-2-yl)allyl 4-methoxybenzoate* **1h**.

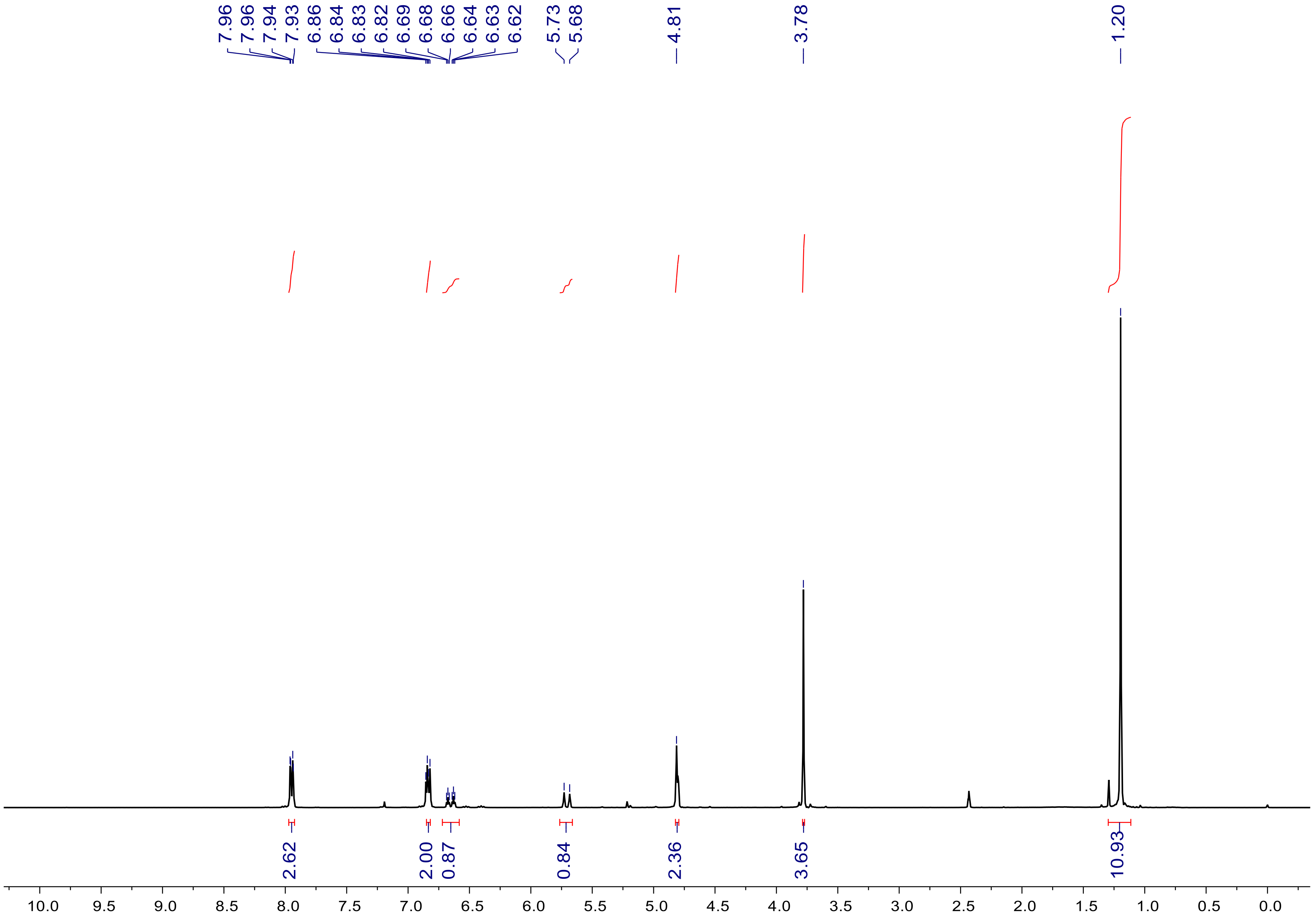

S48 ^{11}B NMR (128 MHz, $CDCl_3$, 298 K) spectrum of *(E)-3-(4,4,5,5-tetramethyl-1,3,2-dioxaborolan-2-yl)allyl 4-methoxybenzoate* **1h**.

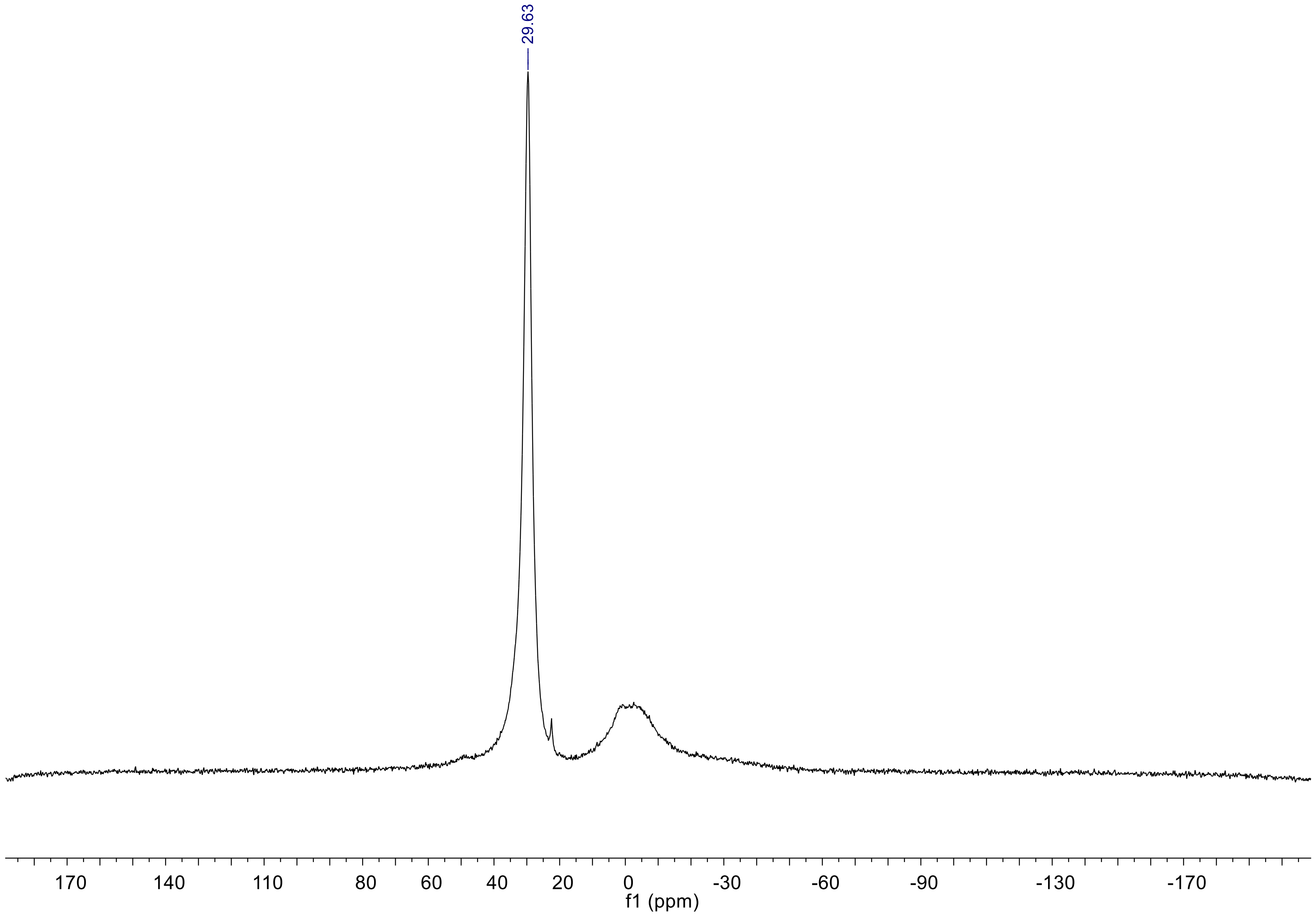

S49 ^{13}C NMR (101 MHz, $CDCl_3$, 298 K) spectrum of *(E)-3-(4,4,5,5-tetramethyl-1,3,2-dioxaborolan-2-yl)allyl 4-methoxybenzoate* **1h**.

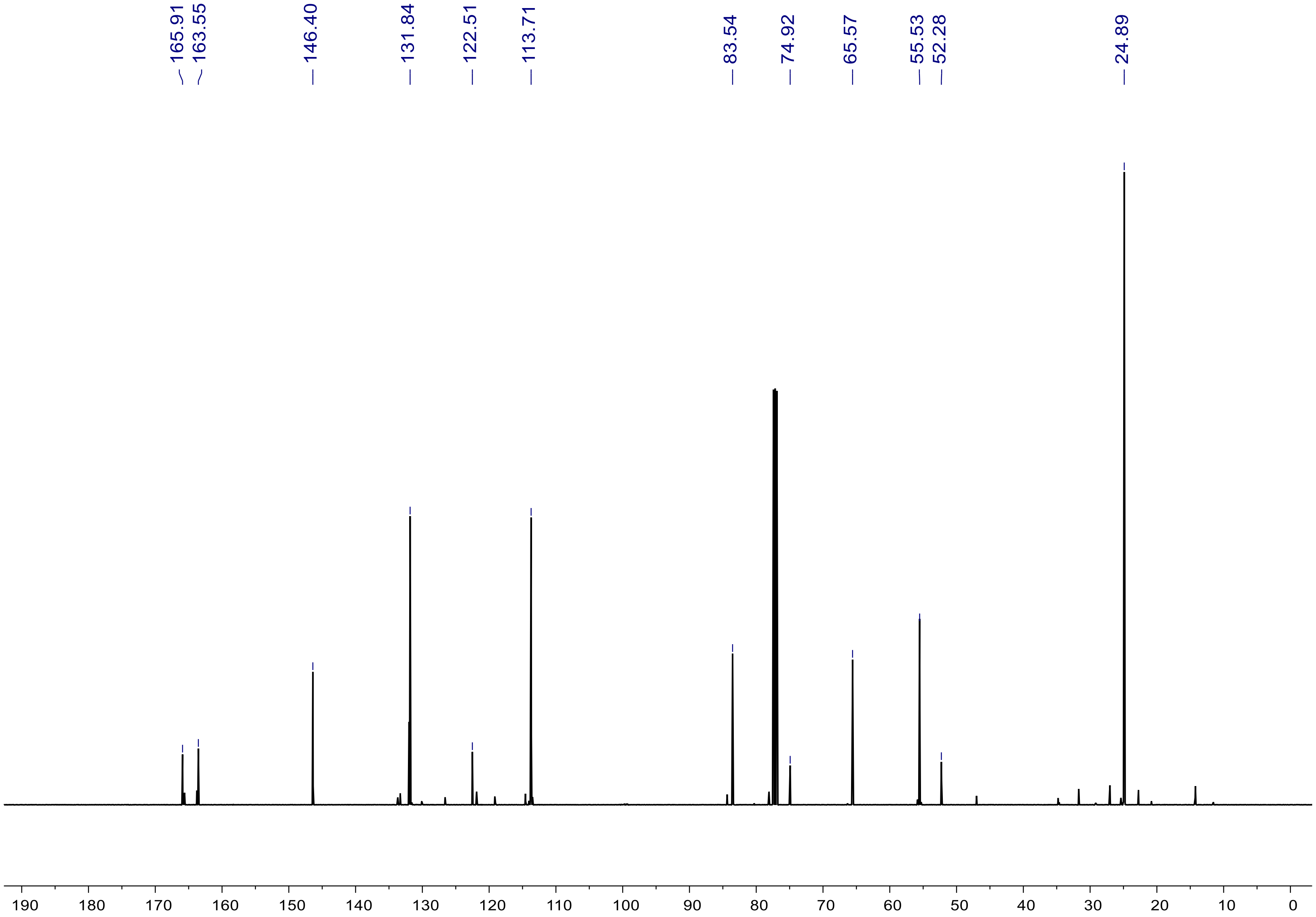

S50 ^{1}H NMR (400 MHz, $CDCl_3$, 298 K) spectrum of *(E)-3-(4,4,5,5-tetramethyl-1,3,2-dioxaborolan-2-yl)allyl acrylate* **1i**.

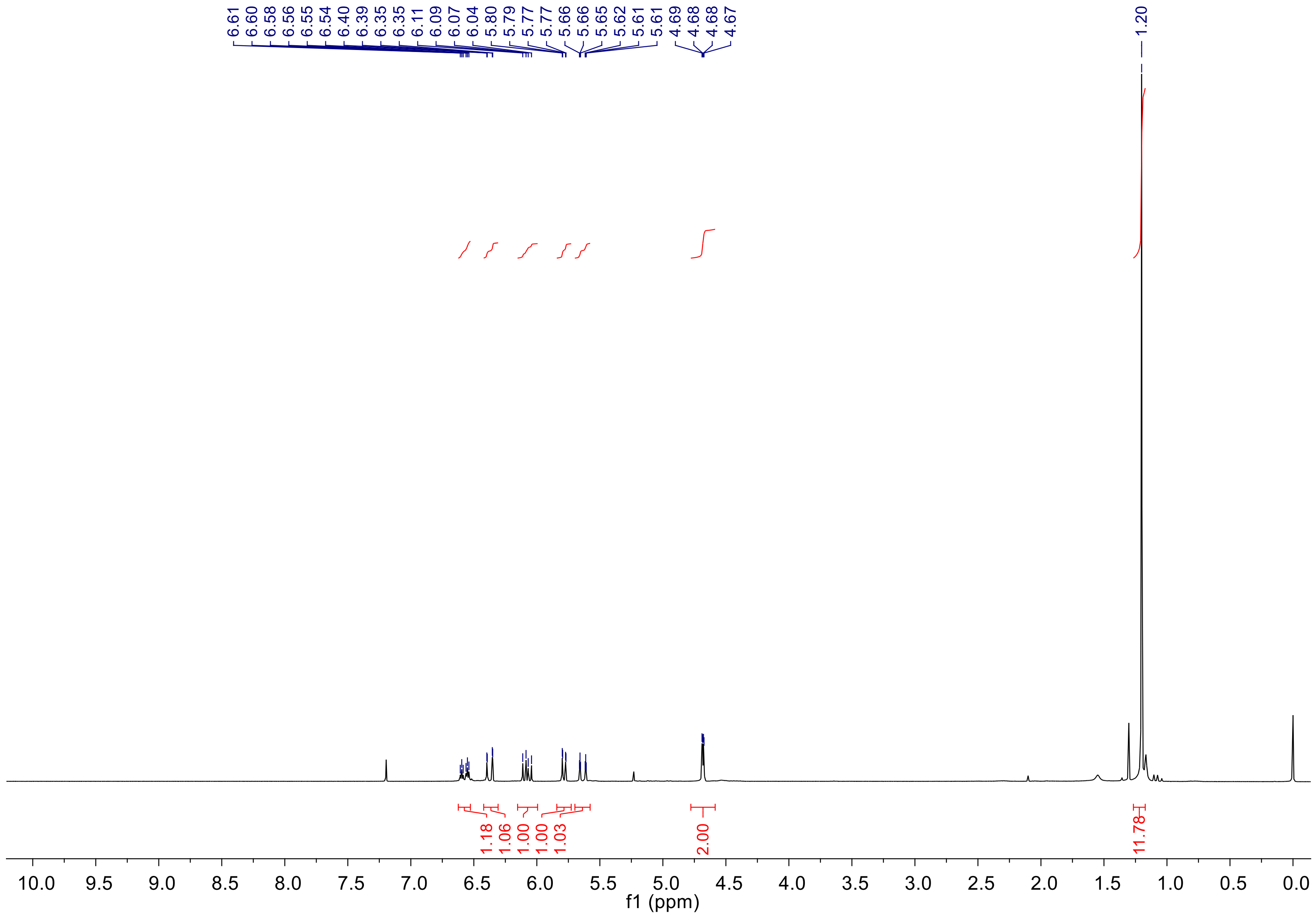

S51 ^{11}B NMR (128 MHz, $CDCl_3$, 298 K) spectrum of *(E)-3-(4,4,5,5-tetramethyl-1,3,2-dioxaborolan-2-yl)allyl acrylate* **1i**.

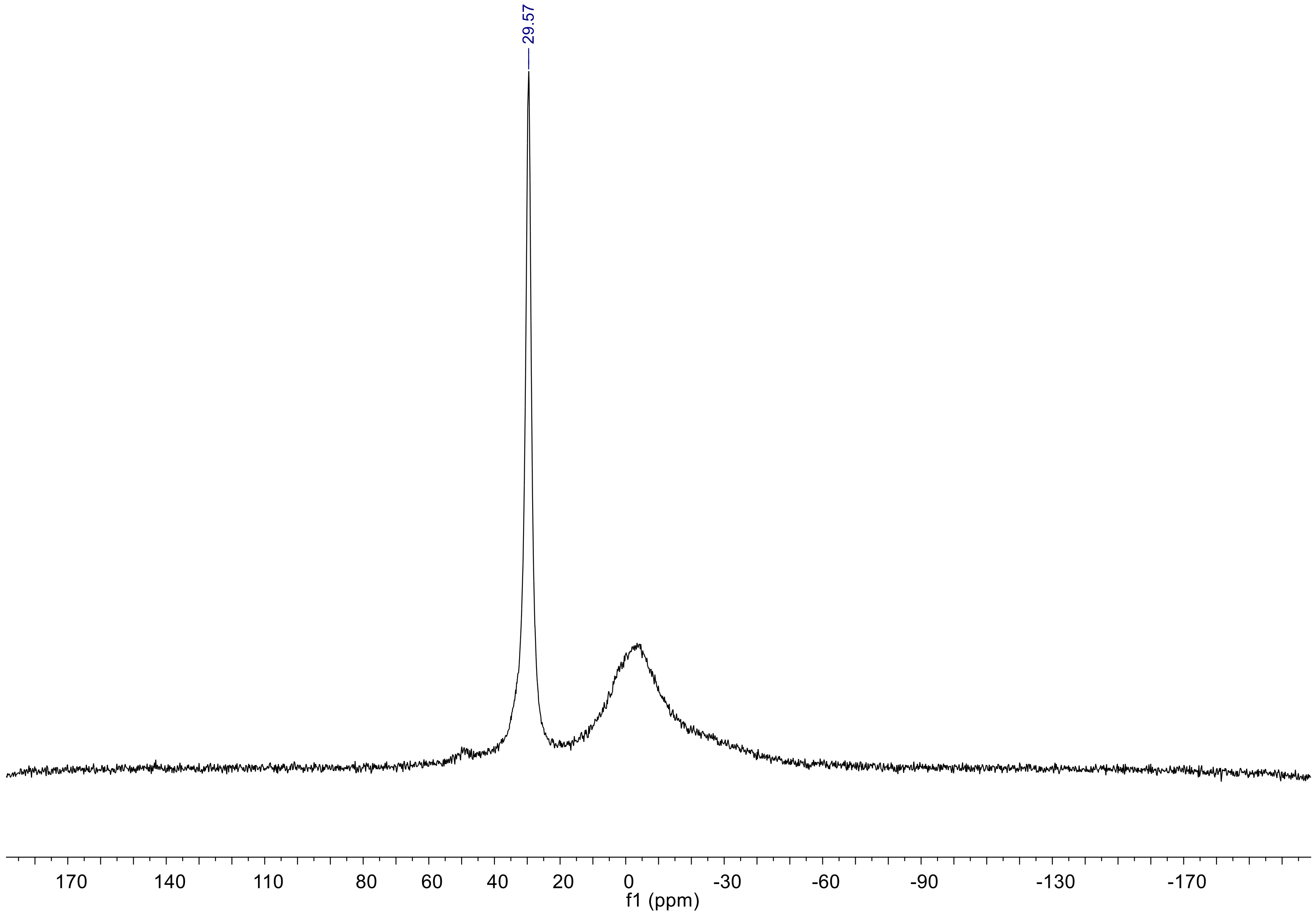

S52 ^{13}C NMR (101 MHz, $CDCl_3$, 298 K) spectrum of *(E)-3-(4,4,5,5-tetramethyl-1,3,2-dioxaborolan-2-yl)allyl acrylate* **1i**.

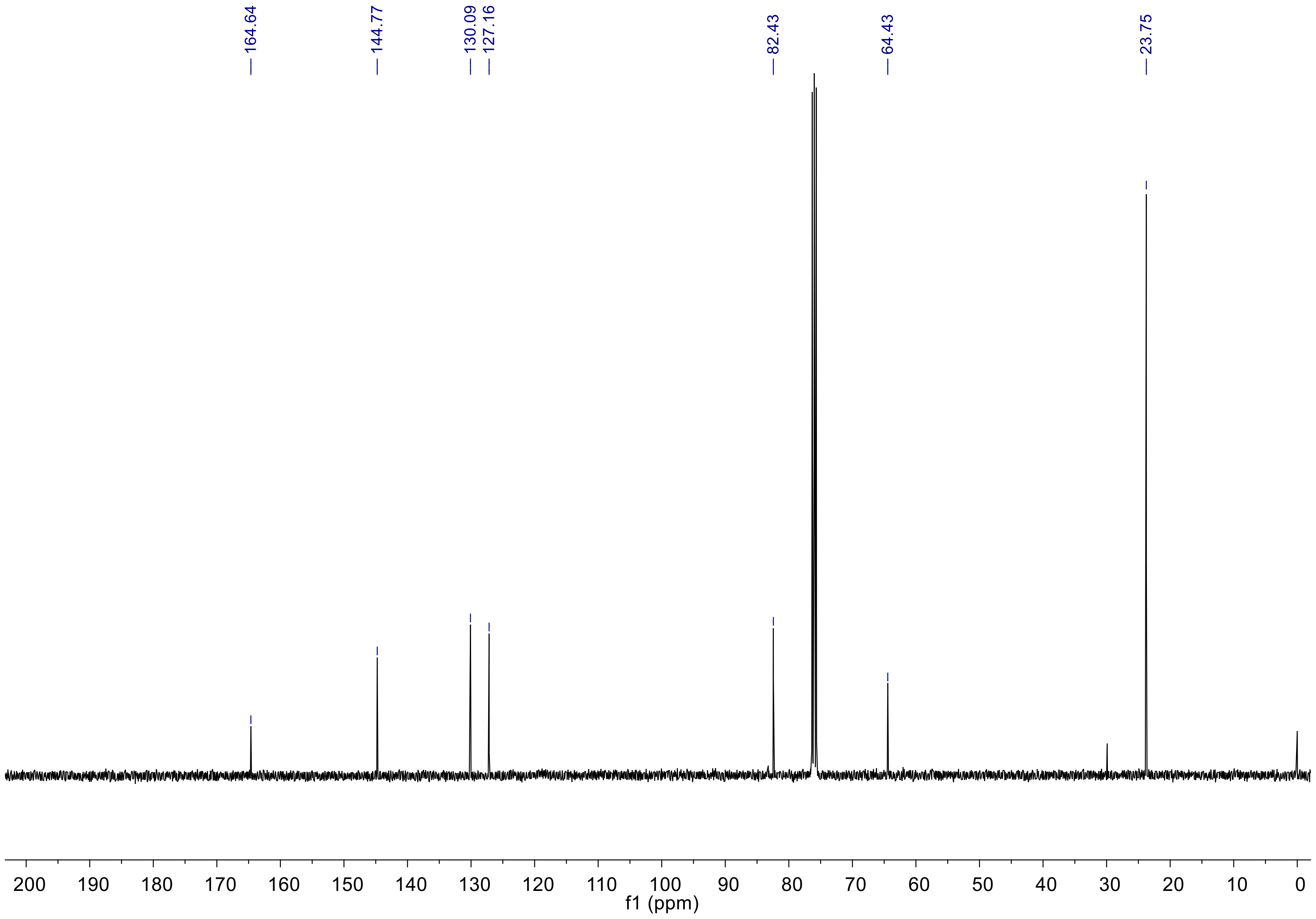

S53 ^{1}H NMR (400 MHz, $CDCl_3$, 298 K) spectrum of *(E)-trimethyl(2-(4,4,5,5-tetramethyl-1,3,2-dioxaborolan-2-yl)vinyl)silane* **1j**.

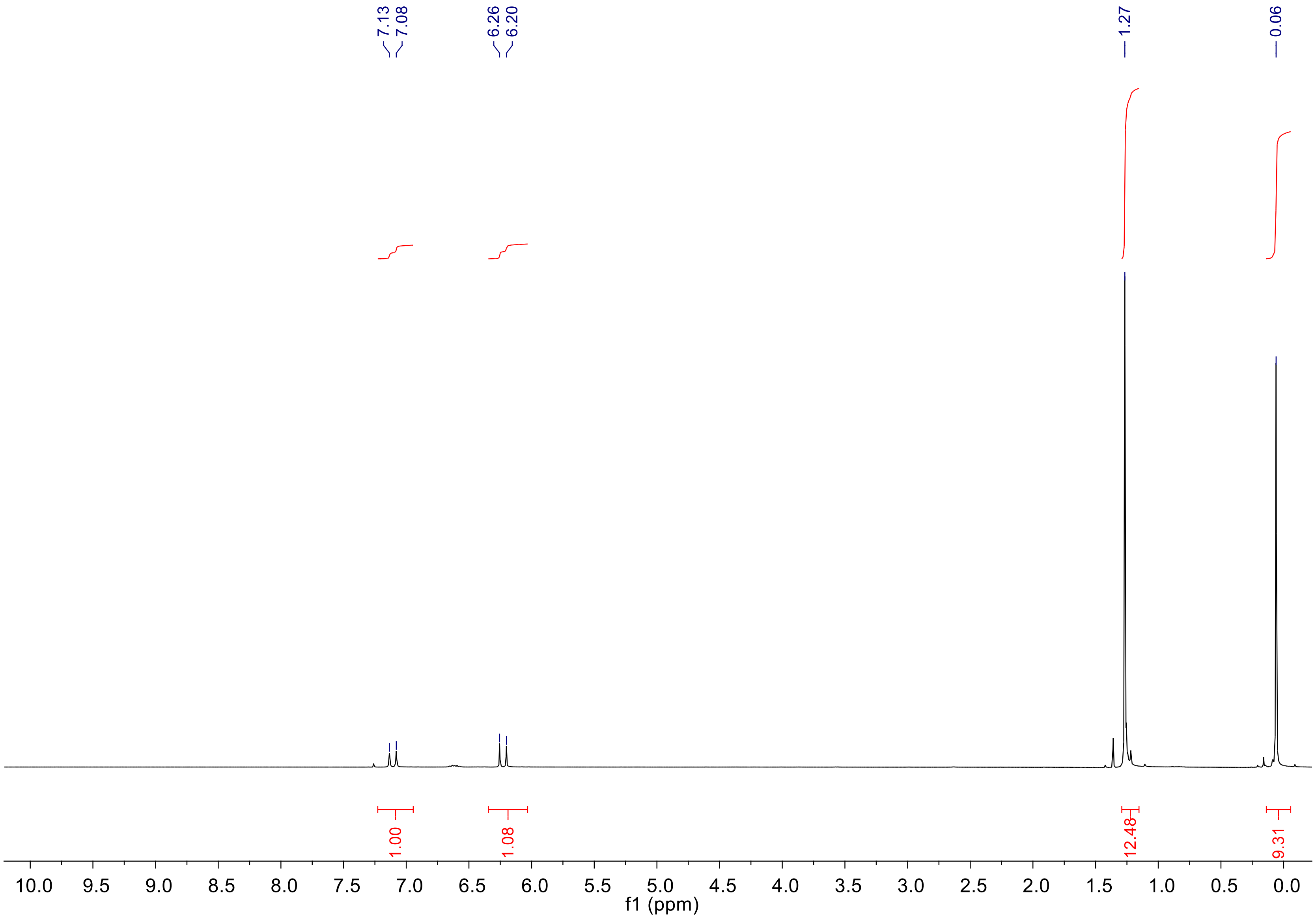

S54 ^{11}B NMR (128 MHz, $CDCl_3$, 298 K) spectrum of *(E)-trimethyl(2-(4,4,5,5-tetramethyl-1,3,2-dioxaborolan-2-yl)vinyl)silane* **1j**.

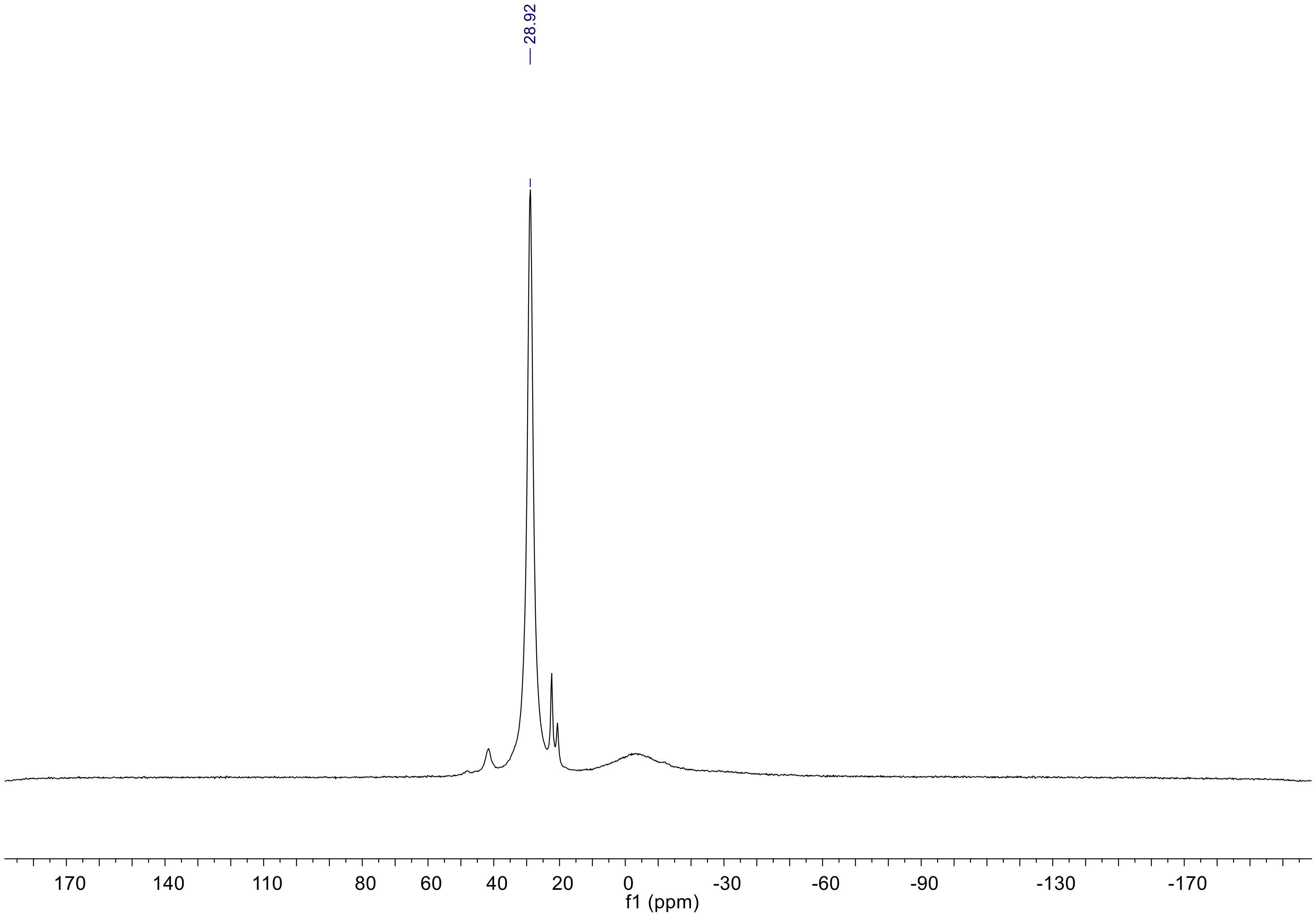

S55 ^{13}C NMR (101 MHz, $CDCl_3$, 298 K) spectrum of *(E)-trimethyl(2-(4,4,5,5-tetramethyl-1,3,2-dioxaborolan-2-yl)vinyl)silane* **1j**.

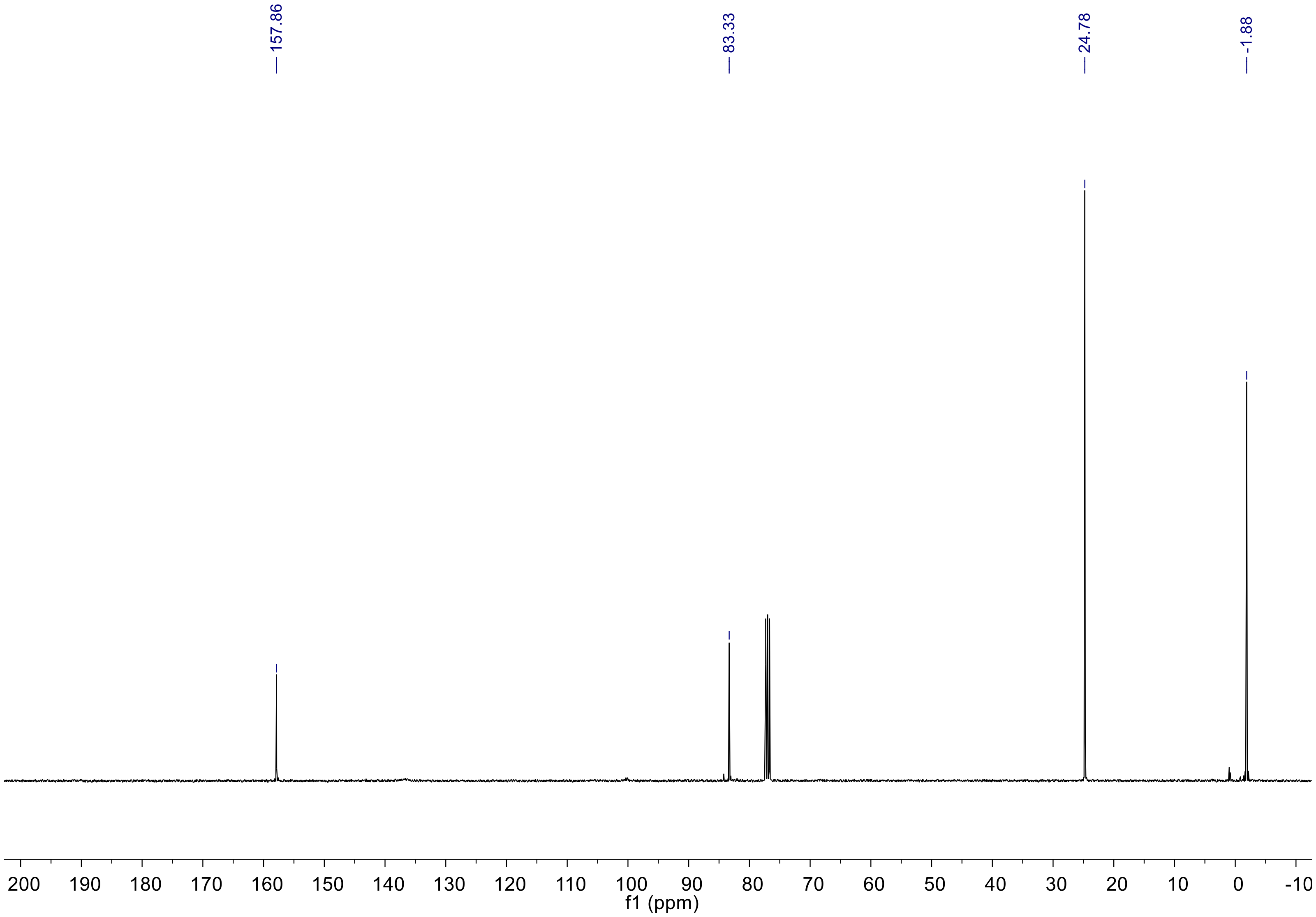

S56 ^{29}Si NMR (80 MHz, $CDCl_3$, 298 K) spectrum of *(E)-trimethyl(2-(4,4,5,5-tetramethyl-1,3,2-dioxaborolan-2-yl)vinyl)silane* **1j**.

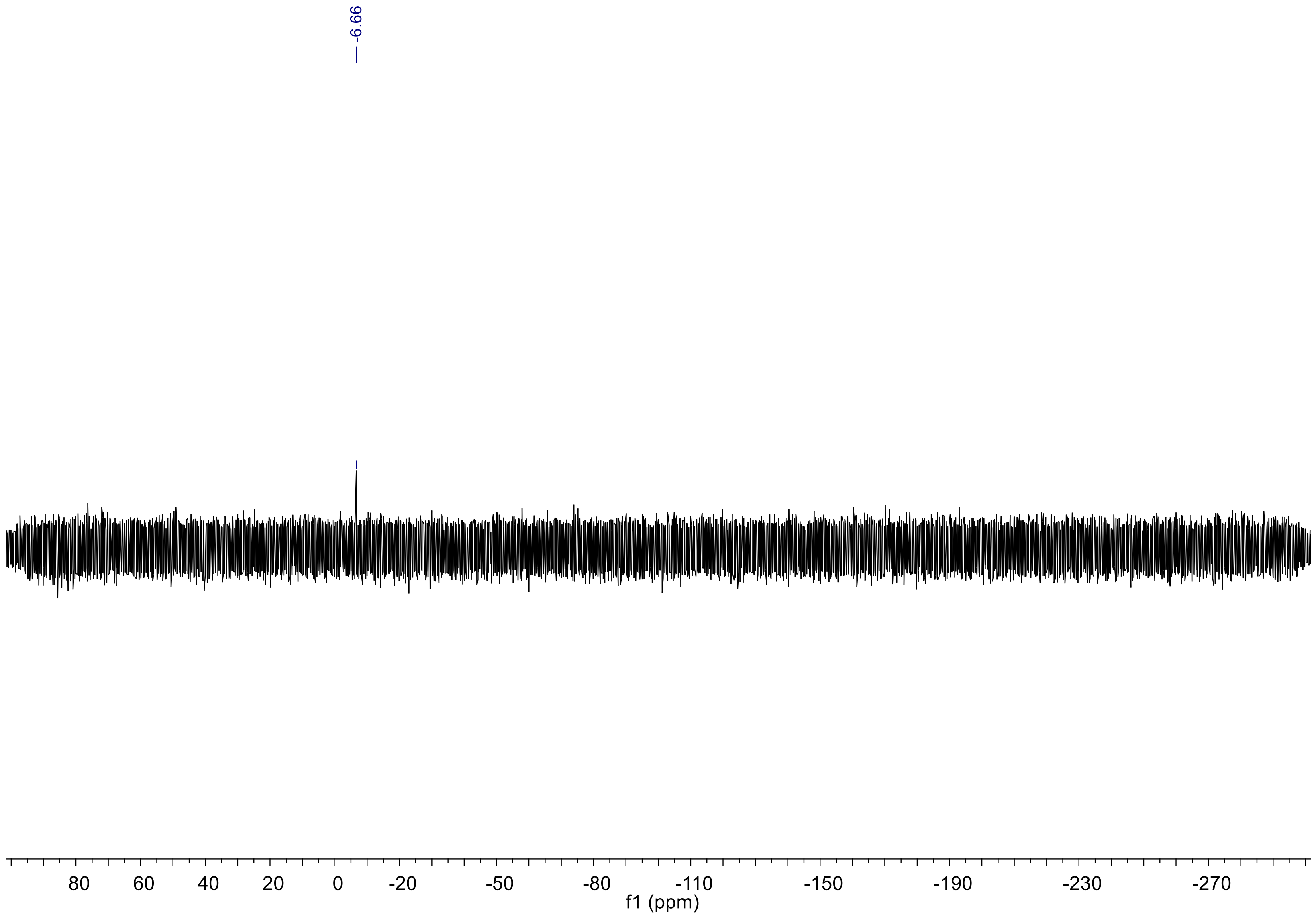

S57 ^{1}H NMR (400 MHz, $CDCl_3$, 298 K) spectrum of *(E)-trimethyl(3-(4,4,5,5-tetramethyl-1,3,2-dioxaborolan-2-yl)allyl)silane* **1k**.

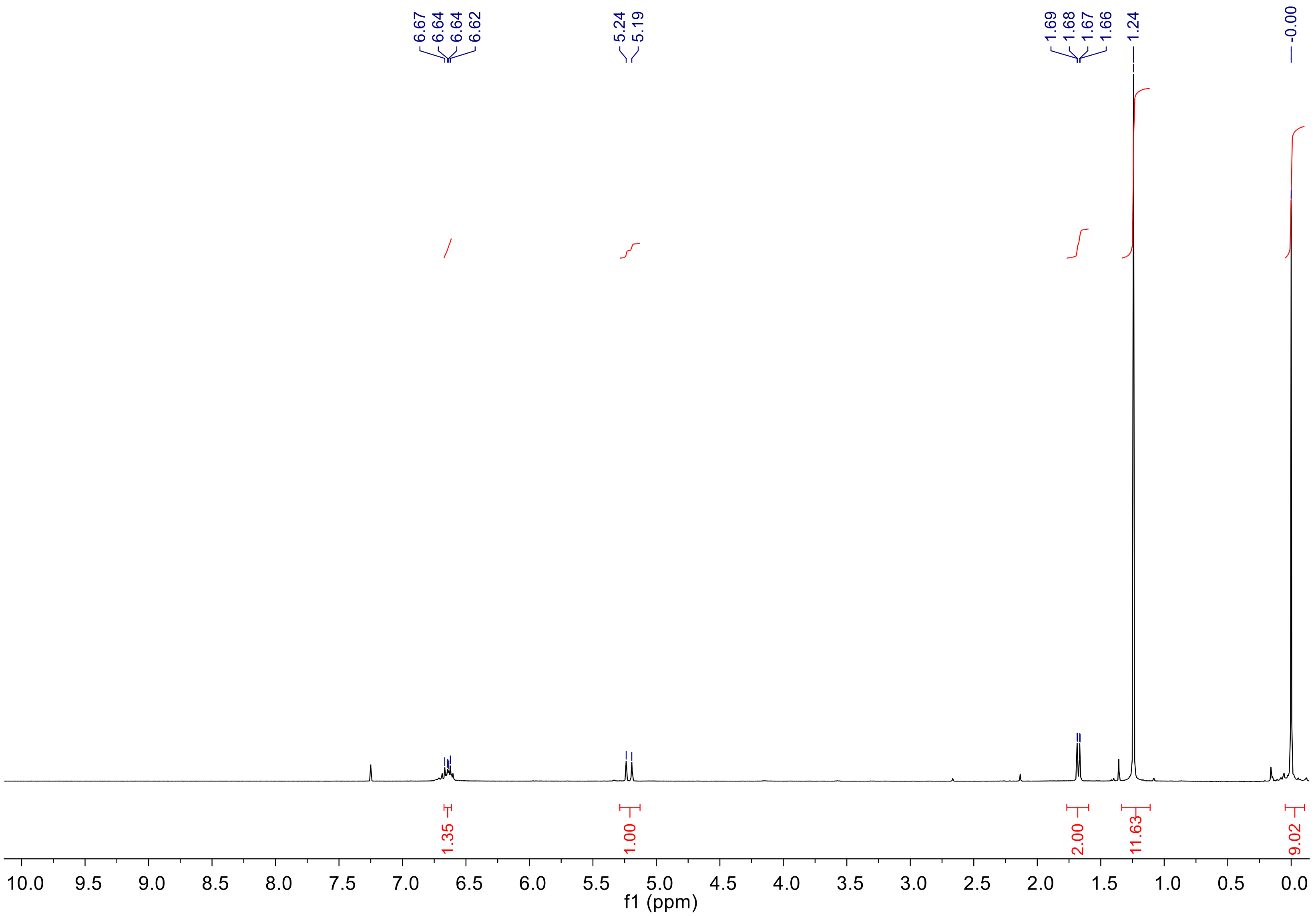

S58 ^{11}B NMR (128 MHz, $CDCl_3$, 298 K) spectrum of *(E)-trimethyl(3-(4,4,5,5-tetramethyl-1,3,2-dioxaborolan-2-yl)allyl)silane* **1k**.

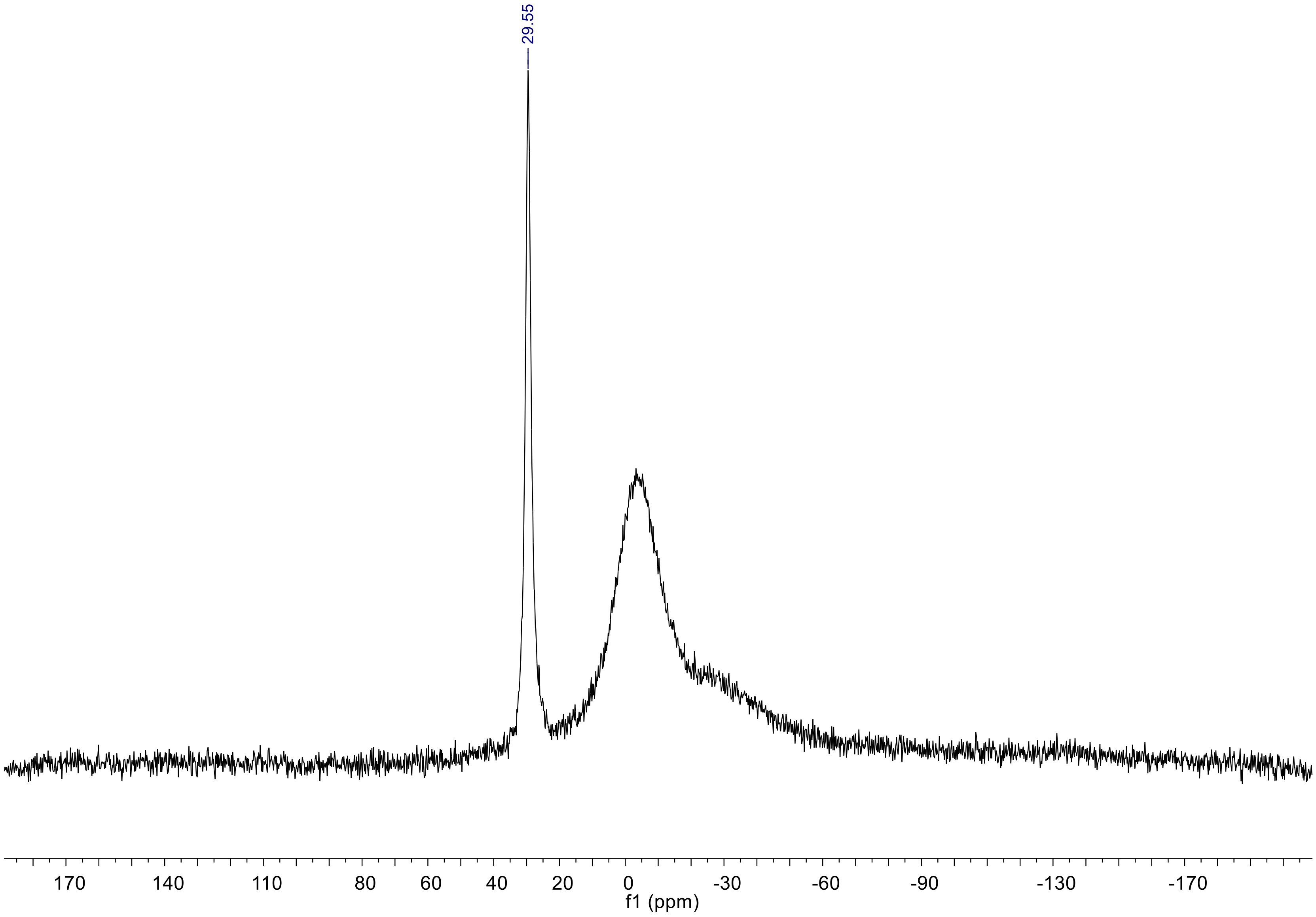

S59 ^{13}C NMR (101 MHz, $CDCl_3$, 298 K) spectrum of *(E)-trimethyl(3-(4,4,5,5-tetramethyl-1,3,2-dioxaborolan-2-yl)allyl)silane* **1k**.

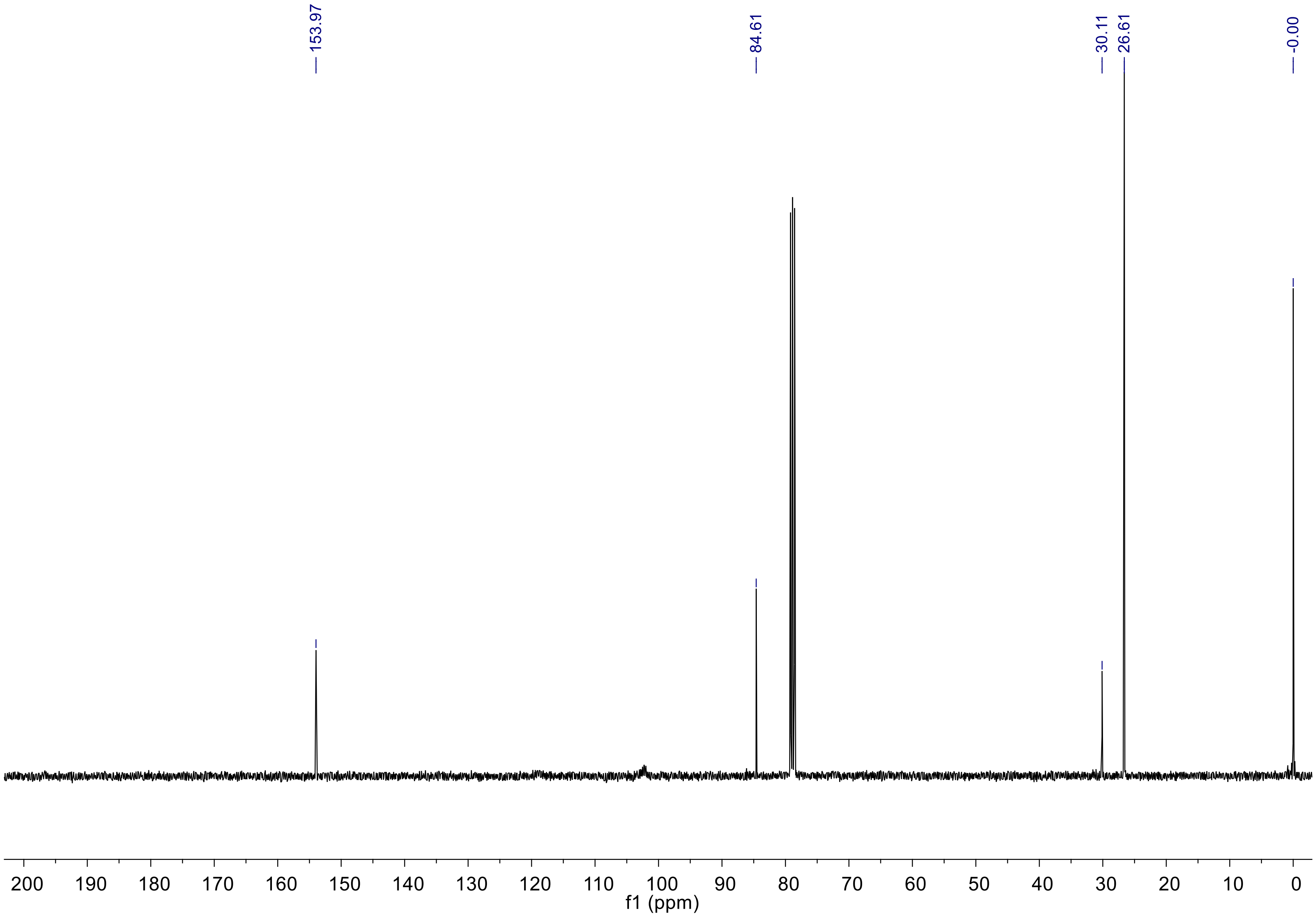

S60 ^{29}Si NMR (80 MHz, $CDCl_3$, 298 K) spectrum of *(E)-trimethyl(3-(4,4,5,5-tetramethyl-1,3,2-dioxaborolan-2-yl)allyl)silane* **1k**.

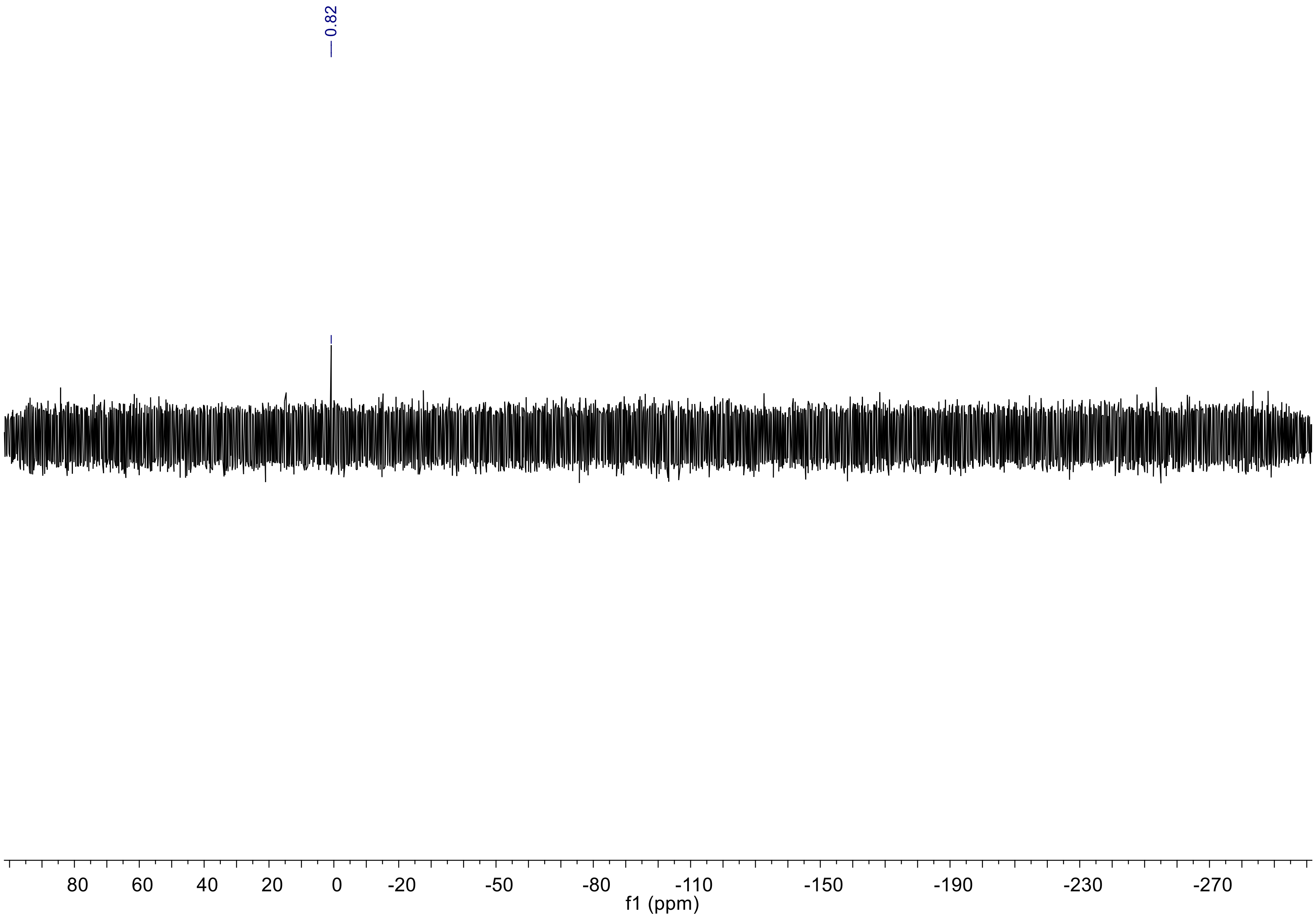

S61 ^{1}H NMR (400 MHz, $CDCl_3$, 298 K) spectrum of *(E)-4,4,5,5-tetramethyl-2-(3-((3-phenylprop-2-yn-1-yl)oxy)prop-1-en-1-yl)-1,3,2-dioxaborolane* **1l**.

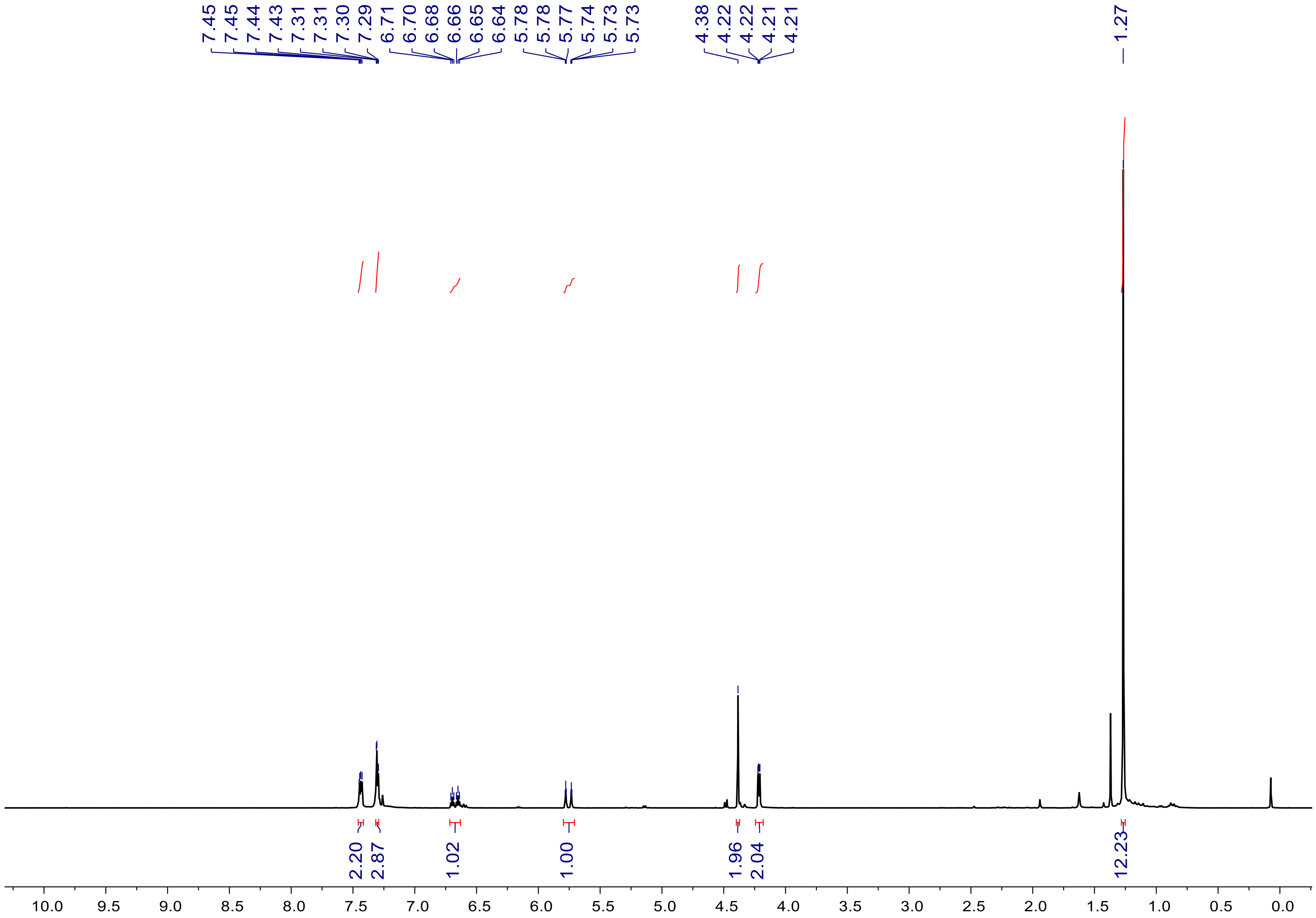

S62 ^{11}B NMR (128 MHz, $CDCl_3$, 298 K) spectrum of *(E)-4,4,5,5-tetramethyl-2-(3-((3-phenylprop-2-yn-1-yl)oxy)prop-1-en-1-yl)-1,3,2-dioxaborolane* **1l**.

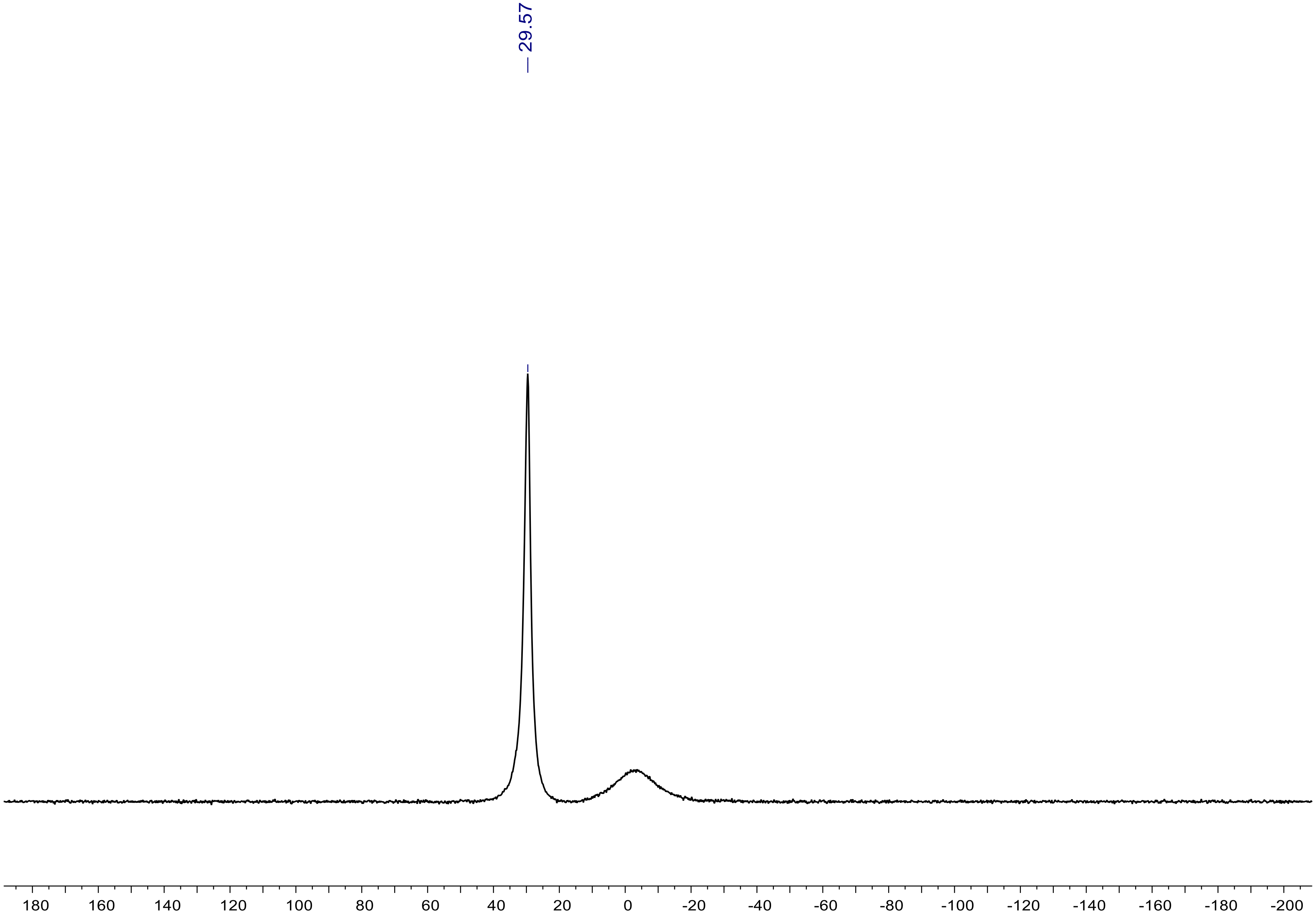

S63 ^{13}C NMR (101 MHz, $CDCl_3$, 298 K) spectrum of *(E)-4,4,5,5-tetramethyl-2-(3-((3-phenylprop-2-yn-1-yl)oxy)prop-1-en-1-yl)-1,3,2-dioxaborolane* **1l**.

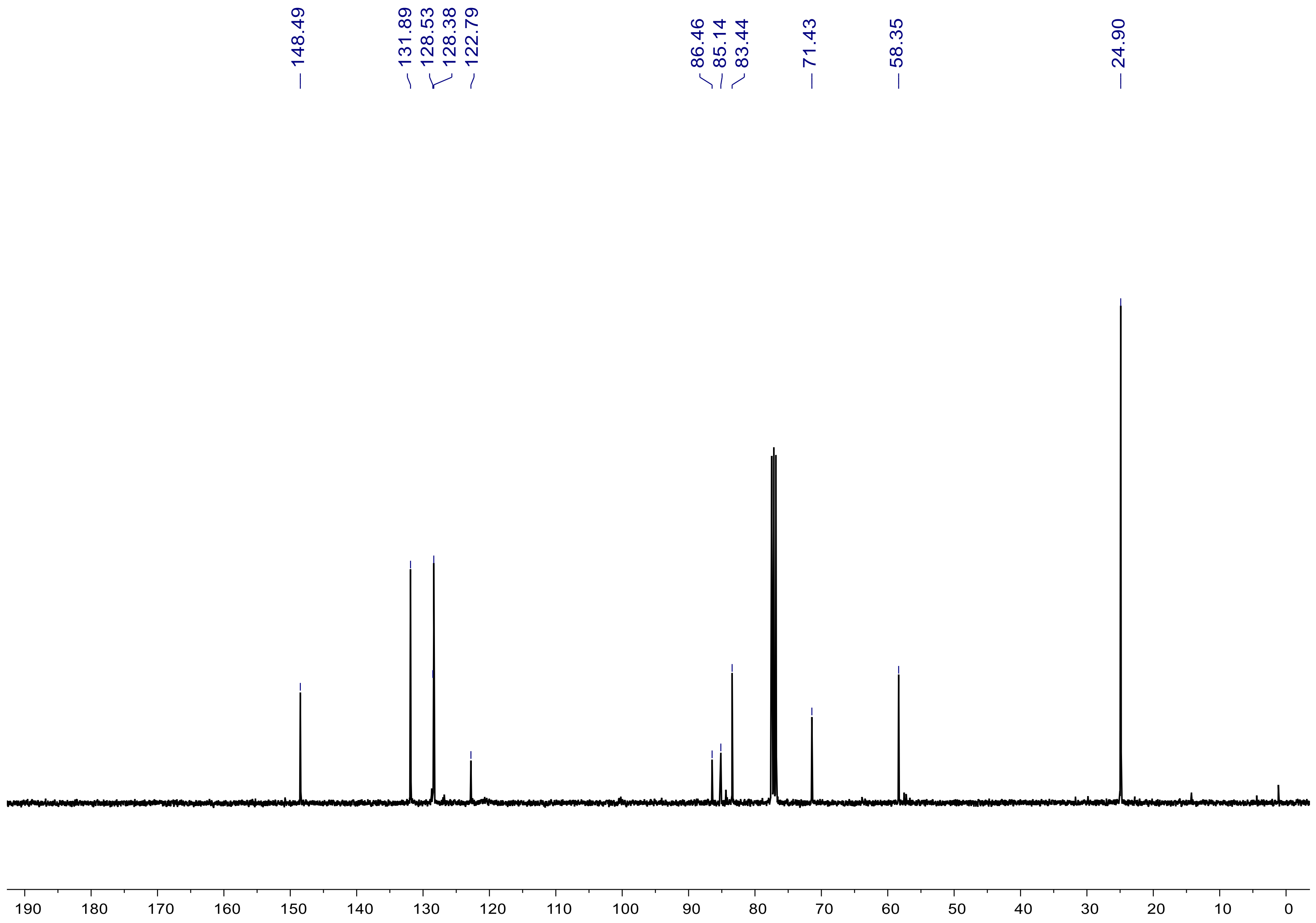

S64 ^{1}H NMR (500 MHz, $CDCl_3$, 298 K) spectrum of *(Z)-4,4,5,5-tetramethyl-2-(1-phenylprop-1-en-2-yl)-1,3,2-dioxaborolane* **1m**.

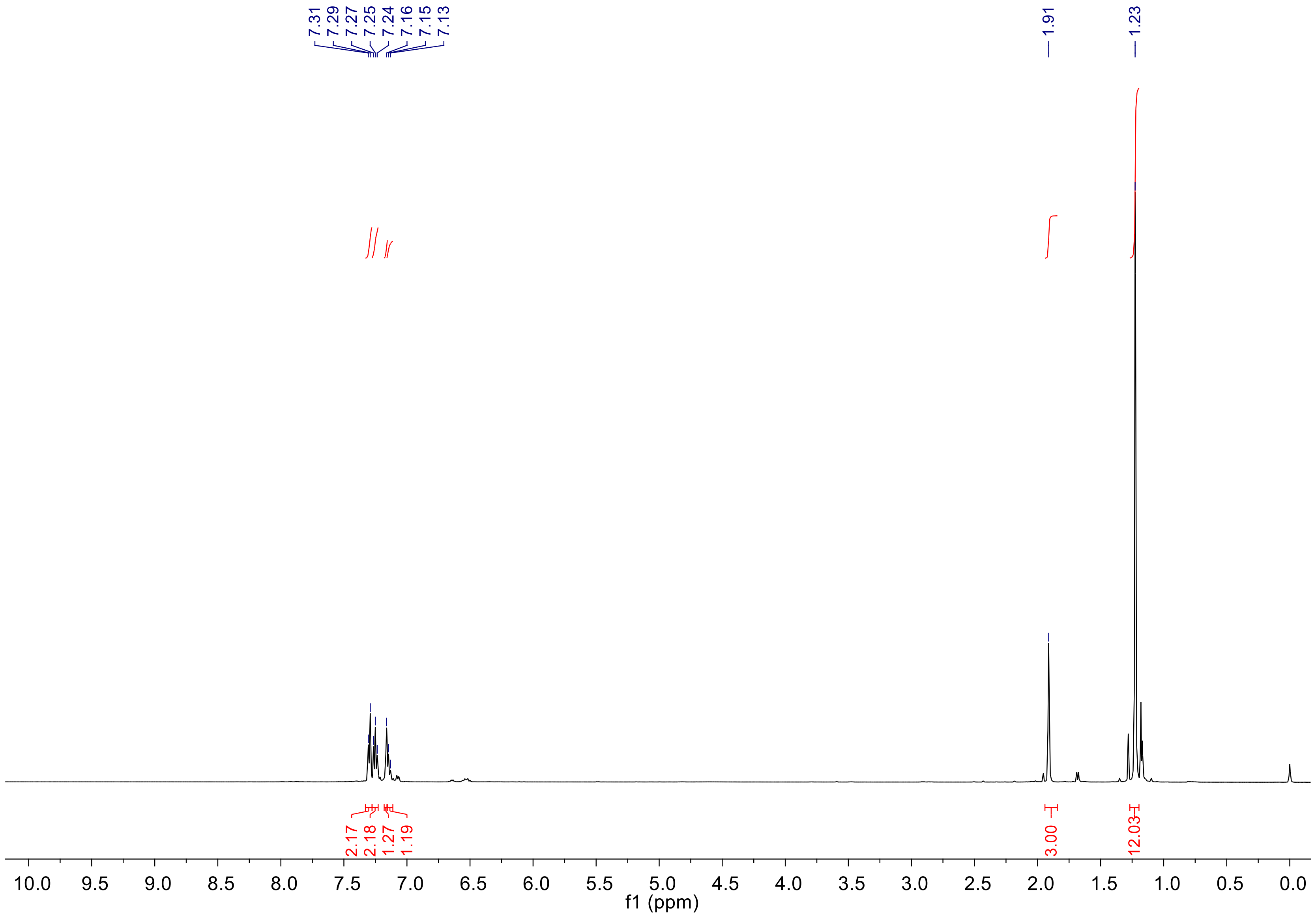

S65 ^{11}B NMR (160 MHz, $CDCl_3$, 298 K) spectrum of *(Z)-4,4,5,5-tetramethyl-2-(1-phenylprop-1-en-2-yl)-1,3,2-dioxaborolane* **1m**.

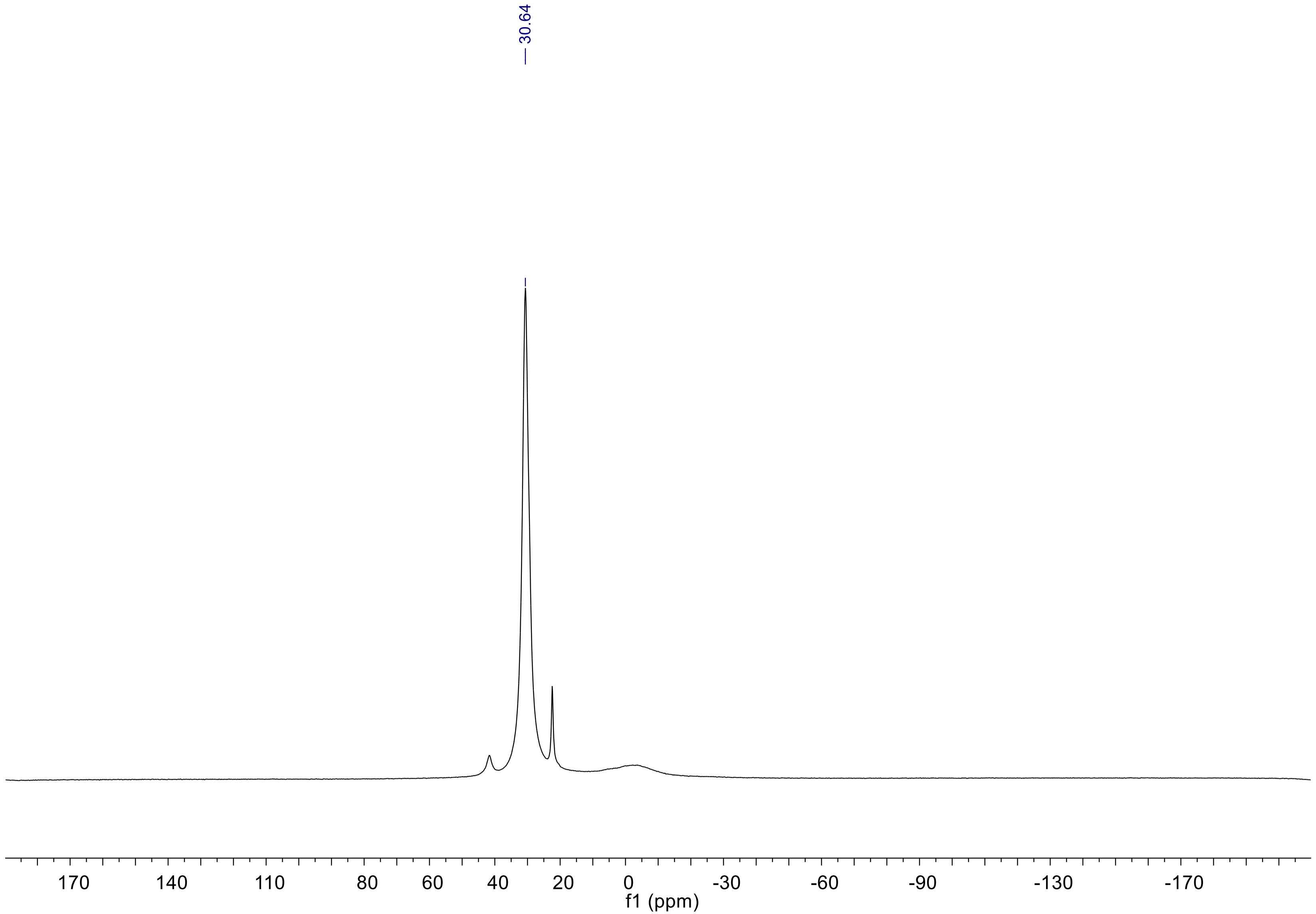

S66 ^{13}C NMR (126 MHz, $CDCl_3$, 298 K) spectrum of *(Z)-4,4,5,5-tetramethyl-2-(1-phenylprop-1-en-2-yl)-1,3,2-dioxaborolane* **1m**.

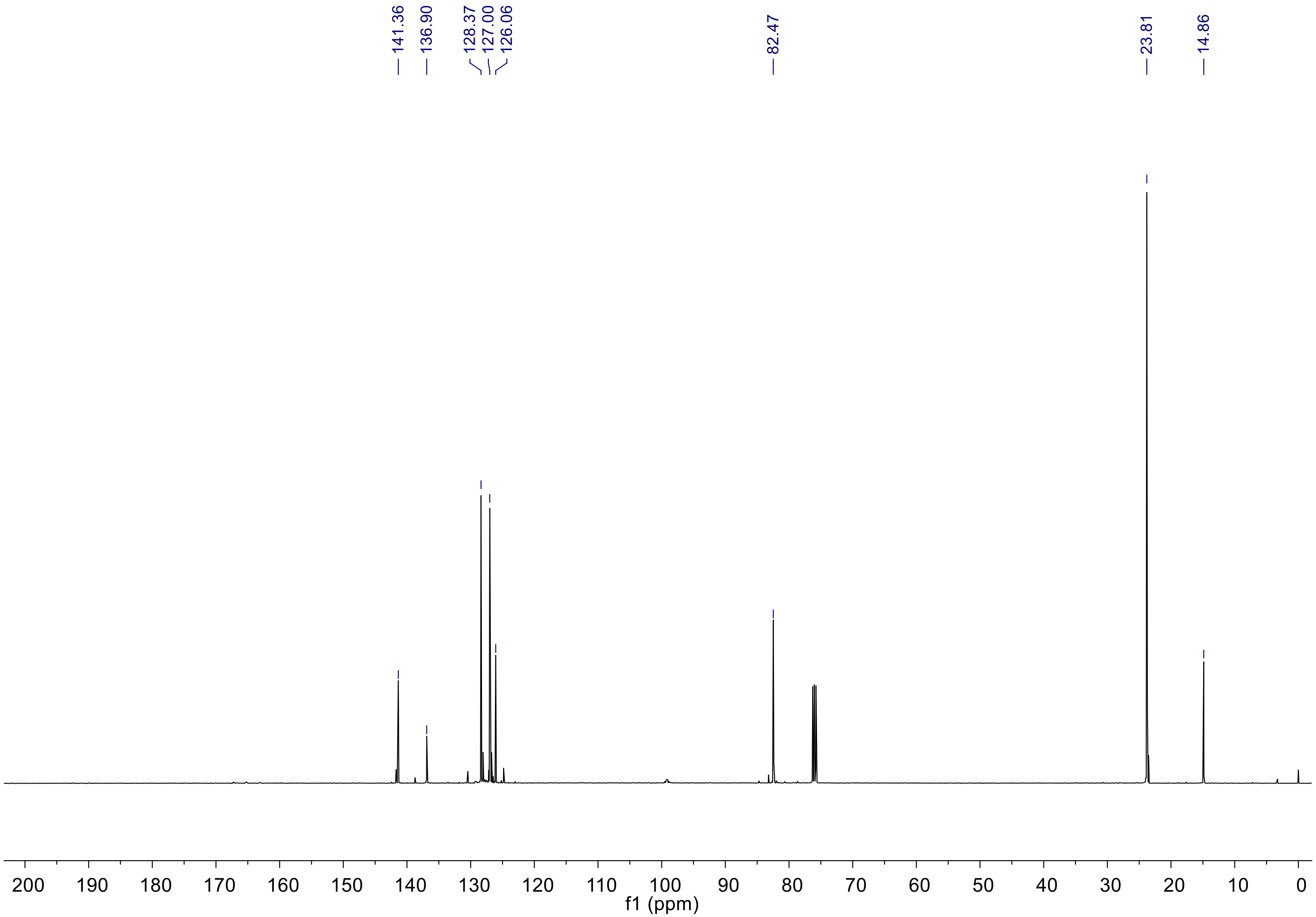

S67 ^{1}H NMR (400 MHz, $CDCl_3$, 298 K) spectrum of *(Z)-2-(but-2-en-2-yl)-4,4,5,5-tetramethyl-1,3,2-dioxaborolane* **1n**.

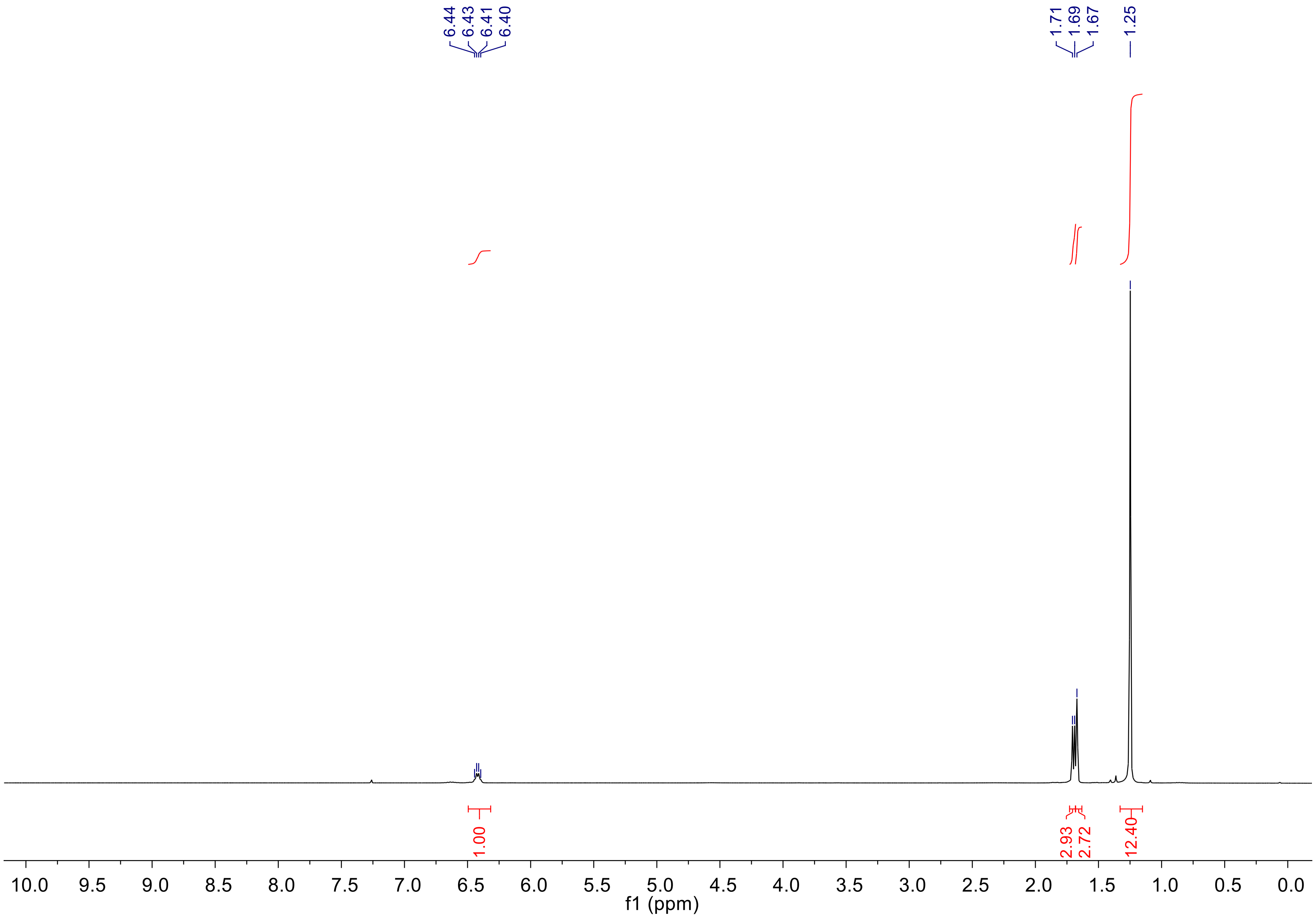

S68 ^{11}B NMR (128 MHz, $CDCl_3$, 298 K) spectrum of *(Z)-2-(but-2-en-2-yl)-4,4,5,5-tetramethyl-1,3,2-dioxaborolane* **1n**.

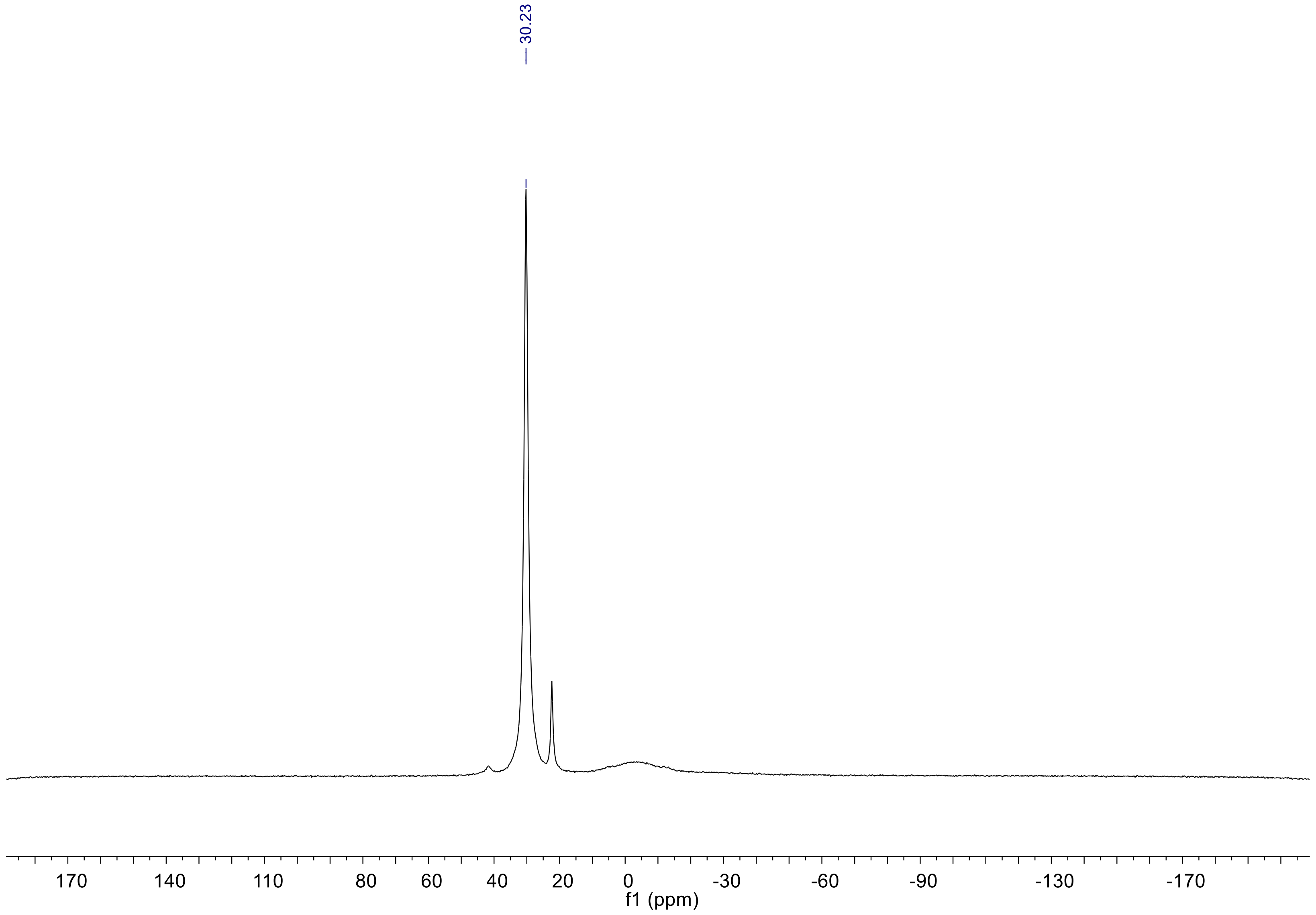

S69 ^{13}C NMR (101 MHz, $CDCl_3$, 298 K) spectrum of *(Z)-2-(but-2-en-2-yl)-4,4,5,5-tetramethyl-1,3,2-dioxaborolane* **1n**.

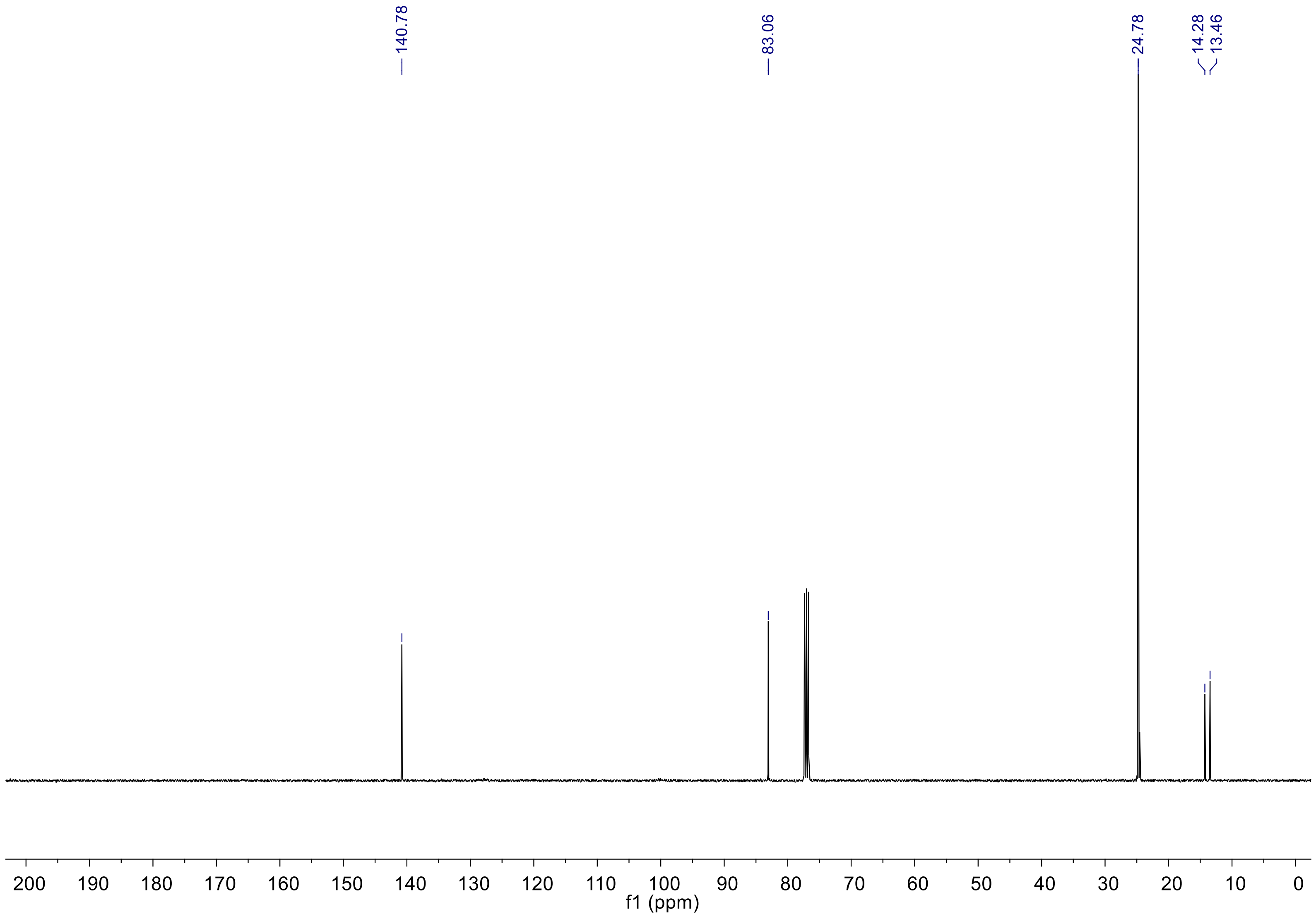

S70 ^{1}H NMR (400 MHz, $CDCl_3$, 298 K) spectrum of *(Z)-trimethyl(2-(4,4,5,5-tetramethyl-1,3,2-dioxaborolan-2-yl)prop-1-en-1-yl)silane* **1o**.

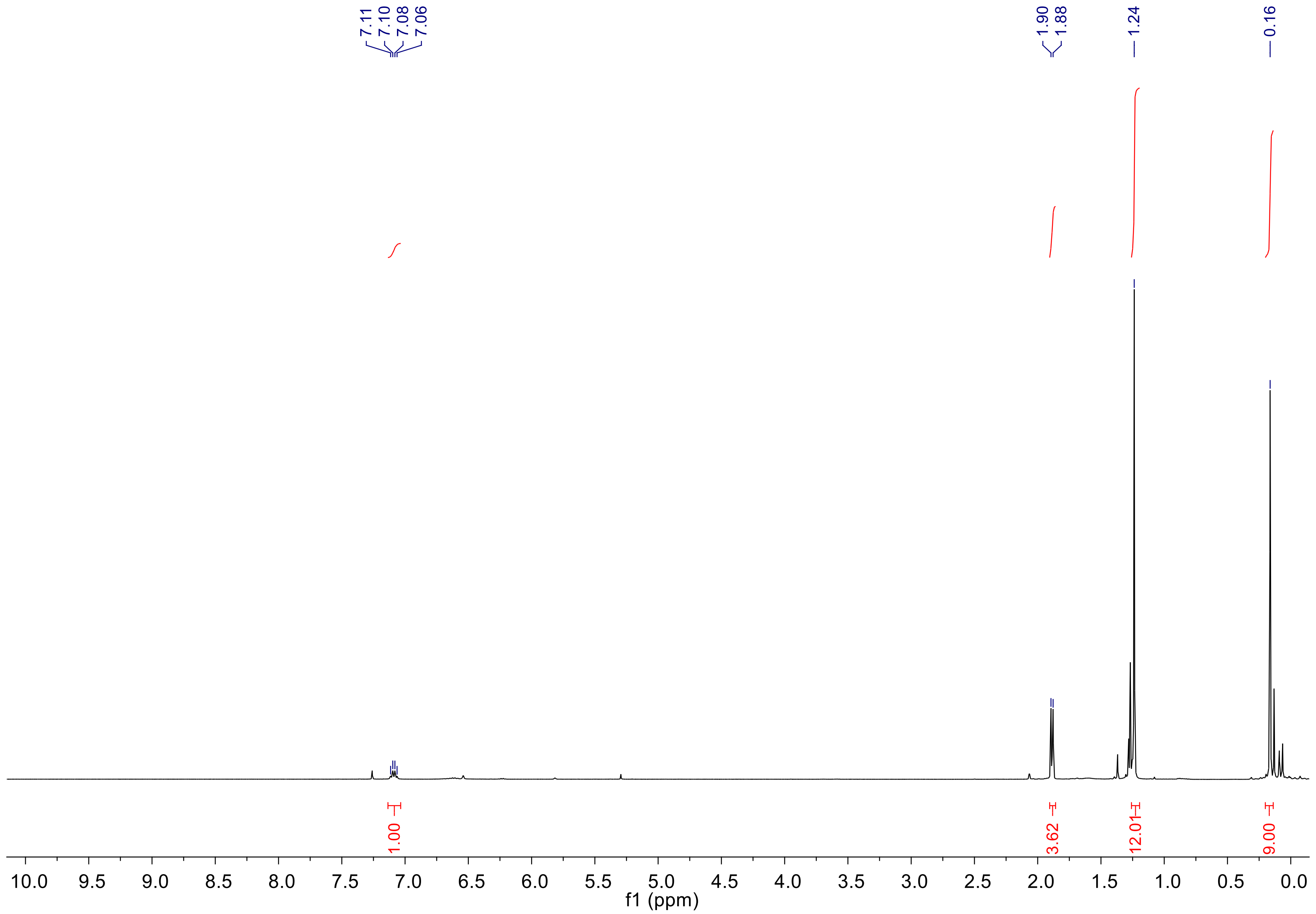

S71 ^{11}B NMR (128 MHz, $CDCl_3$, 298 K) spectrum of *(Z)-trimethyl(2-(4,4,5,5-tetramethyl-1,3,2-dioxaborolan-2-yl)prop-1-en-1-yl)silane* **1o**.

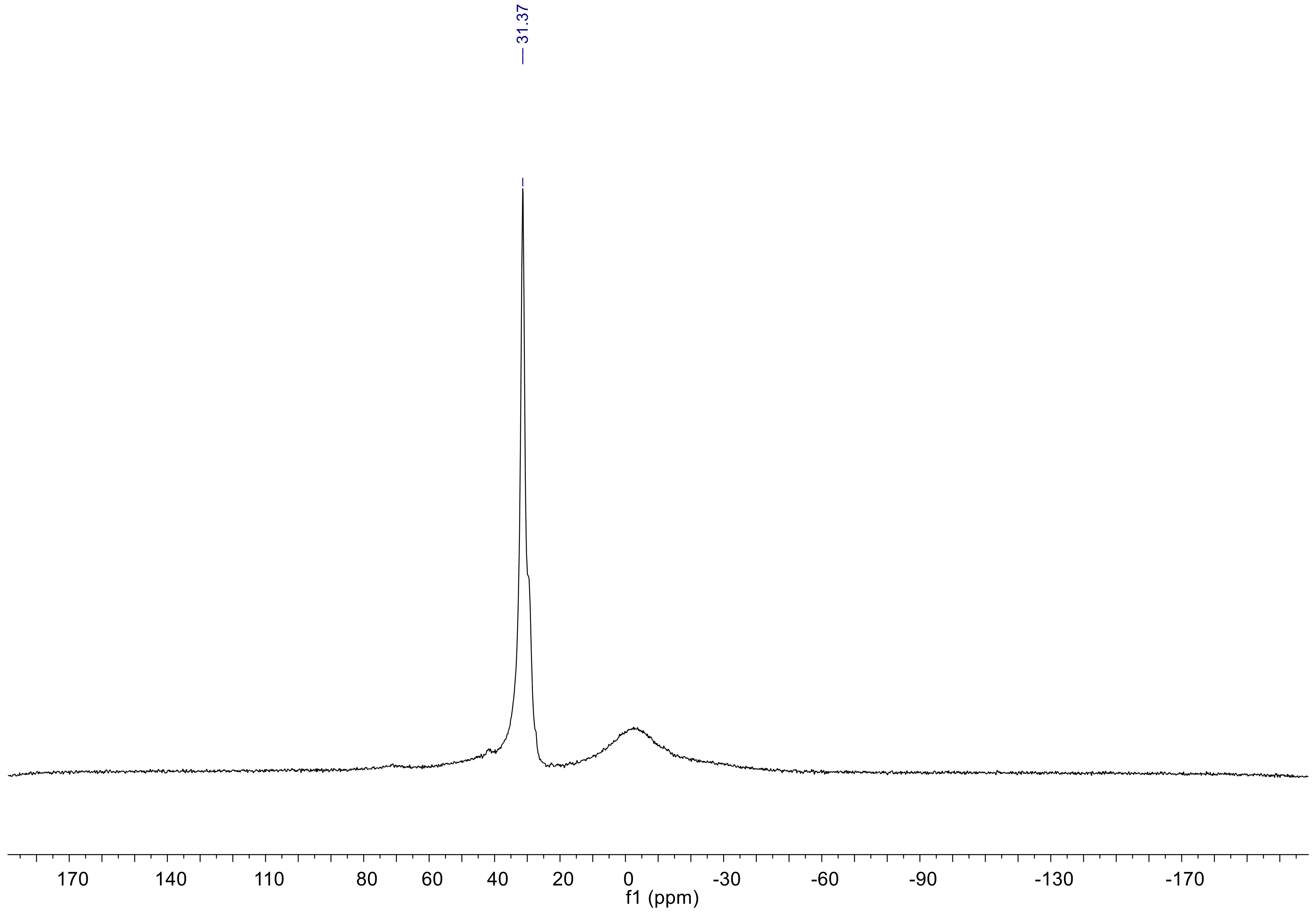

S72 ^{13}C NMR (101 MHz, $CDCl_3$, 298 K) spectrum of *(Z)-trimethyl(2-(4,4,5,5-tetramethyl-1,3,2-dioxaborolan-2-yl)prop-1-en-1-yl)silane* **1o**.

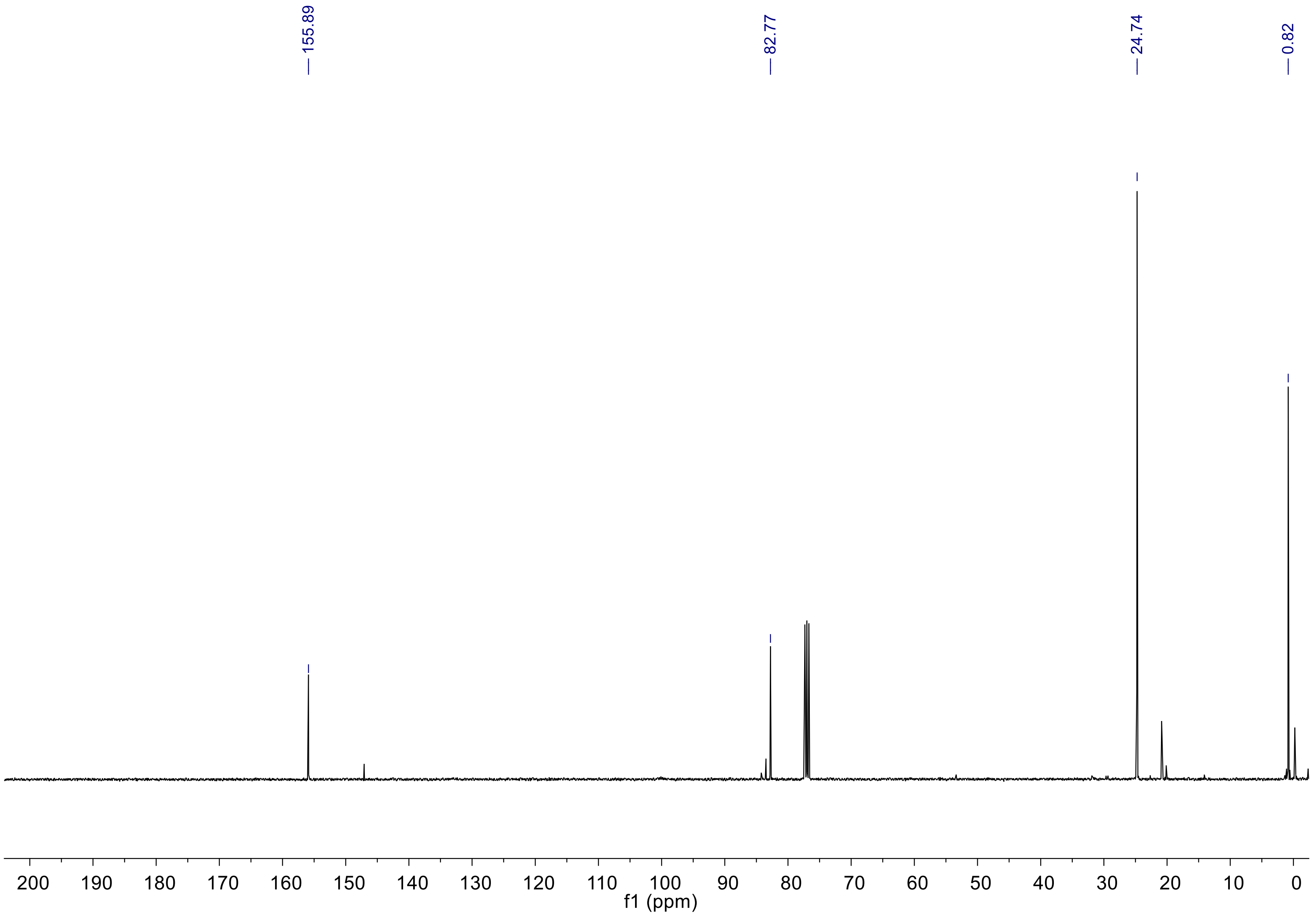

S73 ^{29}Si NMR (80 MHz, $CDCl_3$, 298 K) spectrum of *(Z)-trimethyl(2-(4,4,5,5-tetramethyl-1,3,2-dioxaborolan-2-yl)prop-1-en-1-yl)silane* **1o**.

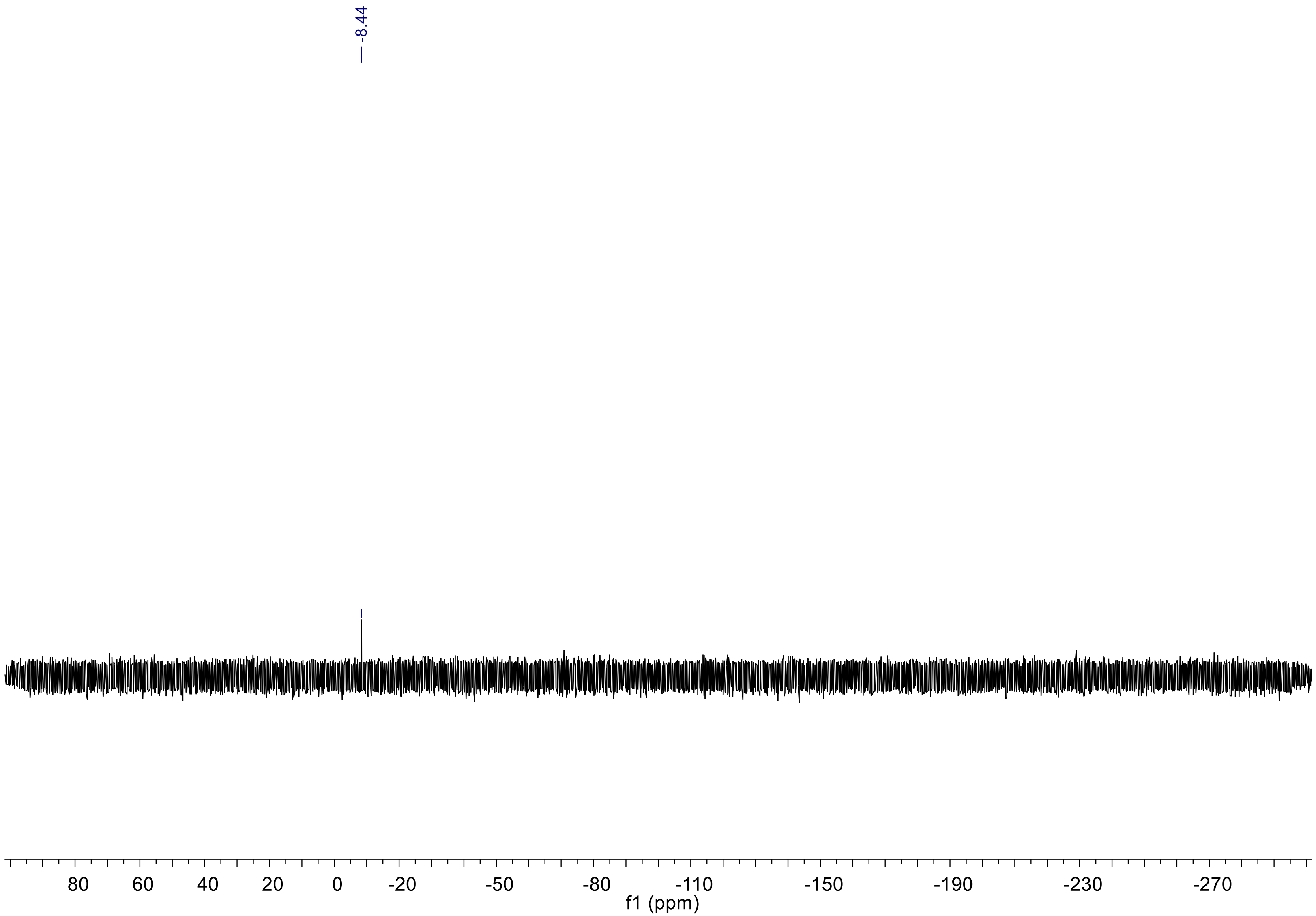

S74 ^{1}H NMR (400 MHz, $CDCl_3$, 298 K) spectrum of *(Z)-2-(1,2-diphenylvinyl)-4,4,5,5-tetramethyl-1,3,2-dioxaborolane* **1p**.

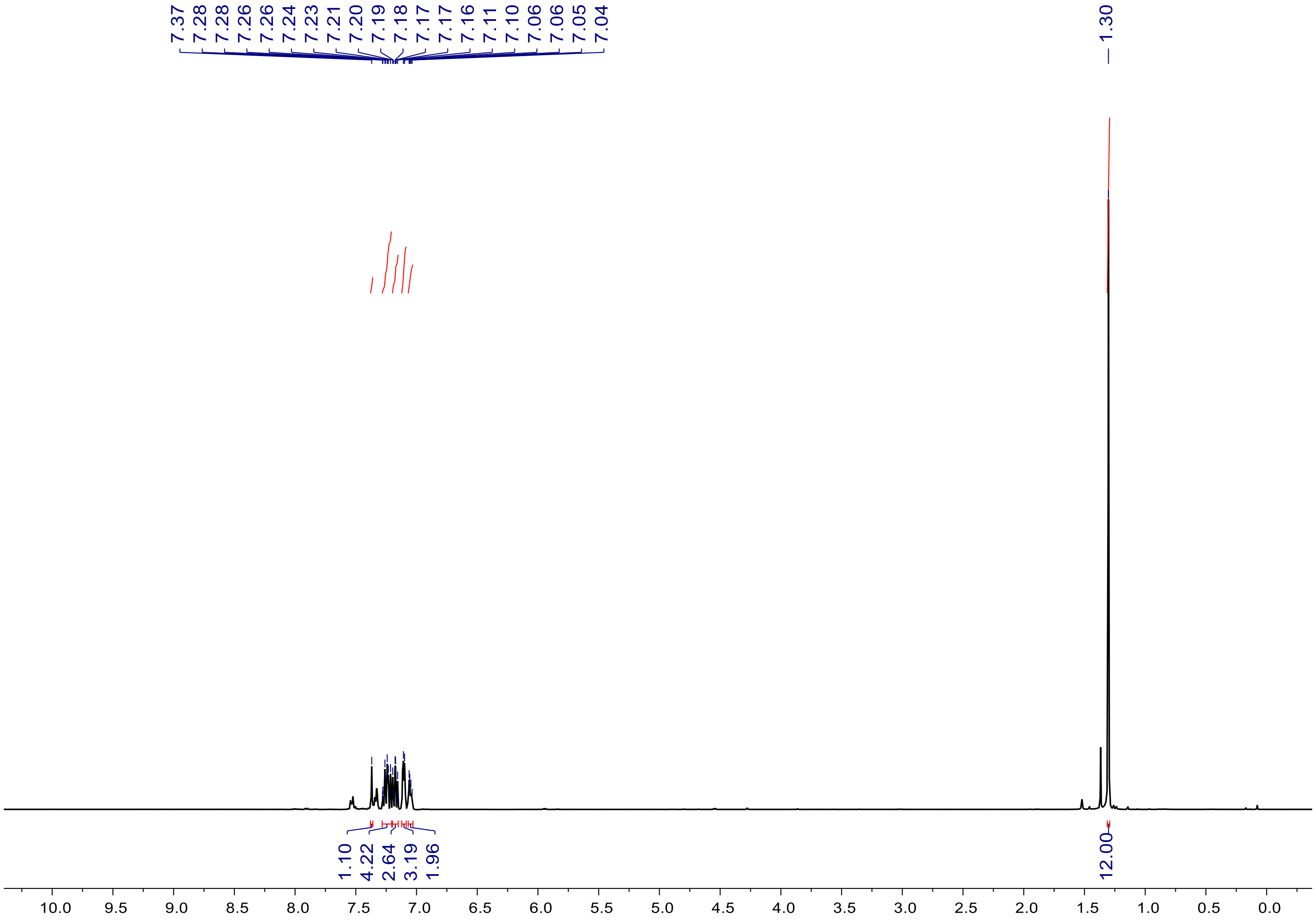

S75 ^{11}B NMR (128 MHz, $CDCl_3$, 298 K) spectrum of *(Z)-2-(1,2-diphenylvinyl)-4,4,5,5-tetramethyl-1,3,2-dioxaborolane* **1p**.

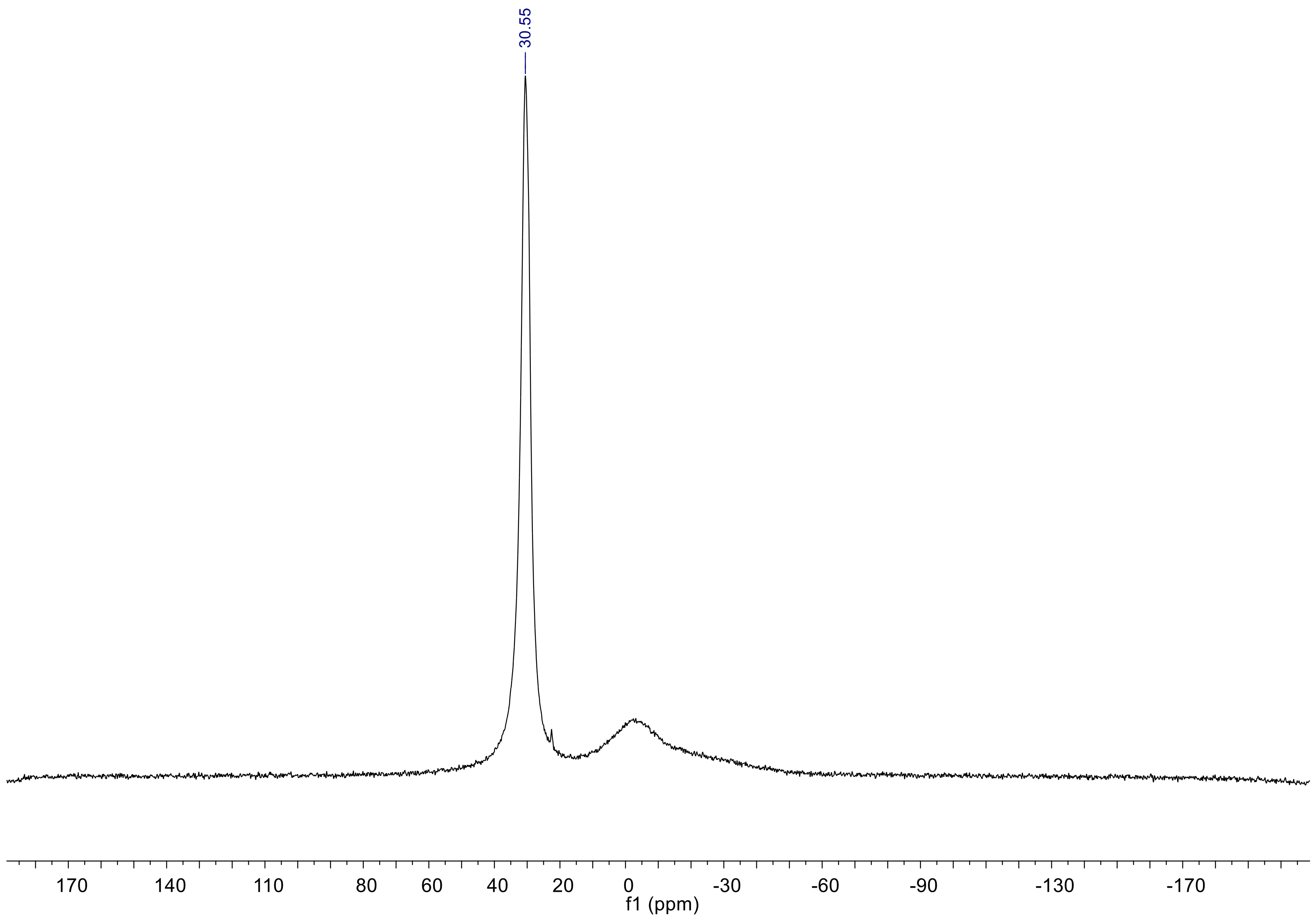

S76 ^{13}C NMR (101 MHz, $CDCl_3$, 298 K) spectrum of *(Z)-2-(1,2-diphenylvinyl)-4,4,5,5-tetramethyl-1,3,2-dioxaborolane* **1p**.

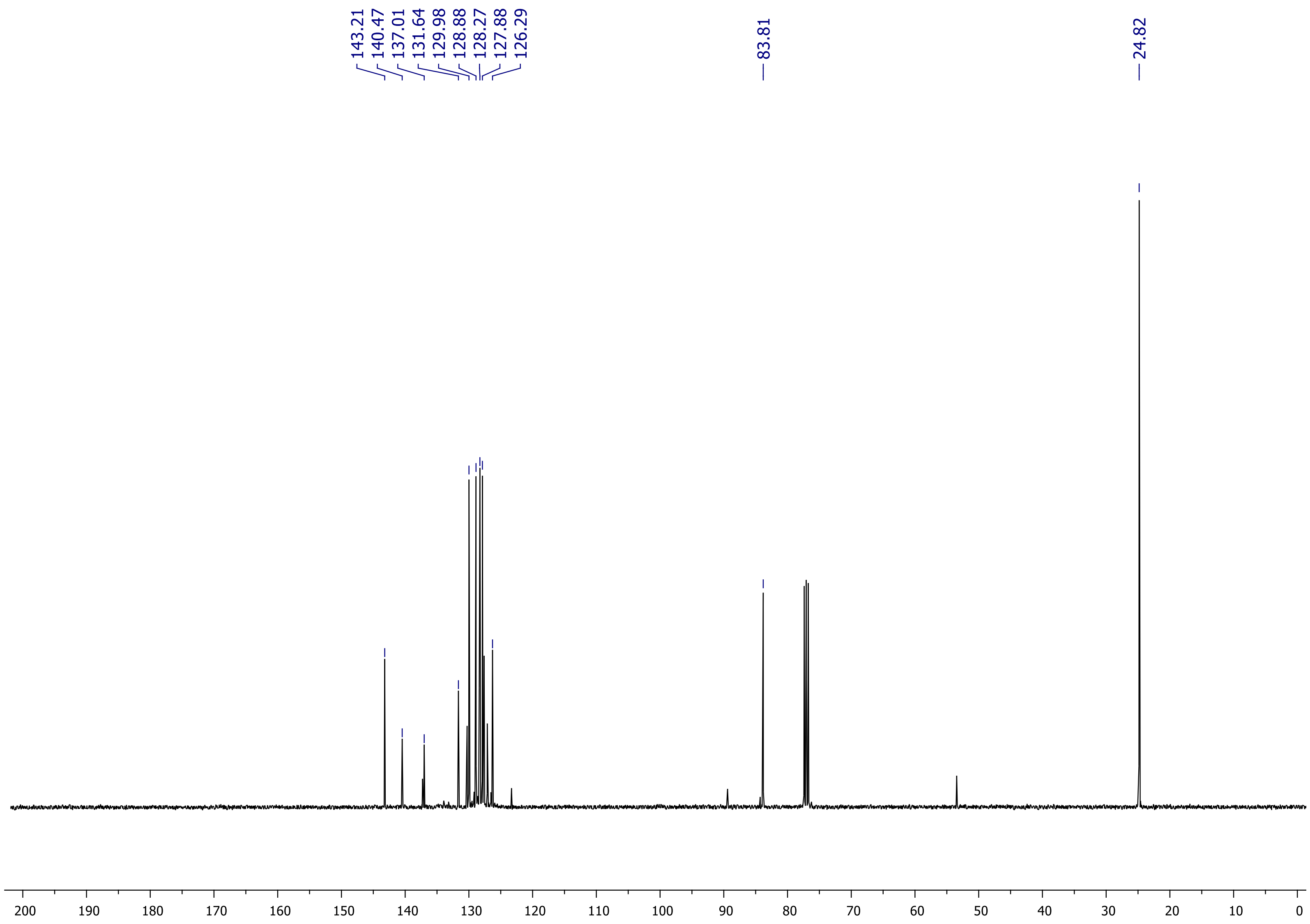

S77 ^{1}H NMR (500 MHz, $CDCl_3$, 298 K) spectrum of *2-(benzyloxy)-4,4,5,5-tetramethyl-1,3,2-dioxaborolane* **2a**.

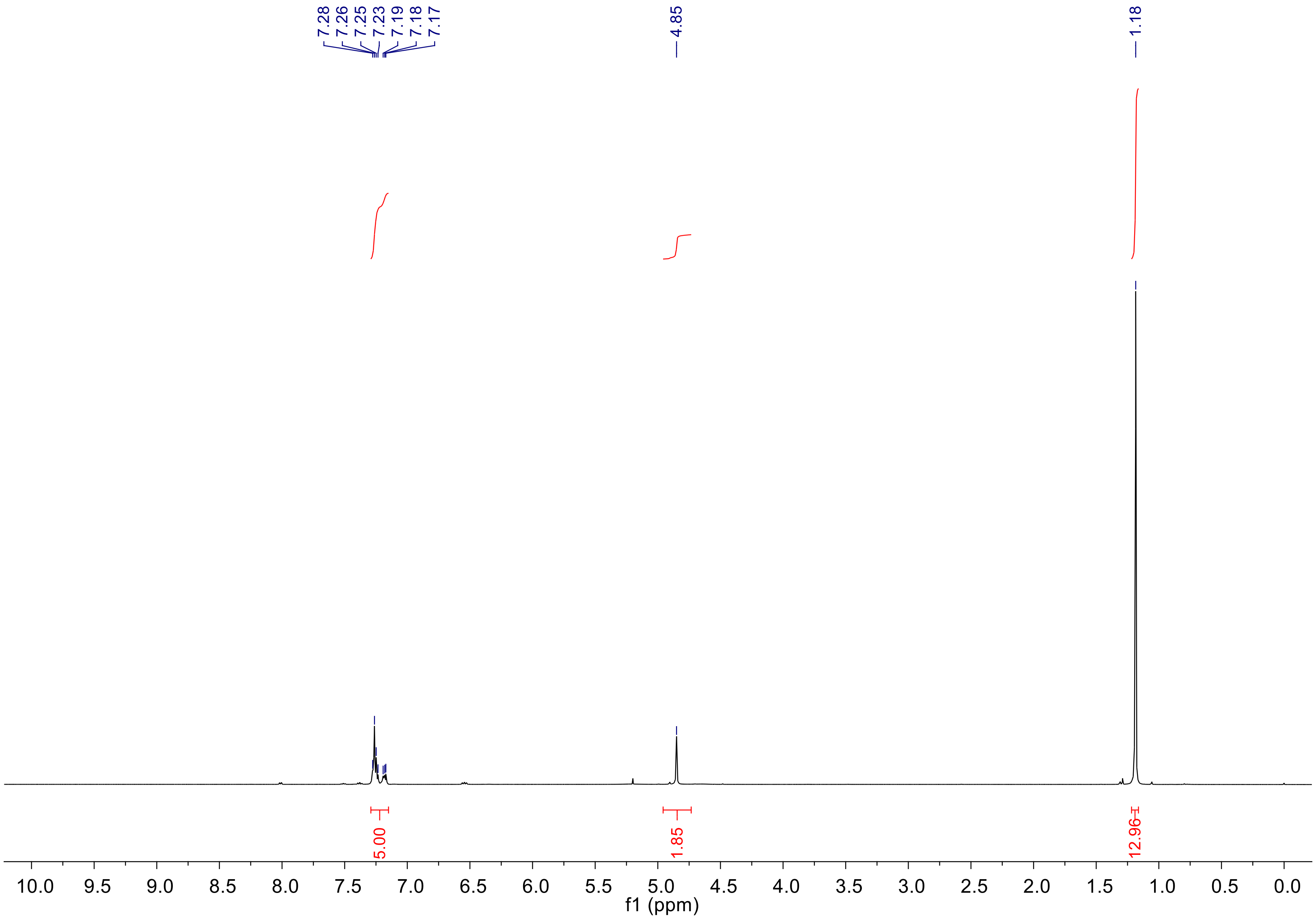

S78 ^{11}B NMR (160 MHz, $CDCl_3$, 298 K) spectrum of *2-(benzyloxy)-4,4,5,5-tetramethyl-1,3,2-dioxaborolane* **2a**.

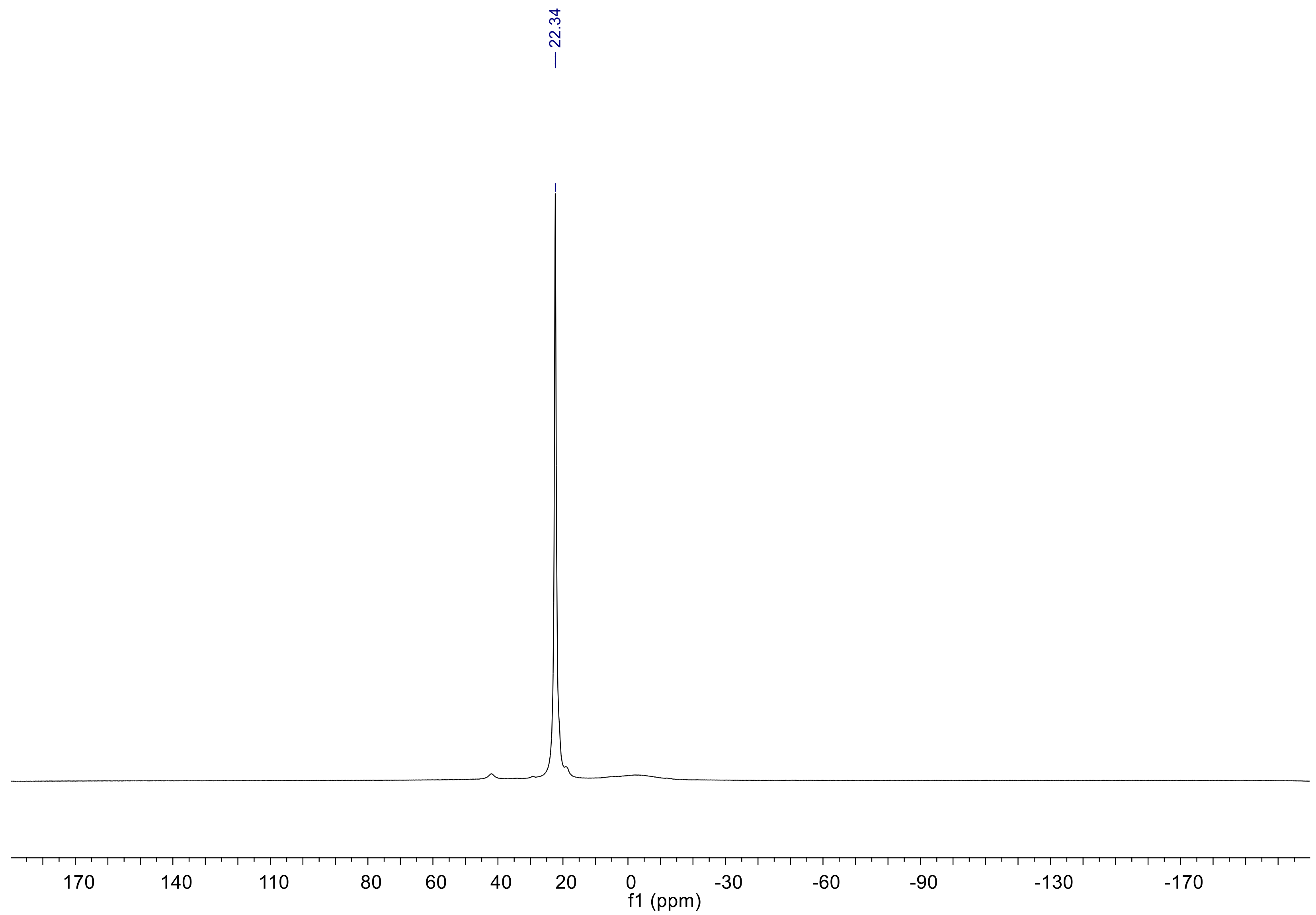

S79 ^{13}C NMR (126 MHz, $CDCl_3$, 298 K) spectrum of *2-(benzyloxy)-4,4,5,5-tetramethyl-1,3,2-dioxaborolane* **2a**.

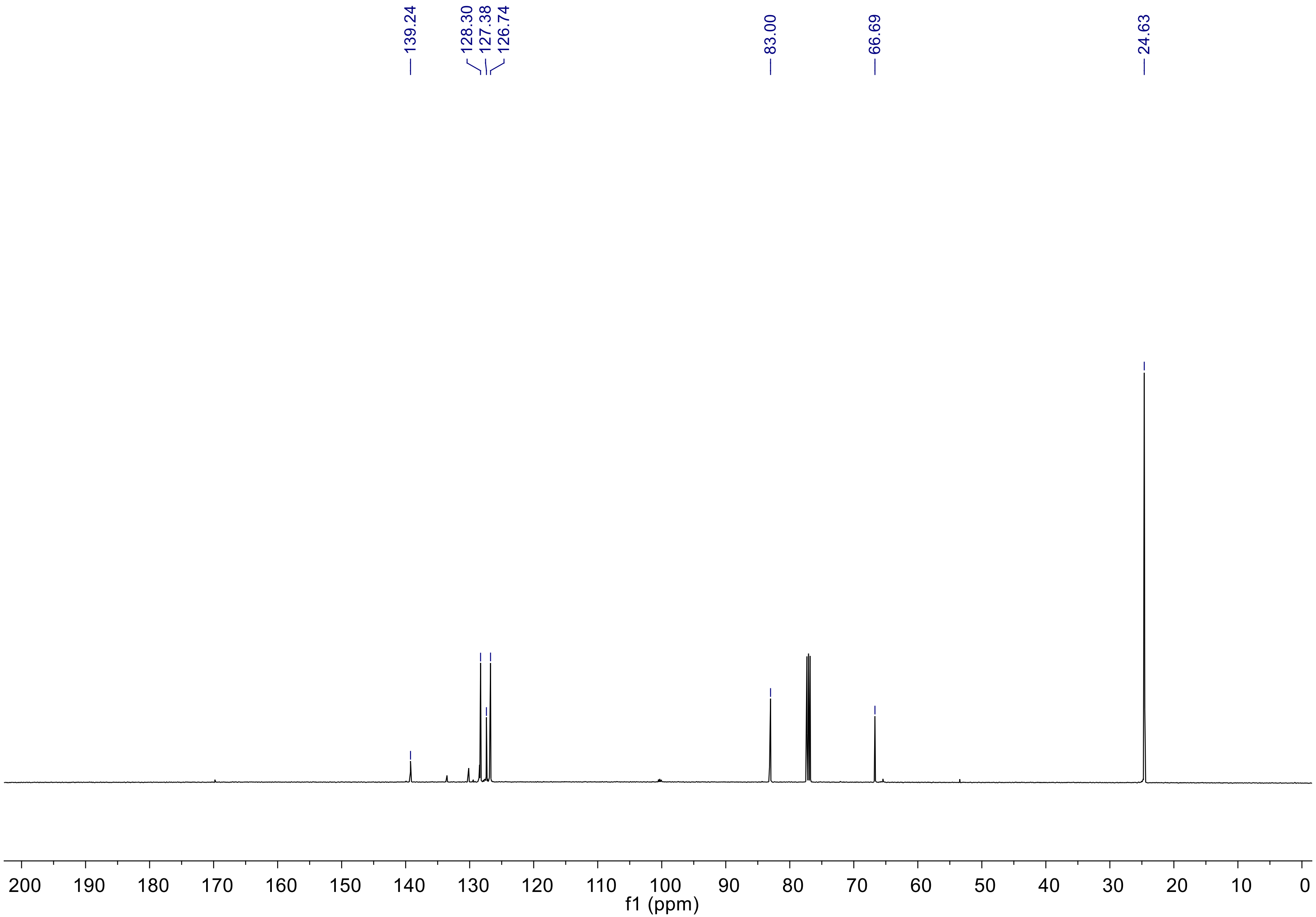

S80 ^{1}H NMR (500 MHz, $CDCl_3$, 298 K) spectrum of *4,4,5,5-tetramethyl-2-((4-methylbenzyl)oxy)-1,3,2-dioxaborolane* **2b**.

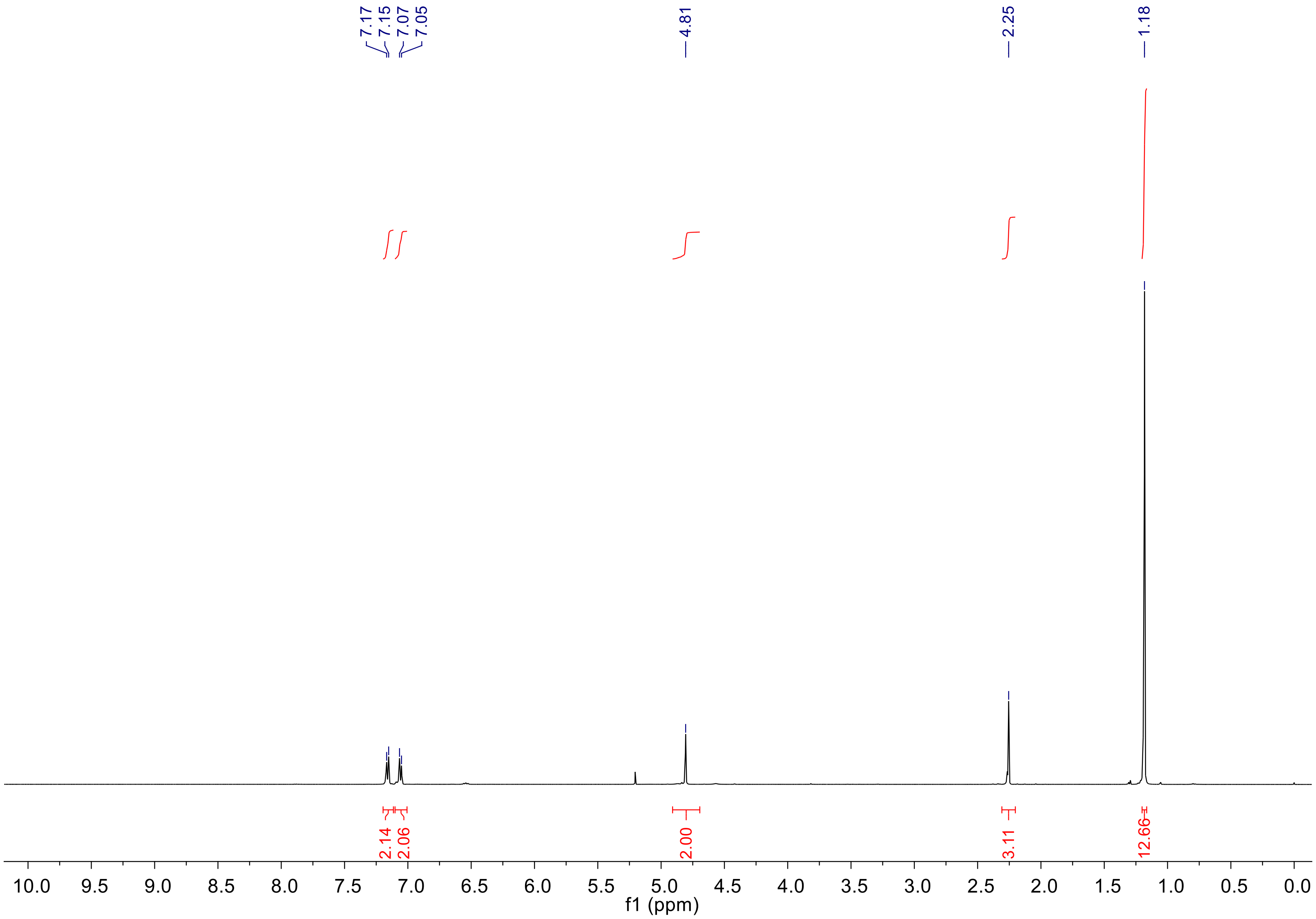

S81 ^{11}B NMR (160 MHz, $CDCl_3$, 298 K) spectrum of *4,4,5,5-tetramethyl-2-((4-methylbenzyl)oxy)-1,3,2-dioxaborolane* **2b**.

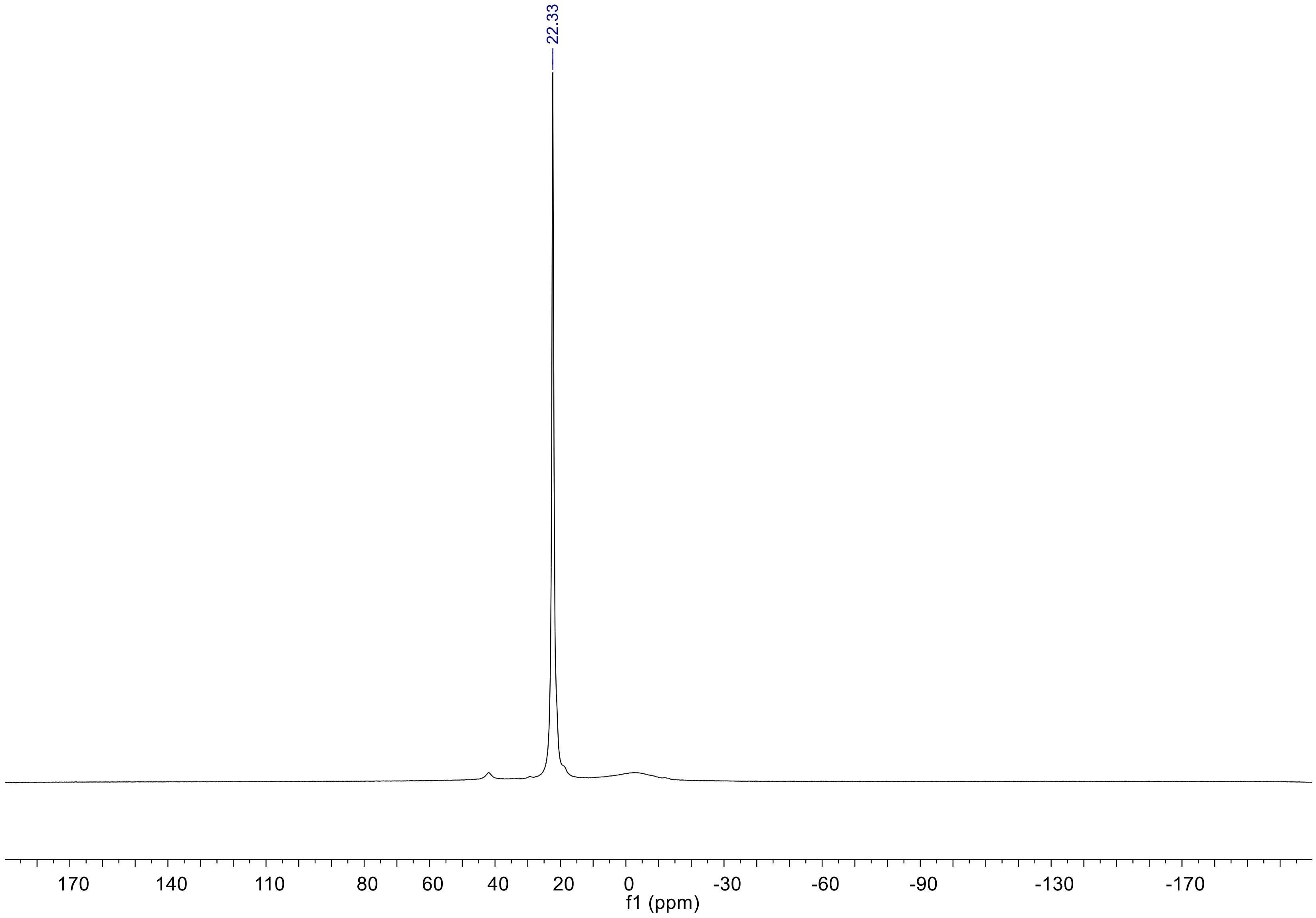

S82 ^{13}C NMR (126 MHz, $CDCl_3$, 298 K) spectrum of *4,4,5,5-tetramethyl-2-((4-methylbenzyl)oxy)-1,3,2-dioxaborolane* **2b**.

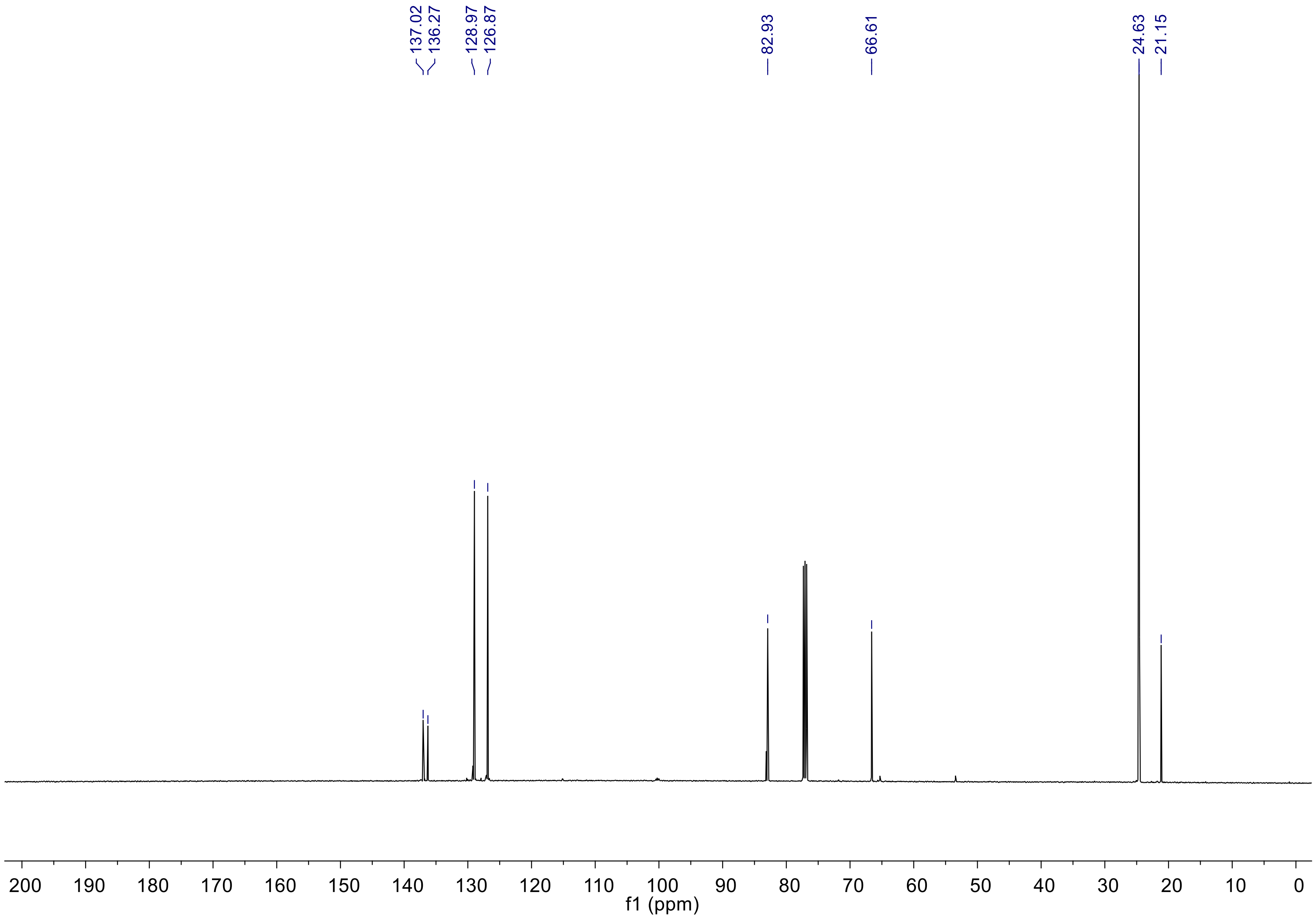

S83 ^{1}H NMR (500 MHz, $CDCl_3$, 298 K) spectrum of *4,4,5,5-tetramethyl-2-((2,4,6-trimethylbenzyl)oxy)-1,3,2-dioxaborolane* **2c**.

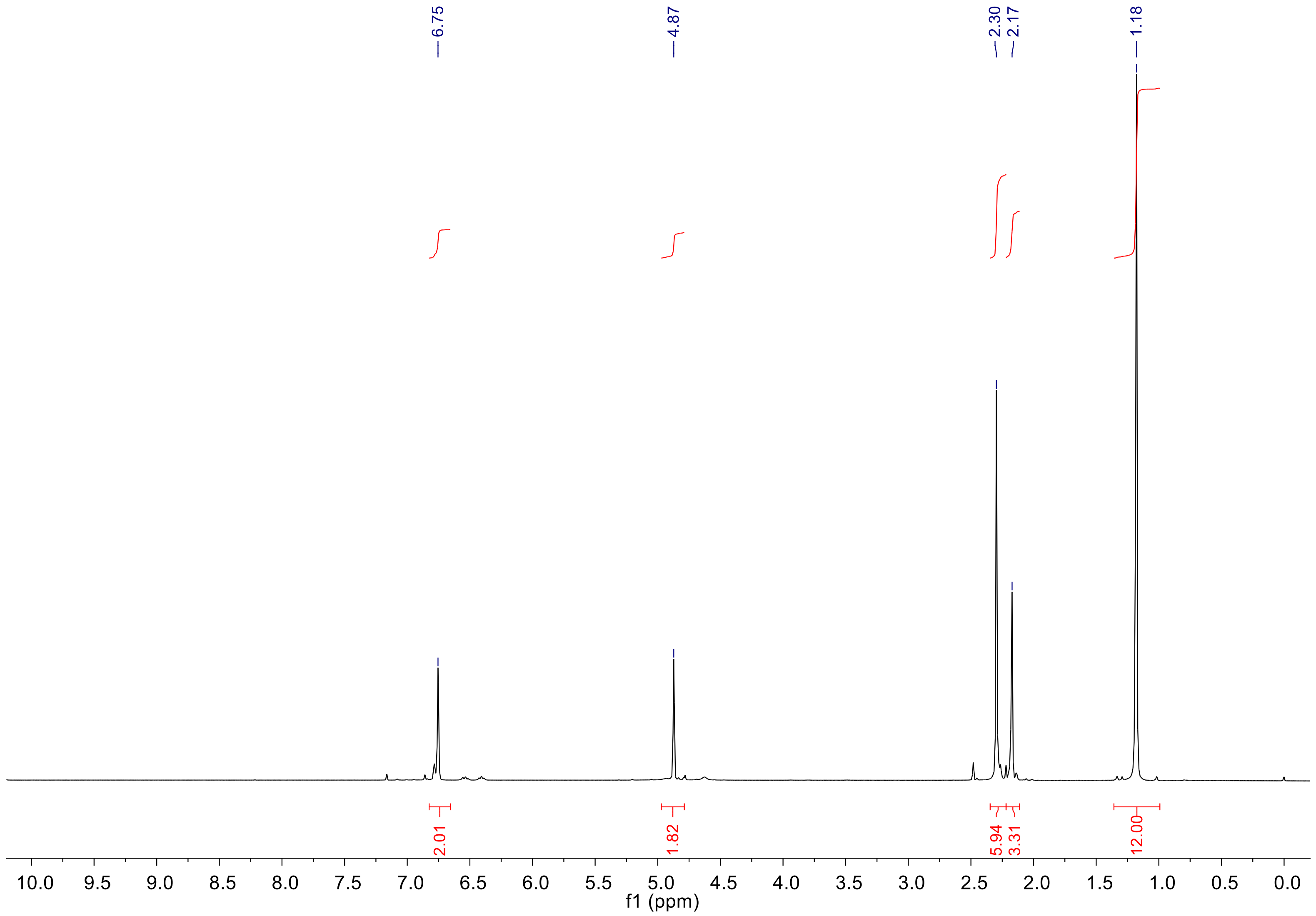

S84 ^{11}B NMR (160 MHz, $CDCl_3$, 298 K) spectrum of *4,4,5,5-tetramethyl-2-((2,4,6-trimethylbenzyl)oxy)-1,3,2-dioxaborolane* **2c**.

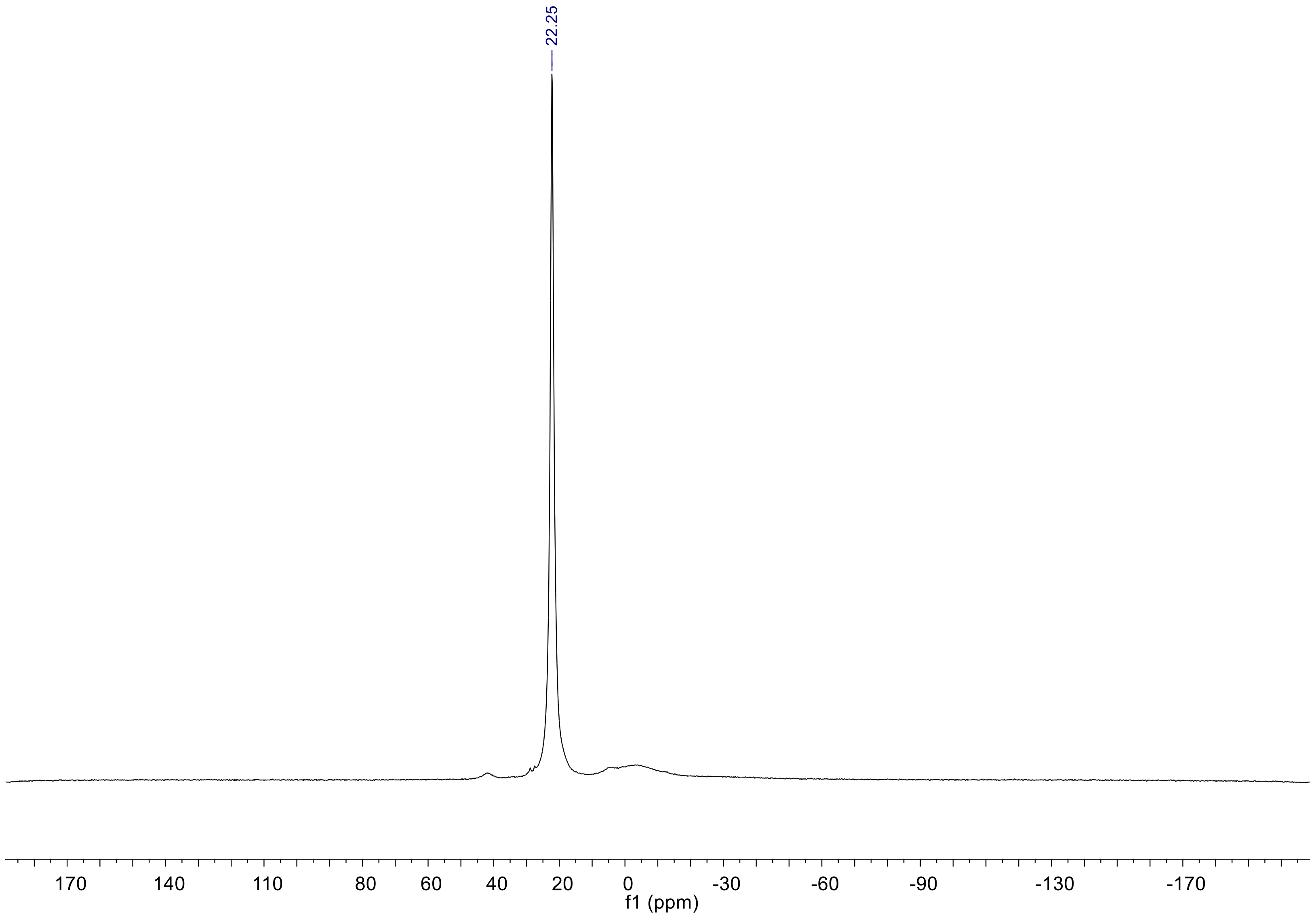

S85 ^{13}C NMR (126 MHz, $CDCl_3$, 298 K) spectrum of *4,4,5,5-tetramethyl-2-((2,4,6-trimethylbenzyl)oxy)-1,3,2-dioxaborolane* **2c**.

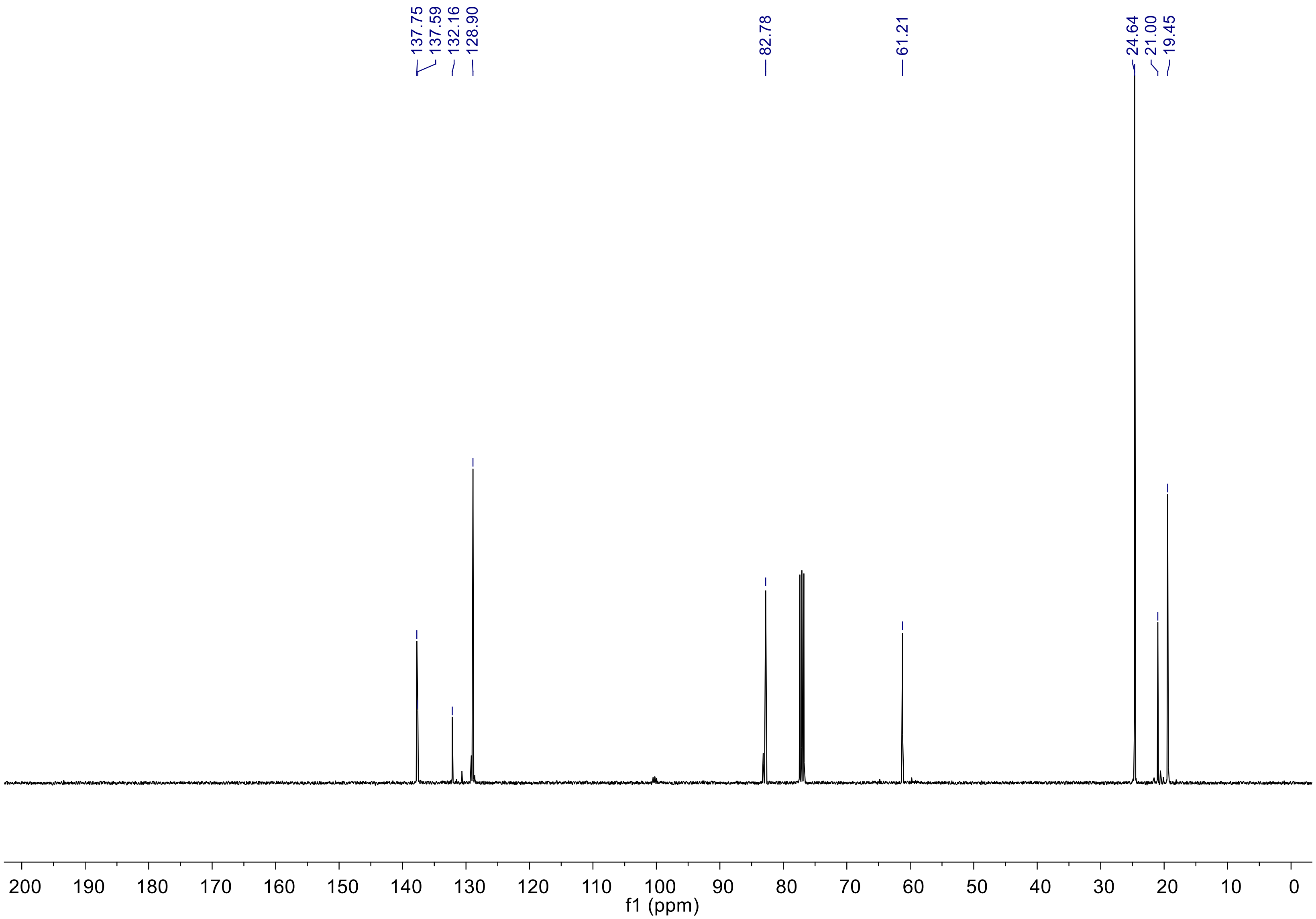

S86 ^{1}H NMR (500 MHz, $CDCl_3$, 298 K) spectrum of *2-((4-fluorobenzyl)oxy)-4,4,5,5-tetramethyl-1,3,2-dioxaborolane* **2d**.

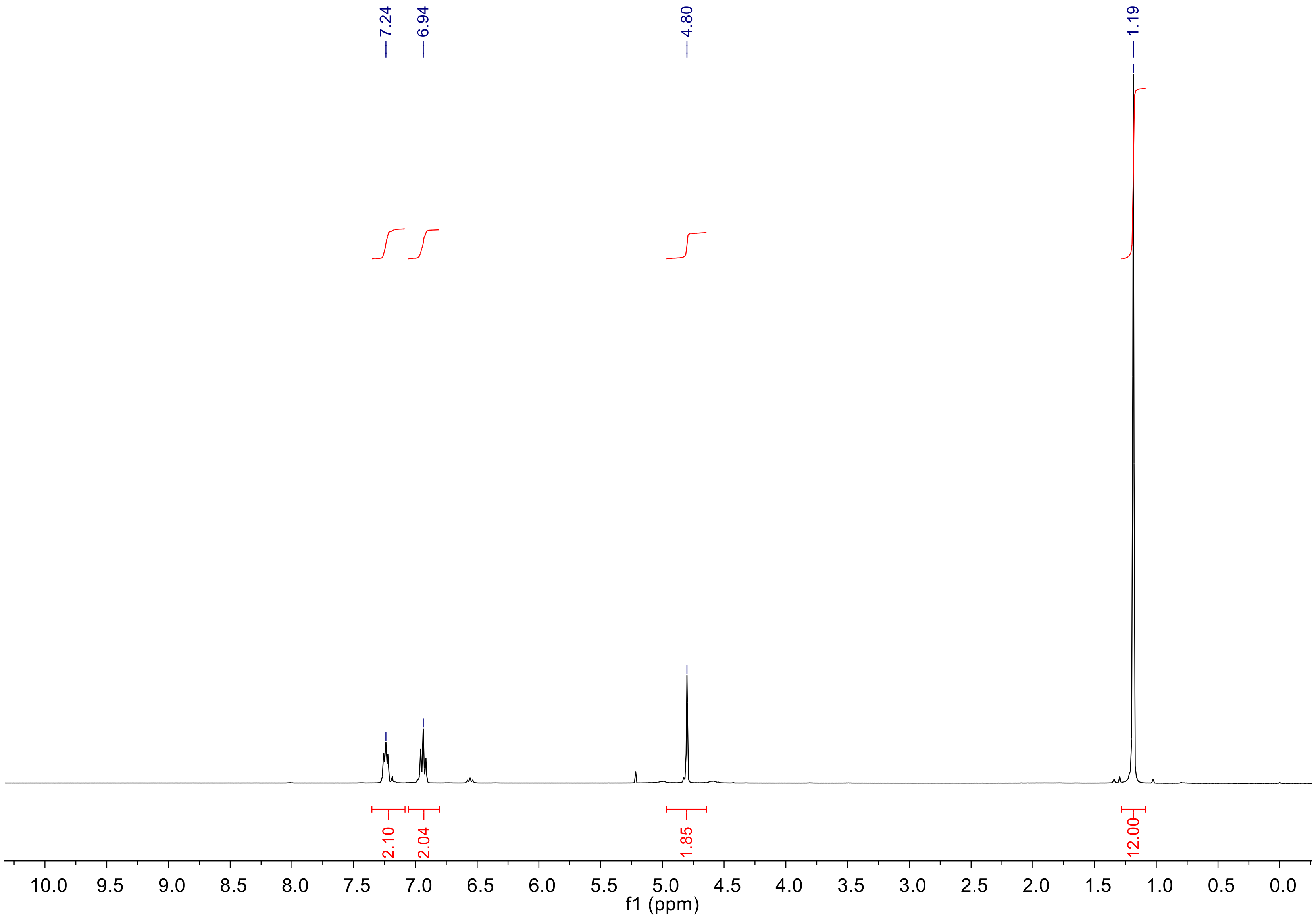

S87 ^{11}B NMR (160 MHz, $CDCl_3$, 298 K) spectrum of *2-((4-fluorobenzyl)oxy)-4,4,5,5-tetramethyl-1,3,2-dioxaborolane* **2d**.

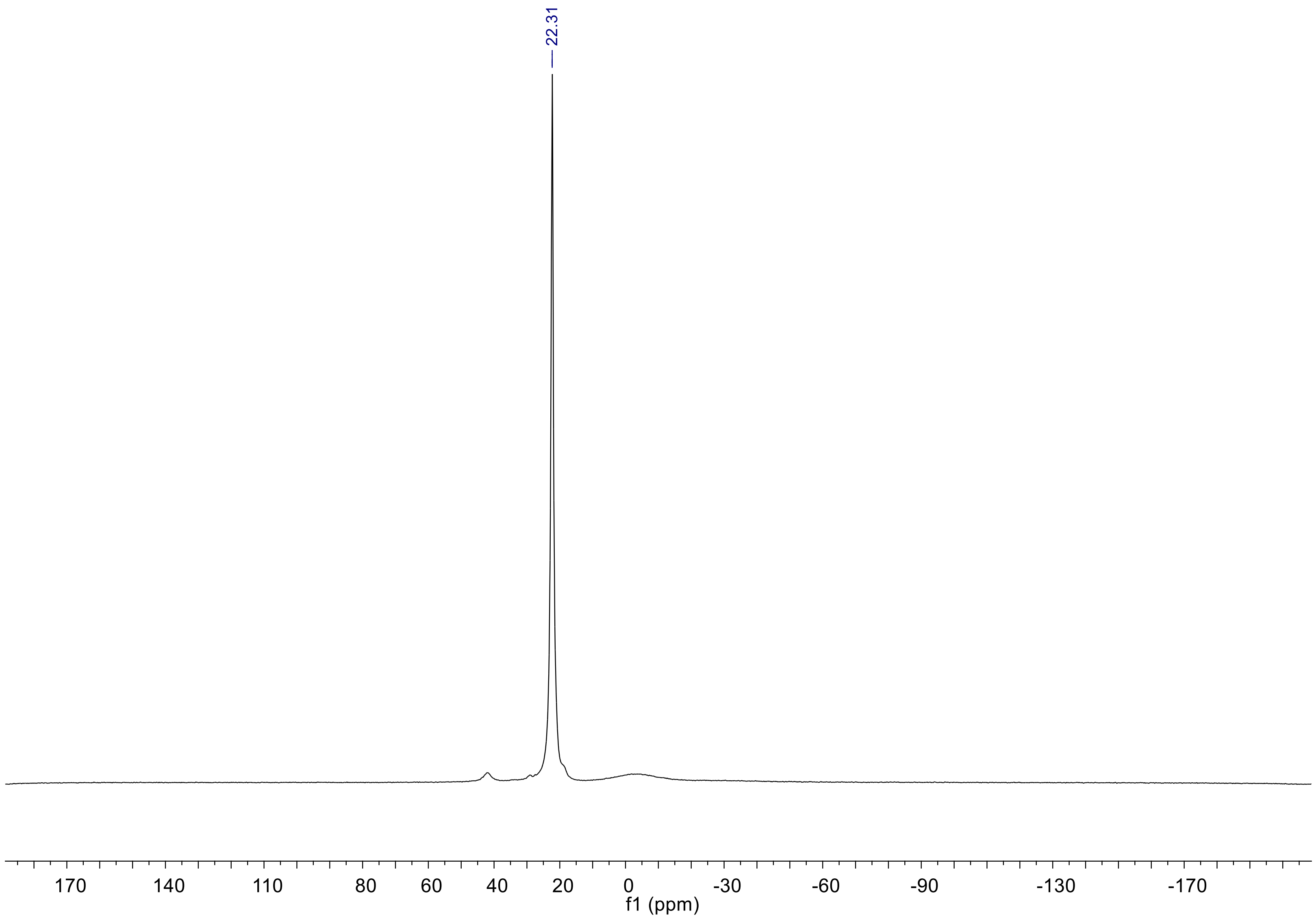

S88 ^{13}C NMR (126 MHz, $CDCl_3$, 298 K) spectrum of *2-((4-fluorobenzyl)oxy)-4,4,5,5-tetramethyl-1,3,2-dioxaborolane* **2d**.

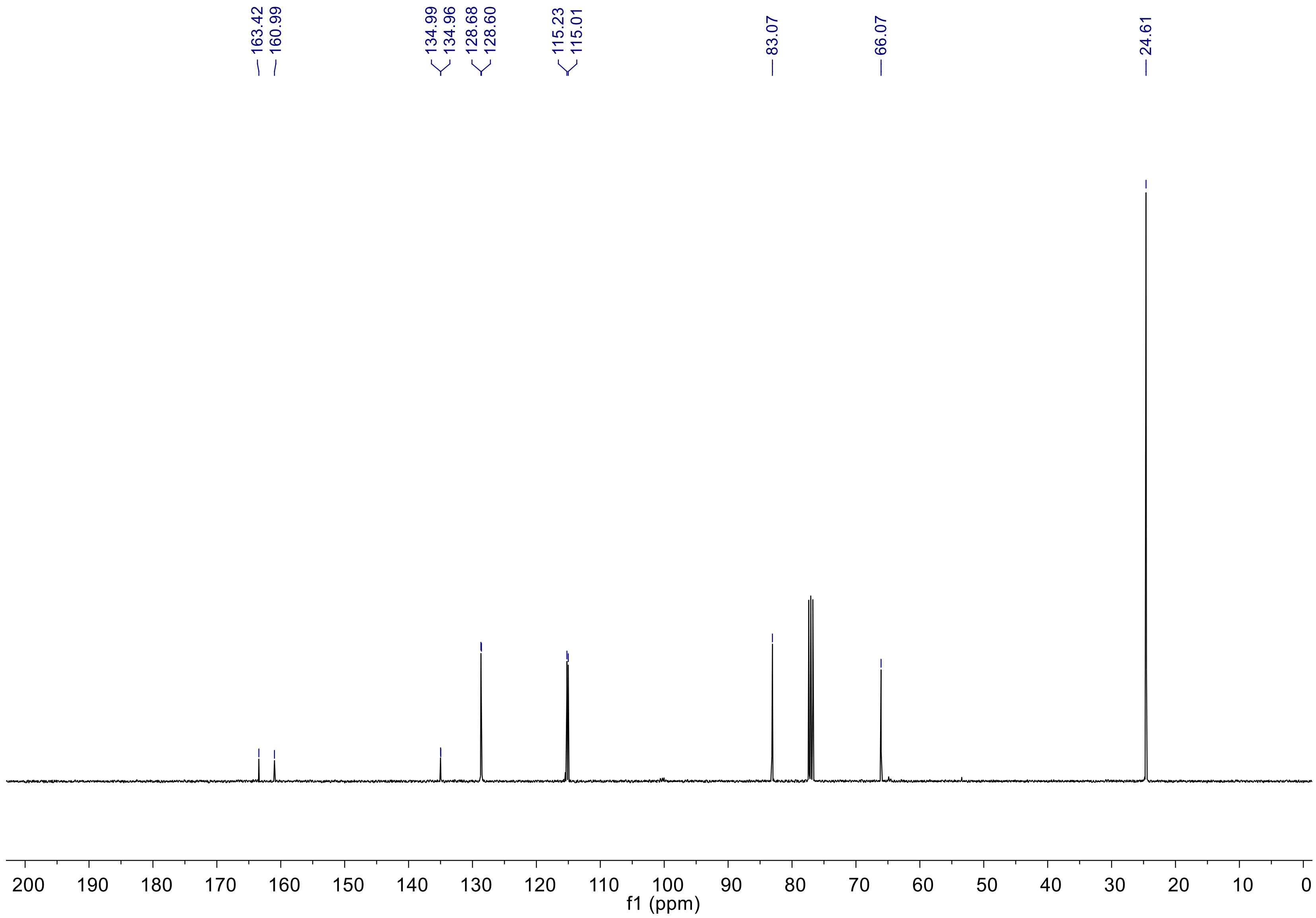

S89 ^{19}F NMR (471 MHz, $CDCl_3$, 298 K) spectrum of *2-((4-fluorobenzyl)oxy)-4,4,5,5-tetramethyl-1,3,2-dioxaborolane* **2d**.

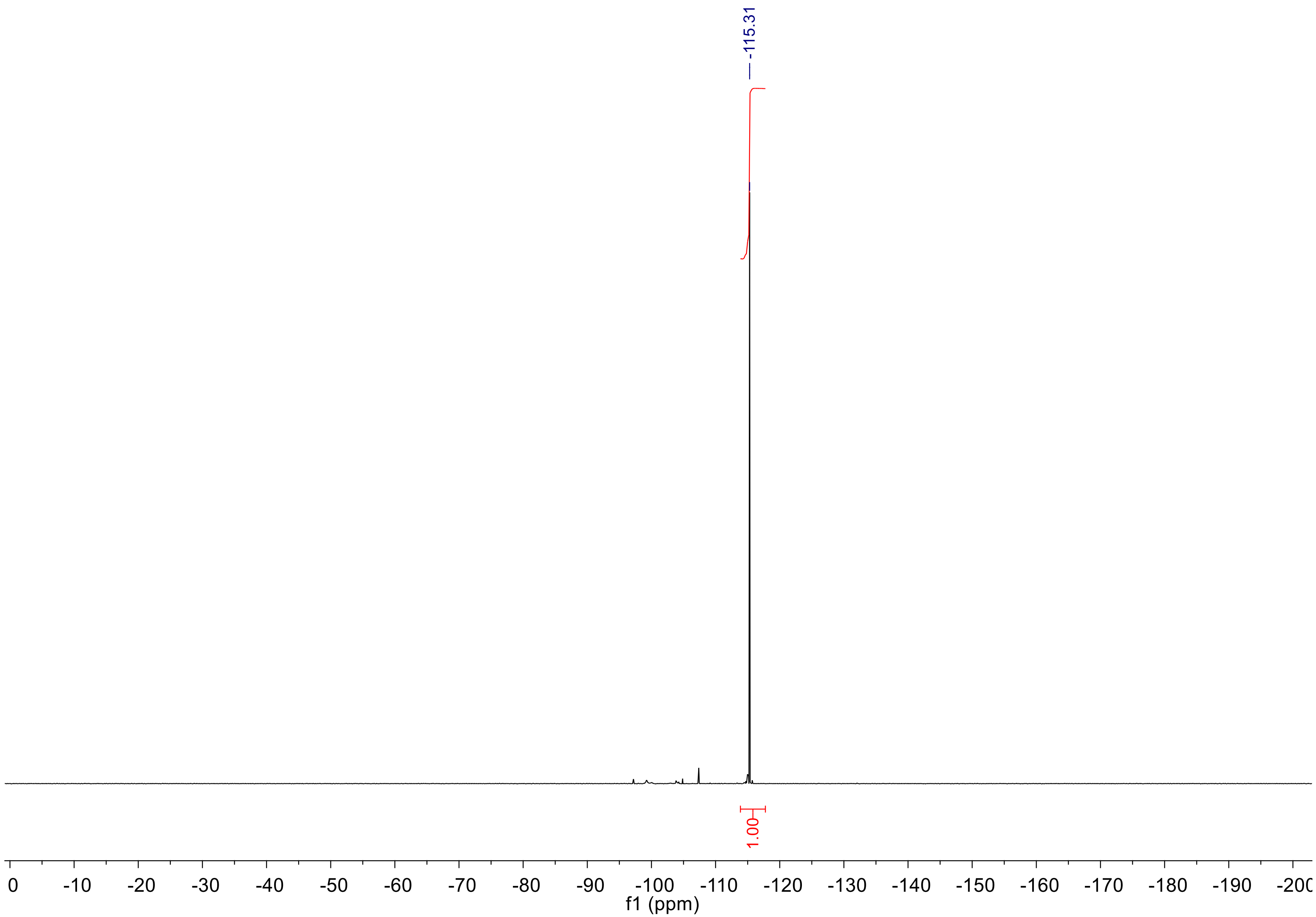

S90 ¹H NMR (500 MHz, $CDCl_3$, 298 K) spectrum of *2-((4-bromobenzyl)oxy)-4,4,5,5-tetramethyl-1,3,2-dioxaborolane* **2e**.

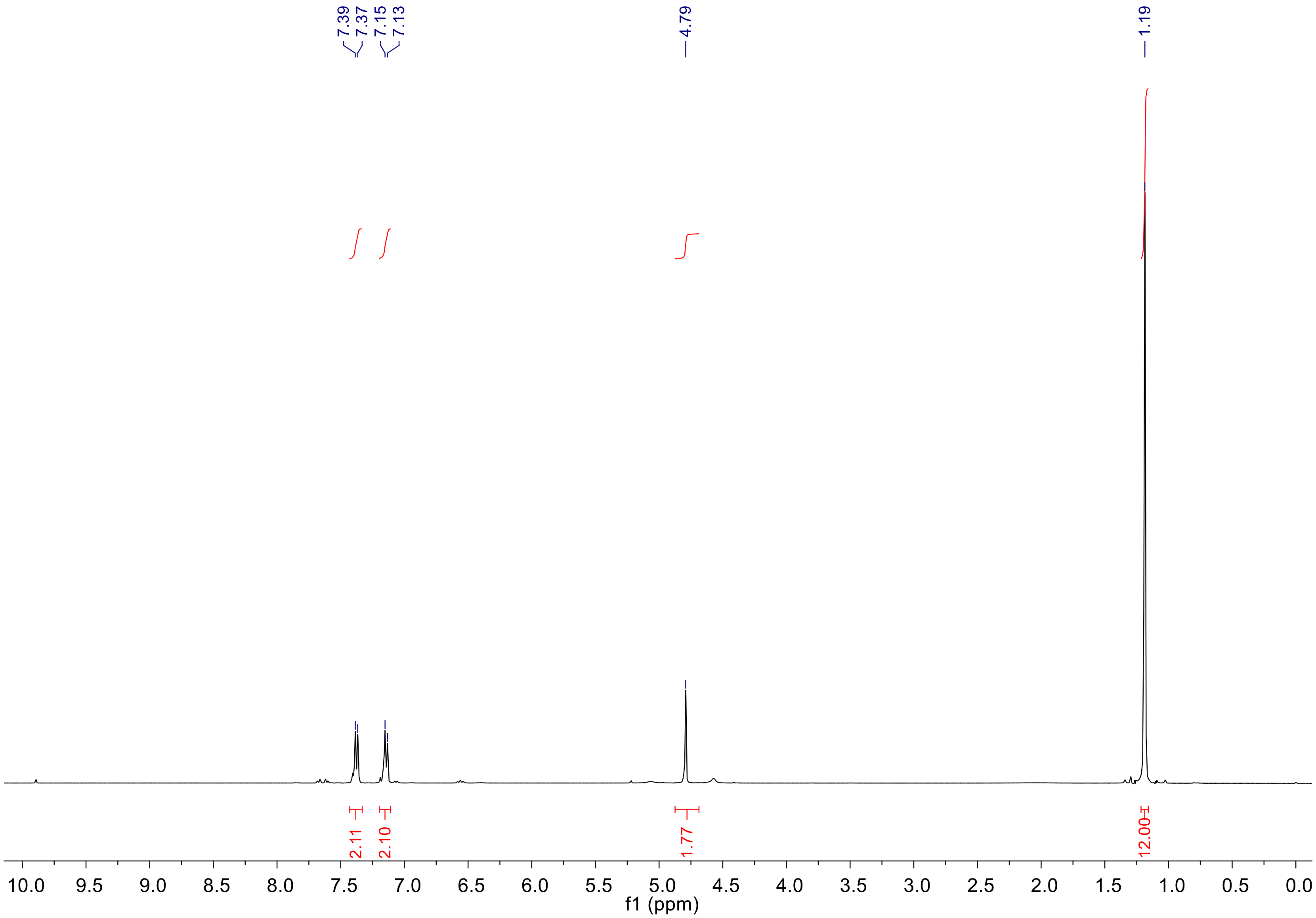

S91 ^{11}B NMR (160 MHz, $CDCl_3$, 298 K) spectrum of *2-((4-bromobenzyl)oxy)-4,4,5,5-tetramethyl-1,3,2-dioxaborolane* **2e**.

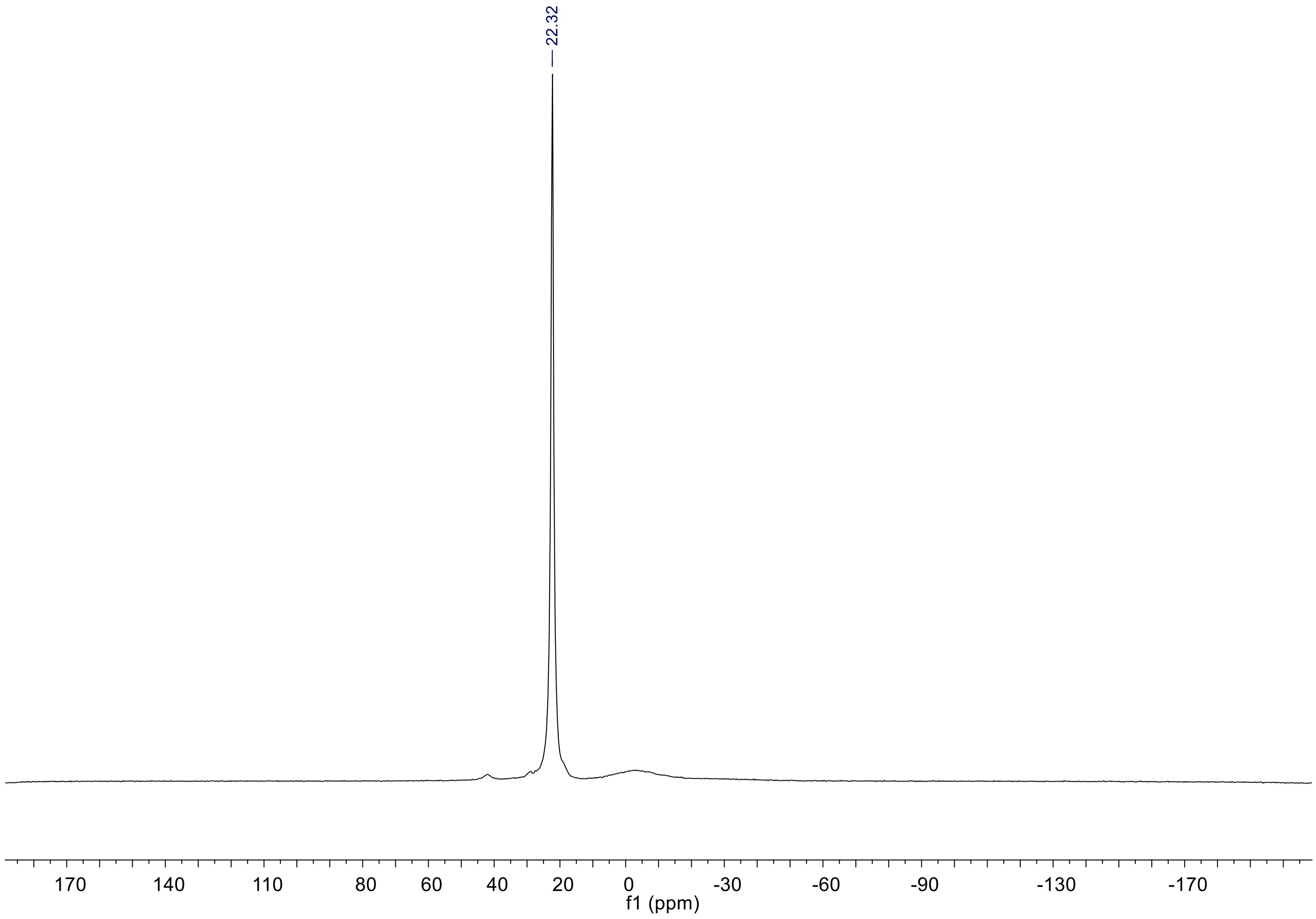

S92 ^{13}C NMR (126 MHz, $CDCl_3$, 298 K) spectrum of *2-((4-bromobenzyl)oxy)-4,4,5,5-tetramethyl-1,3,2-dioxaborolane* **2e**.

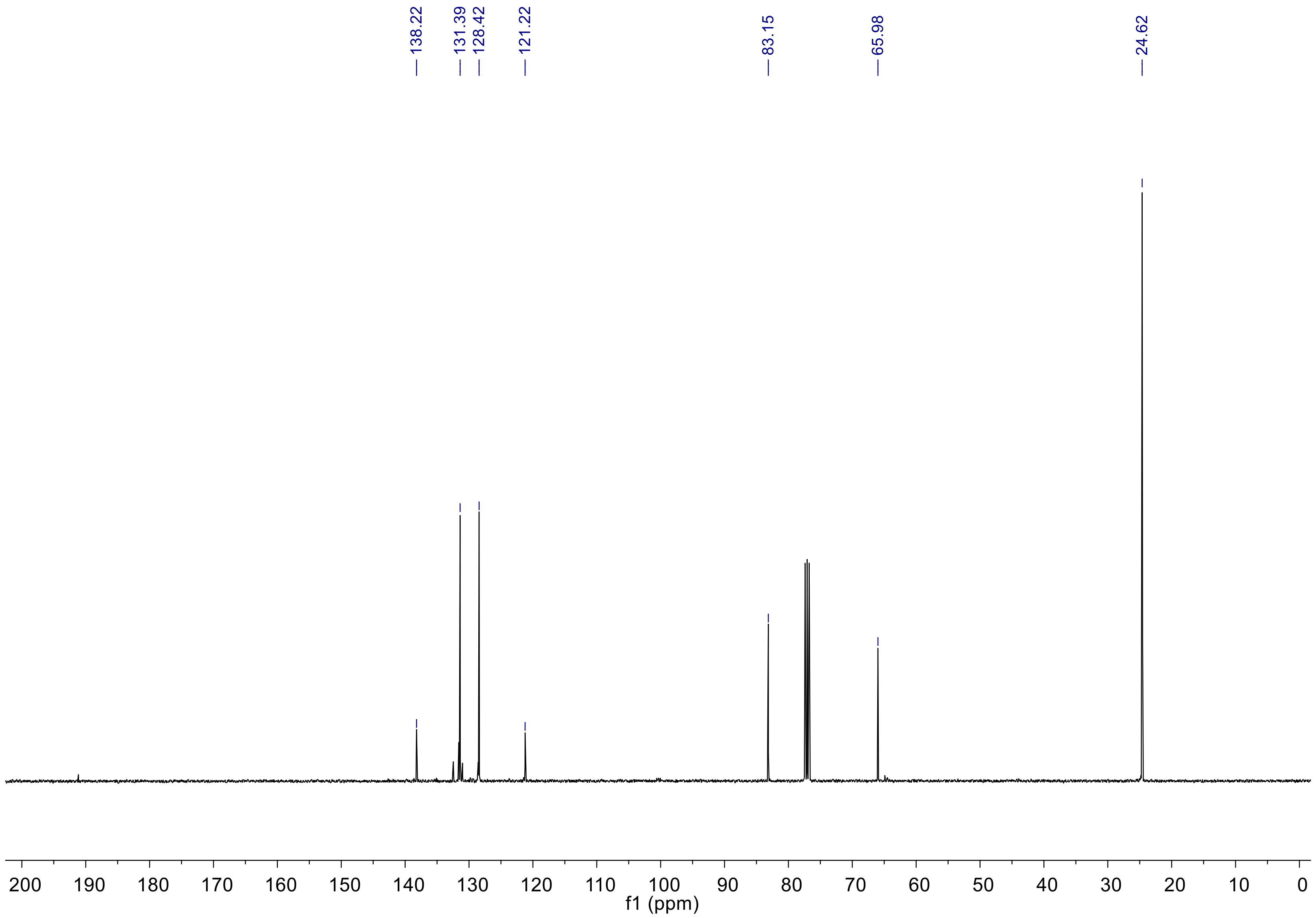

S93 ^{1}H NMR (500 MHz, $CDCl_3$, 298 K) spectrum of *2-((4-methoxybenzyl)oxy)-4,4,5,5-tetramethyl-1,3,2-dioxaborolane* **2f**.

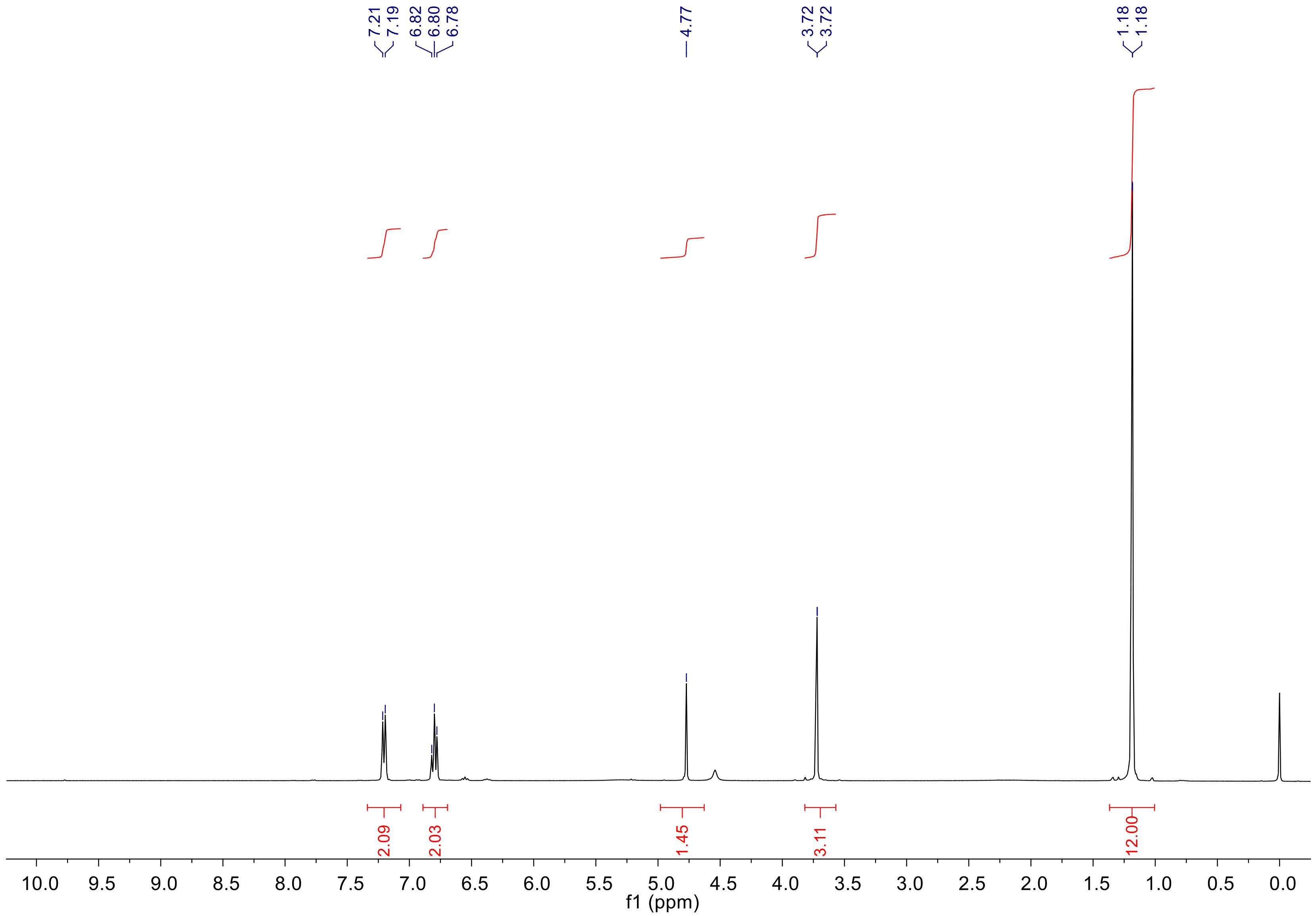

S94 ^{11}B NMR (160 MHz, $CDCl_3$, 298 K) spectrum of *2-((4-methoxybenzyl)oxy)-4,4,5,5-tetramethyl-1,3,2-dioxaborolane* **2f**.

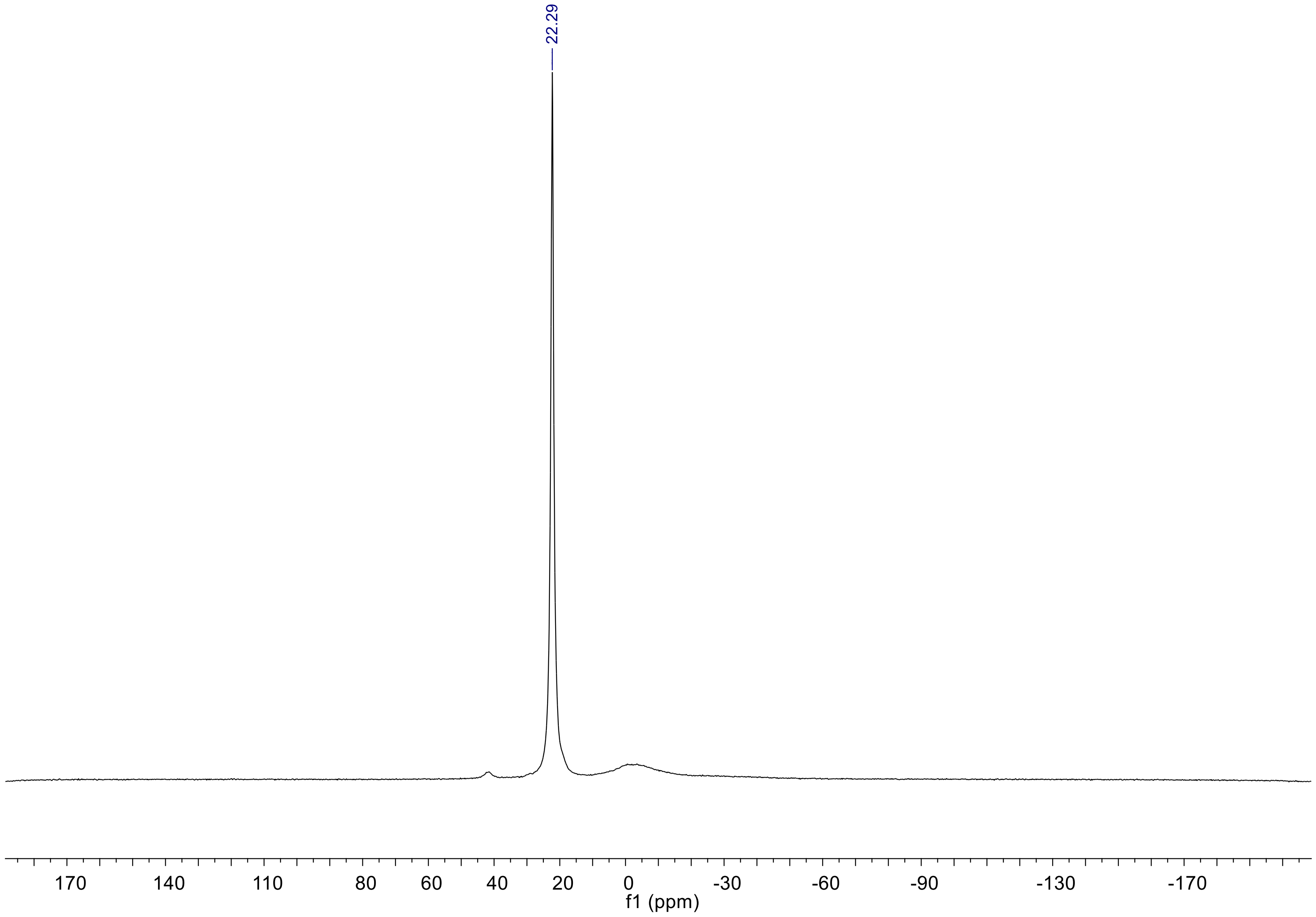

S95 ^{13}C NMR (126 MHz, $CDCl_3$, 298 K) spectrum of *2-((4-methoxybenzyl)oxy)-4,4,5,5-tetramethyl-1,3,2-dioxaborolane* **2f**.

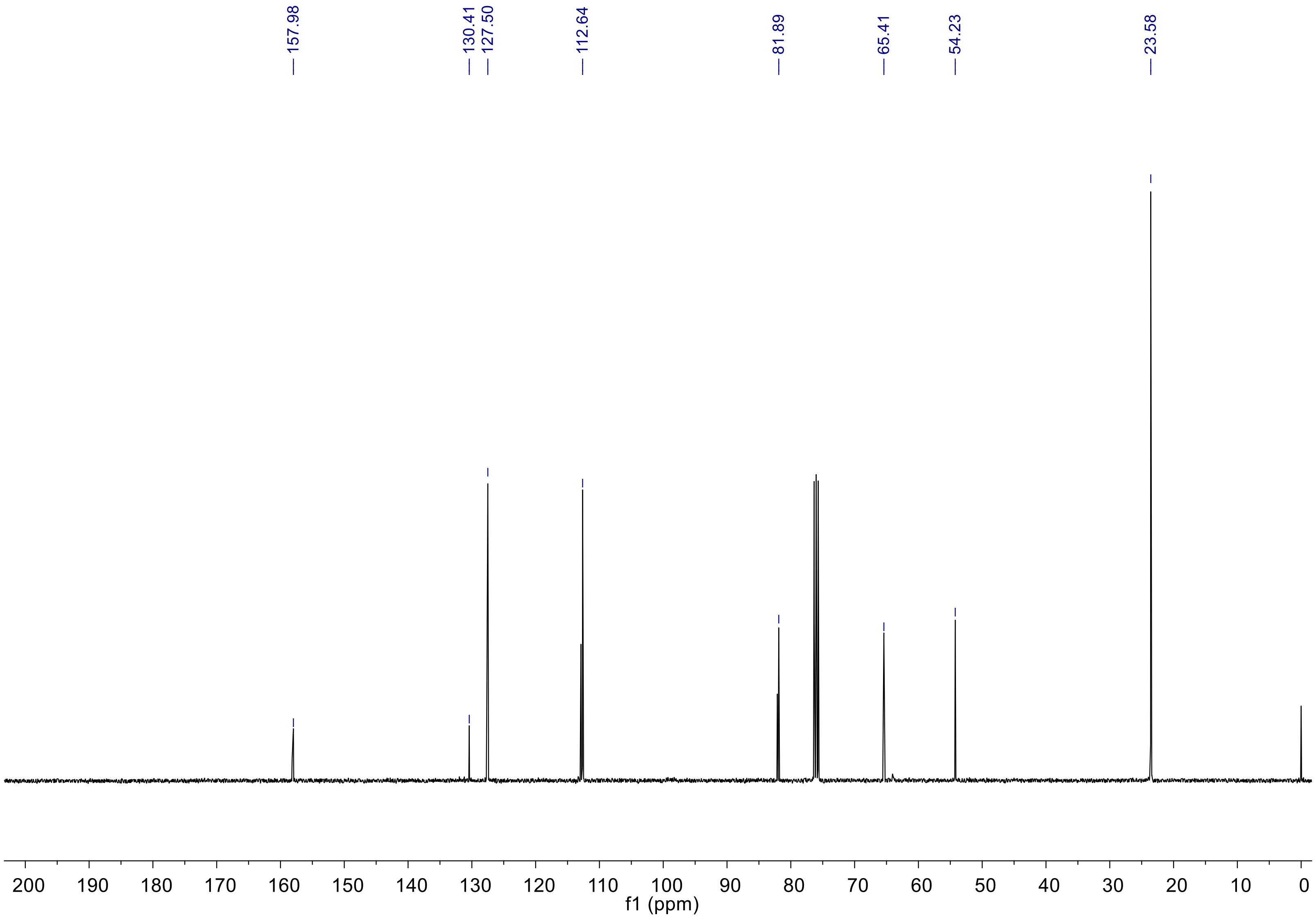

S96 ^{1}H NMR (500 MHz, $CDCl_3$, 298 K) spectrum of *4,4,5,5-tetramethyl-2-((4-nitrobenzyl)oxy)-1,3,2-dioxaborolane* **2g**.

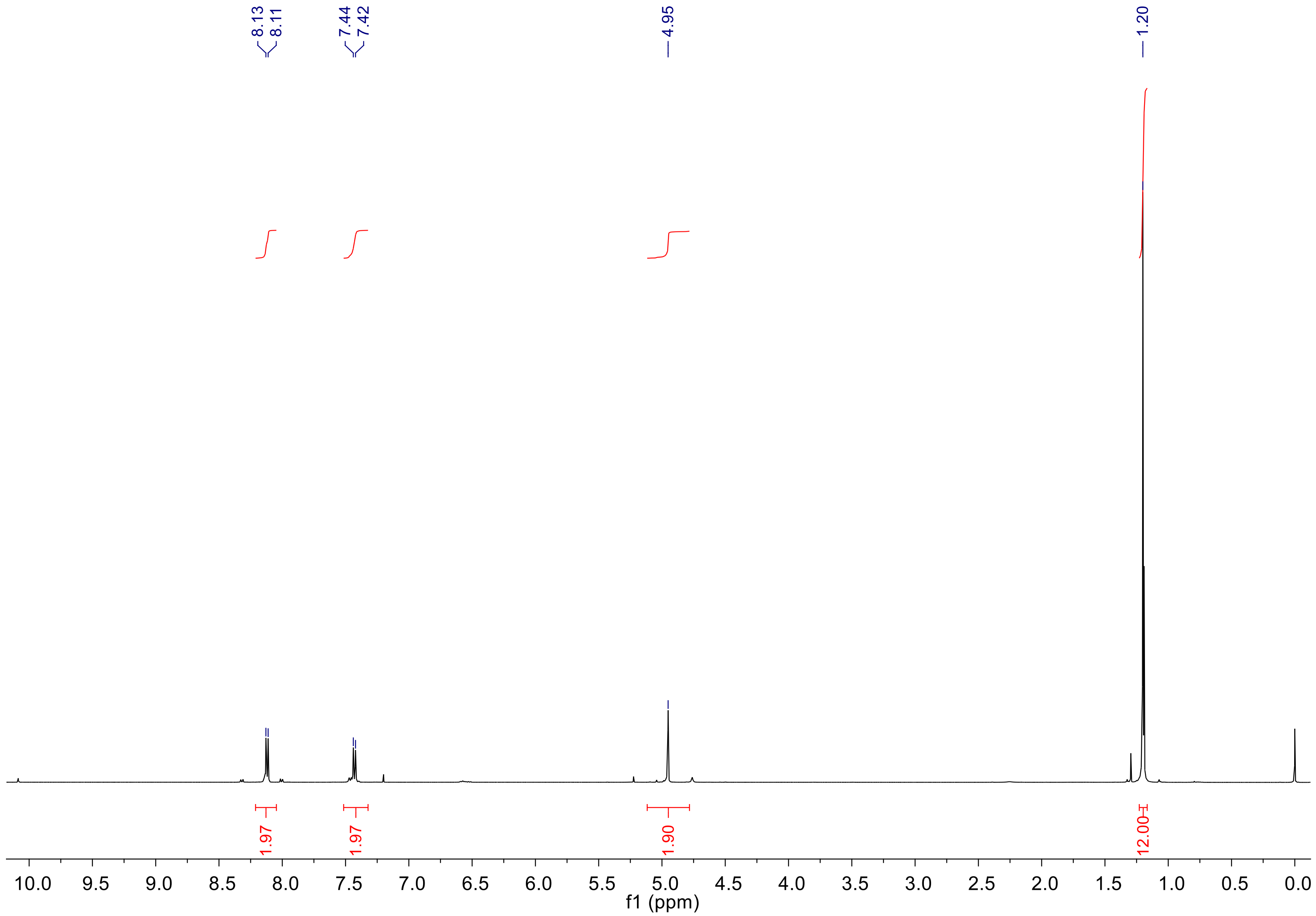

S97 ^{11}B NMR (160 MHz, $CDCl_3$, 298 K) spectrum of *4,4,5,5-tetramethyl-2-((4-nitrobenzyl)oxy)-1,3,2-dioxaborolane* **2g**.

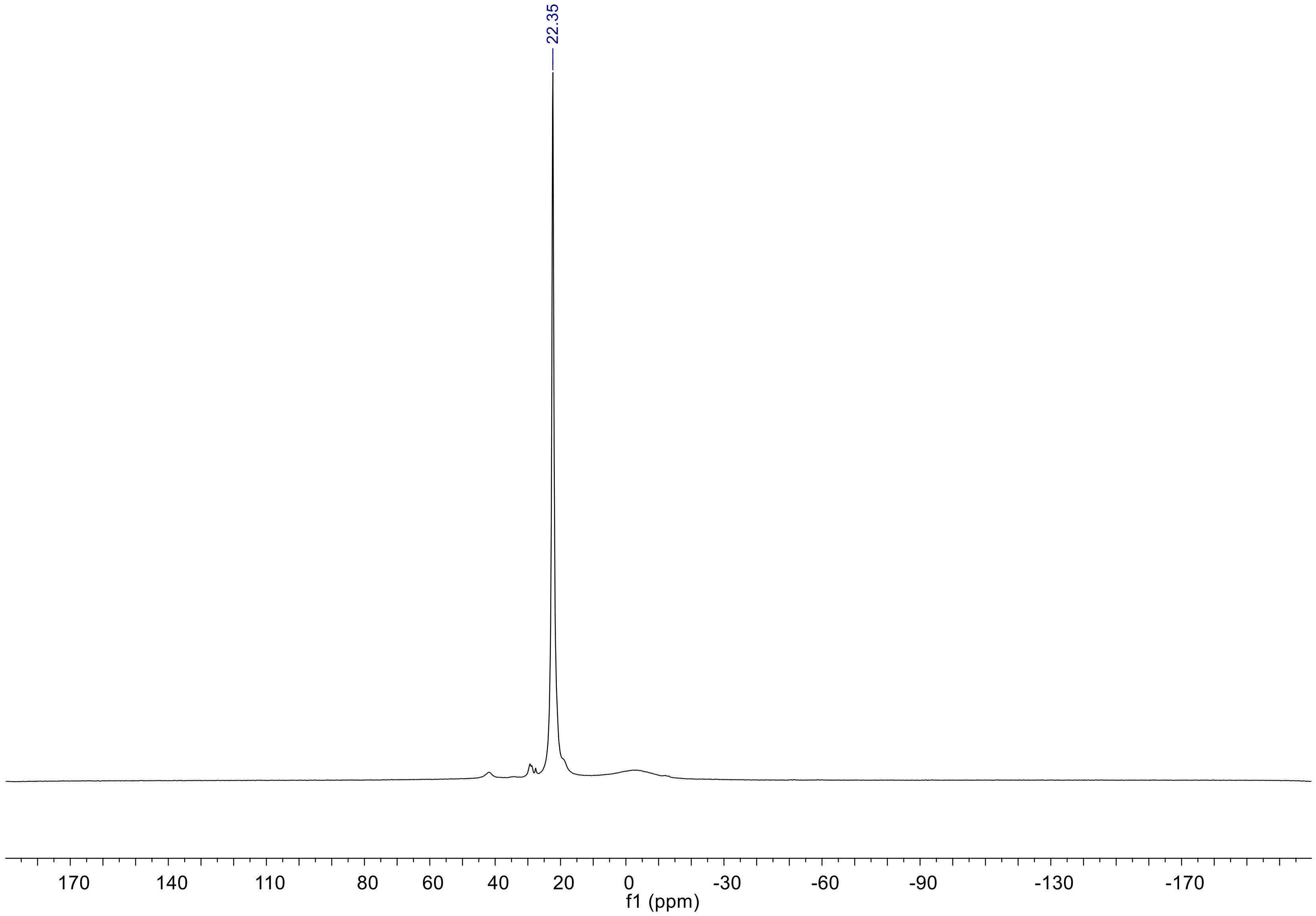

S98 ^{13}C NMR (126 MHz, $CDCl_3$, 298 K) spectrum of *4,4,5,5-tetramethyl-2-((4-nitrobenzyl)oxy)-1,3,2-dioxaborolane* **2g**.

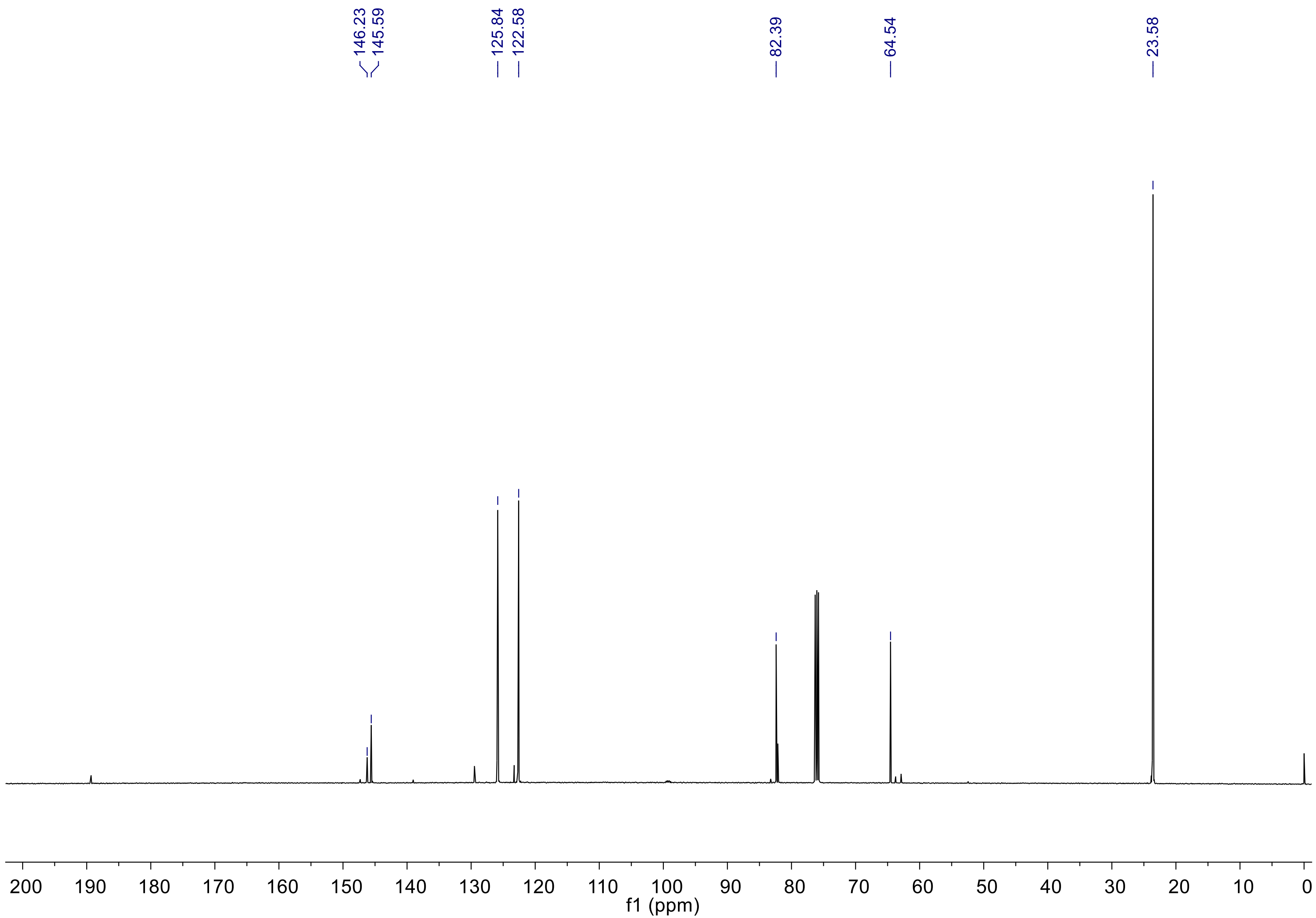

S99 ^{1}H NMR (500 MHz, $CDCl_3$, 298 K) spectrum of *4,4,5,5-tetramethyl-2-((4-(trifluoromethyl)benzyl)oxy)-1,3,2-dioxaborolane* **2h**.

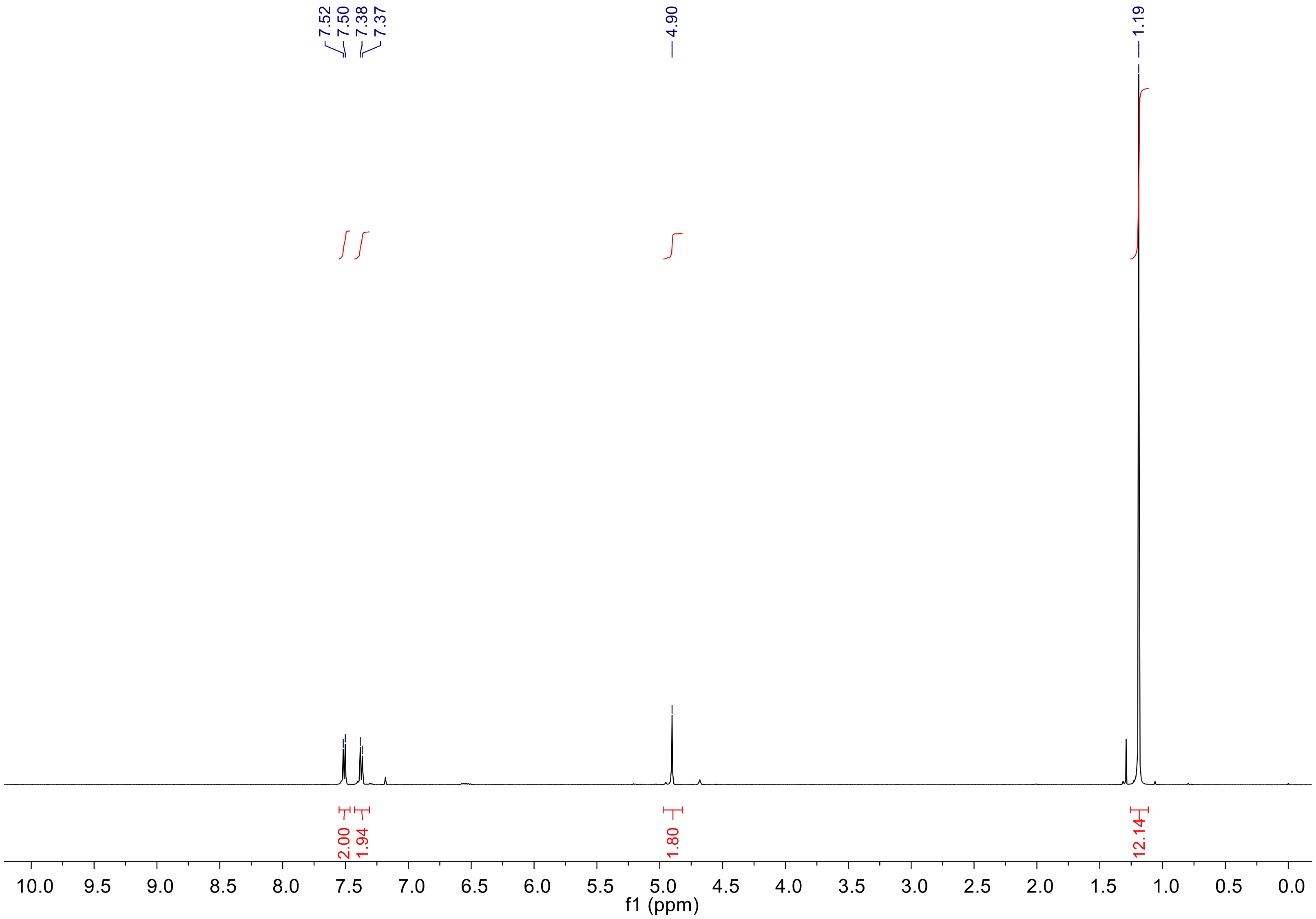

S100 ^{11}B NMR (160 MHz, $CDCl_3$, 298 K) spectrum of *4,4,5,5-tetramethyl-2-((4-(trifluoromethyl)benzyl)oxy)-1,3,2-dioxaborolane* **2h**.

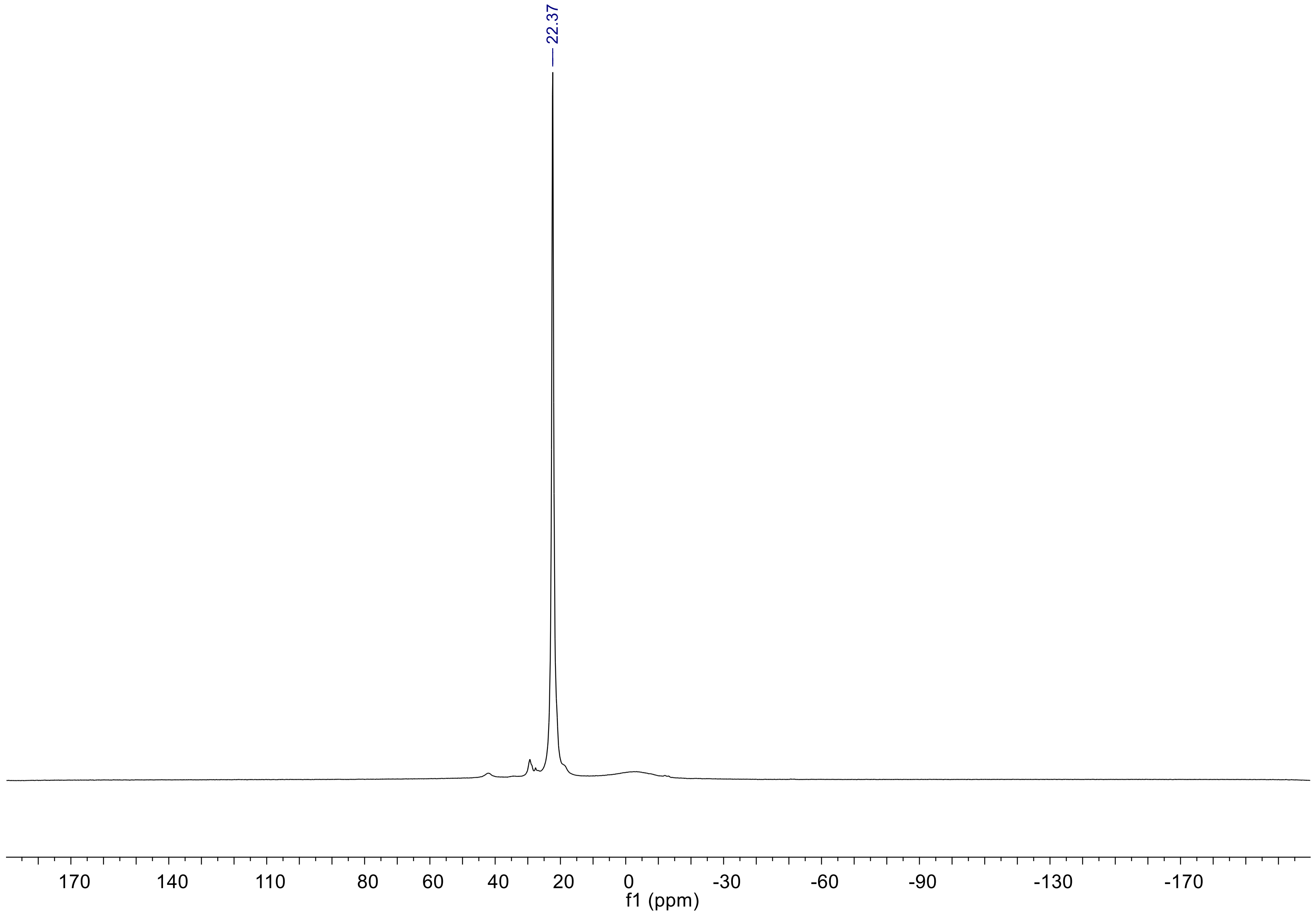

S101 ^{13}C NMR (126 MHz, $CDCl_3$, 298 K) spectrum of *4,4,5,5-tetramethyl-2-((4-(trifluoromethyl)benzyl)oxy)-1,3,2-dioxaborolane* **2h**.

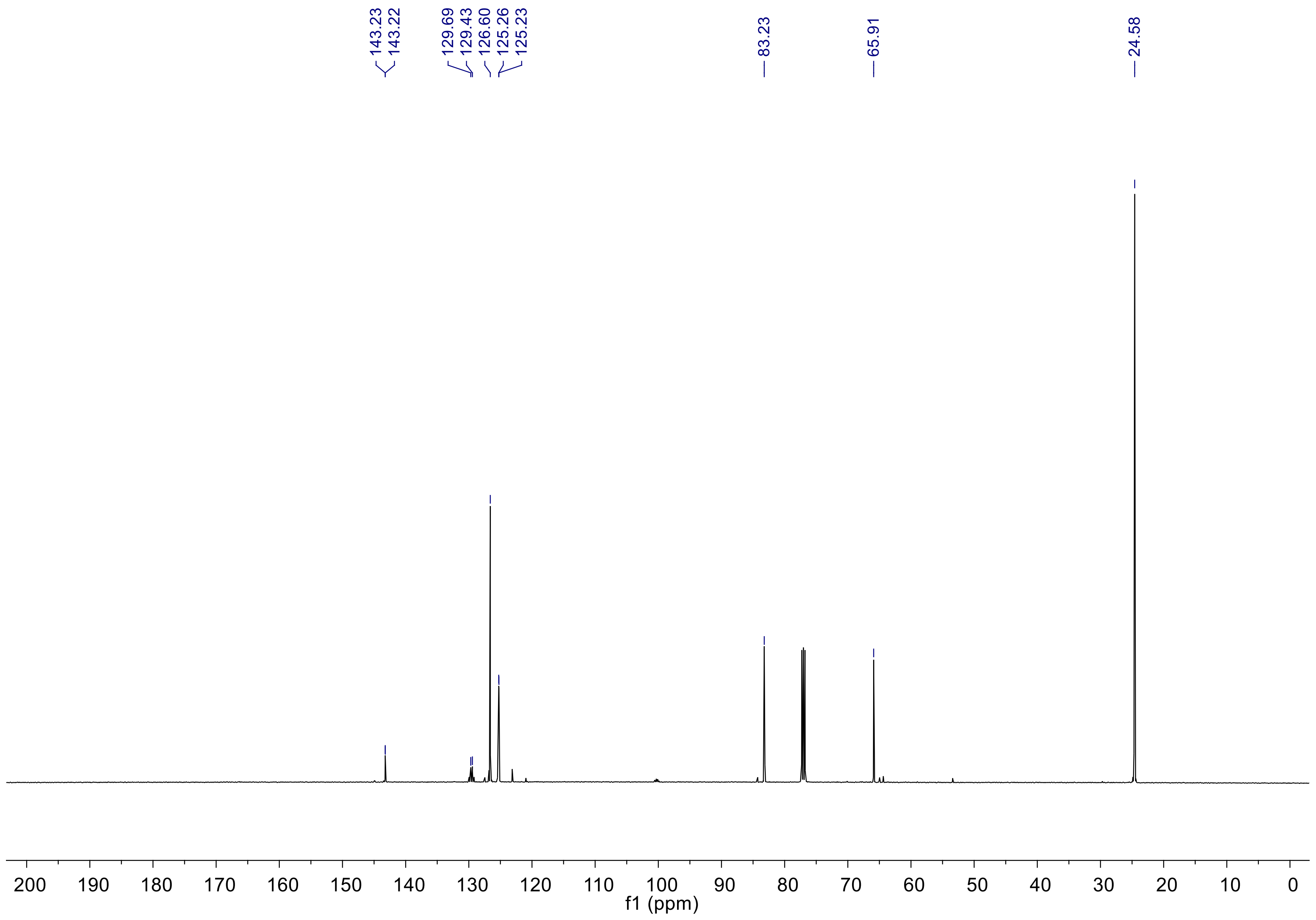

S102 ^{19}F NMR (471 MHz, $CDCl_3$, 298 K) spectrum of *4,4,5,5-tetramethyl-2-((4-(trifluoromethyl)benzyl)oxy)-1,3,2-dioxaborolane* **2h**.

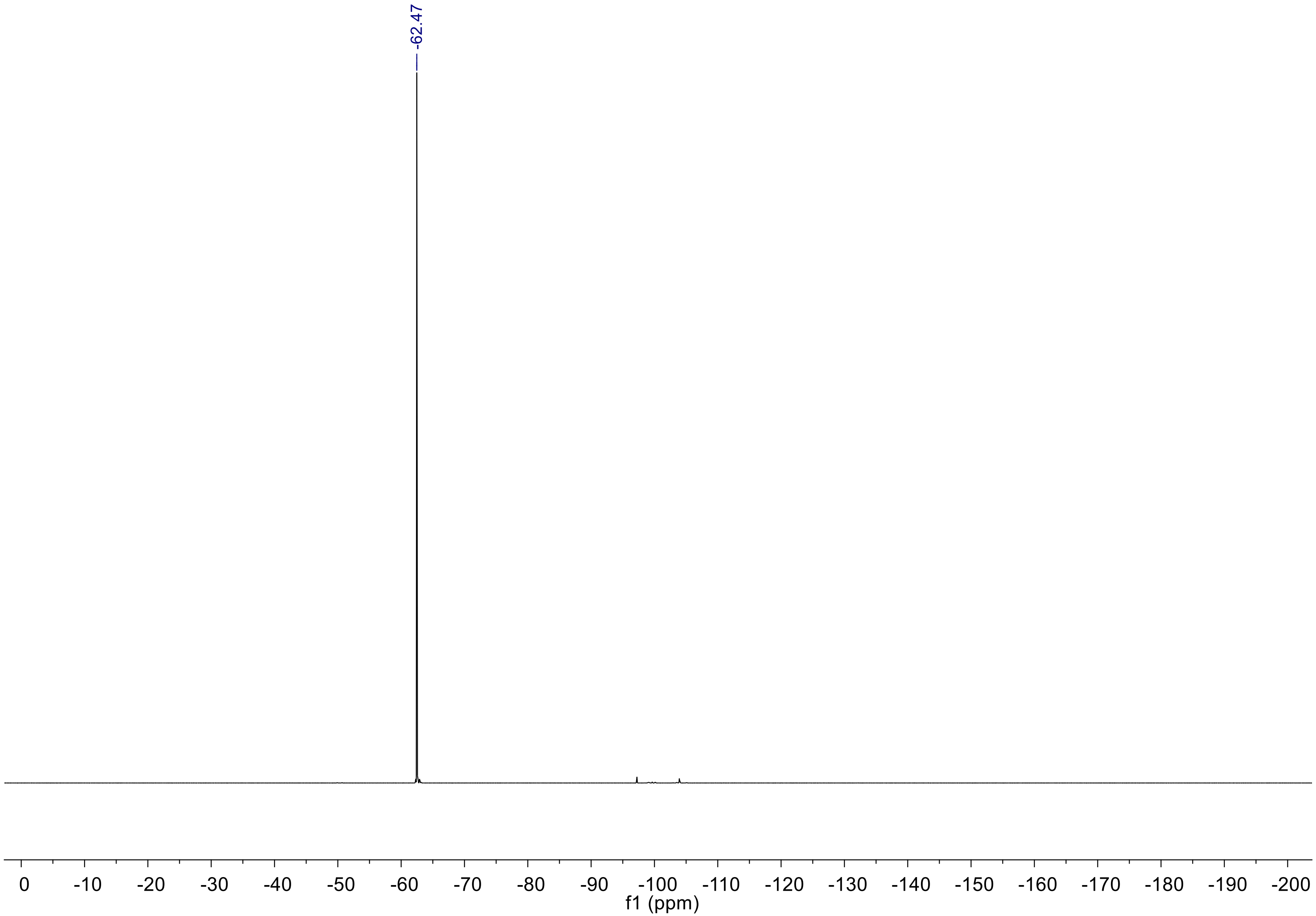

S103 ^{1}H NMR (400 MHz, $CDCl_3$, 298 K) spectrum of *2-((2-bromobenzyl)oxy)-4,4,5,5-tetramethyl-1,3,2-dioxaborolane* **2i**.

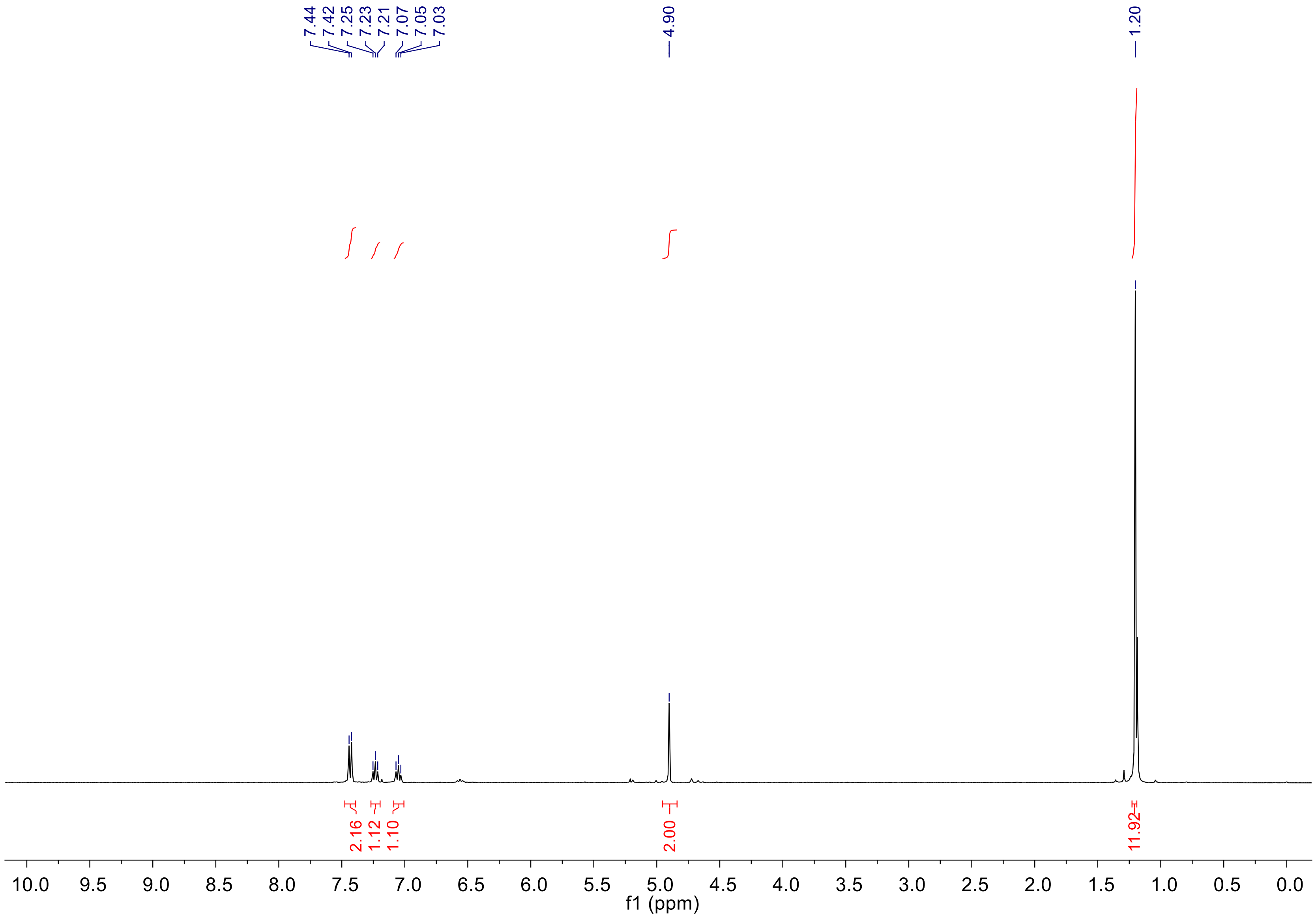

S104 ^{11}B NMR (128 MHz, $CDCl_3$, 298 K) spectrum of *2-((2-bromobenzyl)oxy)-4,4,5,5-tetramethyl-1,3,2-dioxaborolane* **2i**.

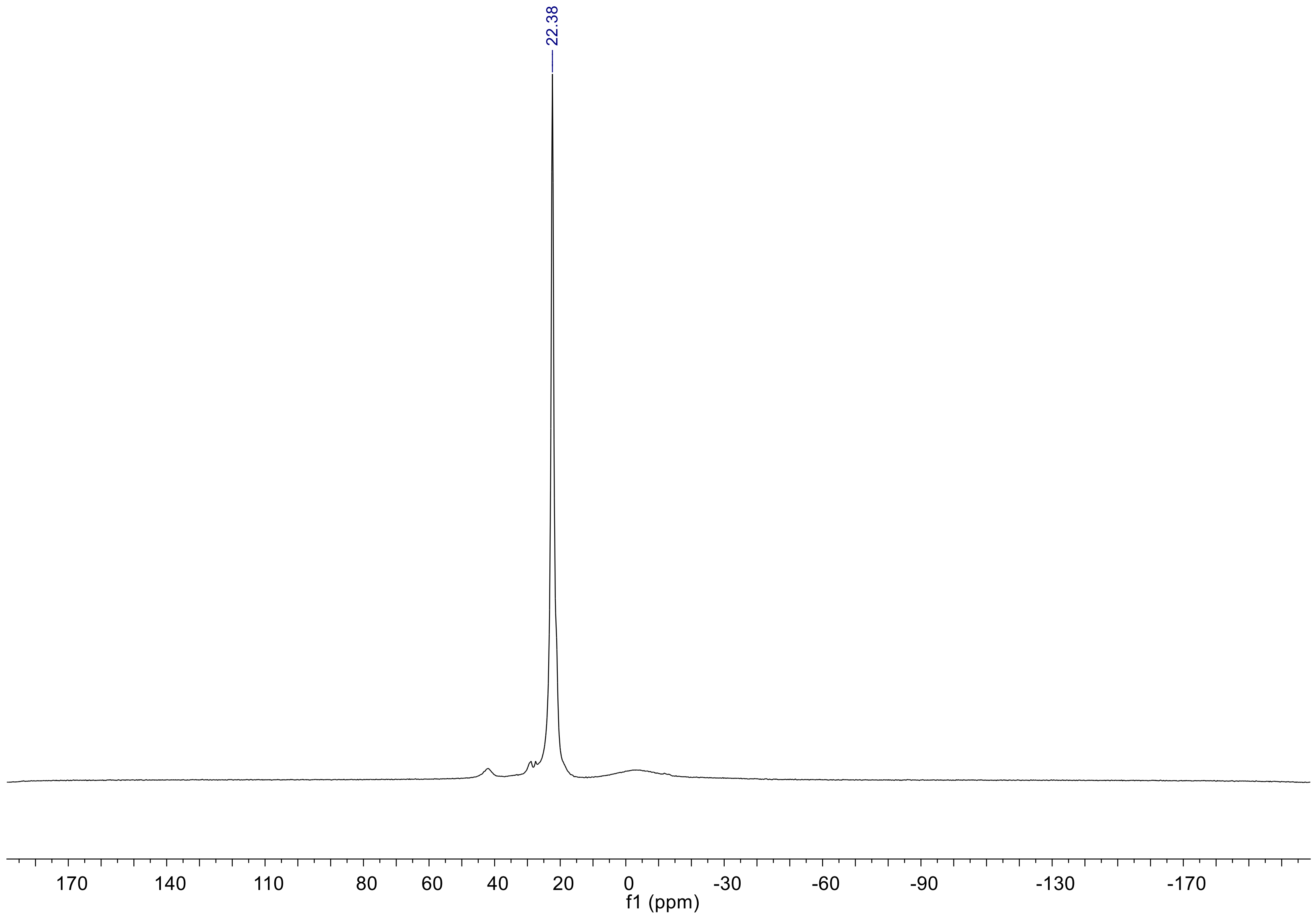

S105 ^{13}C NMR (101 MHz, $CDCl_3$, 298 K) spectrum of *2-((2-bromobenzyl)oxy)-4,4,5,5-tetramethyl-1,3,2-dioxaborolane* **2i**.

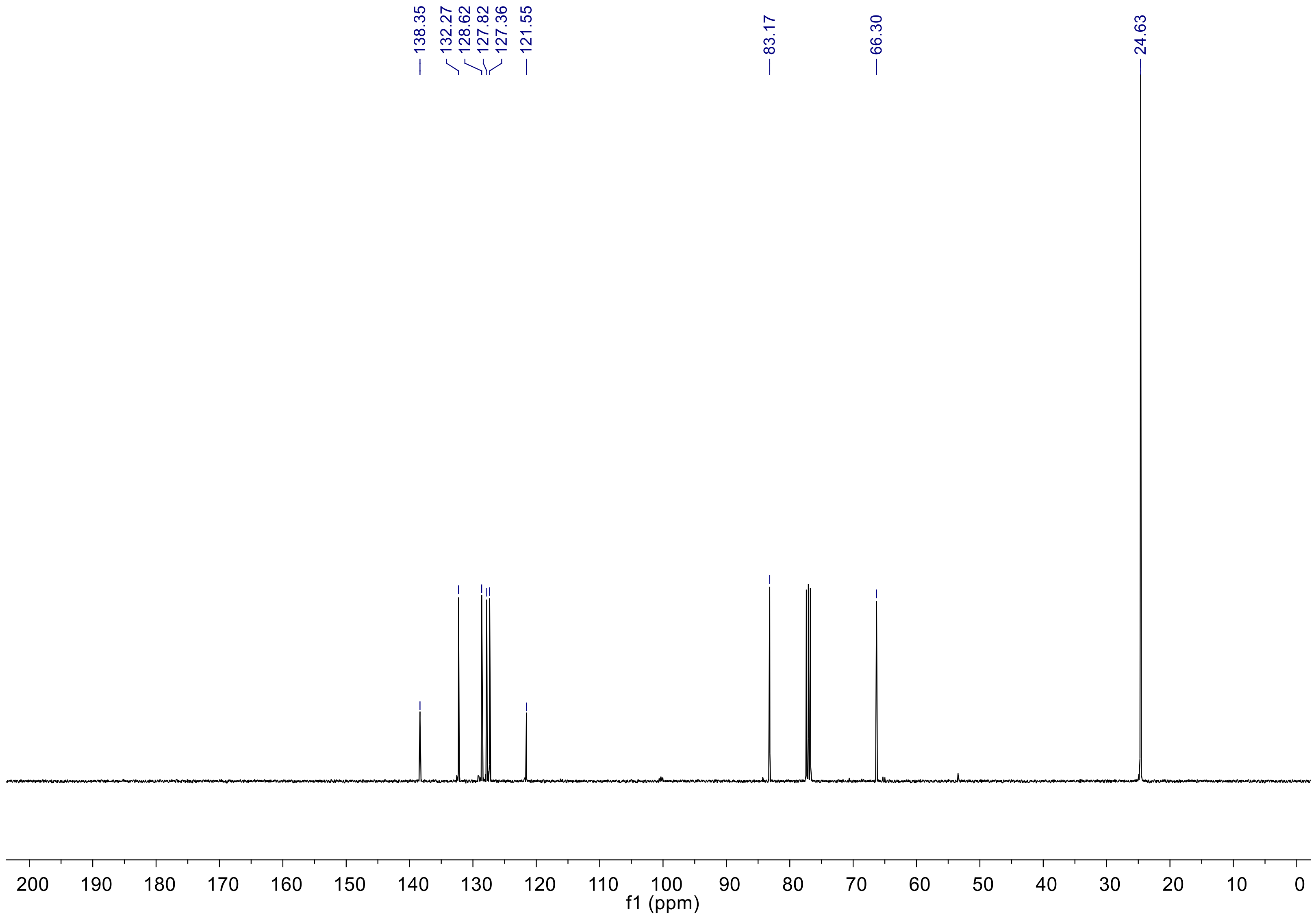

S106 ^{1}H NMR (500 MHz, $CDCl_3$, 298 K) spectrum of *2-((2-methoxybenzyl)oxy)-4,4,5,5-tetramethyl-1,3,2-dioxaborolane* **2j**.

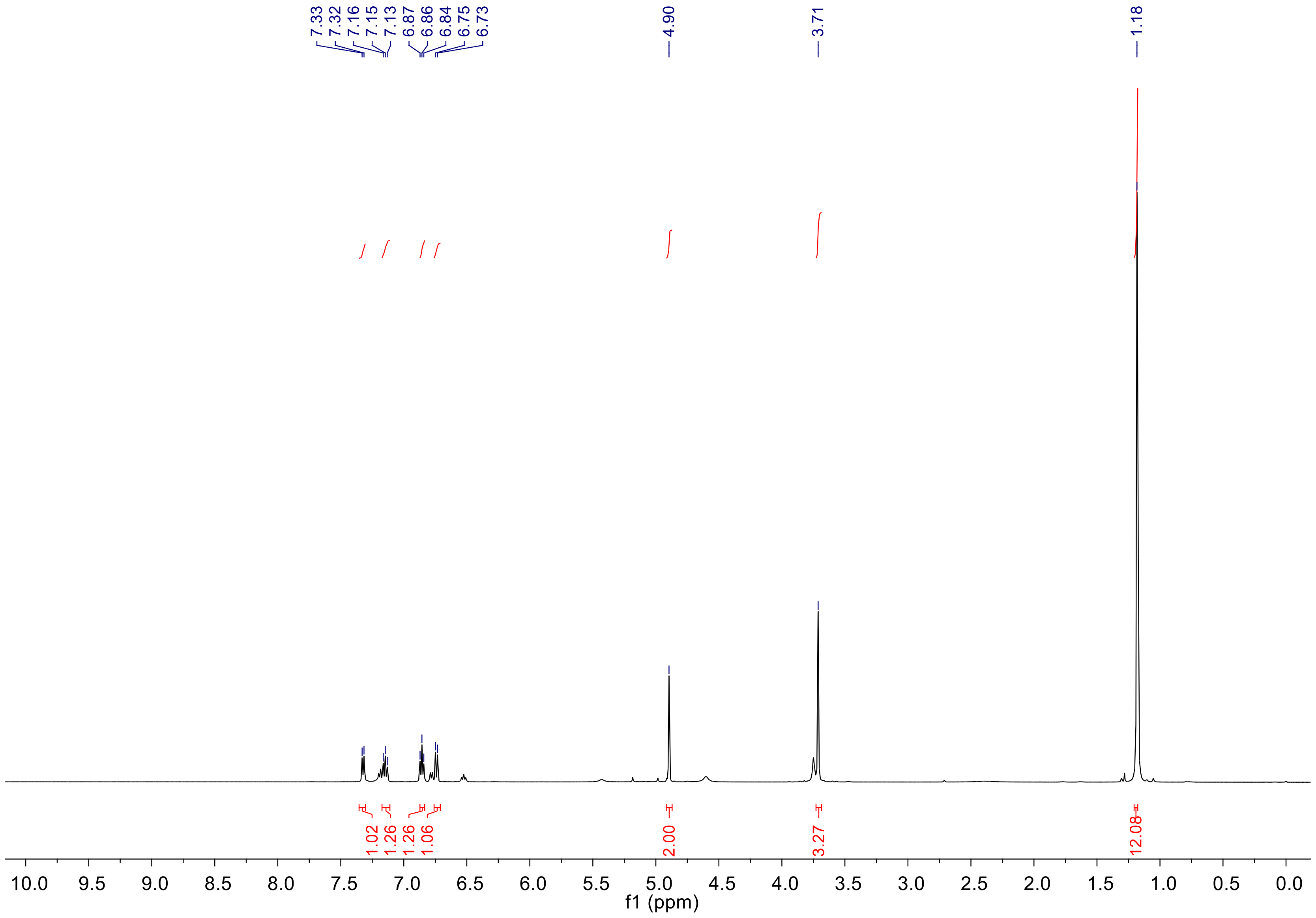

S107 ^{11}B NMR (160 MHz, $CDCl_3$, 298 K) spectrum of *2-((2-methoxybenzyl)oxy)-4,4,5,5-tetramethyl-1,3,2-dioxaborolane* **2j**.

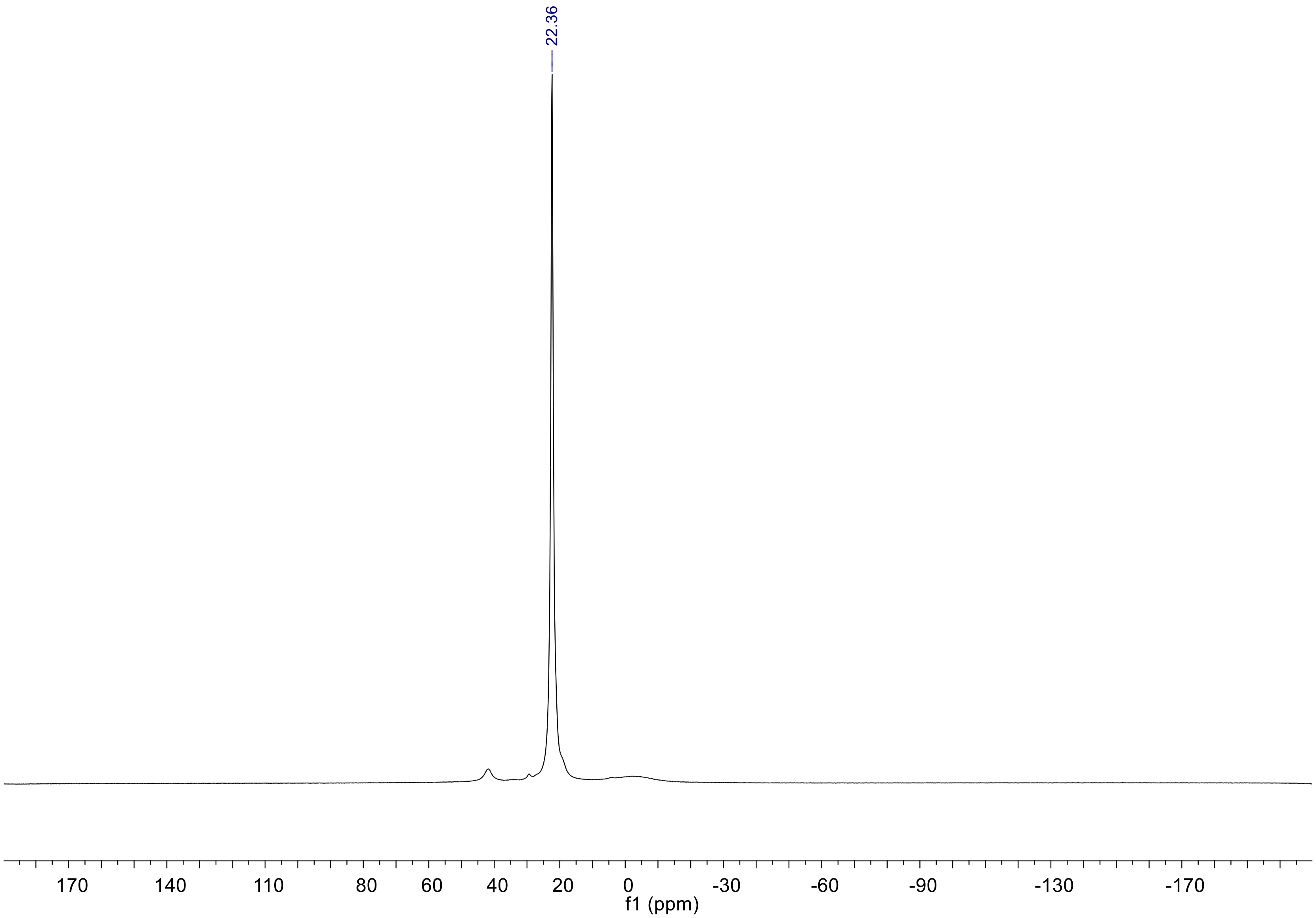

S108 ^{13}C NMR (126 MHz, $CDCl_3$, 298 K) spectrum of *2-((2-methoxybenzyl)oxy)-4,4,5,5-tetramethyl-1,3,2-dioxaborolane* **2j**.

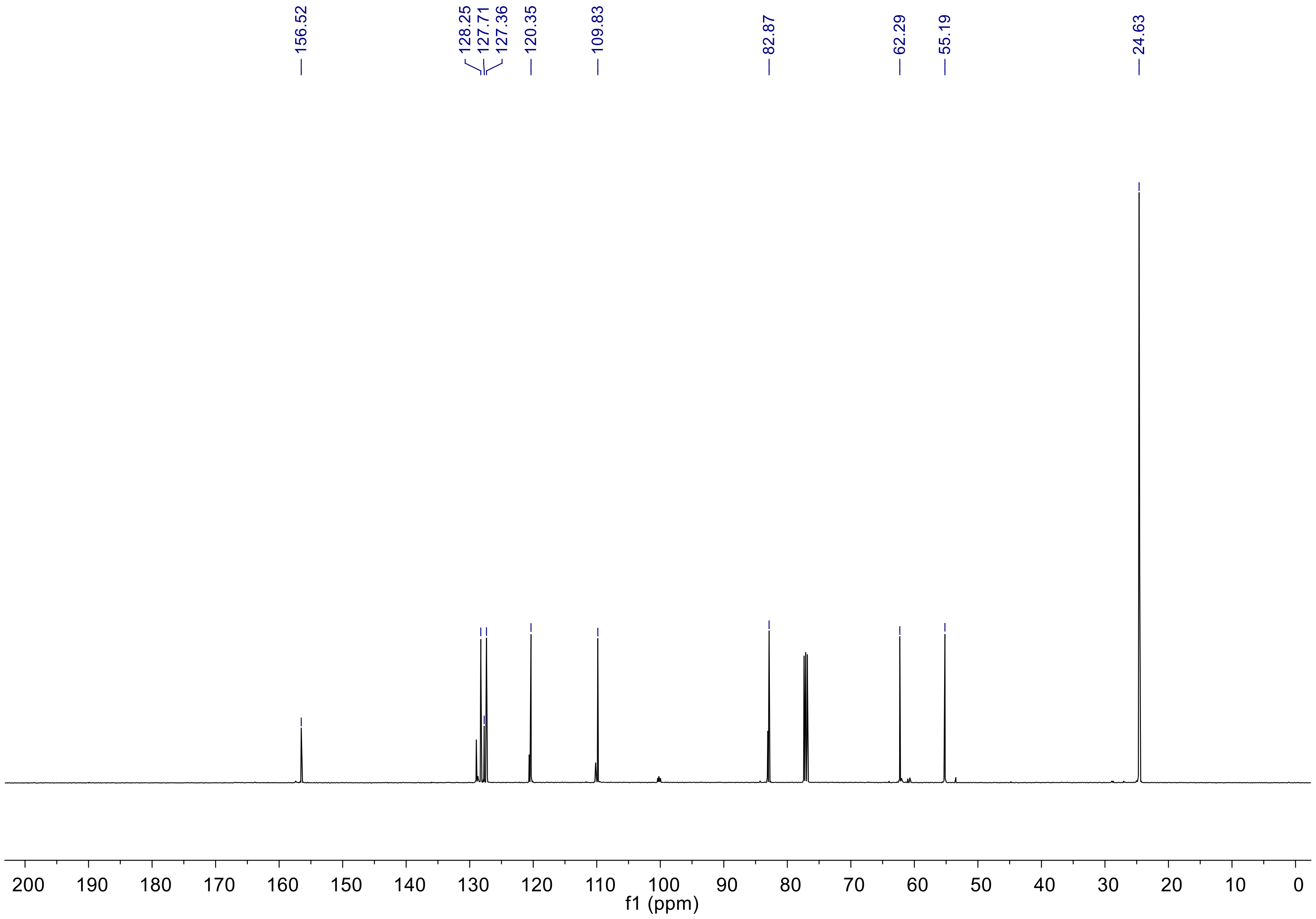

S109 ^{1}H NMR (400 MHz, $CDCl_3$, 298 K) spectrum of the hydrolysis product of *4,4,5,5-tetramethyl-2-((2-nitrobenzyl)oxy)-1,3,2-dioxaborolane* **2k**.

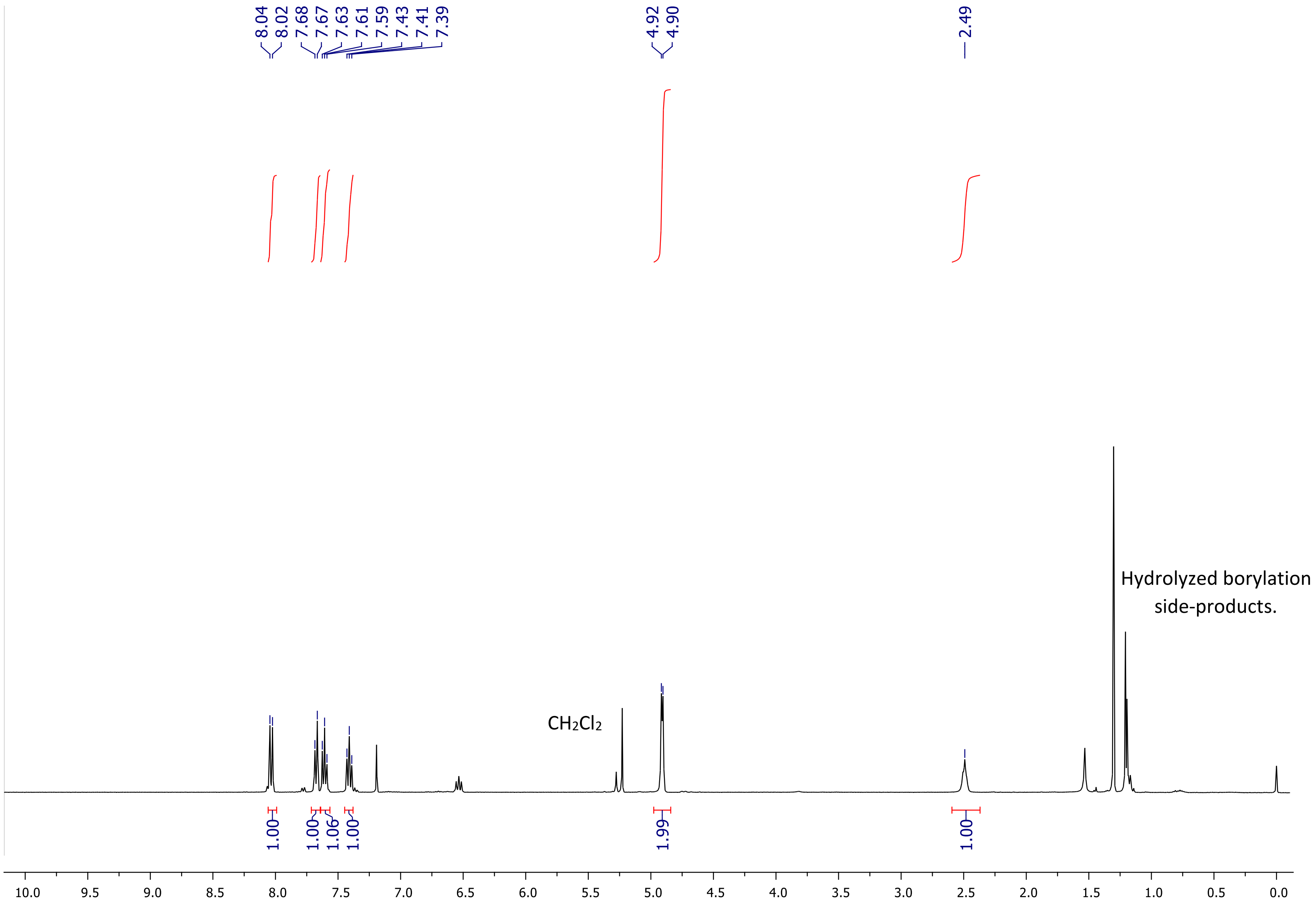

S110 ^{13}C NMR (101 MHz, $CDCl_3$, 298 K) spectrum of the hydrolysis product of *4,4,5,5-tetramethyl-2-((2-nitrobenzyl)oxy)-1,3,2-dioxaborolane* **2k**.

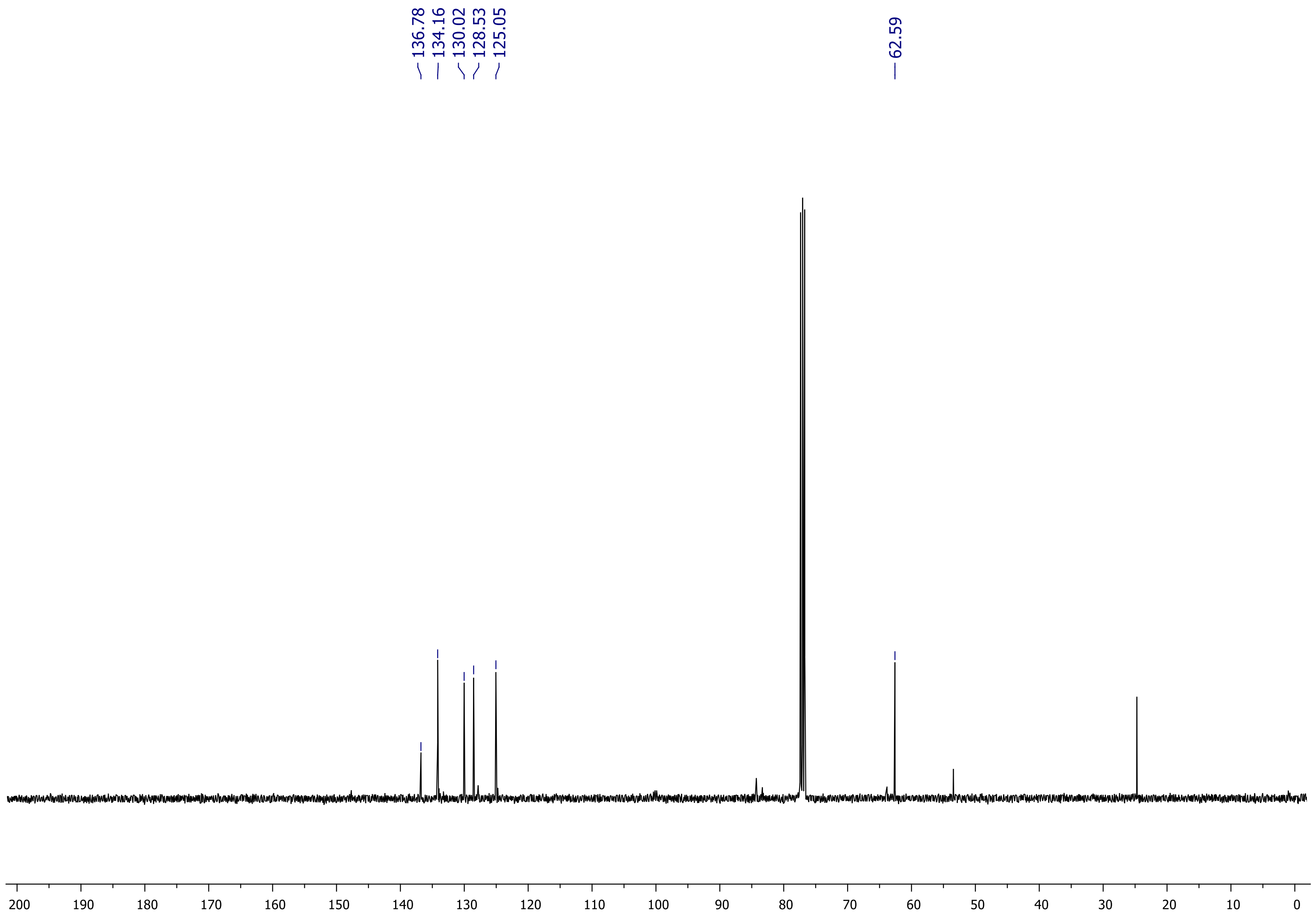

S111 ^{1}H NMR (500 MHz, $CDCl_3$, 298 K) spectrum of *2-(((4,4,5,5-tetramethyl-1,3,2-dioxaborolan-2-yl)oxy)methyl)benzonitrile* **2l**.

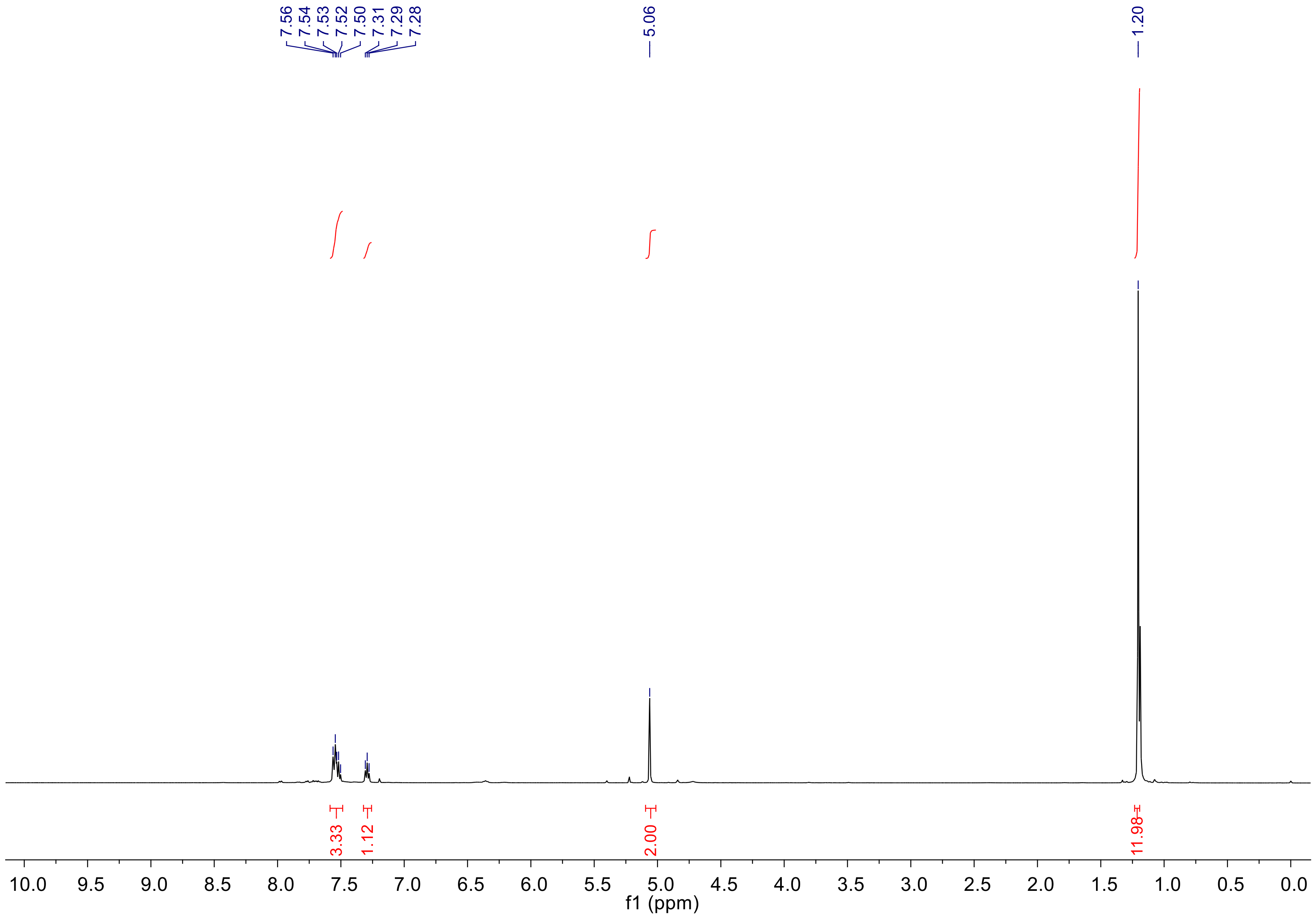

S112 ^{11}B NMR (160 MHz, $CDCl_3$, 298 K) spectrum of *2-(((4,4,5,5-tetramethyl-1,3,2-dioxaborolan-2-yl)oxy)methyl)benzonitrile* **2l**.

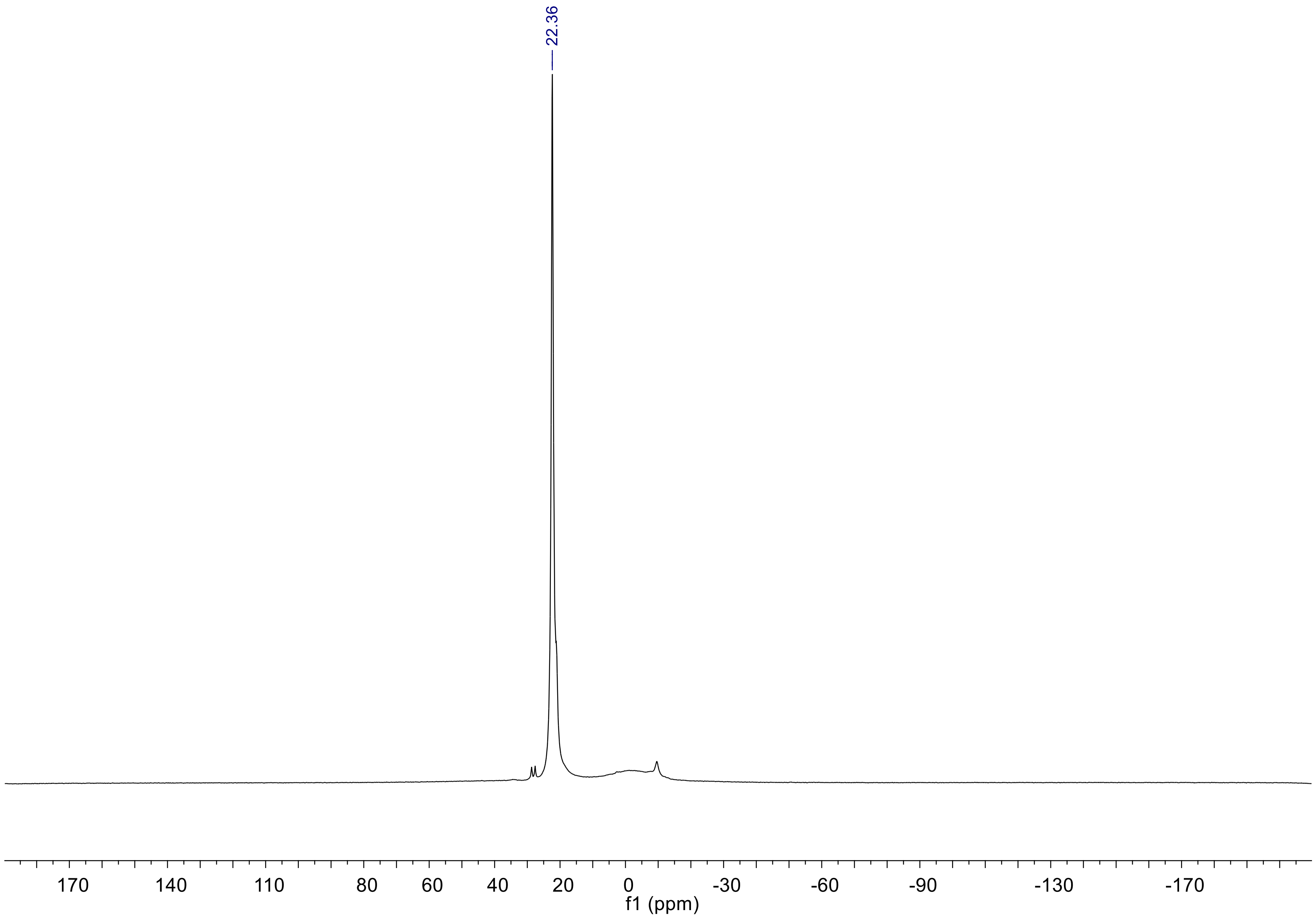

S113 ^{13}C NMR (126 MHz, $CDCl_3$, 298 K) spectrum of *2-(((4,4,5,5-tetramethyl-1,3,2-dioxaborolan-2-yl)oxy)methyl)benzonitrile* **2l**.

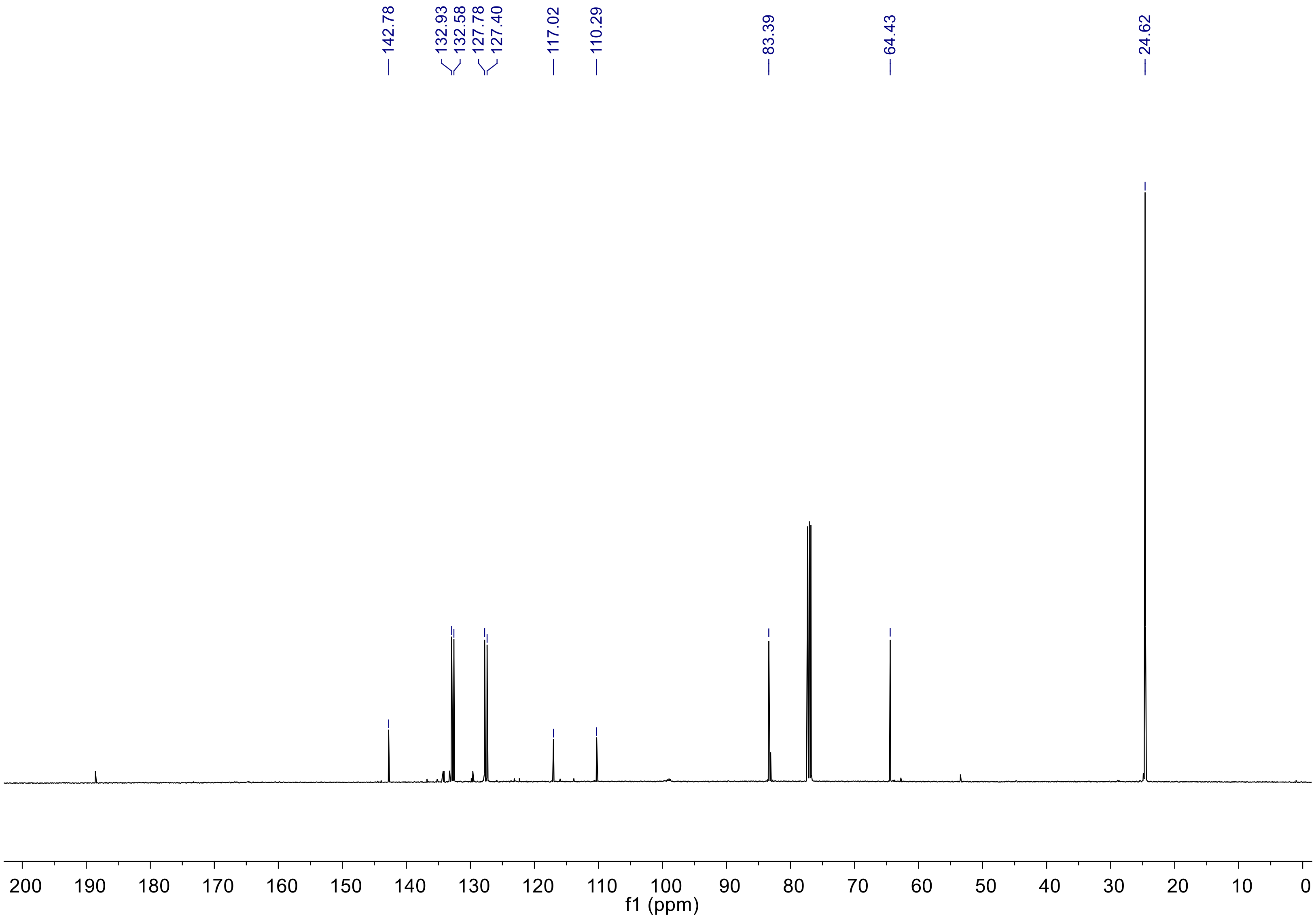

S114 ^{1}H NMR (500 MHz, $CDCl_3$, 298 K) spectrum of *4,4,5,5-tetramethyl-2-((perfluorophenyl)methoxy)-1,3,2-dioxaborolane* **2m**.

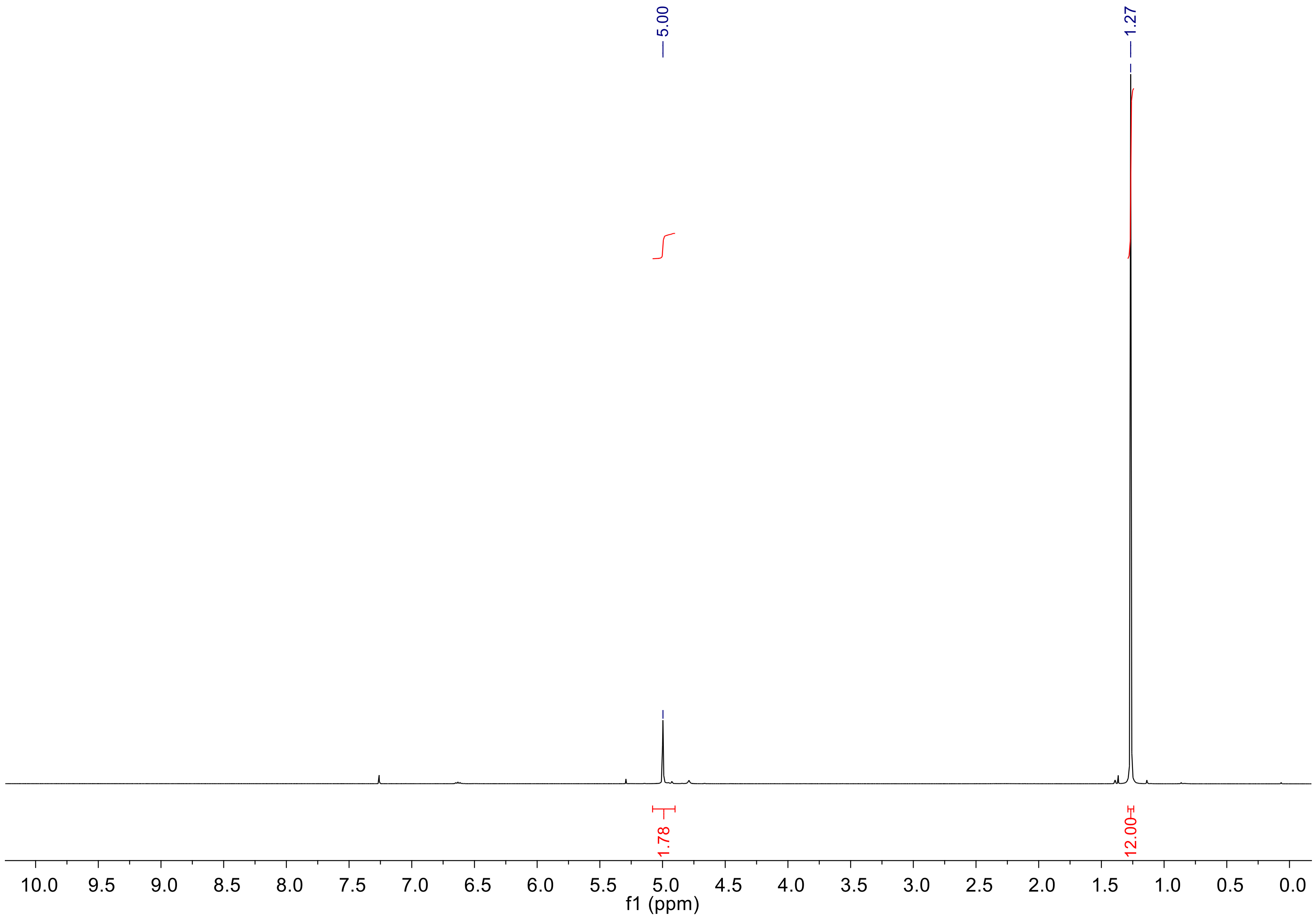

S115 ^{11}B NMR (160 MHz, $CDCl_3$, 298 K) spectrum of *4,4,5,5-tetramethyl-2-((perfluorophenyl)methoxy)-1,3,2-dioxaborolane* **2m**.

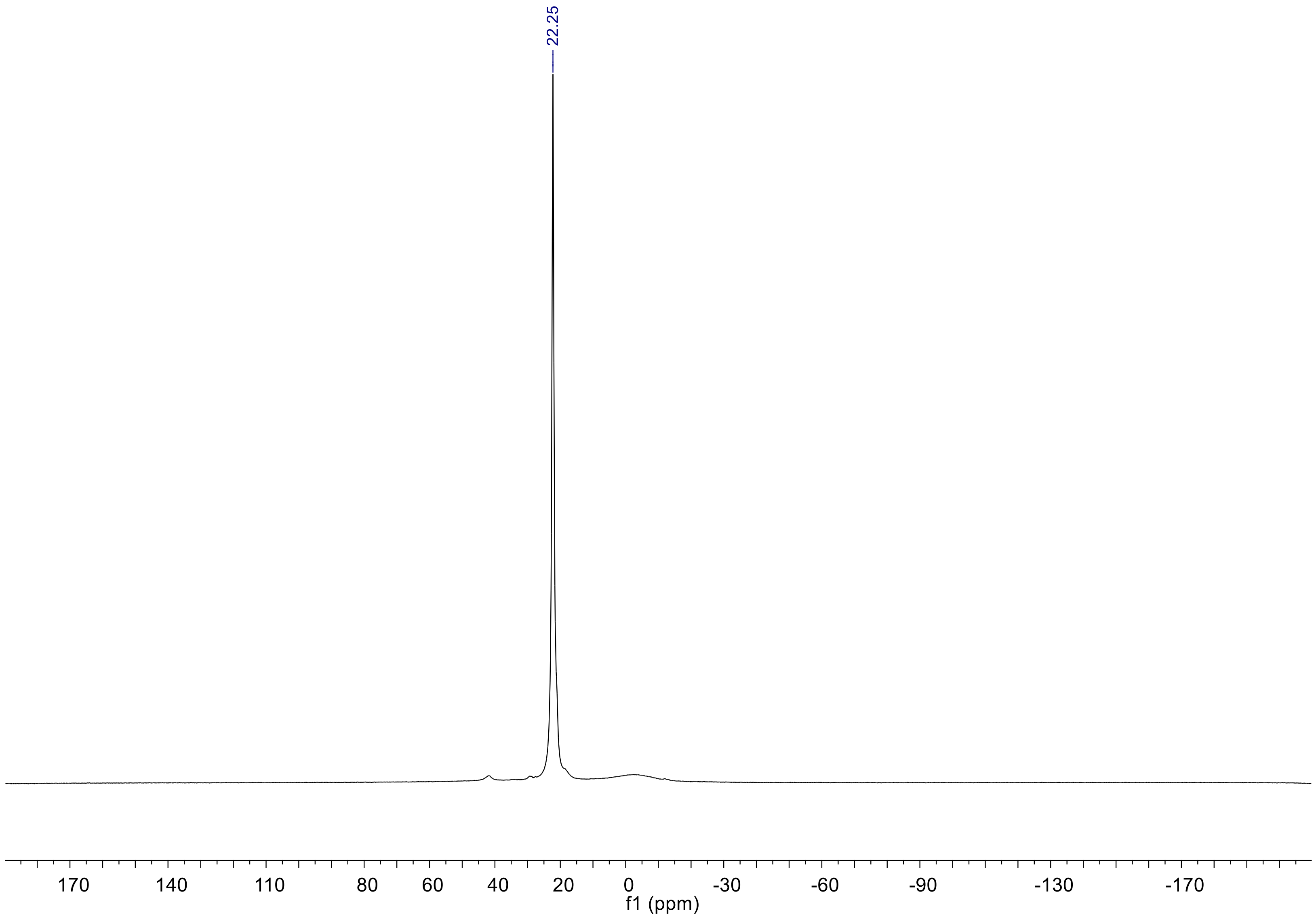

S116 ^{13}C NMR (126 MHz, $CDCl_3$, 298 K) spectrum of *4,4,5,5-tetramethyl-2-((perfluorophenyl)methoxy)-1,3,2-dioxaborolane* **2m**.

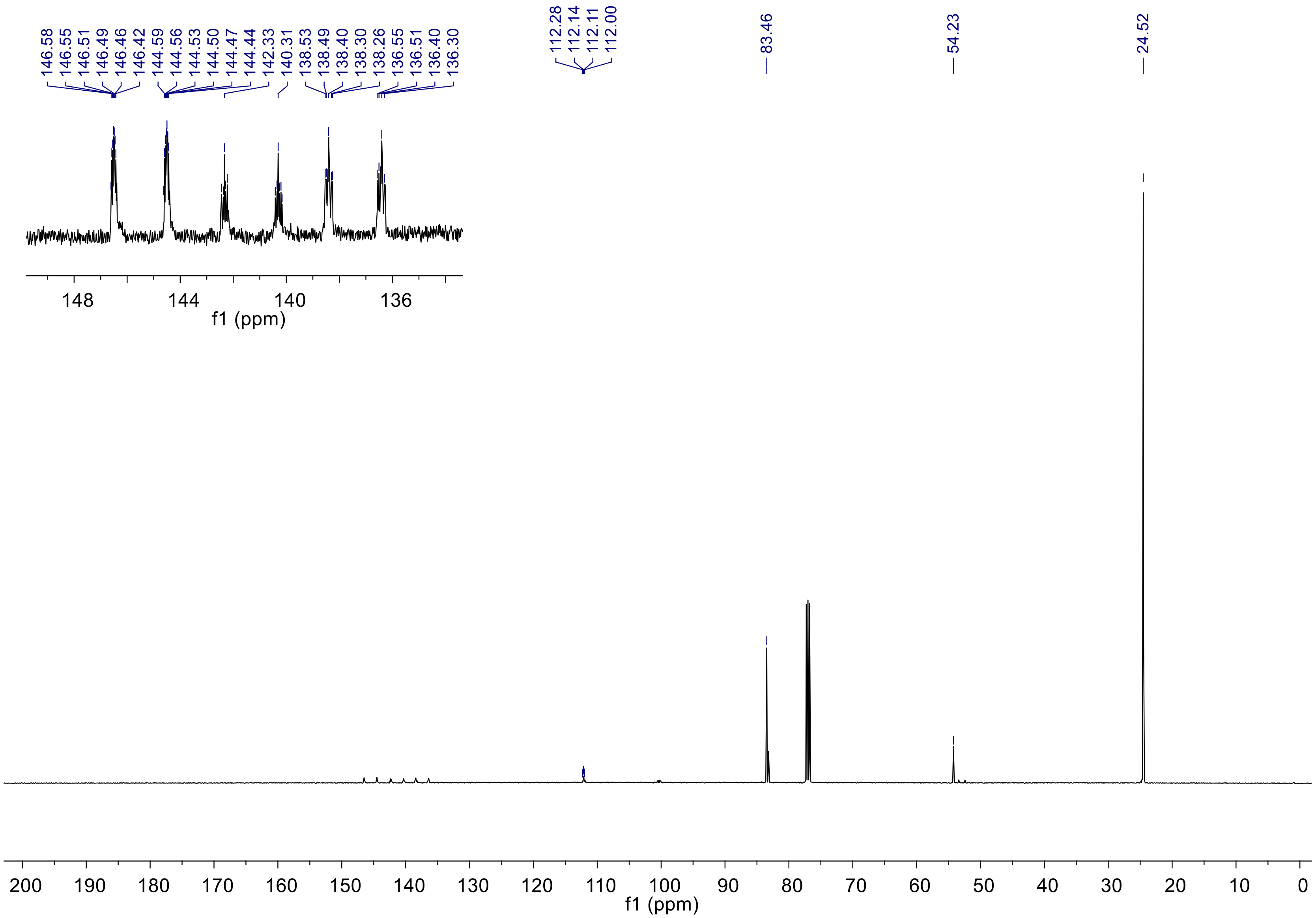

S117 ^{19}F NMR (565 MHz, $CDCl_3$, 298 K) spectrum of *4,4,5,5-tetramethyl-2-((perfluorophenyl)methoxy)-1,3,2-dioxaborolane* **2m**.

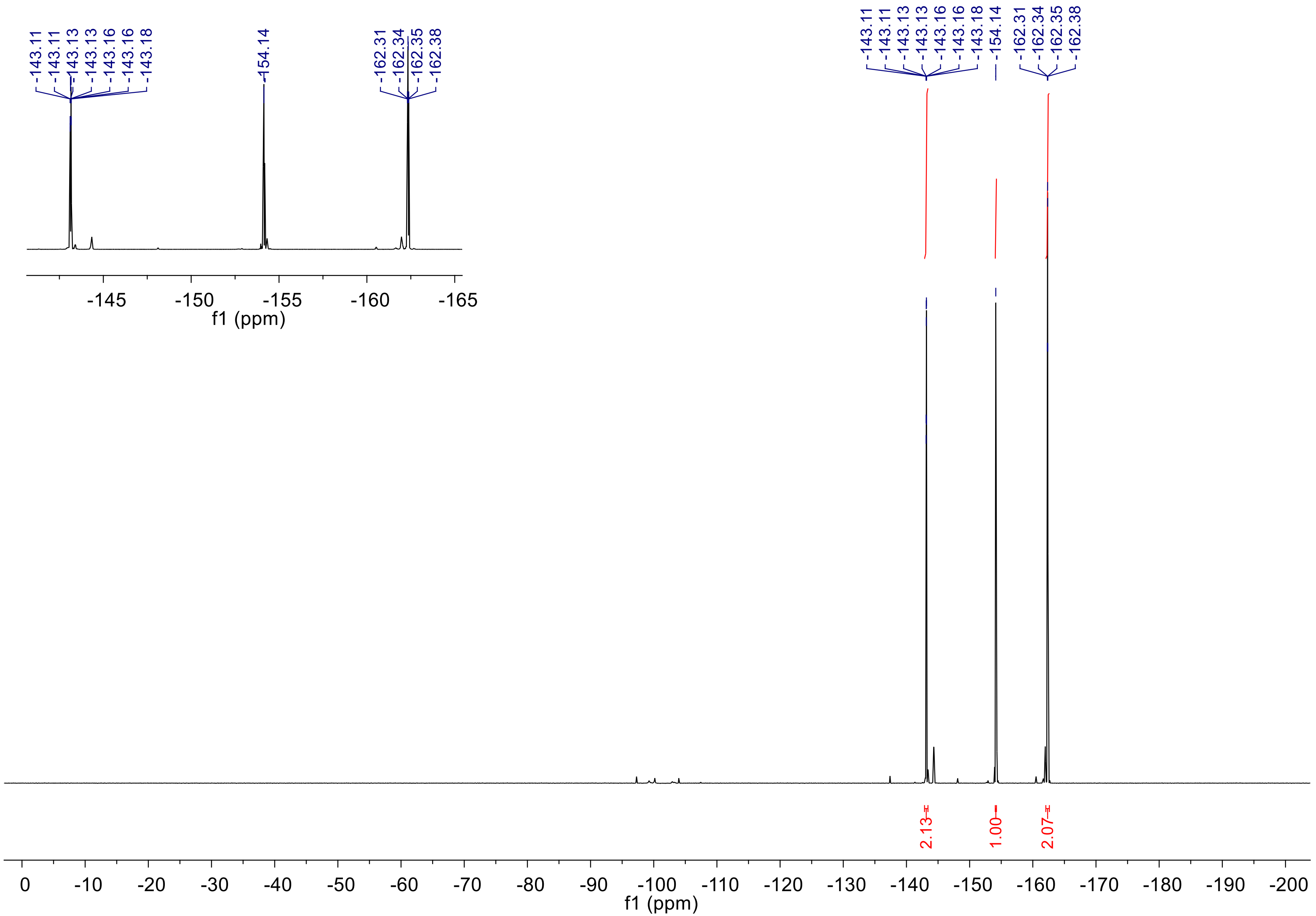

S118 ^{1}H NMR (500 MHz, $CDCl_3$, 298 K) spectrum of *4,4,5,5-tetramethyl-2-(naphthalen-2-ylmethoxy)-1,3,2-dioxaborolane* **2n**.

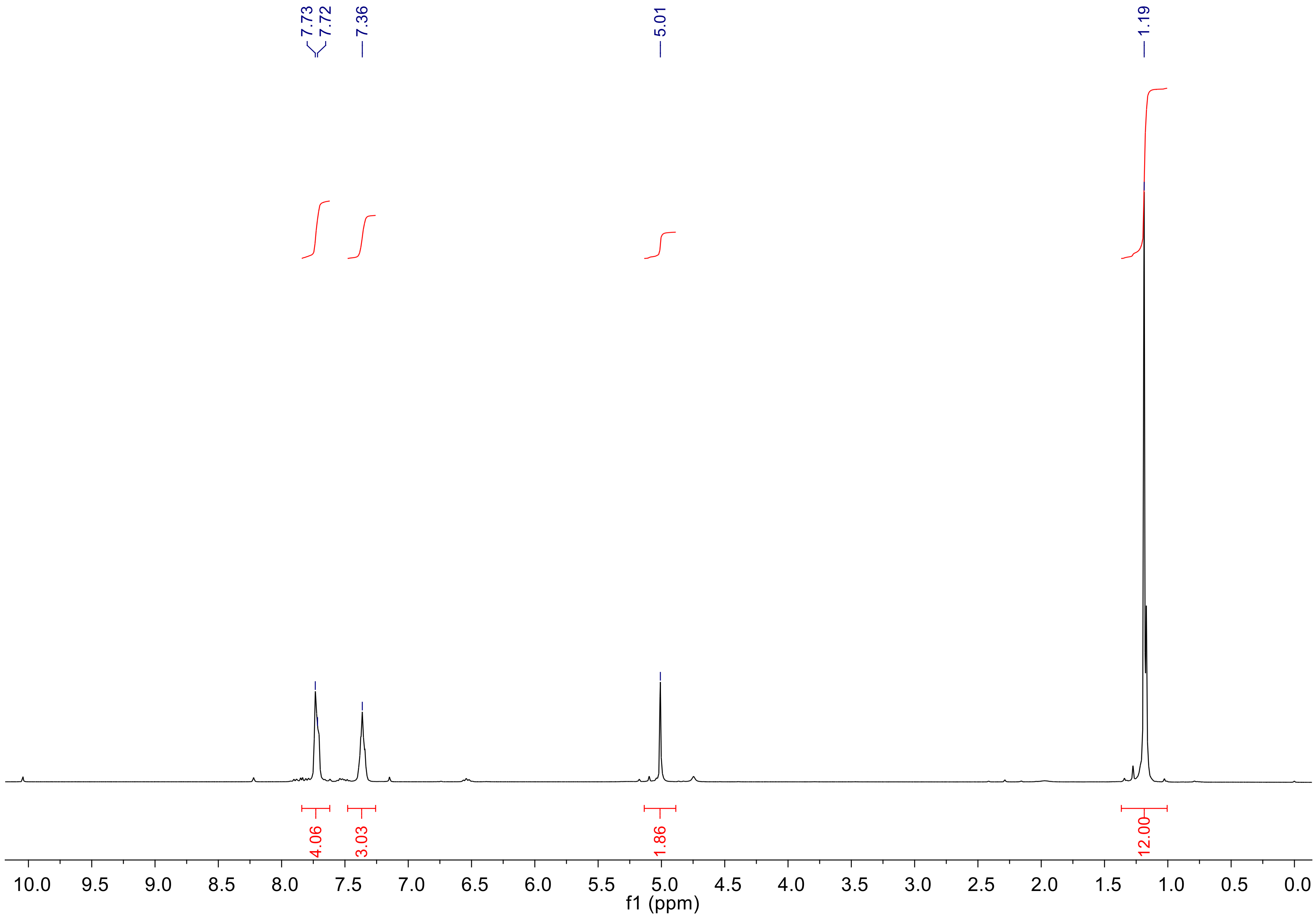

S119 ^{11}B NMR (160 MHz, $CDCl_3$, 298 K) spectrum of *4,4,5,5-tetramethyl-2-(naphthalen-2-ylmethoxy)-1,3,2-dioxaborolane* **2n**.

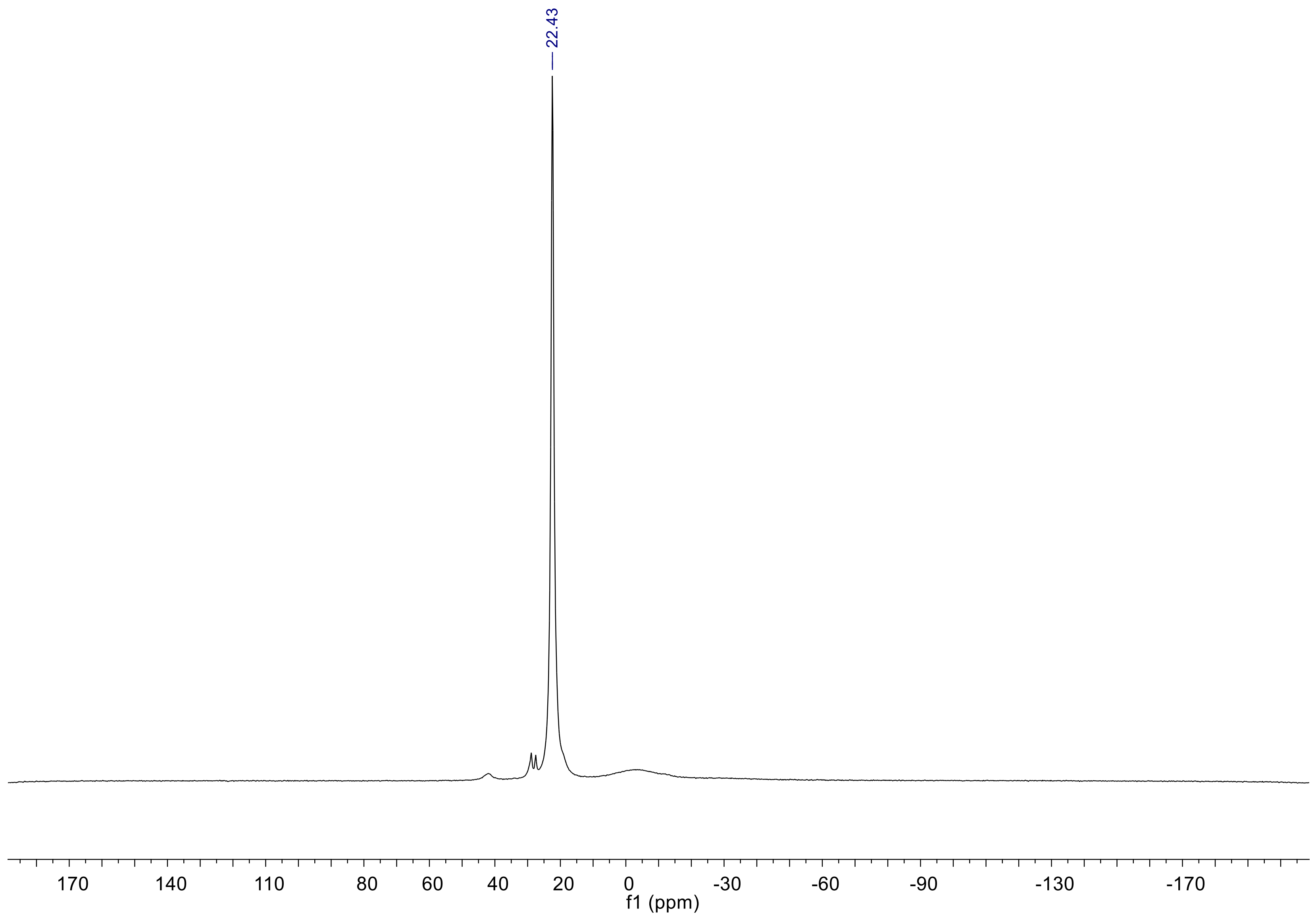

S120 ^{13}C NMR (126 MHz, $CDCl_3$, 298 K) spectrum of *4,4,5,5-tetramethyl-2-(naphthalen-2-ylmethoxy)-1,3,2-dioxaborolane* **2n**.

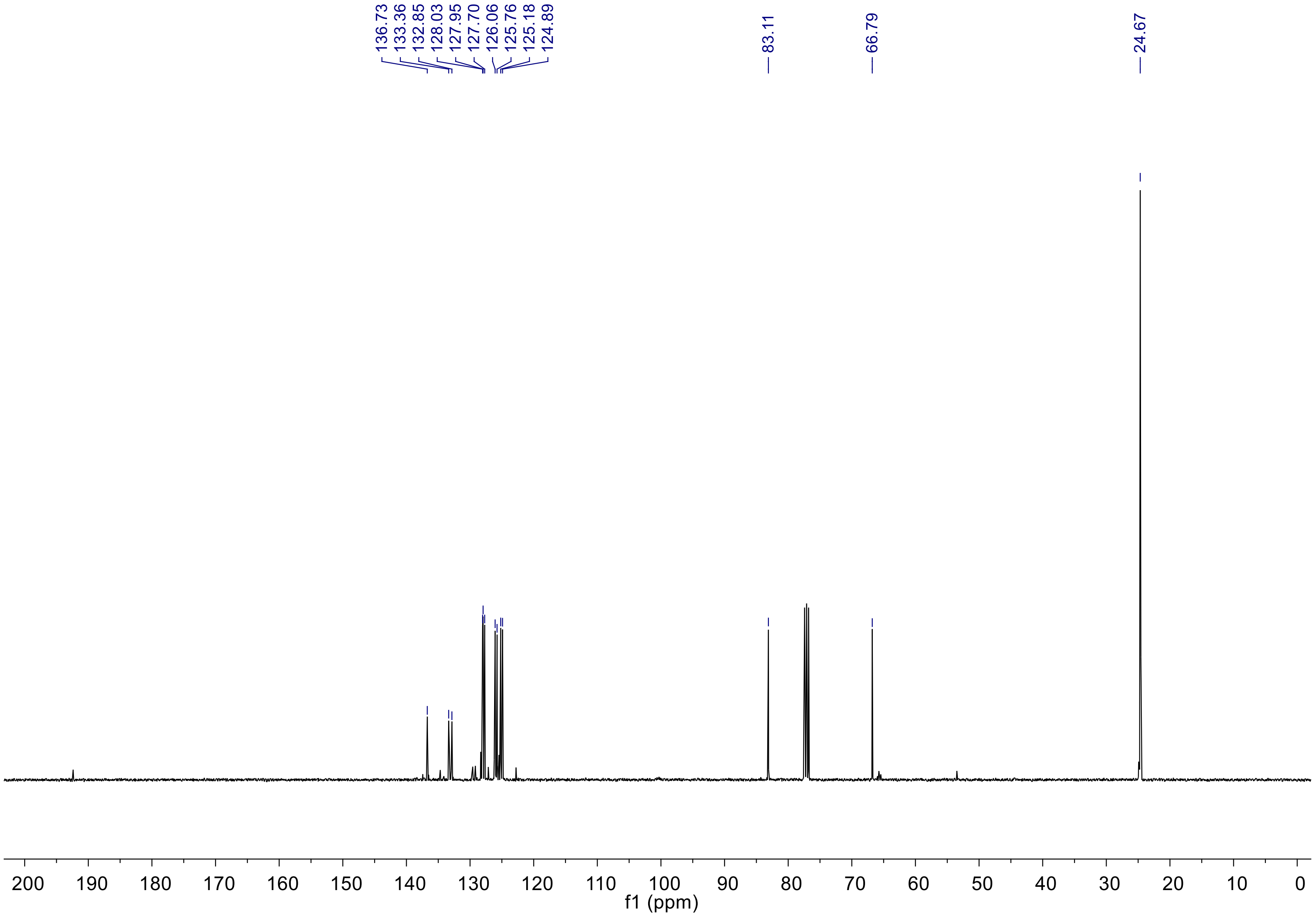

S121 ^{1}H NMR (500 MHz, $CDCl_3$, 298 K) spectrum of *3-(((4,4,5,5-tetramethyl-1,3,2-dioxaborolan-2-yl)oxy)methyl)pyridine* **2o**.

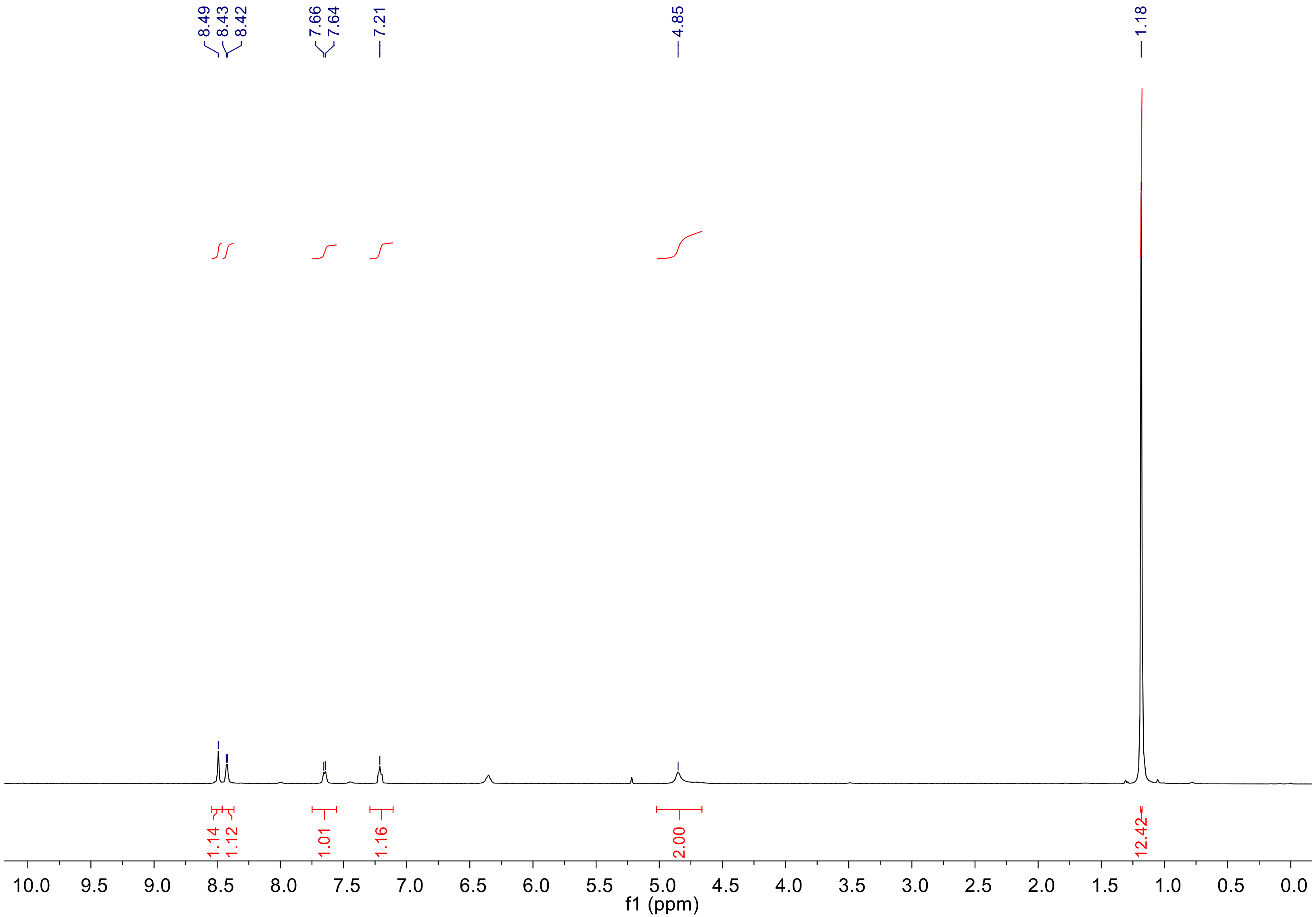

S122 ^{11}B NMR (160 MHz, $CDCl_3$, 298 K) spectrum of *3-(((4,4,5,5-tetramethyl-1,3,2-dioxaborolan-2-yl)oxy)methyl)pyridine* **2o**.

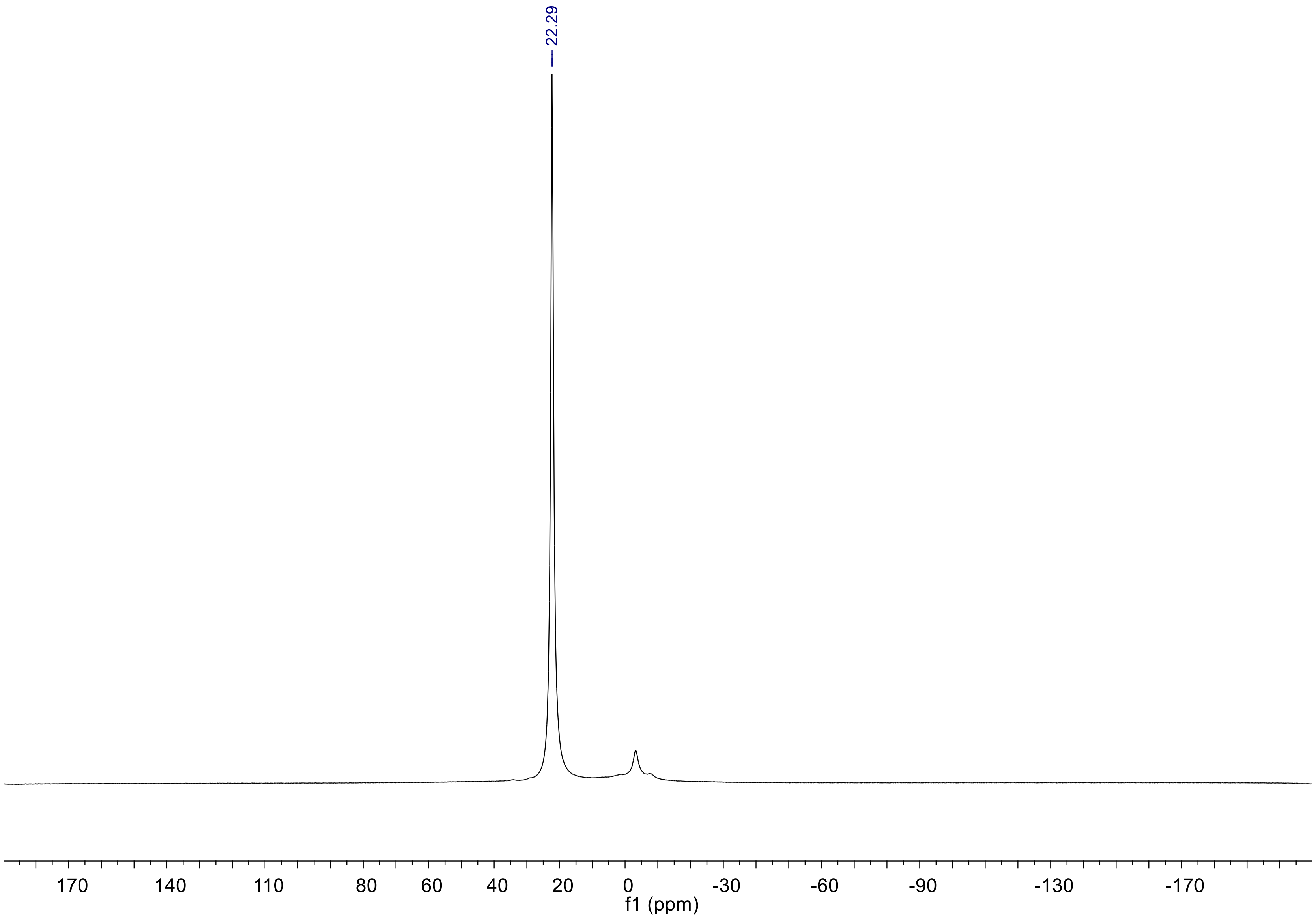

S123 ^{13}C NMR (126 MHz, $CDCl_3$, 298 K) spectrum of *3-(((4,4,5,5-tetramethyl-1,3,2-dioxaborolan-2-yl)oxy)methyl)pyridine* **2o**.

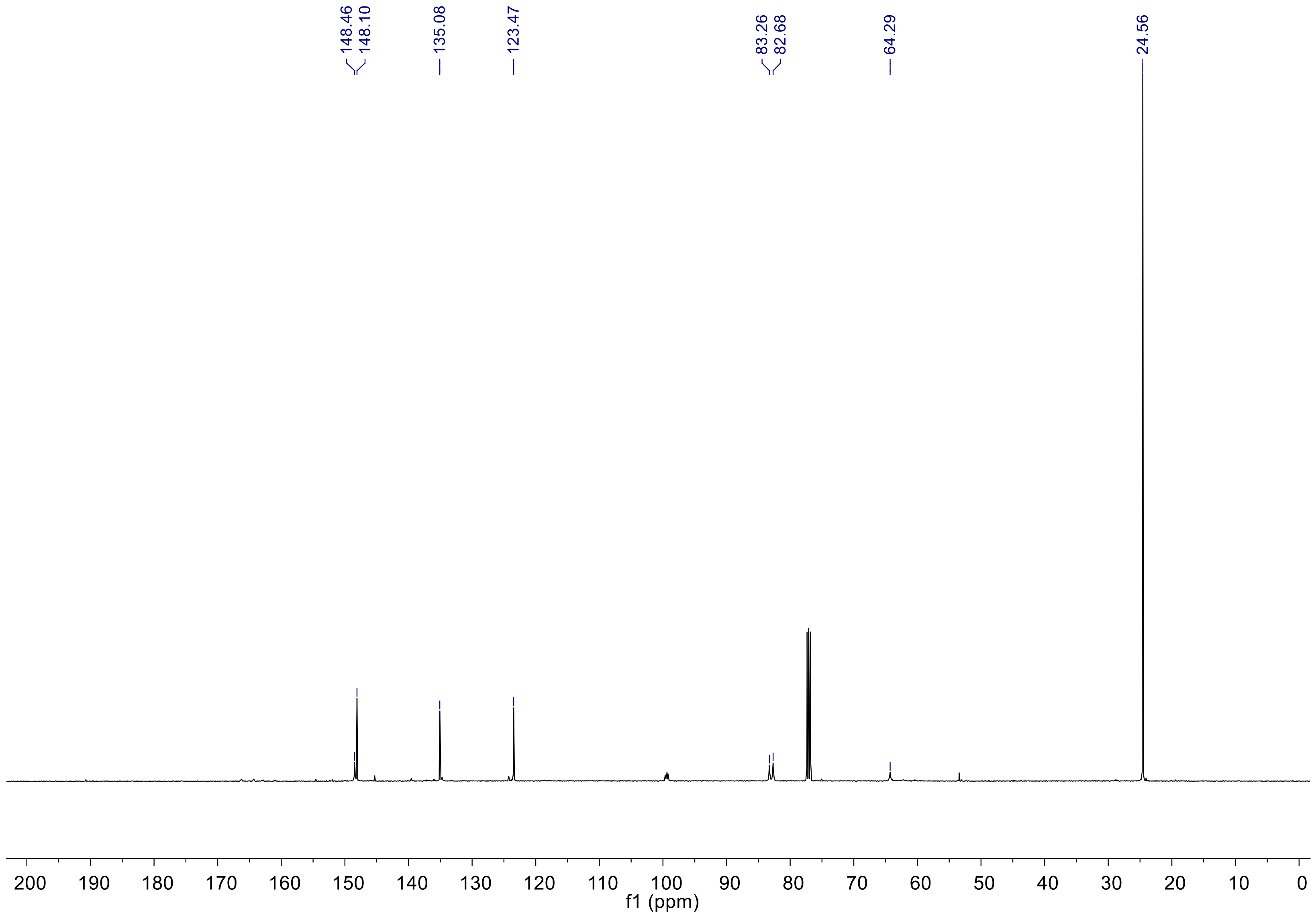

S124 ^{1}H NMR (500 MHz, $CDCl_3$, 298 K) spectrum of *2-(furan-2-ylmethoxy)-4,4,5,5-tetramethyl-1,3,2-dioxaborolane* **2p**.

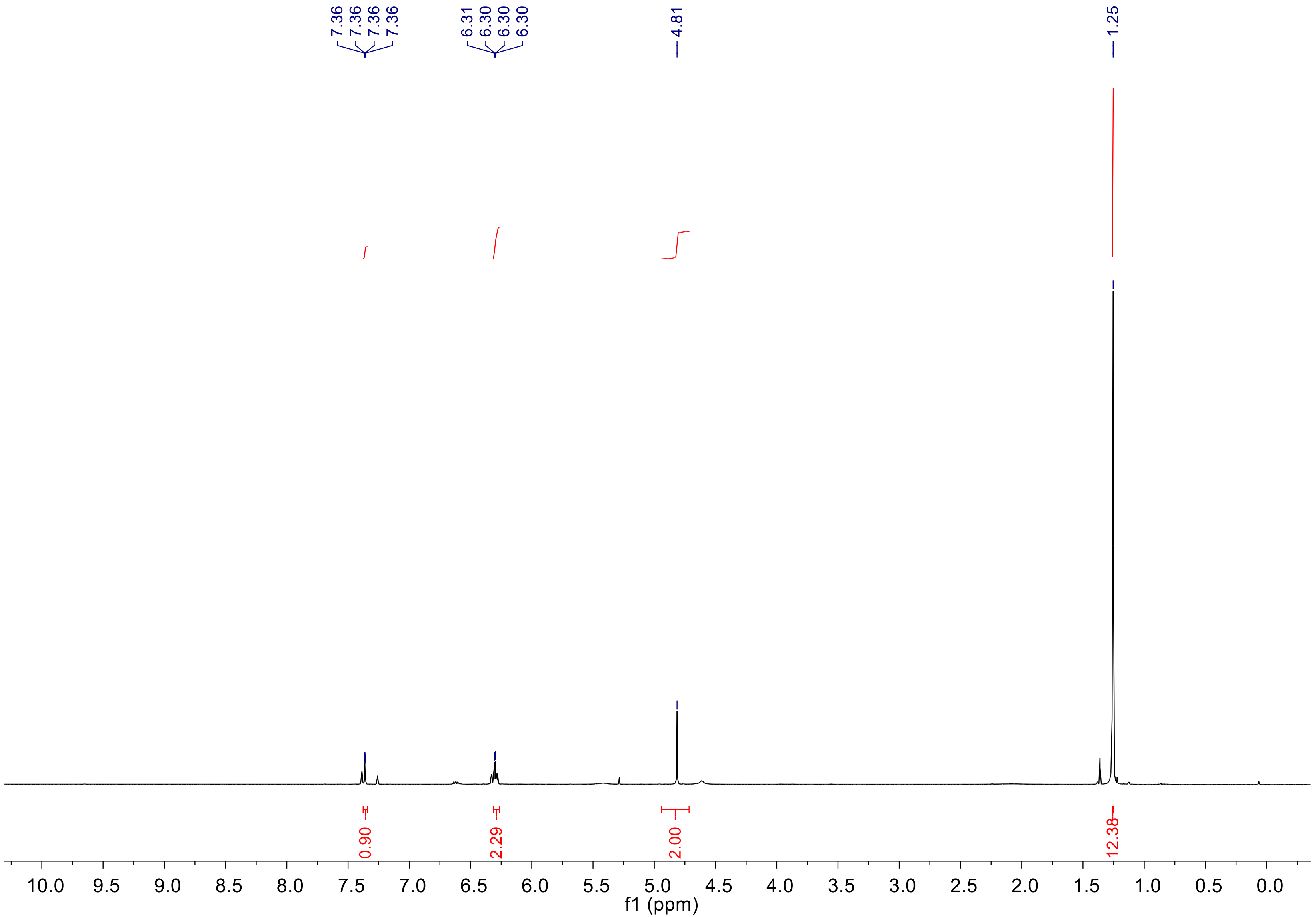

S125 ^{11}B NMR (160 MHz, $CDCl_3$, 298 K) spectrum of *2-(furan-2-ylmethoxy)-4,4,5,5-tetramethyl-1,3,2-dioxaborolane* **2p**.

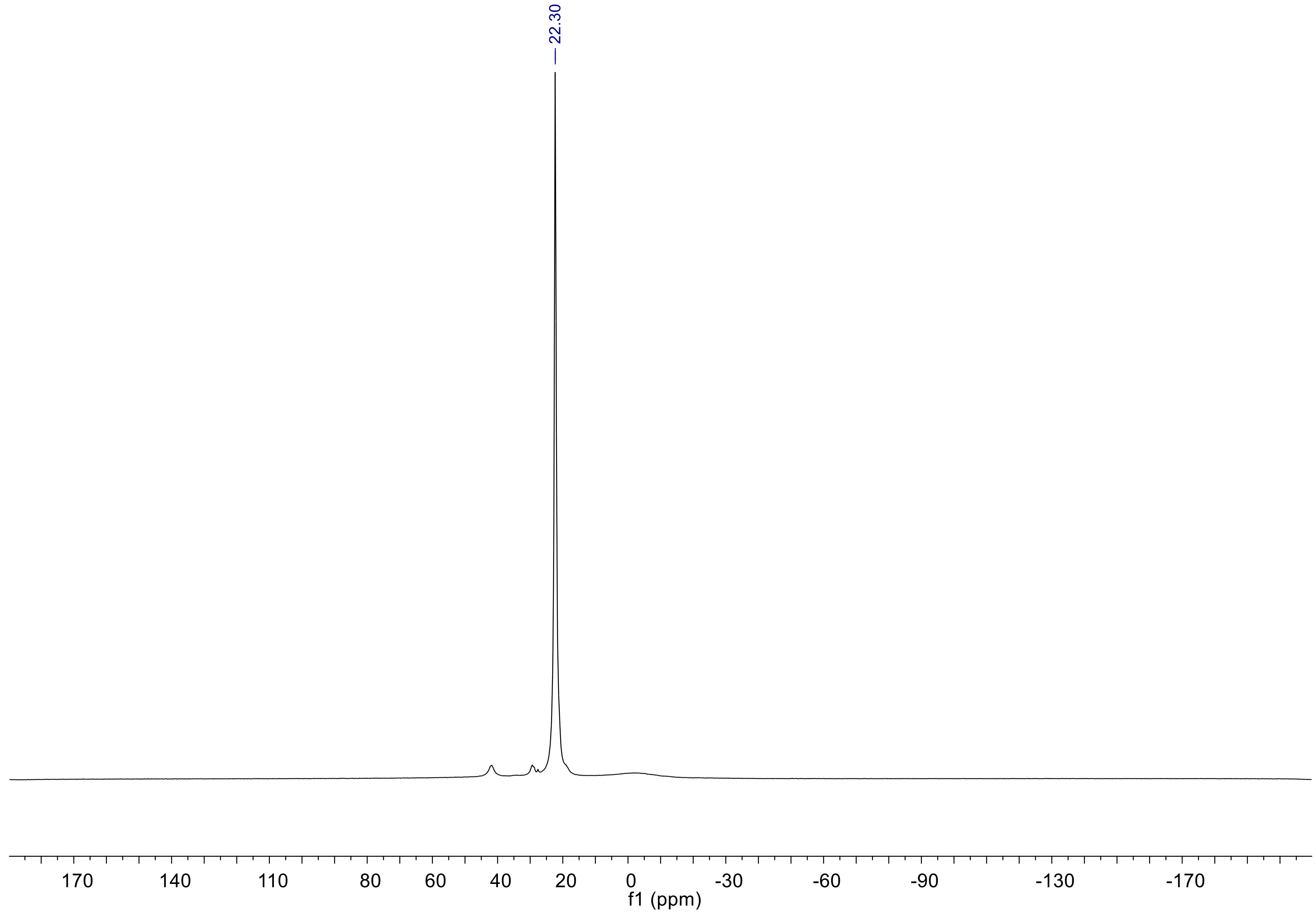

S126 ^{13}C NMR (126 MHz, $CDCl_3$, 298 K) spectrum of *2-(furan-2-ylmethoxy)-4,4,5,5-tetramethyl-1,3,2-dioxaborolane* **2p**.

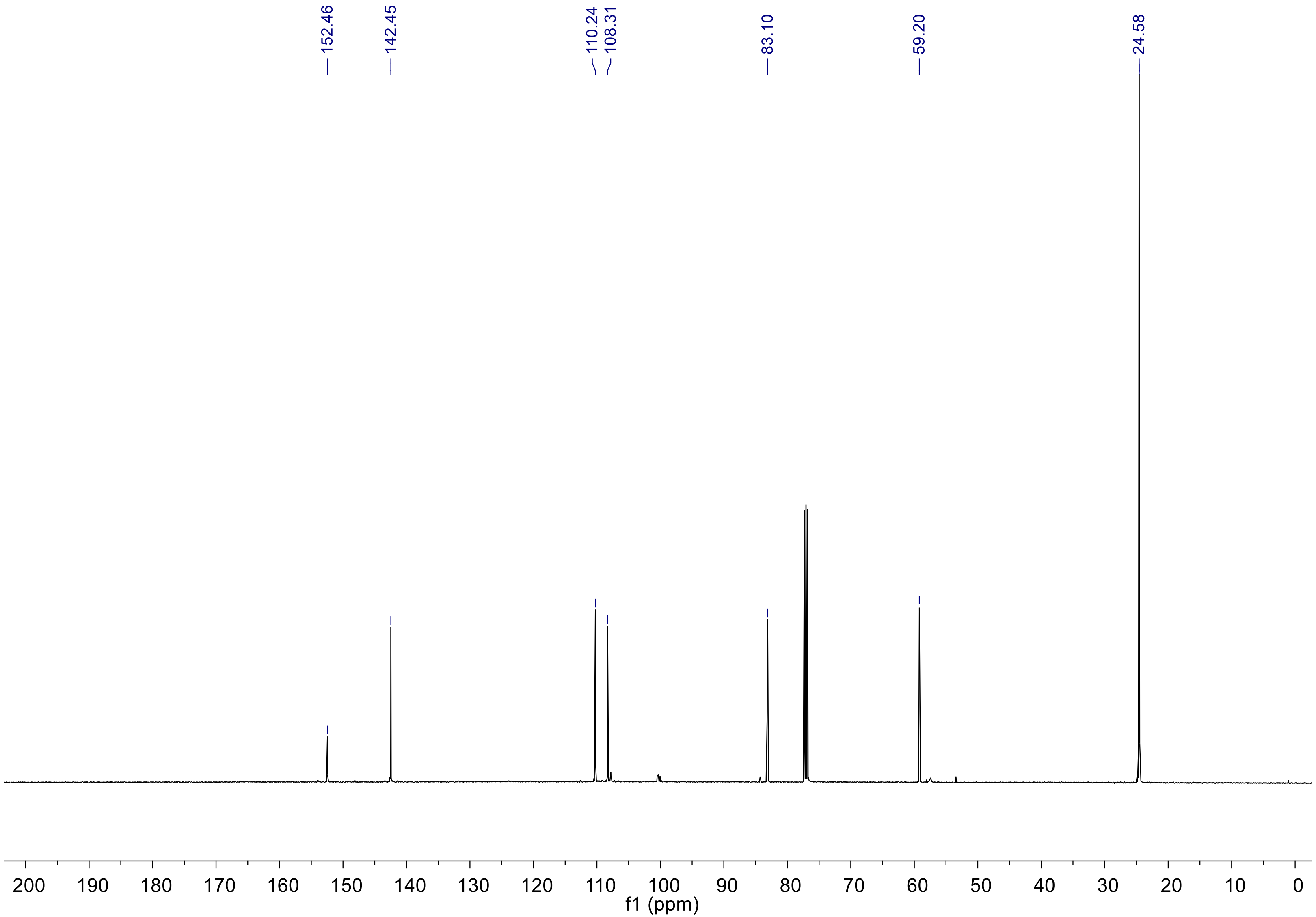

S127 ^{1}H NMR (400 MHz, $CDCl_3$, 298 K) spectrum of *2-ethoxy-4,4,5,5-tetramethyl-1,3,2-dioxaborolane* **2q**.

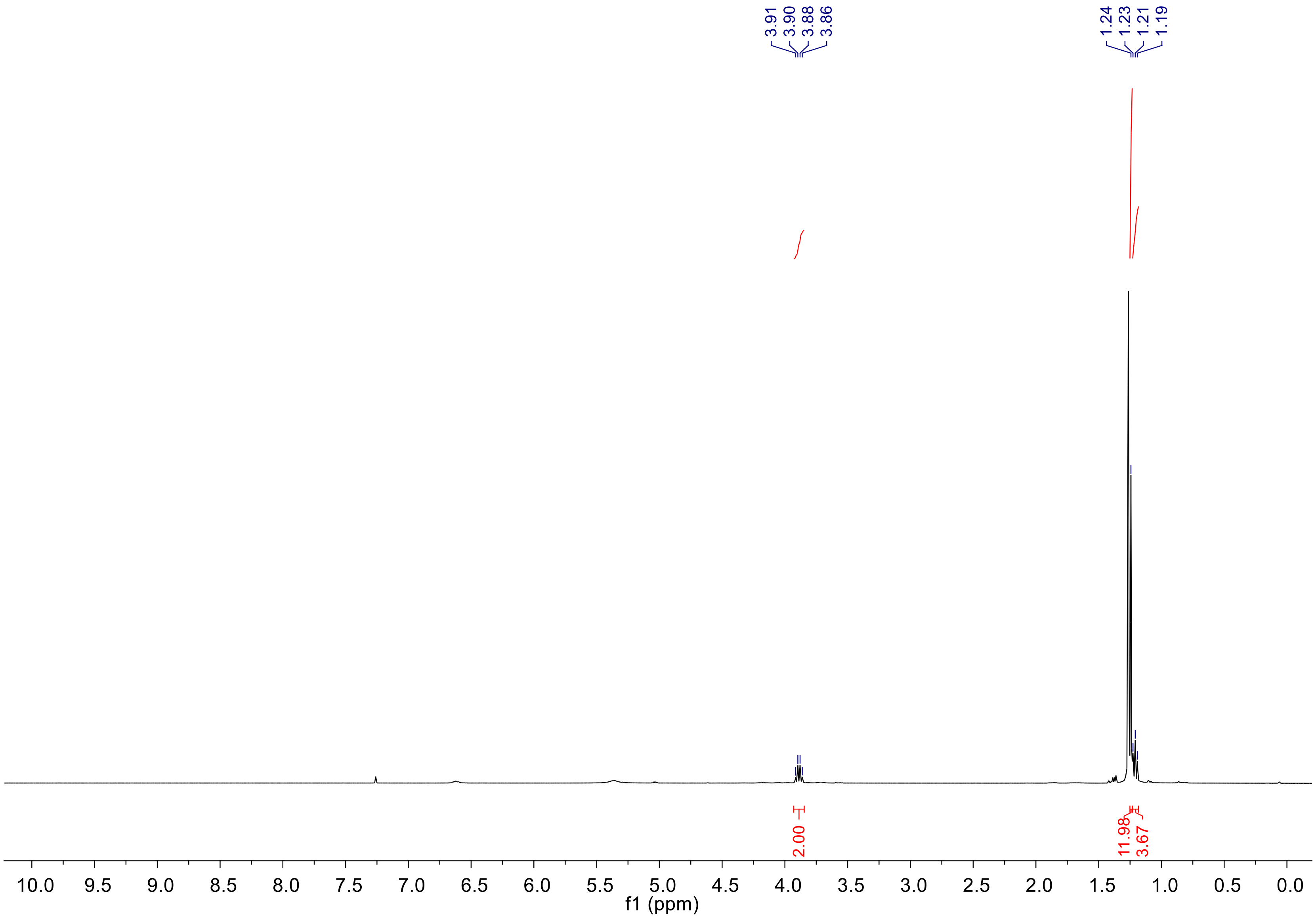

S128 ^{11}B NMR (128 MHz, $CDCl_3$, 298 K) spectrum of *2-ethoxy-4,4,5,5-tetramethyl-1,3,2-dioxaborolane* **2q**.

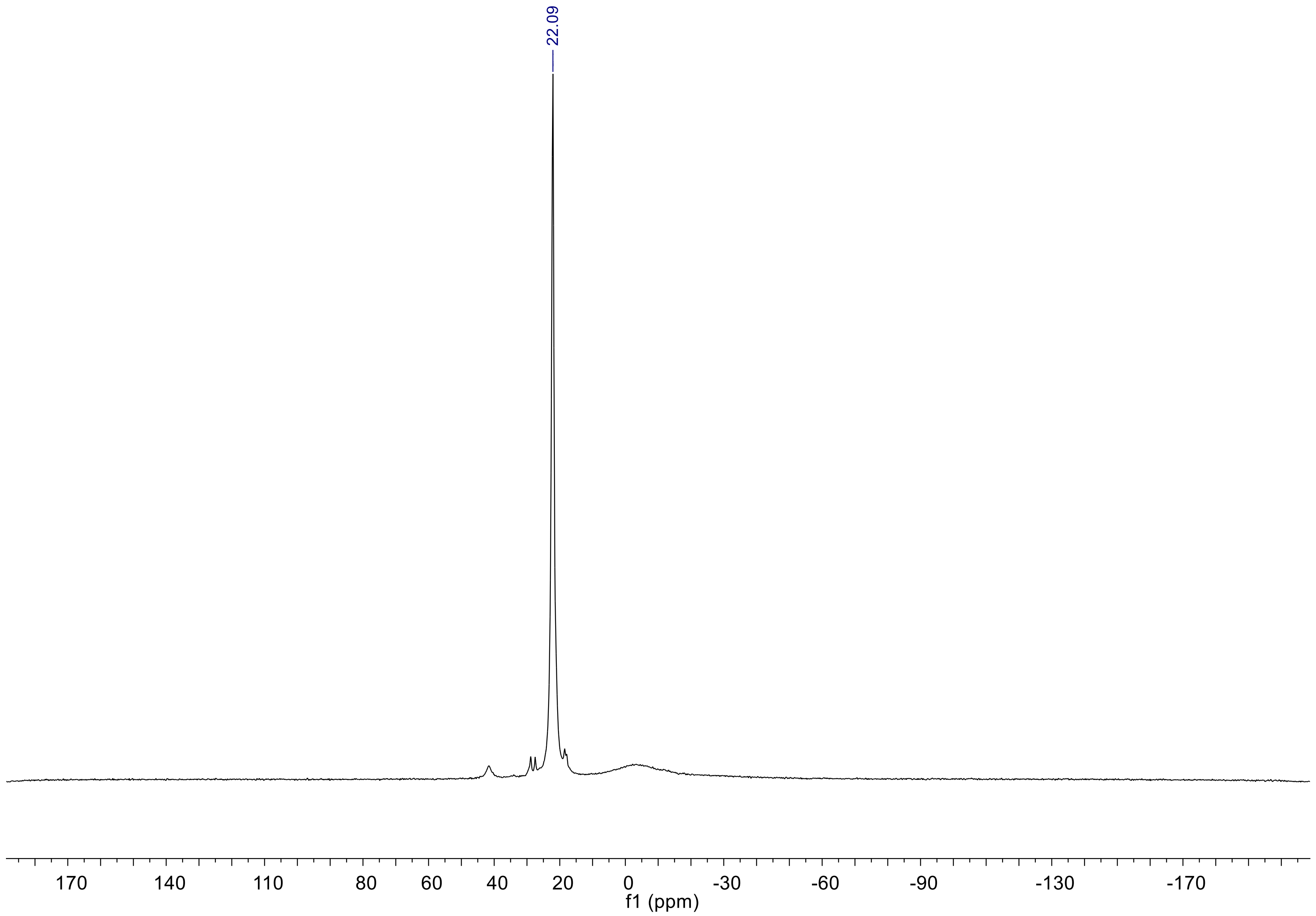

S129 ^{13}C NMR (101 MHz, $CDCl_3$, 298 K) spectrum of *2-ethoxy-4,4,5,5-tetramethyl-1,3,2-dioxaborolane* **2q**.

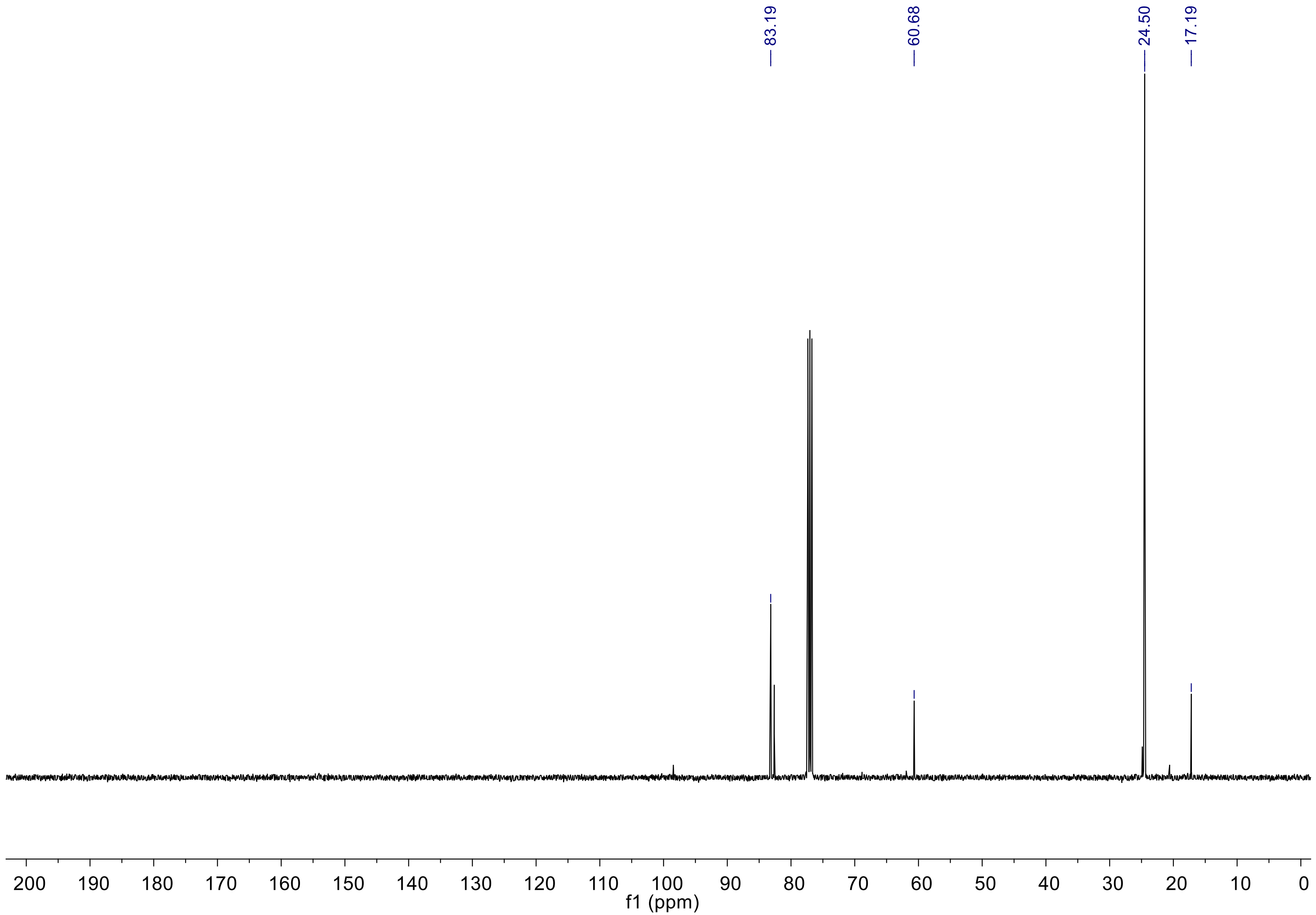

S130 ^{1}H NMR (400 MHz, $CDCl_3$, 298 K) spectrum of *4,4,5,5-tetramethyl-2-(pentyloxy)-1,3,2-dioxaborolane* **2r**.

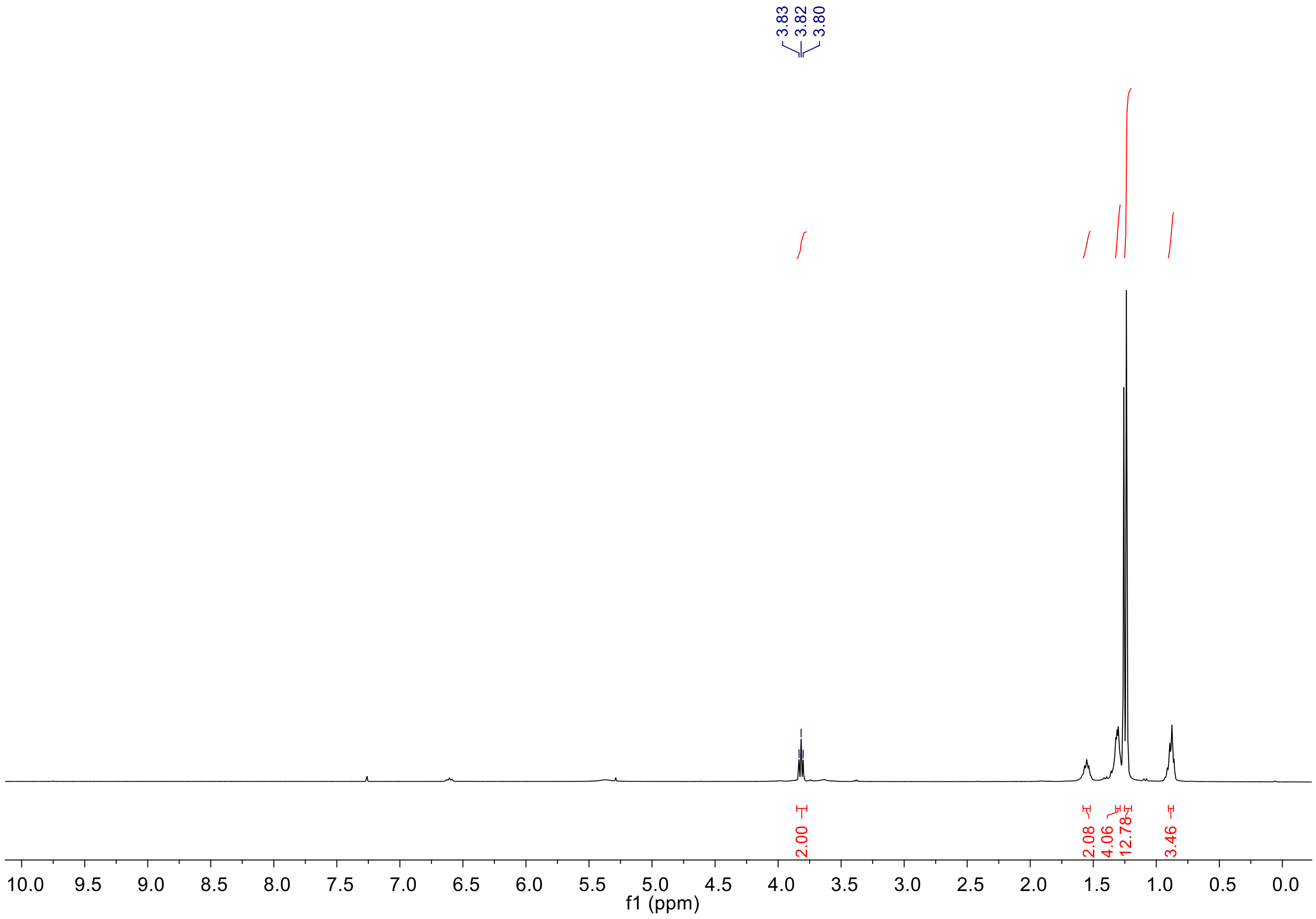

S131 ^{11}B NMR (128 MHz, $CDCl_3$, 298 K) spectrum of *4,4,5,5-tetramethyl-2-(pentyloxy)-1,3,2-dioxaborolane* **2r**.

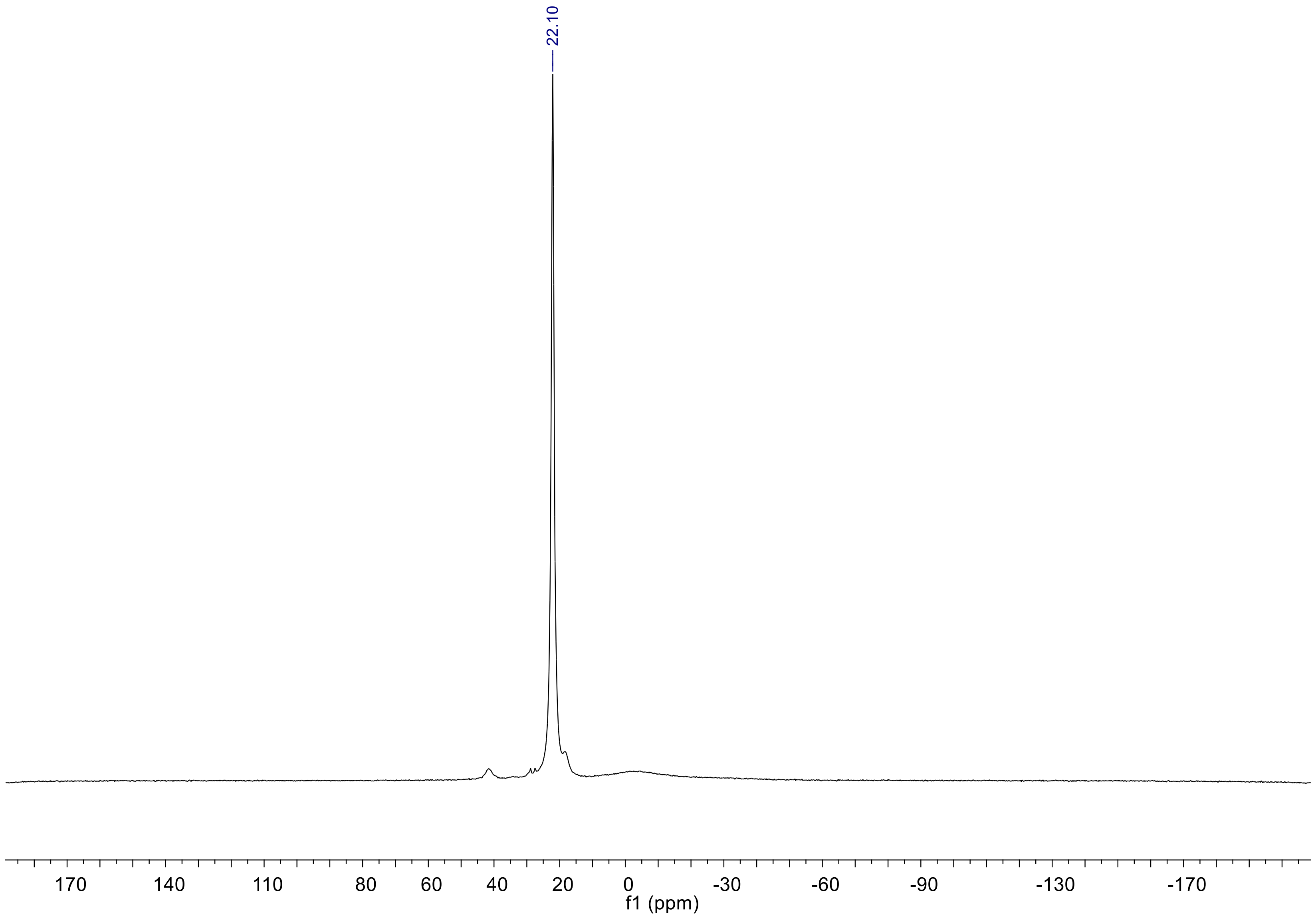

S132 ^{13}C NMR (101 MHz, $CDCl_3$, 298 K) spectrum of *4,4,5,5-tetramethyl-2-(pentyloxy)-1,3,2-dioxaborolane* **2r**.

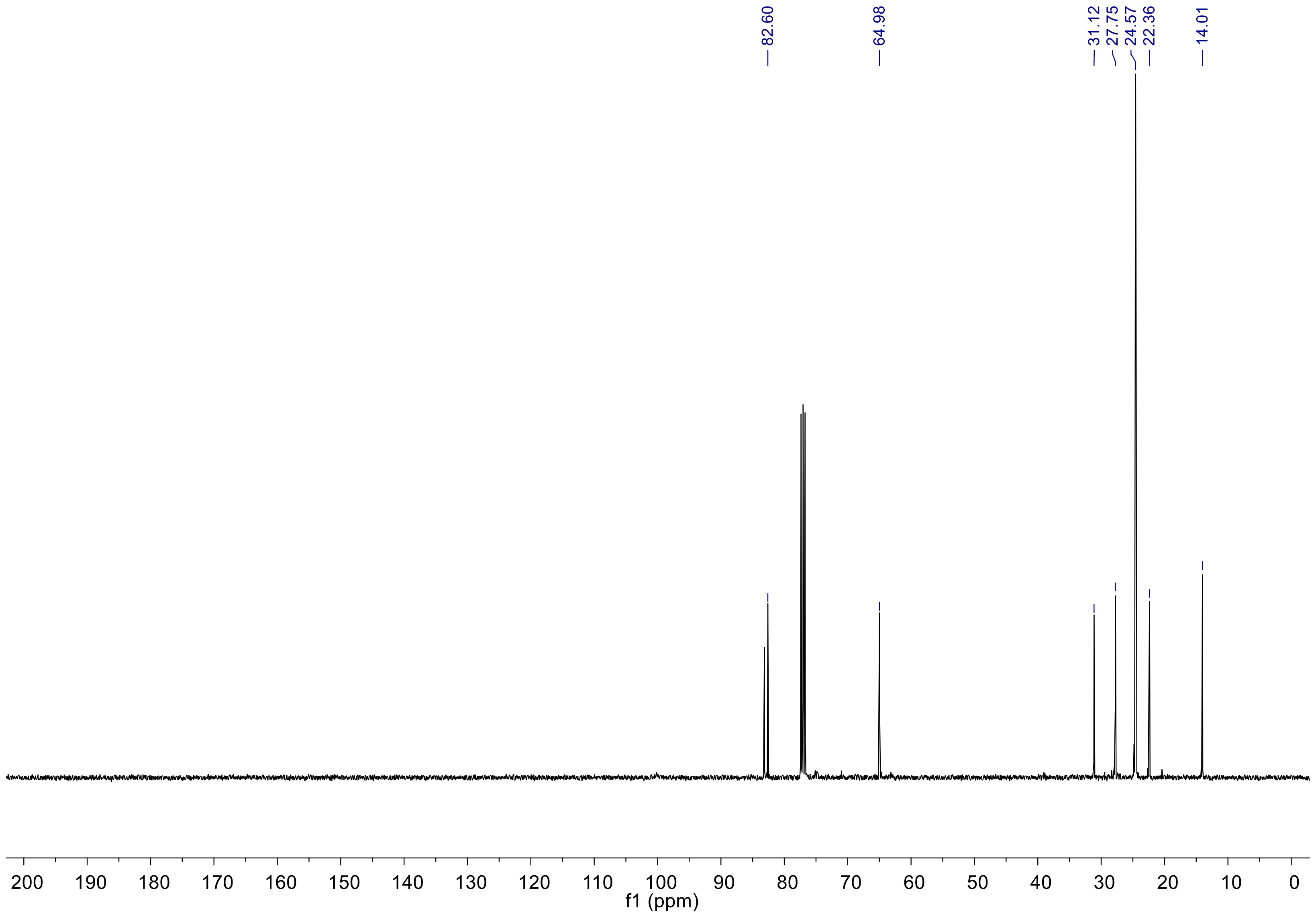

S133 ^{1}H NMR (400 MHz, $CDCl_3$, 298 K) spectrum of *4,4,5,5-tetramethyl-2-(neopentyloxy)-1,3,2-dioxaborolane* **2s**.

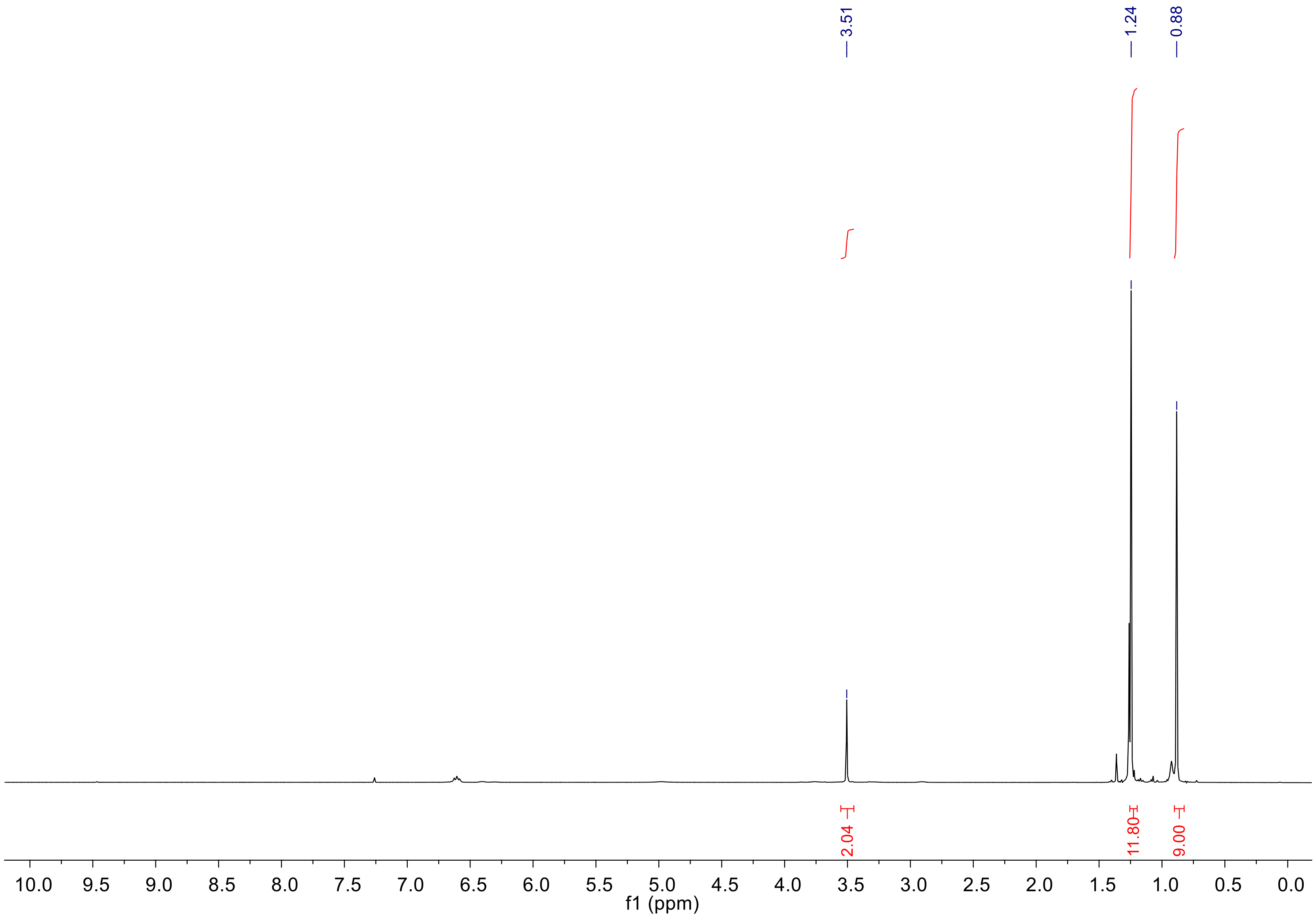

S134 ^{11}B NMR (128 MHz, $CDCl_3$, 298 K) spectrum of *4,4,5,5-tetramethyl-2-(neopentyloxy)-1,3,2-dioxaborolane* **2s**.

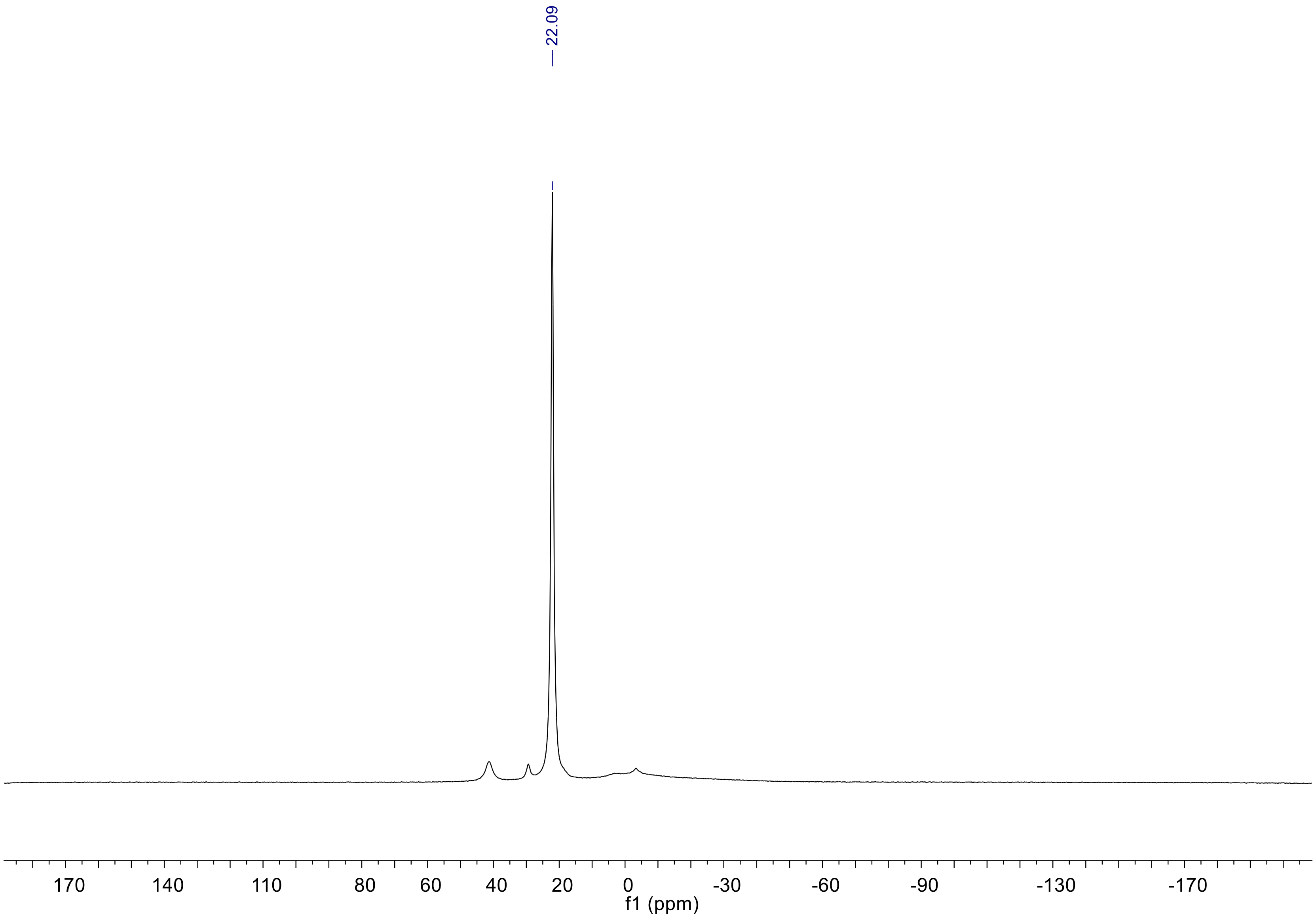

S135 ^{13}C NMR (101 MHz, $CDCl_3$, 298 K) spectrum of *4,4,5,5-tetramethyl-2-(neopentyloxy)-1,3,2-dioxaborolane* **2s**.

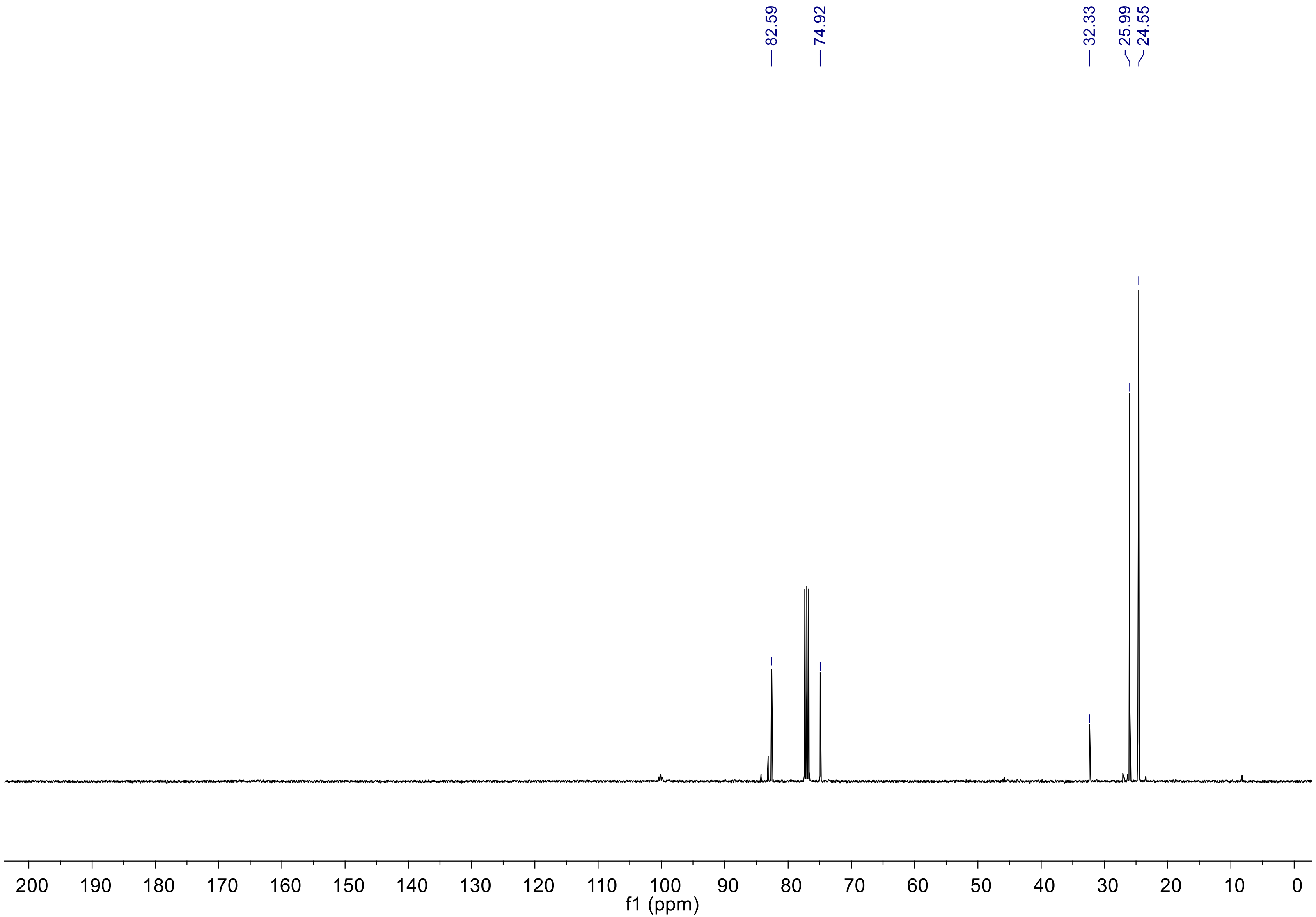

S136 ^{1}H NMR (500 MHz, $CDCl_3$, 298 K) spectrum of *2-(cyclohexylmethoxy)-4,4,5,5-tetramethyl-1,3,2-dioxaborolane* **2t**.

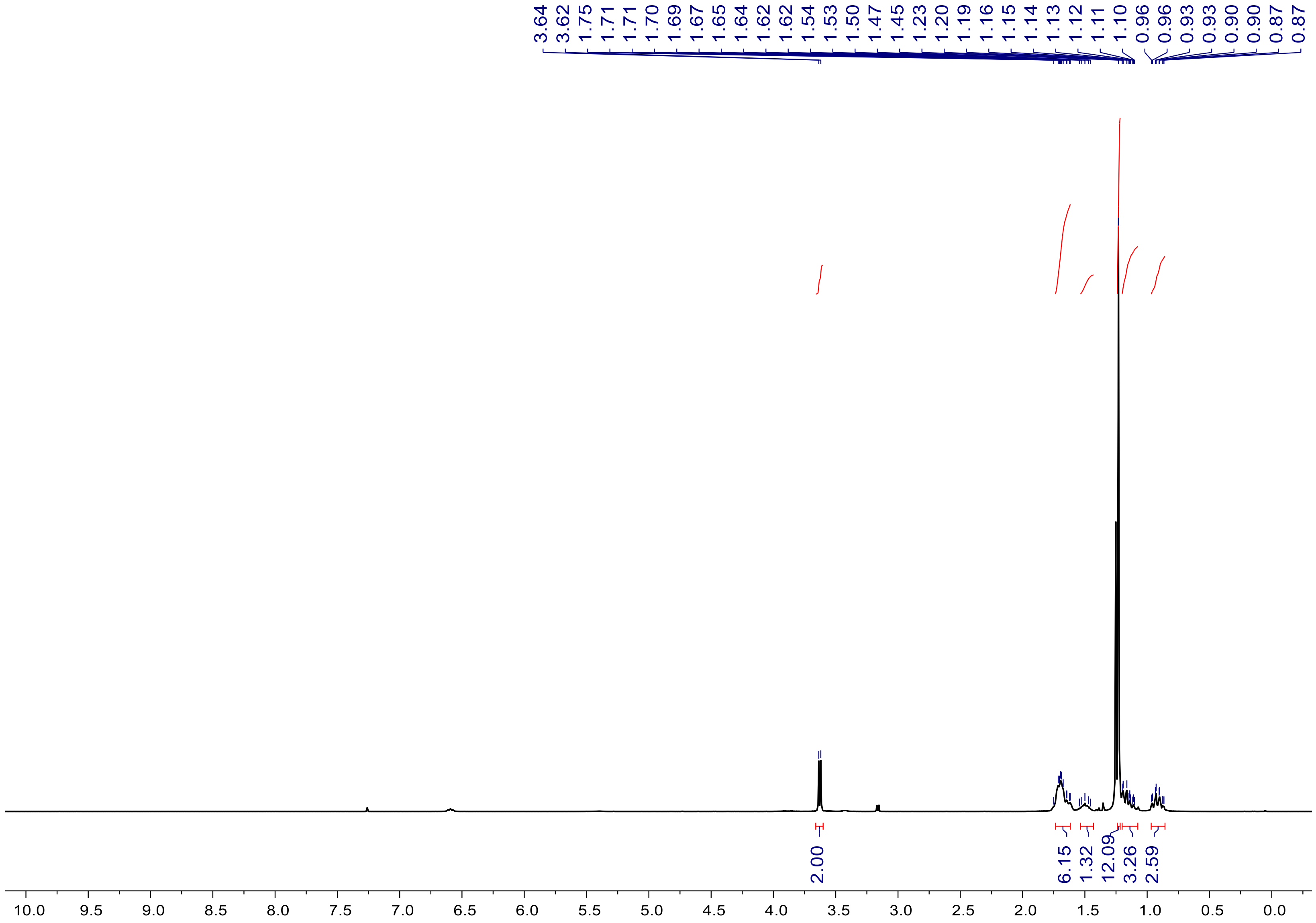

S137 ^{11}B NMR (160 MHz, $CDCl_3$, 298 K) spectrum of *2-(cyclohexylmethoxy)-4,4,5,5-tetramethyl-1,3,2-dioxaborolane* **2t**.

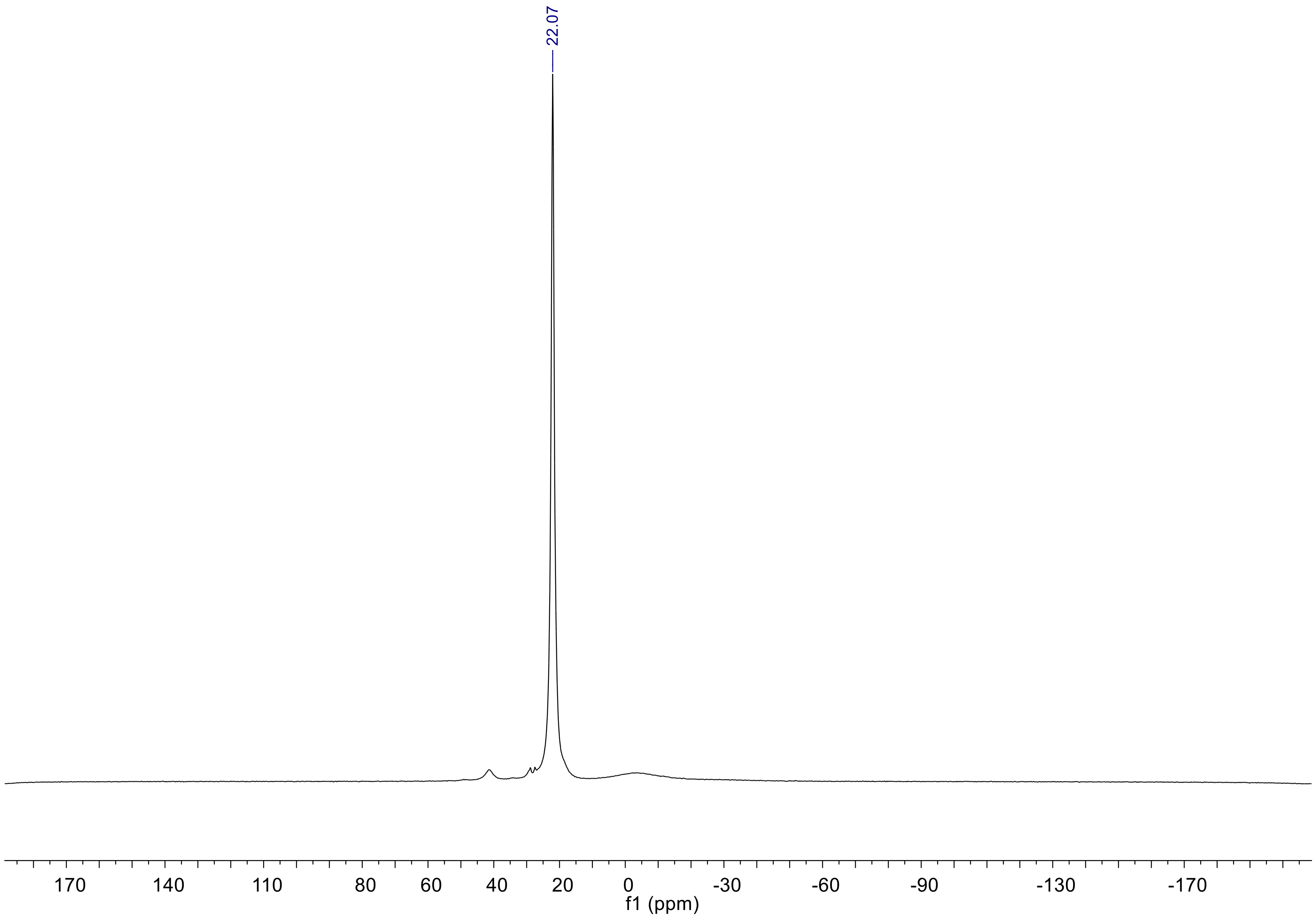

S138 ^{13}C NMR (126 MHz, $CDCl_3$, 298 K) spectrum of *2-(cyclohexylmethoxy)-4,4,5,5-tetramethyl-1,3,2-dioxaborolane* **2t**.

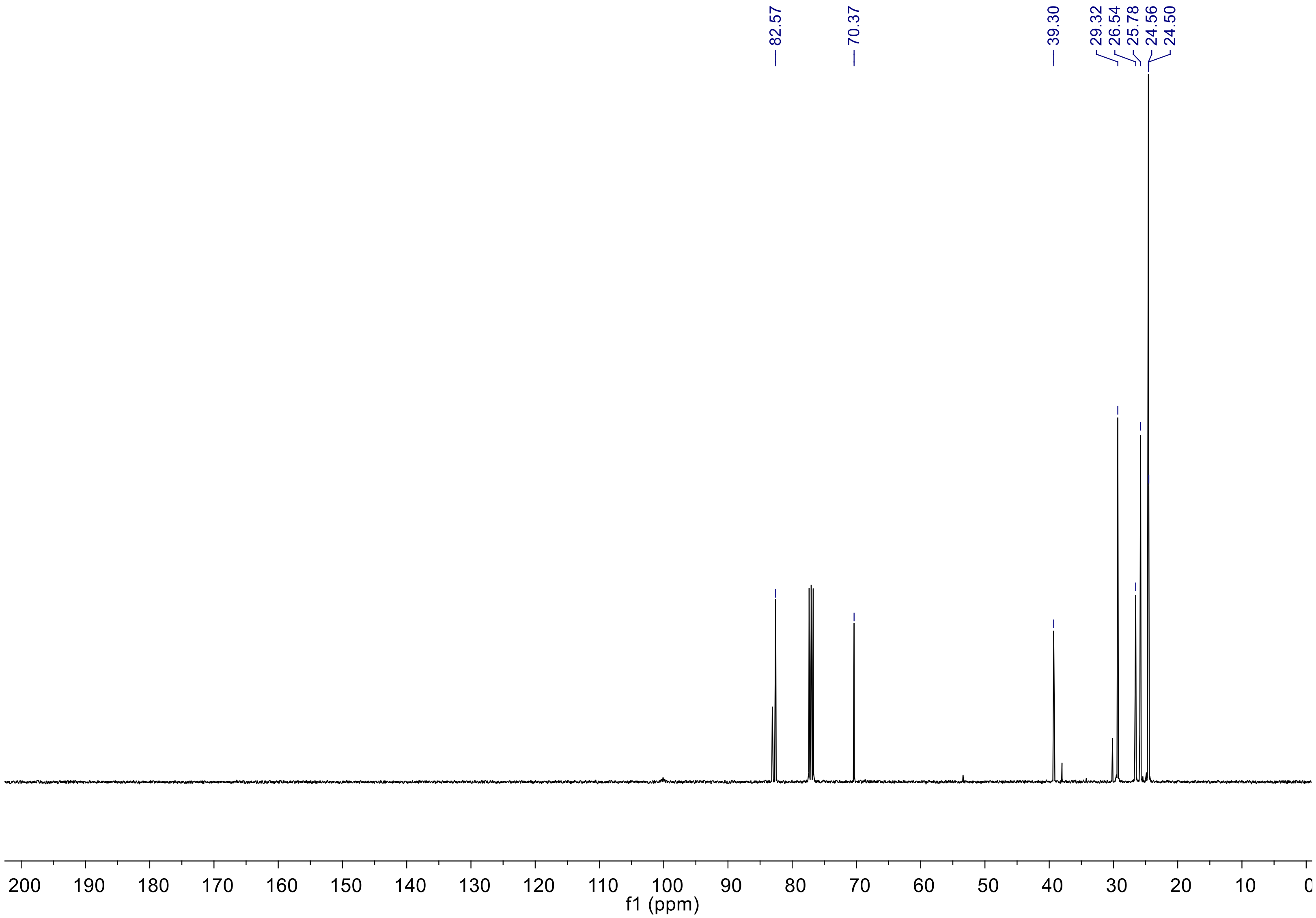

S139 ^{1}H NMR (500 MHz, $CDCl_3$, 298 K) spectrum of *N-benzyl-4,4,5,5-tetramethyl-N-phenyl-1,3,2-dioxaborolan-2-amine* **3a**.

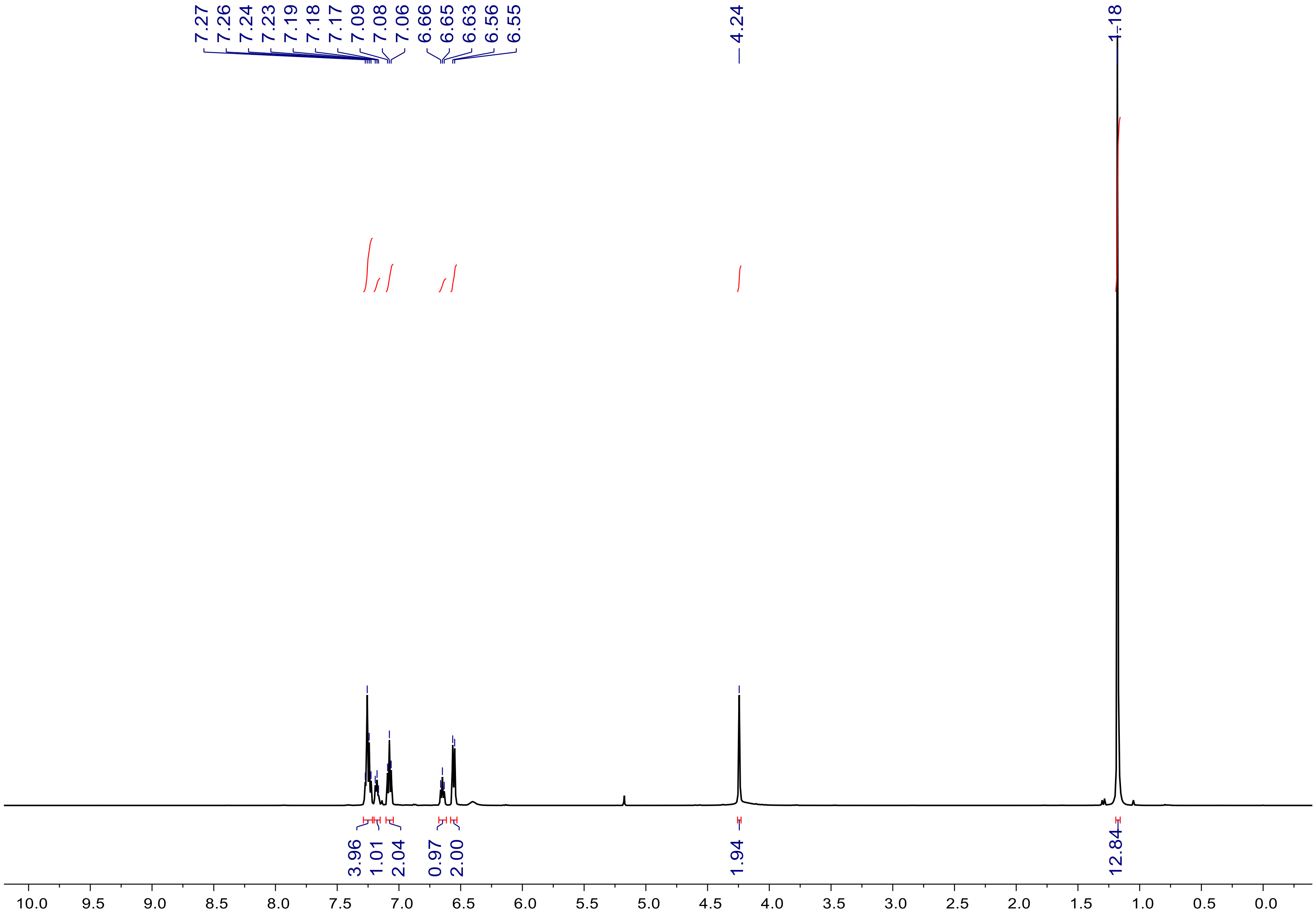

S140 ^{11}B NMR (160 MHz, $CDCl_3$, 298 K) spectrum of *N-benzyl-4,4,5,5-tetramethyl-N-phenyl-1,3,2-dioxaborolan-2-amine* **3a**.

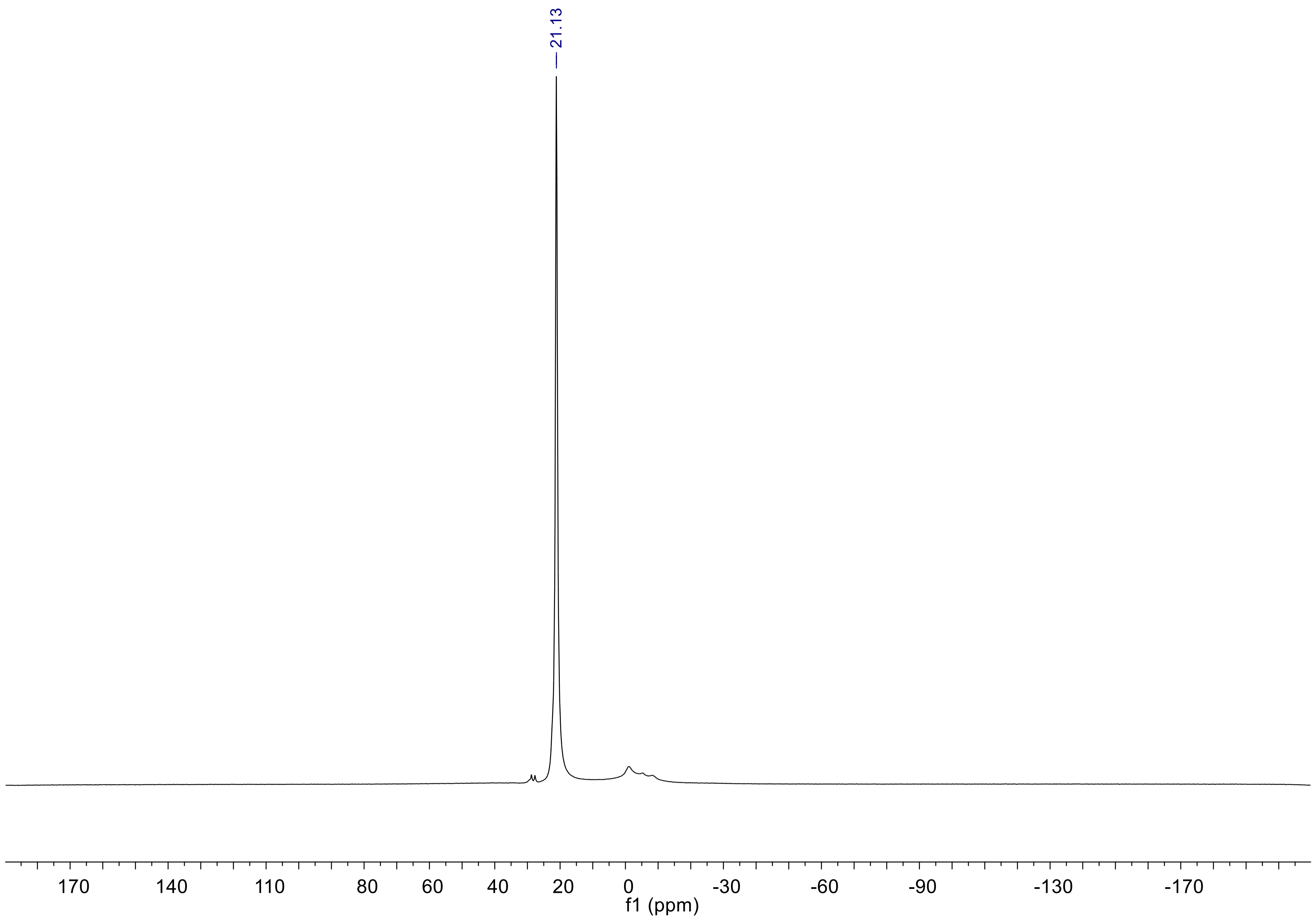

S141 ^{13}C NMR (126 MHz, $CDCl_3$, 298 K) spectrum of *N-benzyl-4,4,5,5-tetramethyl-N-phenyl-1,3,2-dioxaborolan-2-amine* **3a**.

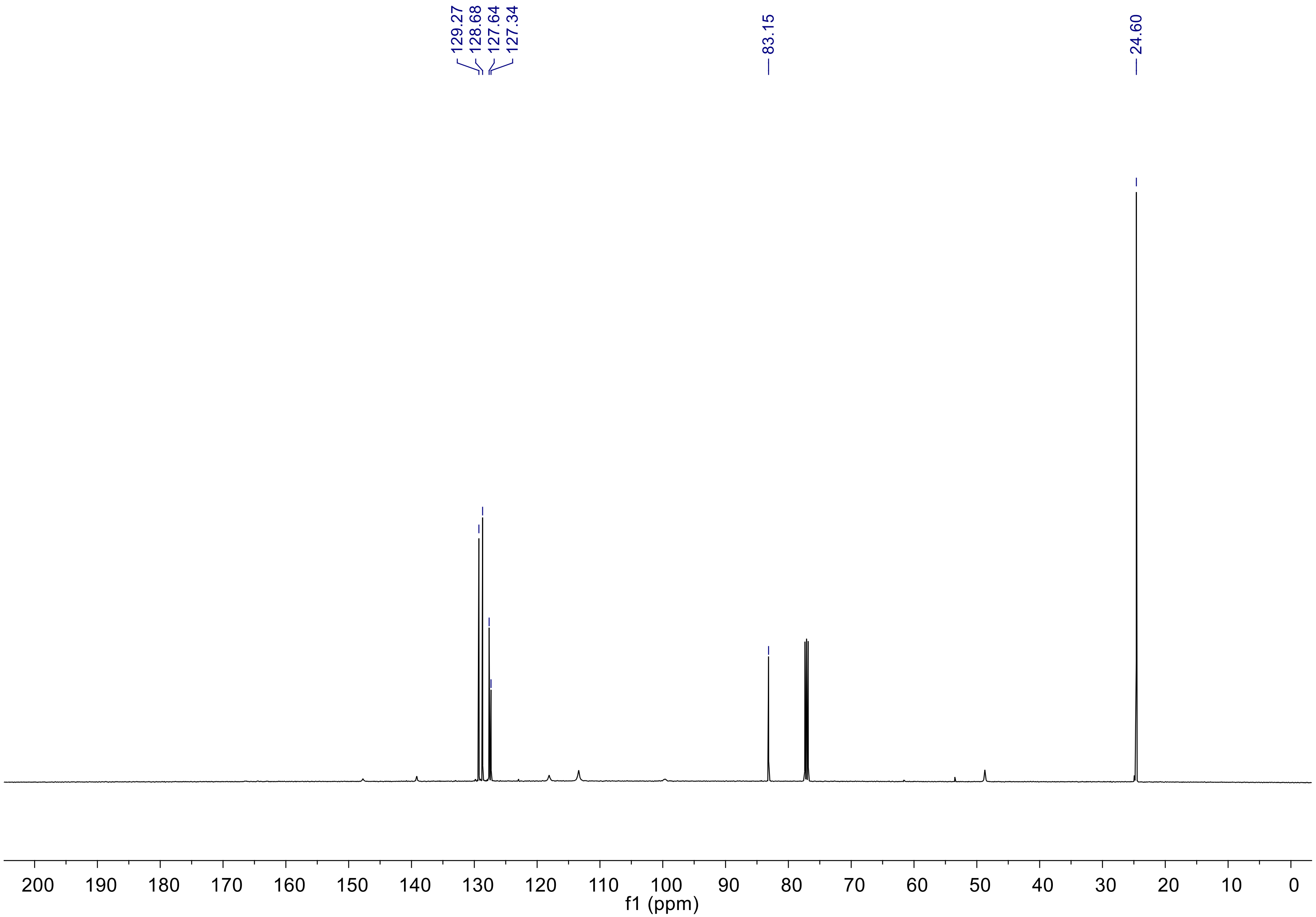

S142 ^{1}H NMR (400 MHz, $CDCl_3$, 298 K) spectrum of *4,4,5,5-tetramethyl-N-(4-methylbenzyl)-N-phenyl-1,3,2-dioxaborolan-2-amine* **3b**.

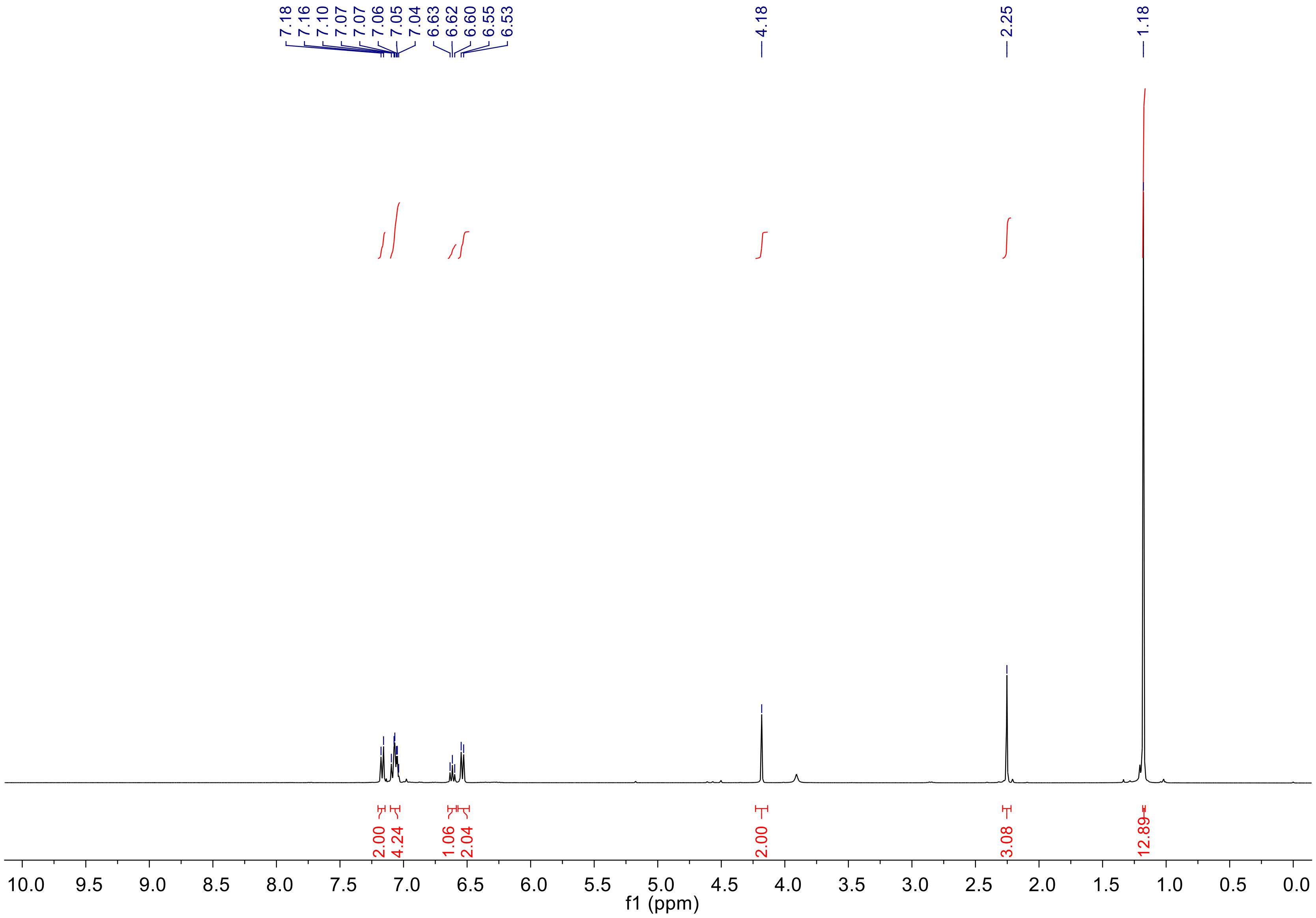

S143 ^{11}B NMR (128 MHz, $CDCl_3$, 298 K) spectrum of *4,4,5,5-tetramethyl-N-(4-methylbenzyl)-N-phenyl-1,3,2-dioxaborolan-2-amine* **3b**.

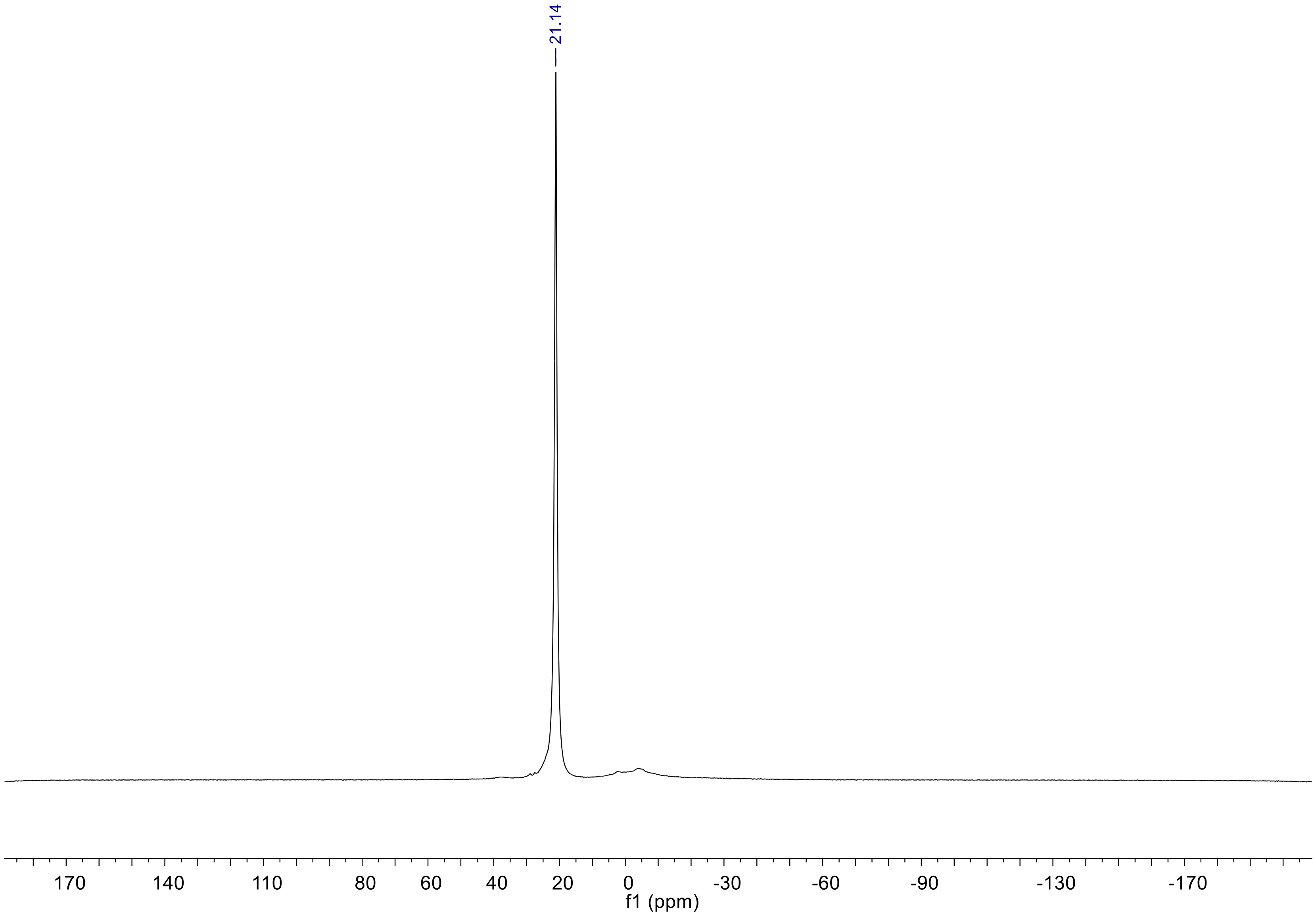

S144 ^{13}C NMR (101 MHz, $CDCl_3$, 298 K) spectrum of *4,4,5,5-tetramethyl-N-(4-methylbenzyl)-N-phenyl-1,3,2-dioxaborolan-2-amine* **3b**.

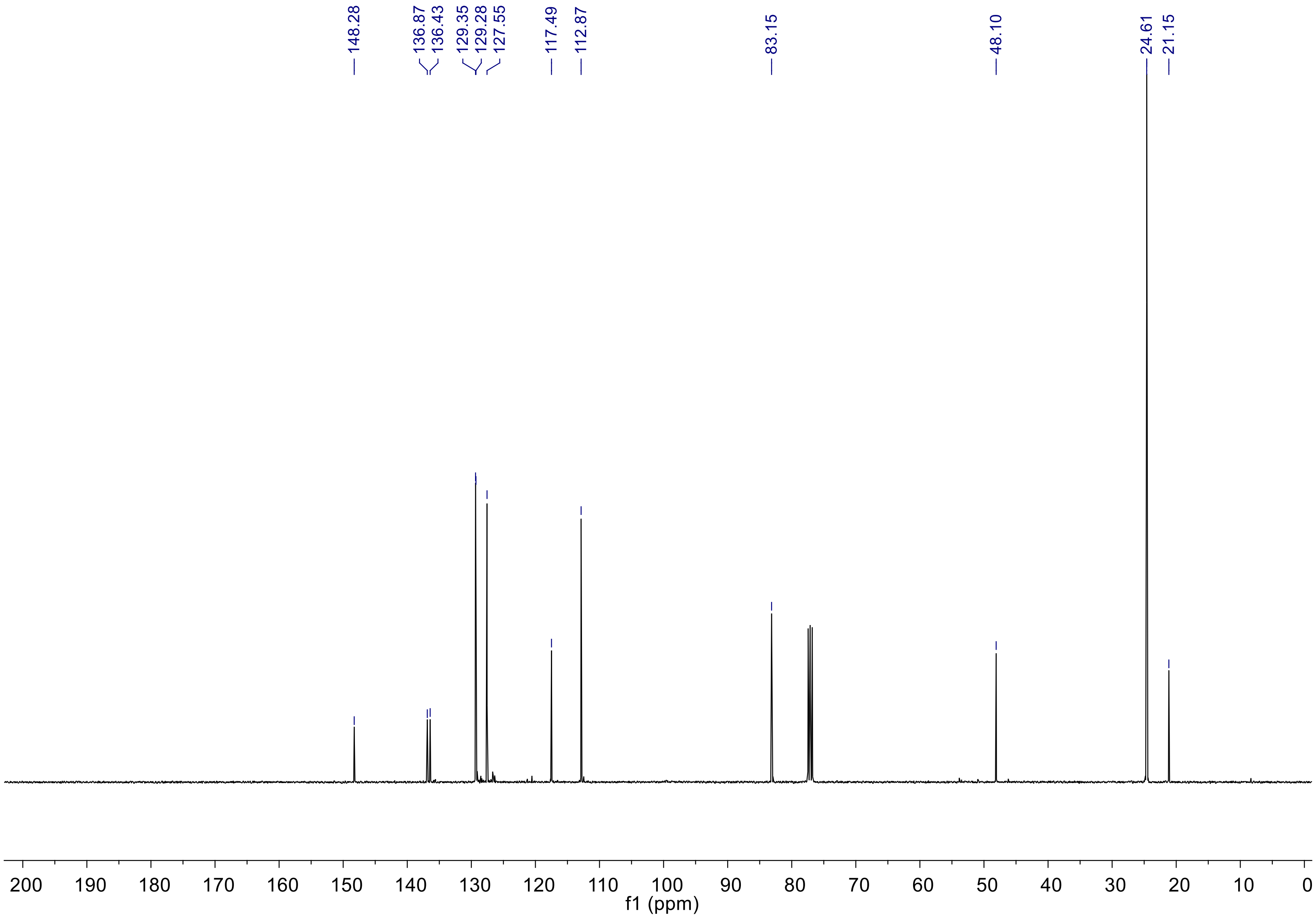

S145 ^{1}H NMR (400 MHz, $CDCl_3$, 298 K) spectrum of *4,4,5,5-tetramethyl-N-phenyl-N-(2,4,6-trimethylbenzyl)-1,3,2-dioxaborolan-2-amine* **3c**.

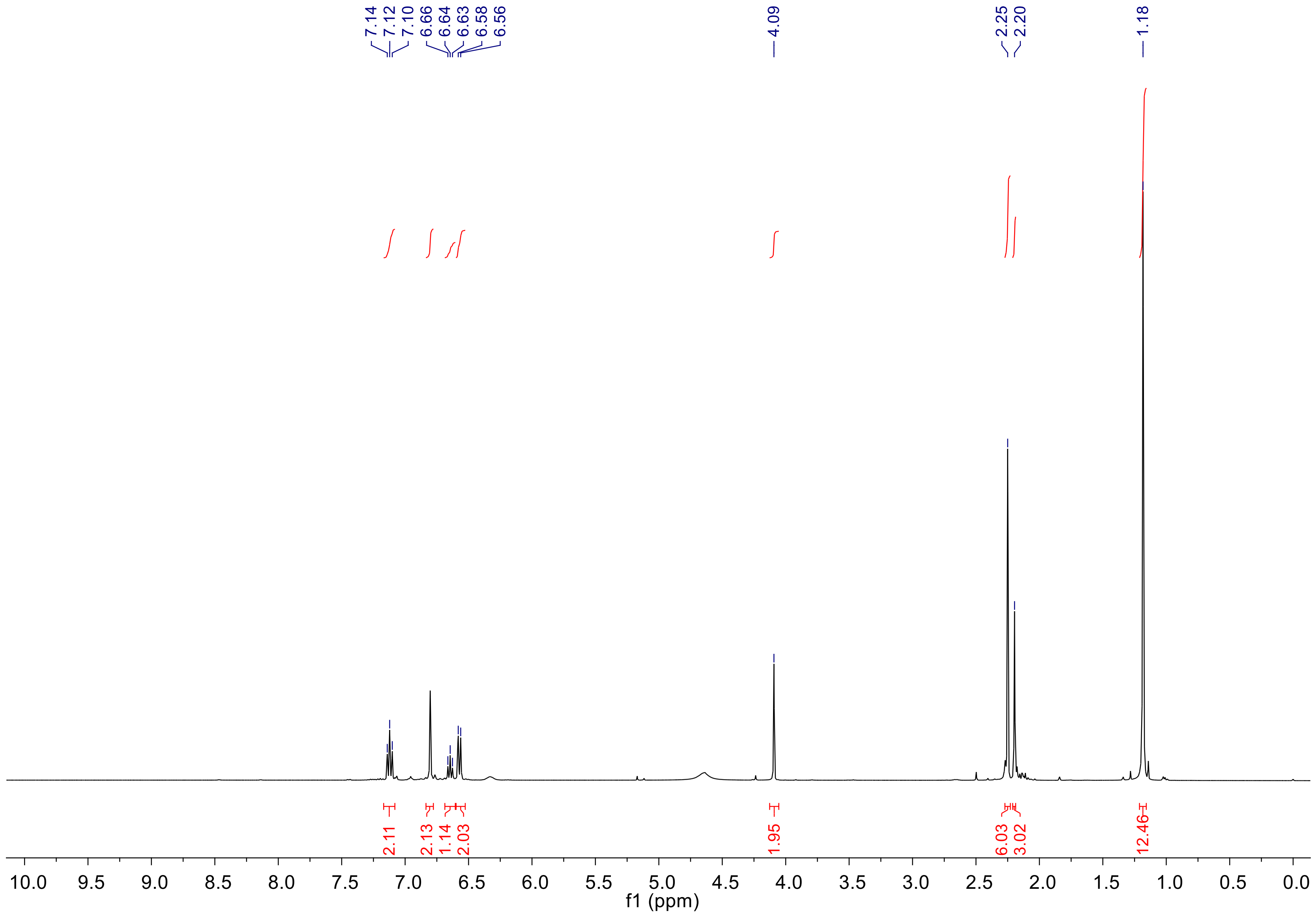

S146 ^{11}B NMR (128 MHz, $CDCl_3$, 298 K) spectrum of *4,4,5,5-tetramethyl-N-phenyl-N-(2,4,6-trimethylbenzyl)-1,3,2-dioxaborolan-2-amine* **3c**.

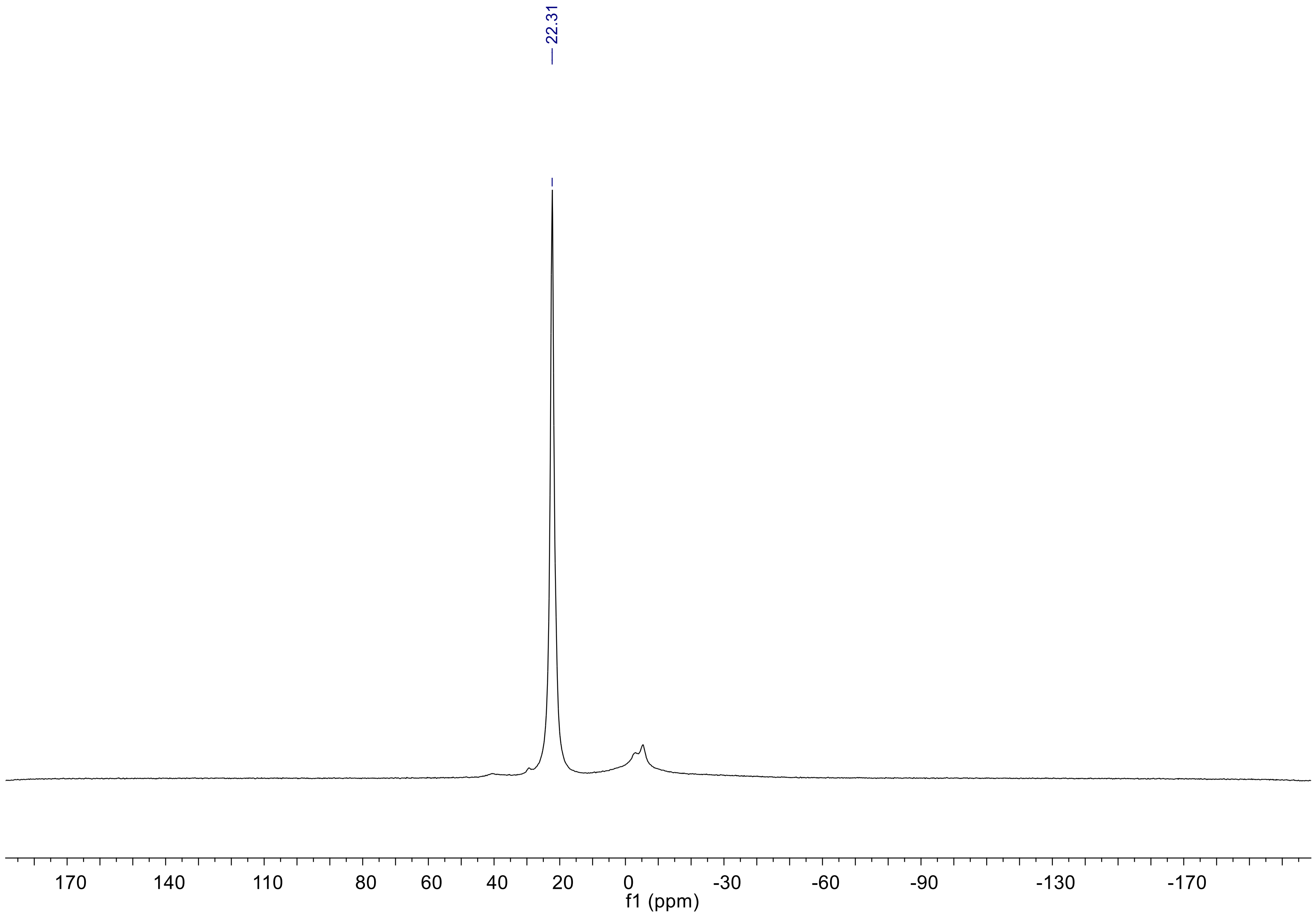

S147 ^{13}C NMR (101 MHz, $CDCl_3$, 298 K) spectrum of *4,4,5,5-tetramethyl-N-phenyl-N-(2,4,6-trimethylbenzyl)-1,3,2-dioxaborolan-2-amine* **3c**.

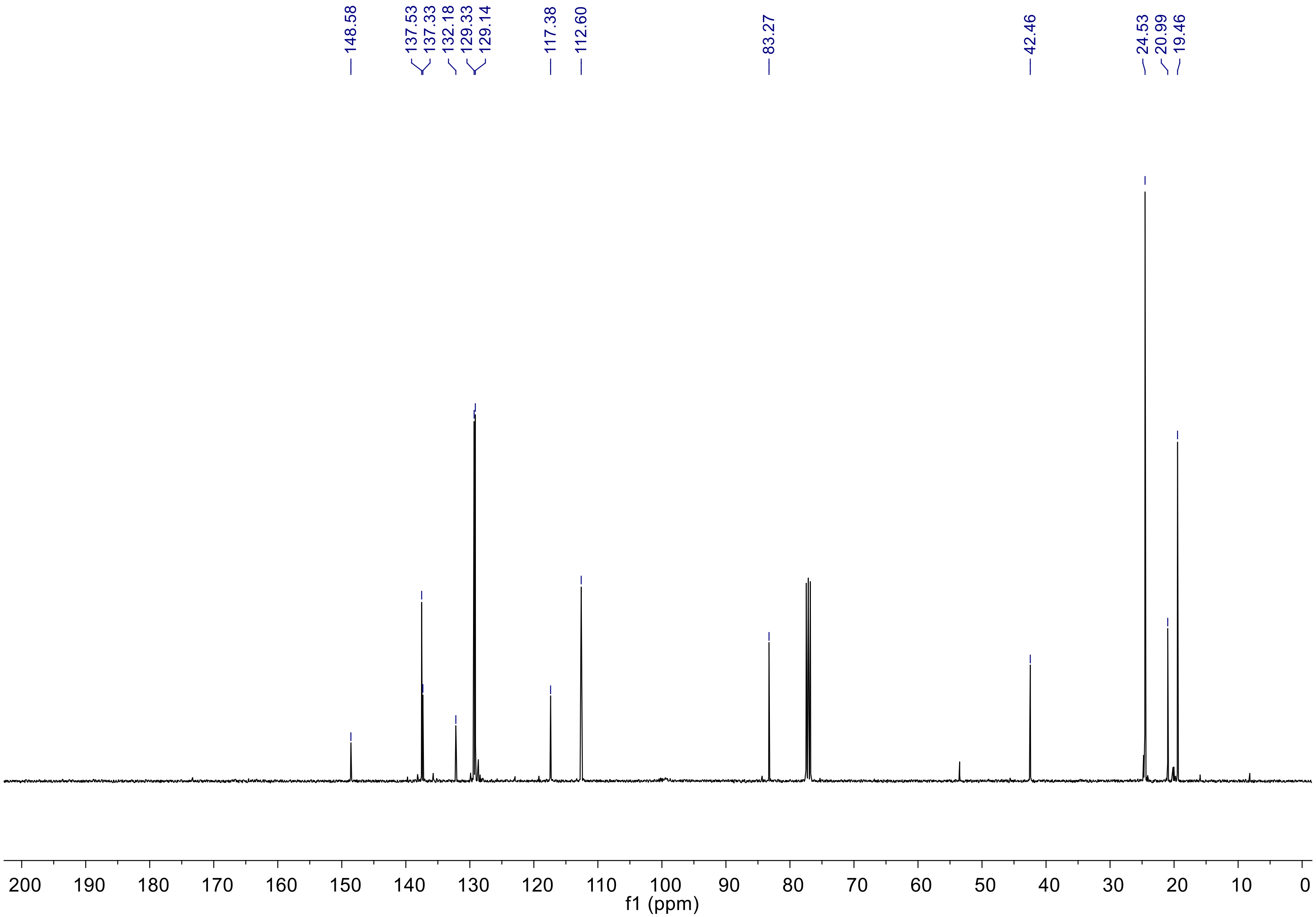

S148 ^{1}H NMR (400 MHz, $CDCl_3$, 298 K) spectrum of the hydrolysis product of *N-(4-methoxybenzyl)-4,4,5,5-tetramethyl-N-phenyl-1,3,2-dioxaborolan-2-amine* **3d**.

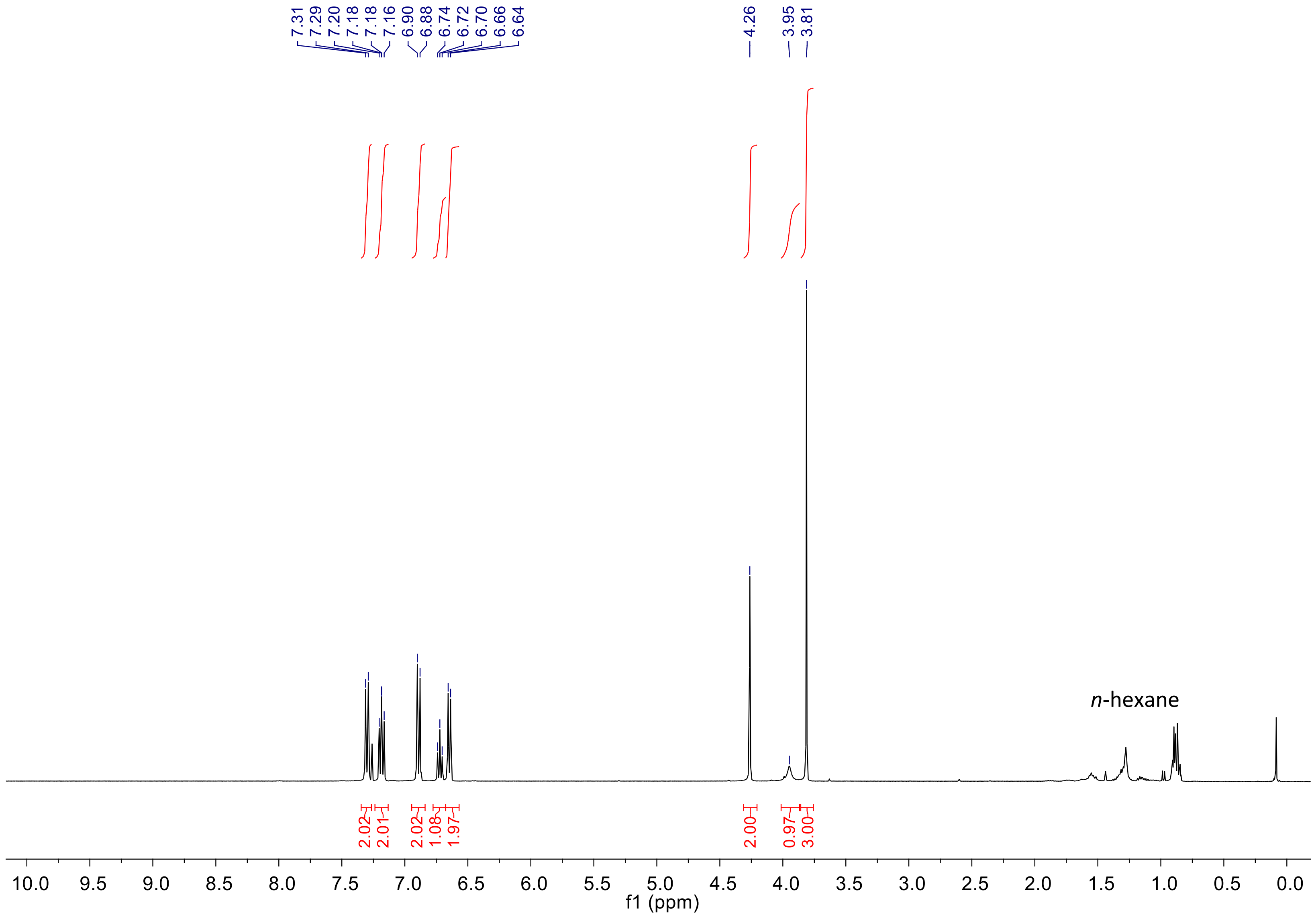

S149 ^{13}C NMR (101 MHz, $CDCl_3$, 298 K) spectrum of the hydrolysis product of *N-(4-methoxybenzyl)-4,4,5,5-tetramethyl-N-phenyl-1,3,2-dioxaborolan-2-amine* **3d**.

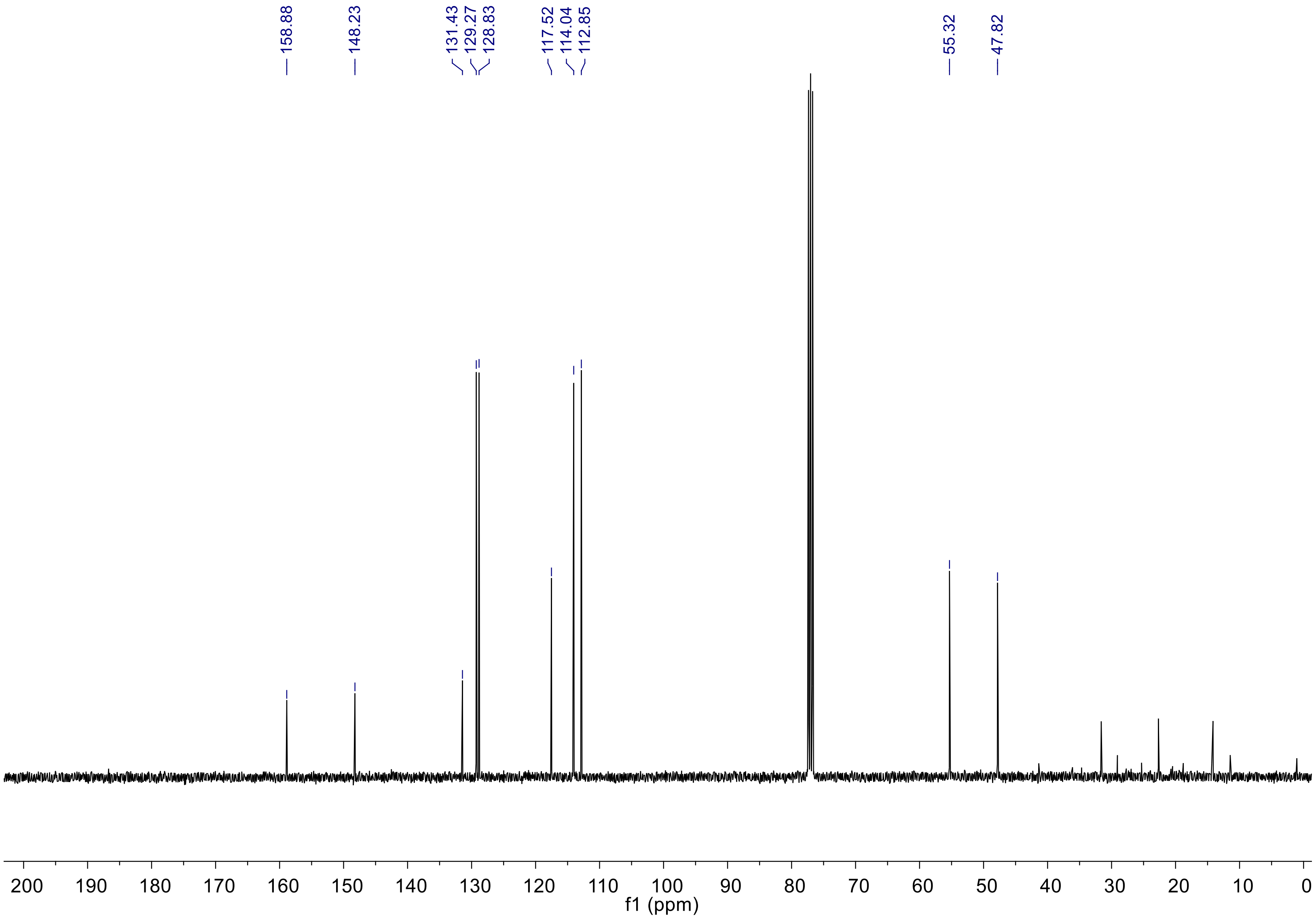

S150 ^{1}H NMR (400 MHz, $CDCl_3$, 298 K) spectrum of the hydrolysis product of *N-(2-methoxybenzyl)-4,4,5,5-tetramethyl-N-phenyl-1,3,2-dioxaborolan-2-amine* **3e**.

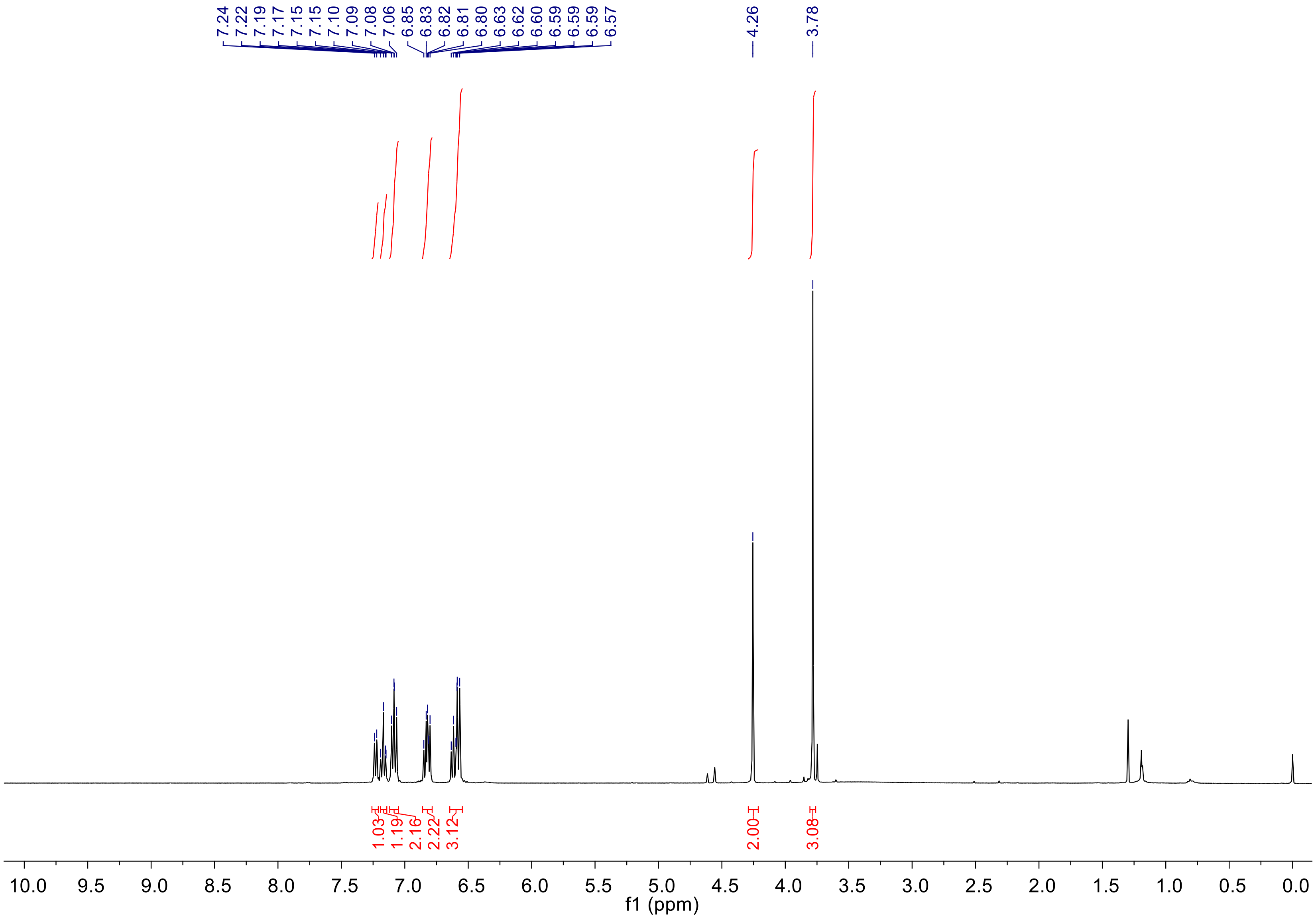

S151 ^{13}C NMR (101 MHz, $CDCl_3$, 298 K) spectrum of the hydrolysis product of *N-(2-methoxybenzyl)-4,4,5,5-tetramethyl-N-phenyl-1,3,2-dioxaborolan-2-amine* **3e**.

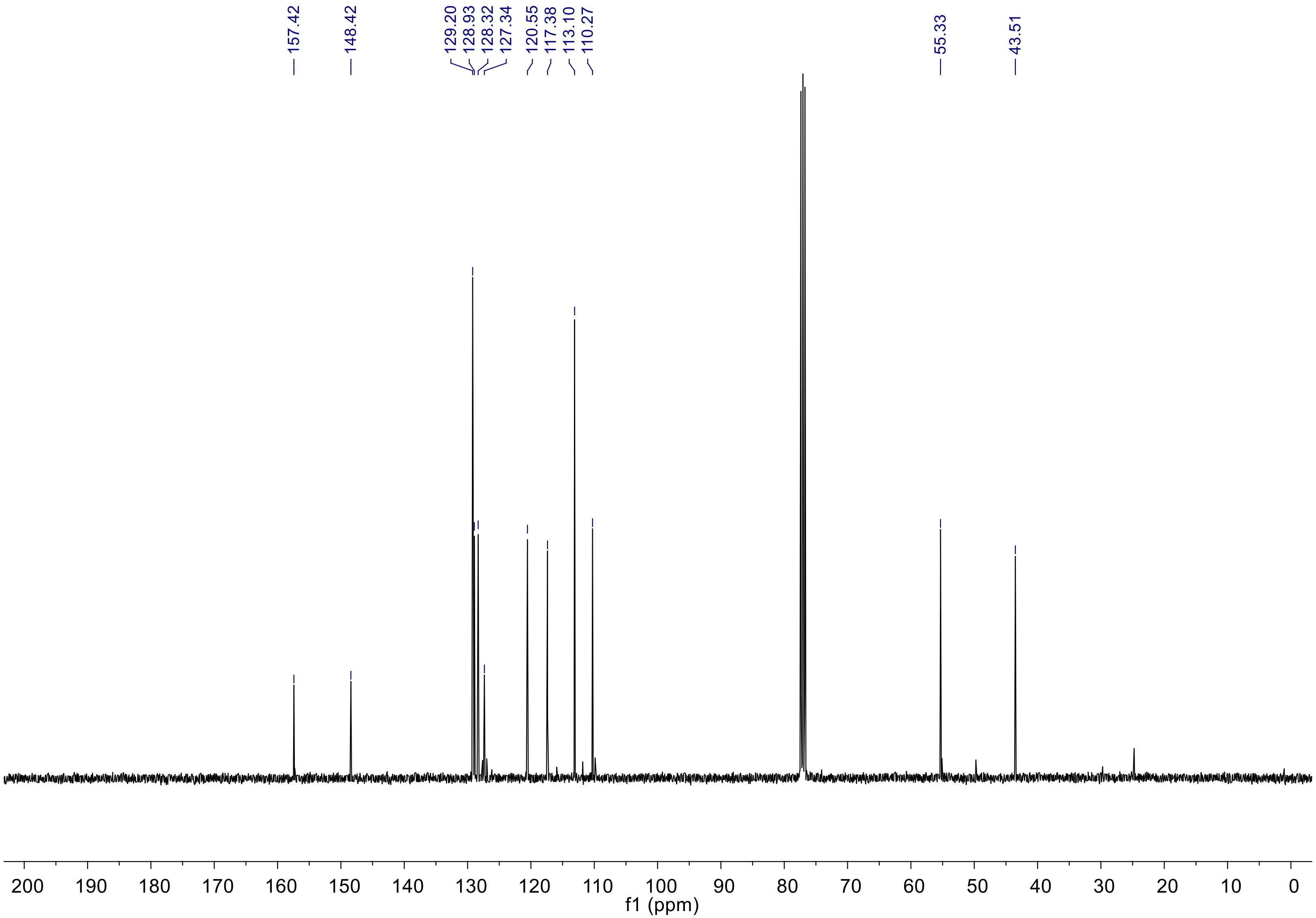

S152 ^{1}H NMR (400 MHz, $CDCl_3$, 298 K) spectrum of the hydrolysis product of *N-(4-fluorobenzyl)-4,4,5,5-tetramethyl-N-phenyl-1,3,2-dioxaborolan-2-amine* **3f**.

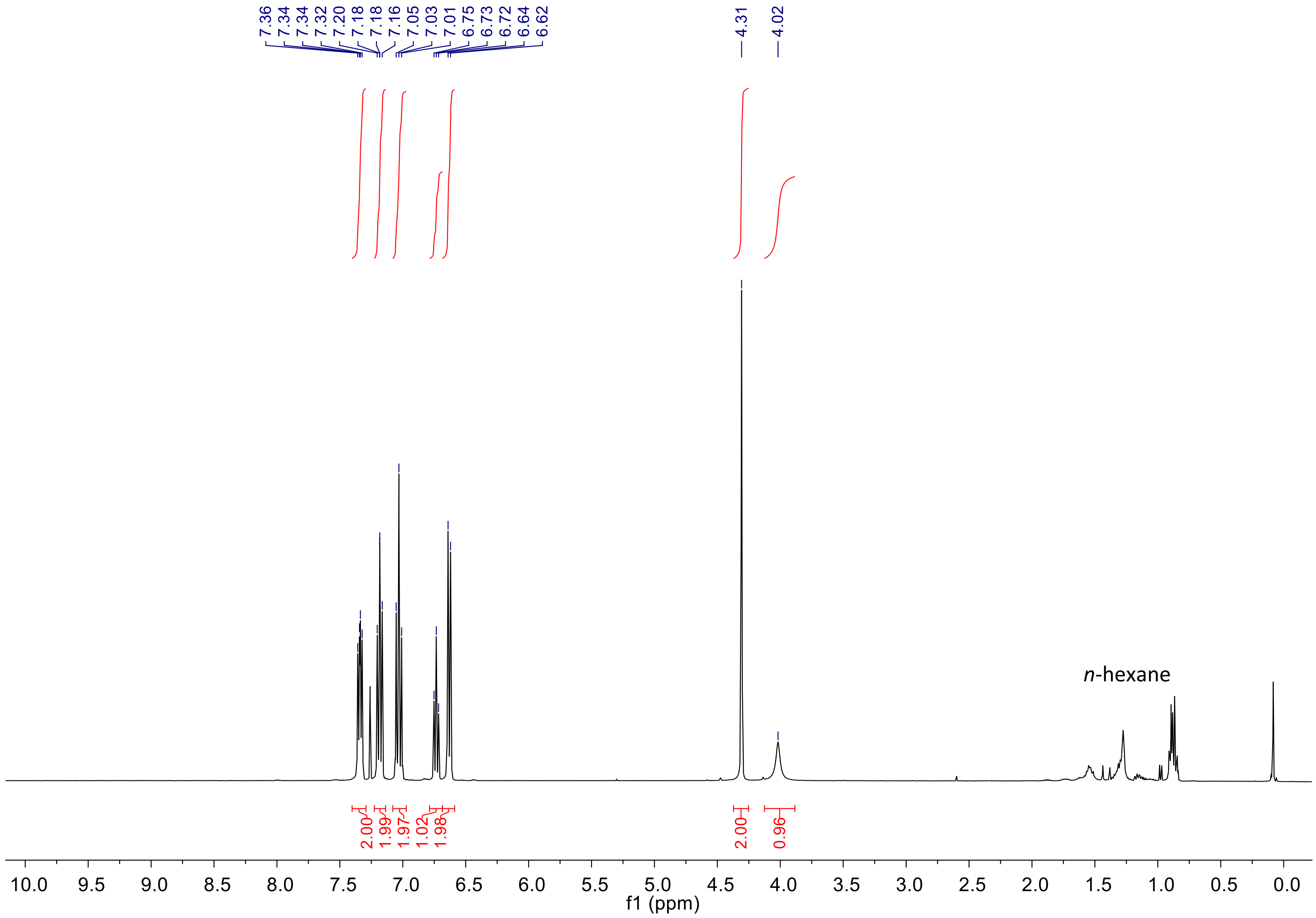

S153 ^{13}C NMR (101 MHz, $CDCl_3$, 298 K) spectrum of the hydrolysis product of *N-(4-fluorobenzyl)-4,4,5,5-tetramethyl-N-phenyl-1,3,2-dioxaborolan-2-amine* **3f**.

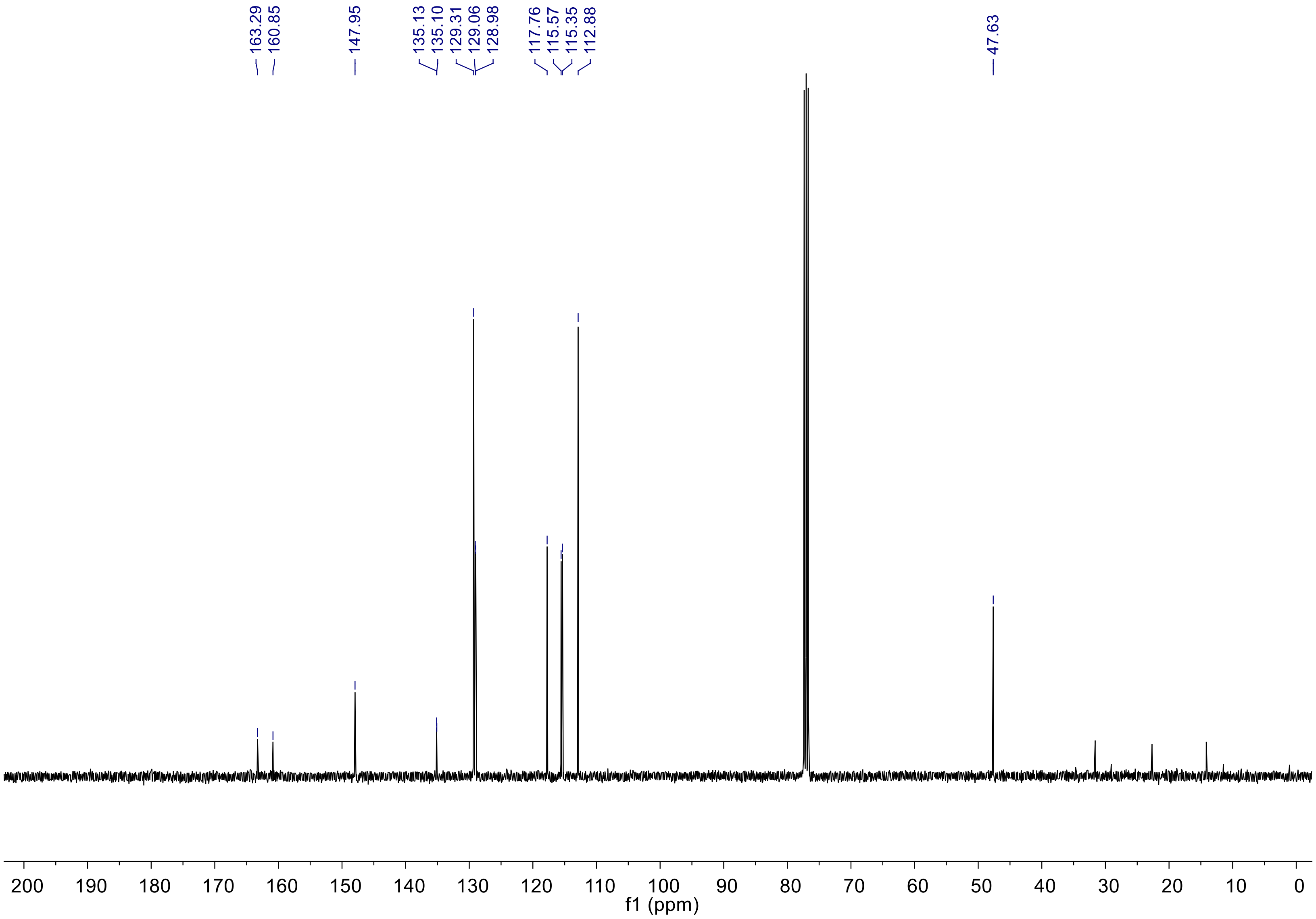

S154 ^{19}F NMR (377 MHz, $CDCl_3$, 298 K) spectrum of the hydrolysis product of *N-(4-fluorobenzyl)-4,4,5,5-tetramethyl-N-phenyl-1,3,2-dioxaborolan-2-amine* **3f**.

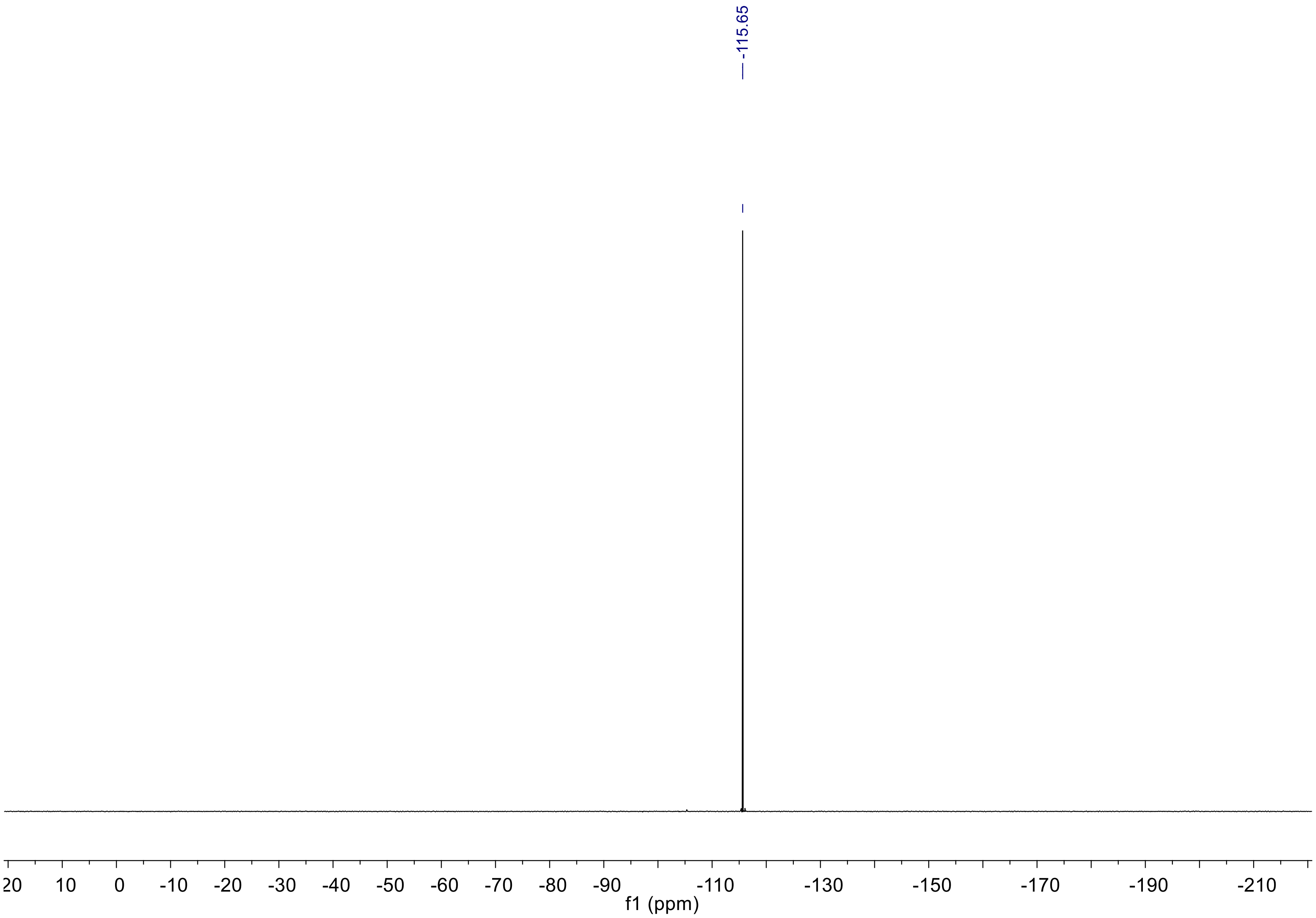

S155 ^{1}H NMR (400 MHz, $CDCl_3$, 298 K) spectrum of *the hydrolysis product of 4,4,5,5-tetramethyl-N-(4-nitrobenzyl)-N-phenyl-1,3,2-dioxaborolan-2-amine* **3g**.

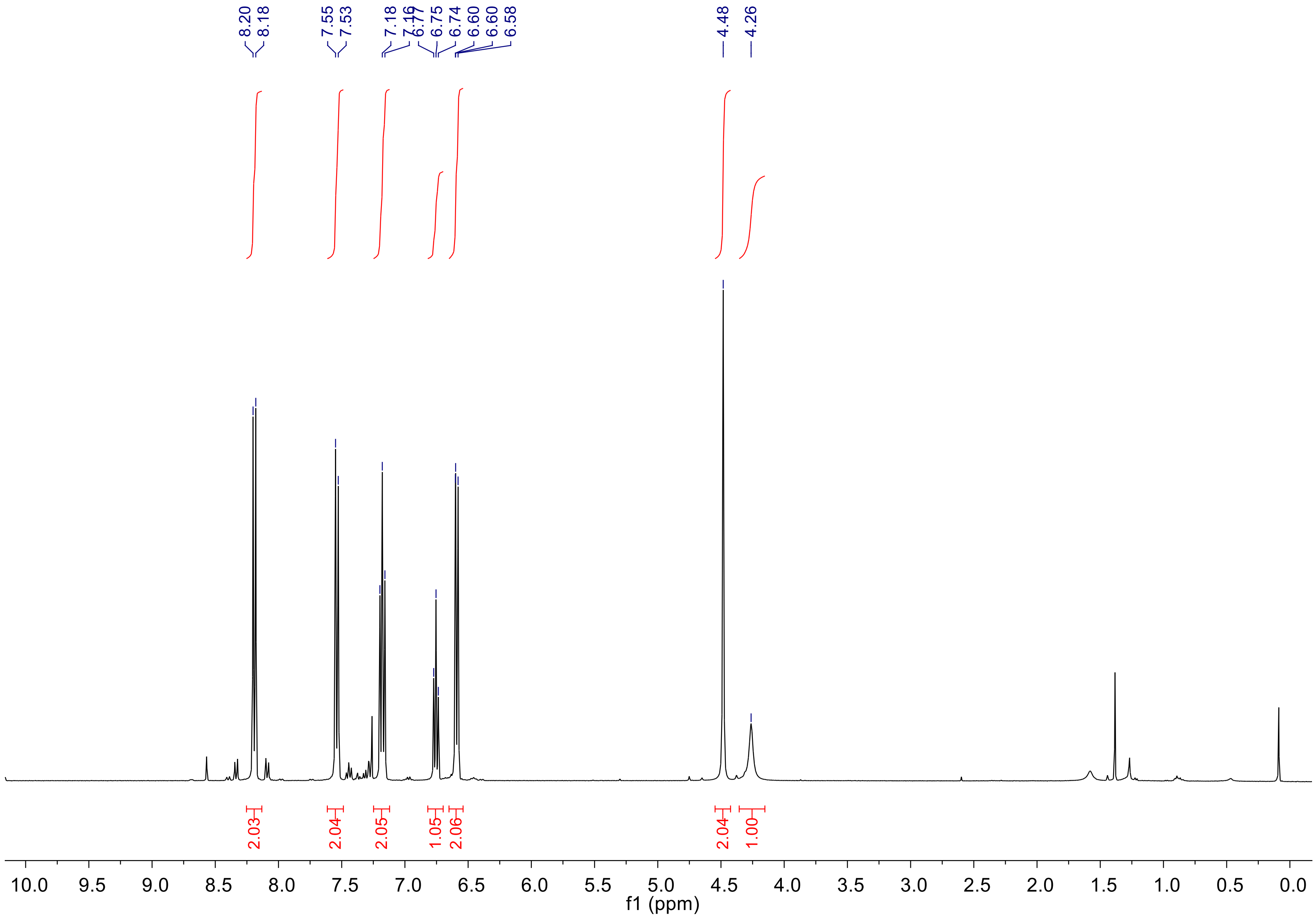

S156 ^{13}C NMR (101 MHz, $CDCl_3$, 298 K) spectrum of *the hydrolysis product of 4,4,5,5-tetramethyl-N-(4-nitrobenzyl)-N-phenyl-1,3,2-dioxaborolan-2-amine* **3g**.

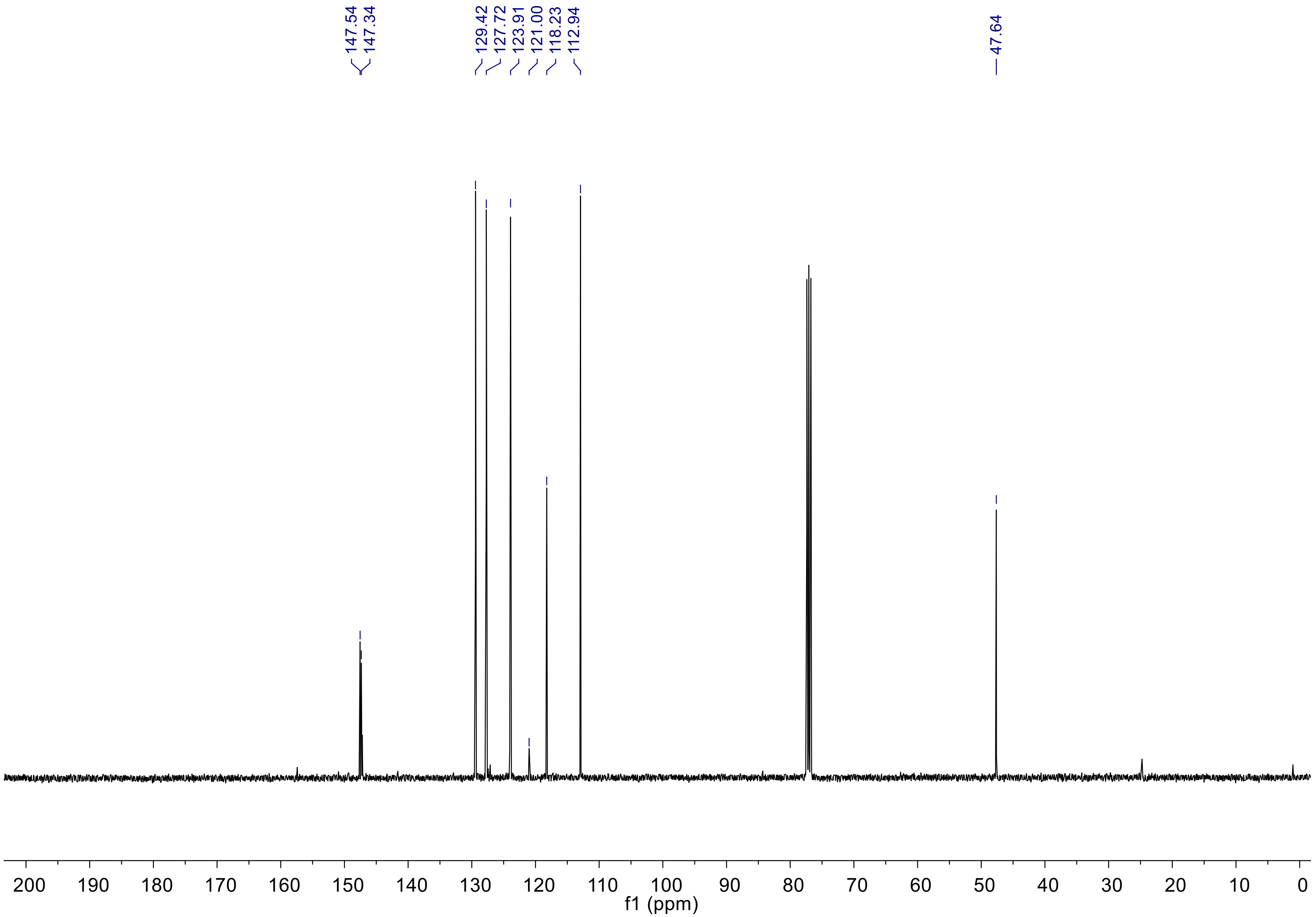

S157 ^{1}H NMR (400 MHz, $CDCl_3$, 298 K) spectrum of *4,4,5,5-tetramethyl-N-(naphthalen-2-ylmethyl)-N-phenyl-1,3,2-dioxaborolan-2-amine* **3h**.

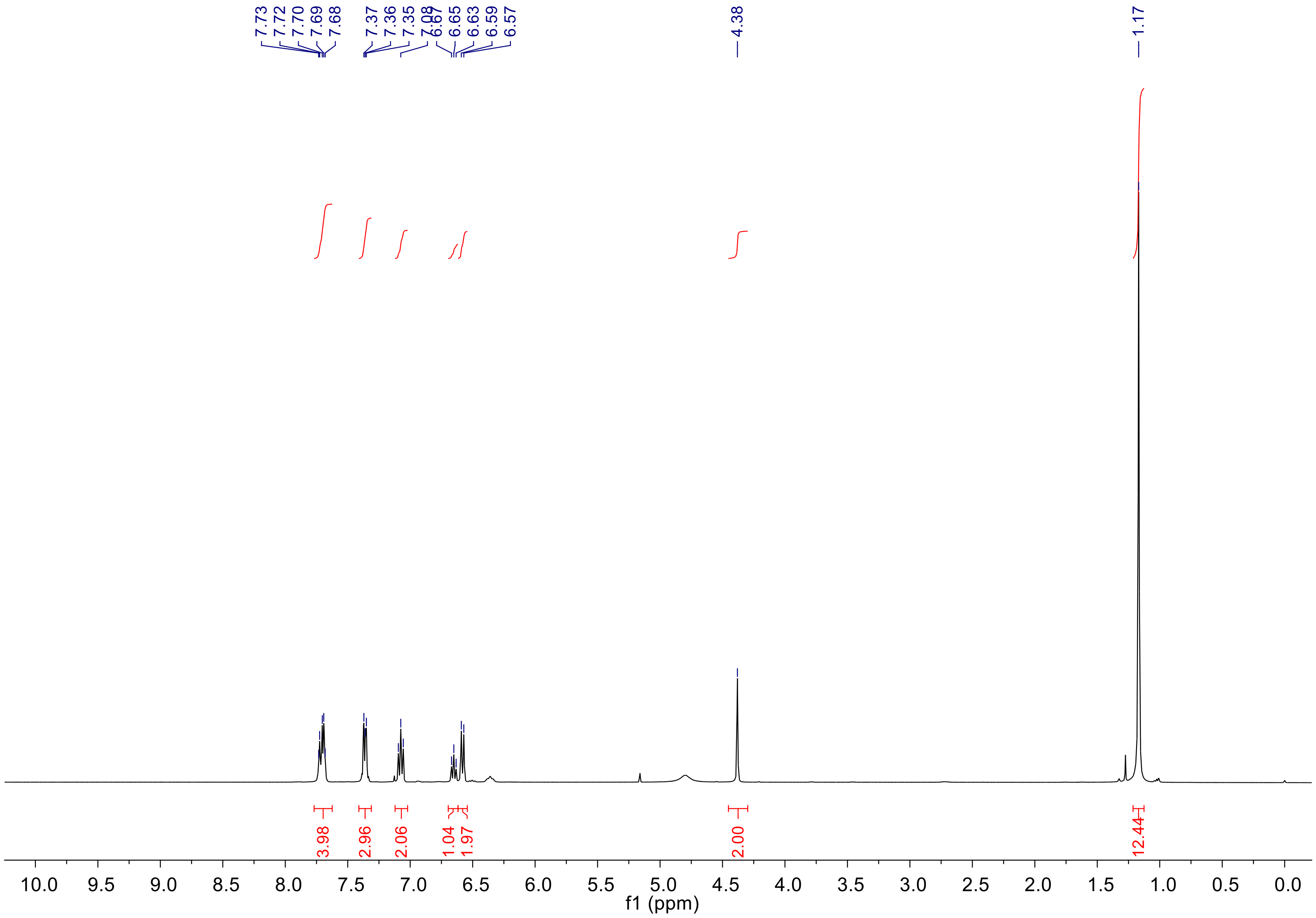

S158 ^{11}B NMR (128 MHz, $CDCl_3$, 298 K) spectrum of *4,4,5,5-tetramethyl-N-(naphthalen-2-ylmethyl)-N-phenyl-1,3,2-dioxaborolan-2-amine* **3h**.

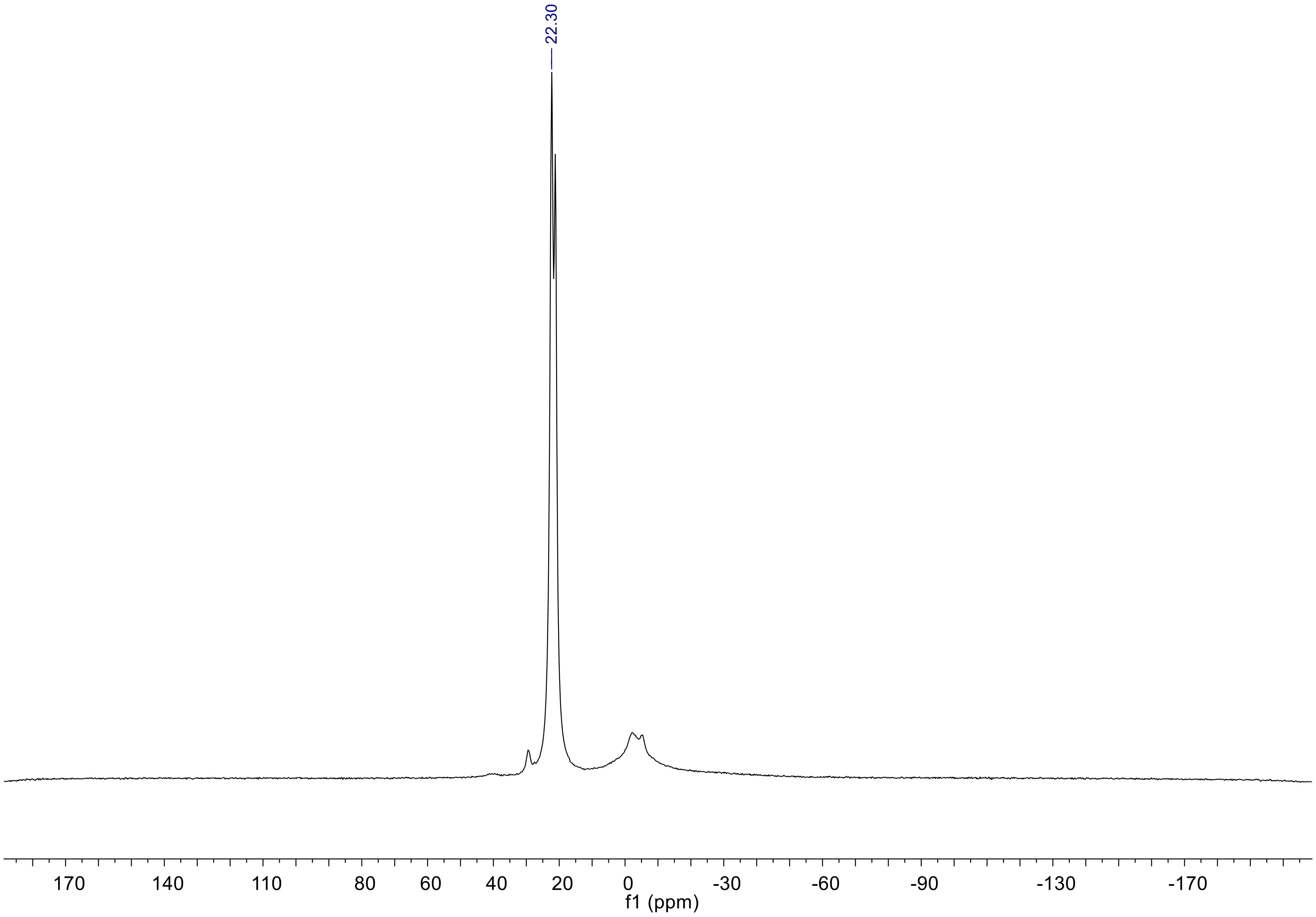

S159 ^{13}C NMR (101 MHz, $CDCl_3$, 298 K) spectrum of *4,4,5,5-tetramethyl-N-(naphthalen-2-ylmethyl)-N-phenyl-1,3,2-dioxaborolan-2-amine* **3h**.

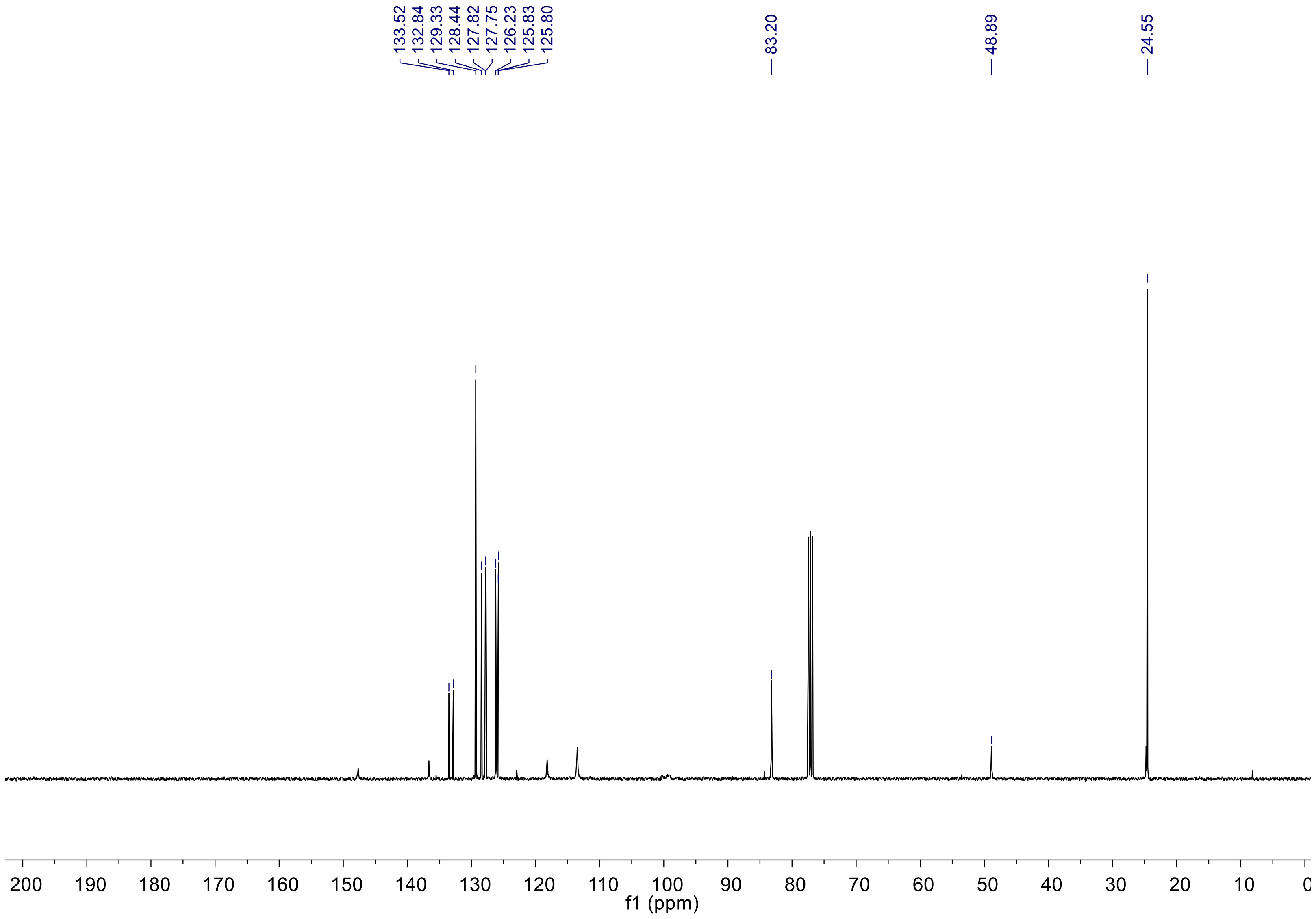

S160 ^{1}H NMR (500 MHz, $CDCl_3$, 298 K) spectrum of *N-benzyl-N-butyl-4,4,5,5-tetramethyl-1,3,2-dioxaborolan-2-amine*. **3i**.

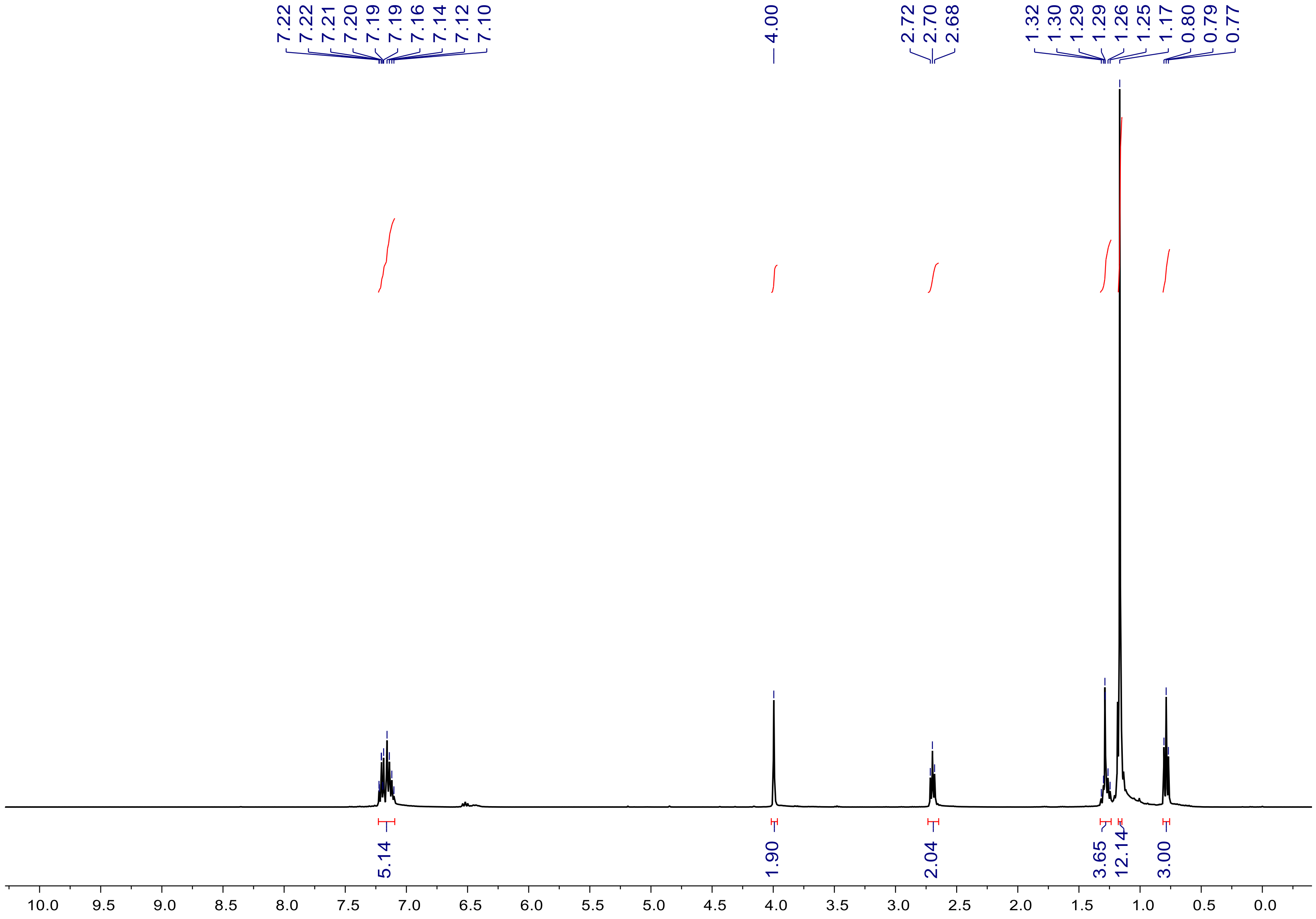

S161 ^{11}B NMR (160 MHz, $CDCl_3$, 298 K) spectrum of *N-benzyl-N-butyl-4,4,5,5-tetramethyl-1,3,2-dioxaborolan-2-amine* **3i.**

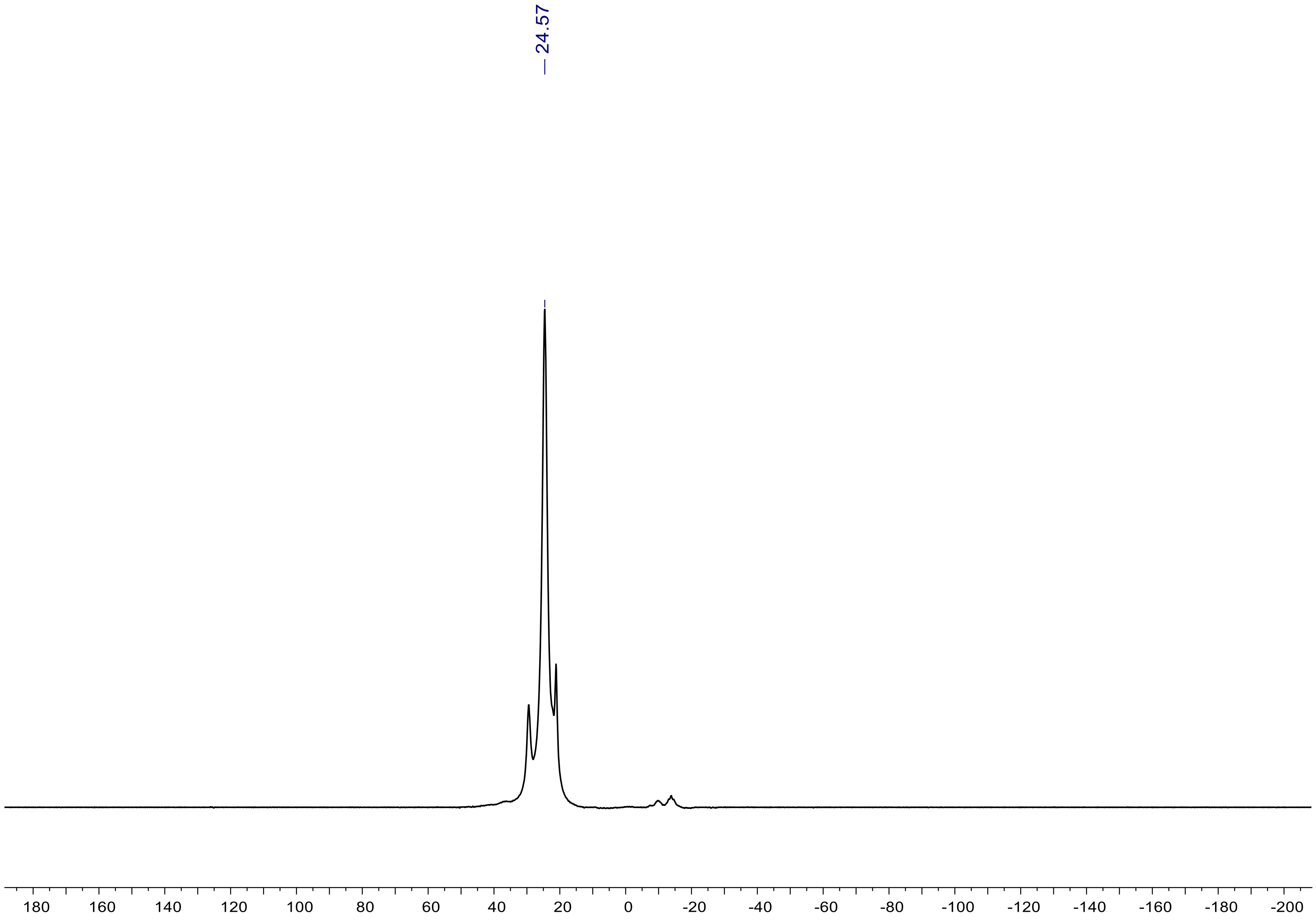

S162 ^{13}C NMR (126 MHz, $CDCl_3$, 298 K) spectrum of *N-benzyl-N-butyl-4,4,5,5-tetramethyl-1,3,2-dioxaborolan-2-amine* **3i**.

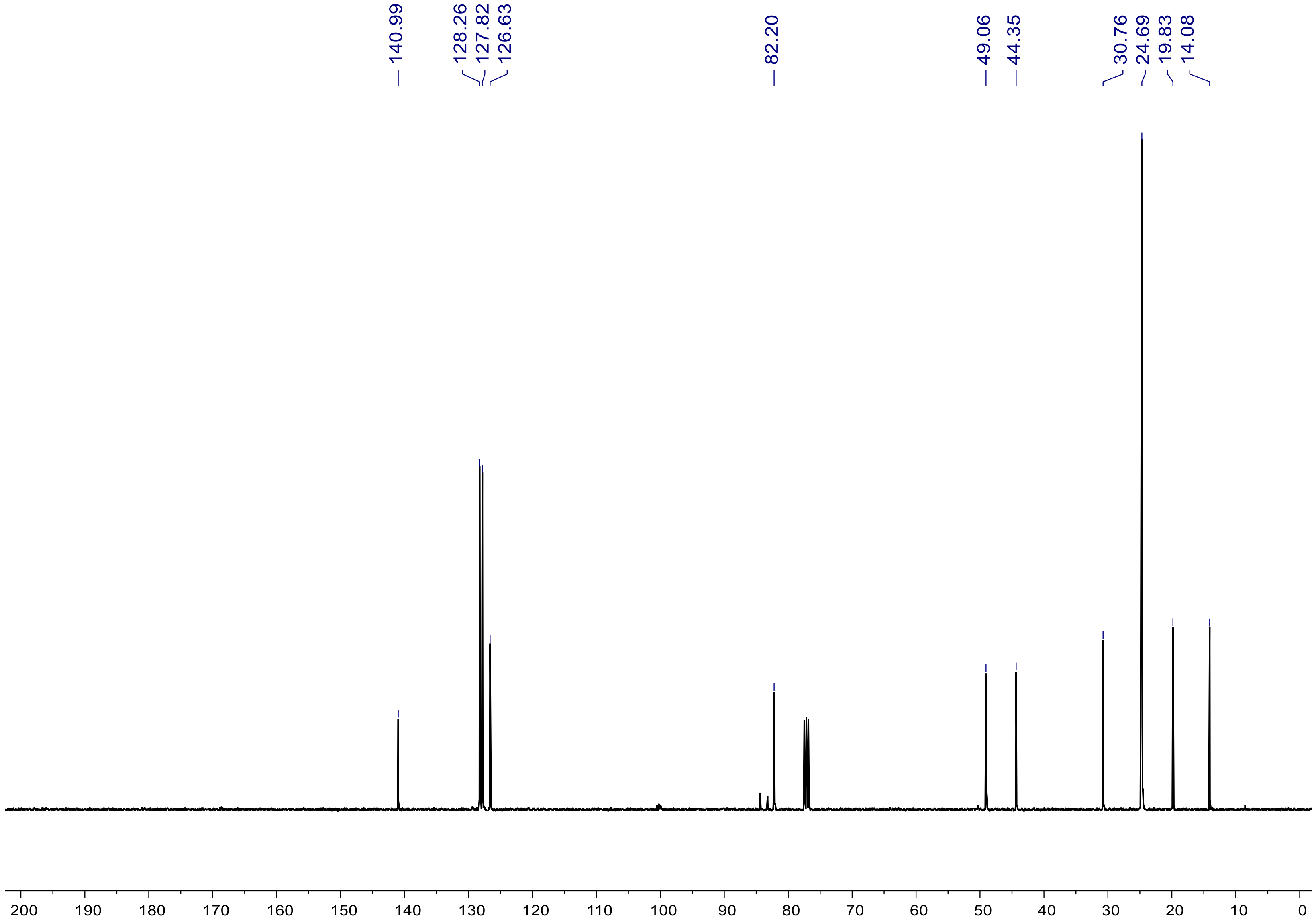

S163 ^{1}H NMR (500 MHz, $CDCl_3$, 298 K) spectrum of *N-benzyl-N-isopropyl-4,4,5,5-tetramethyl-1,3,2-dioxaborolan-2-amine* **3j**.

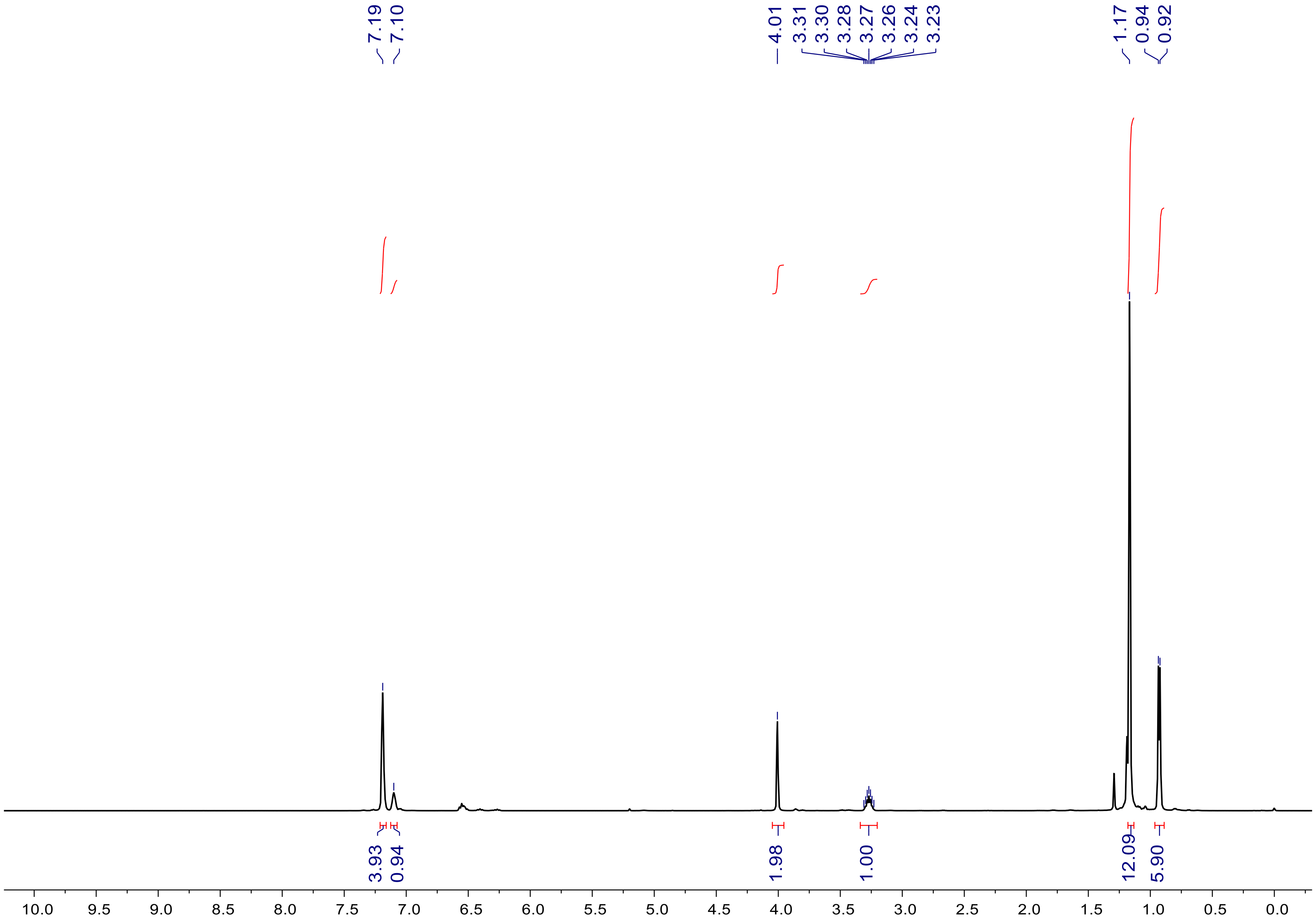

S164 ^{11}B NMR (160 MHz, $CDCl_3$, 298 K) spectrum of *N-benzyl-N-isopropyl-4,4,5,5-tetramethyl-1,3,2-dioxaborolan-2-amine* **3j**.

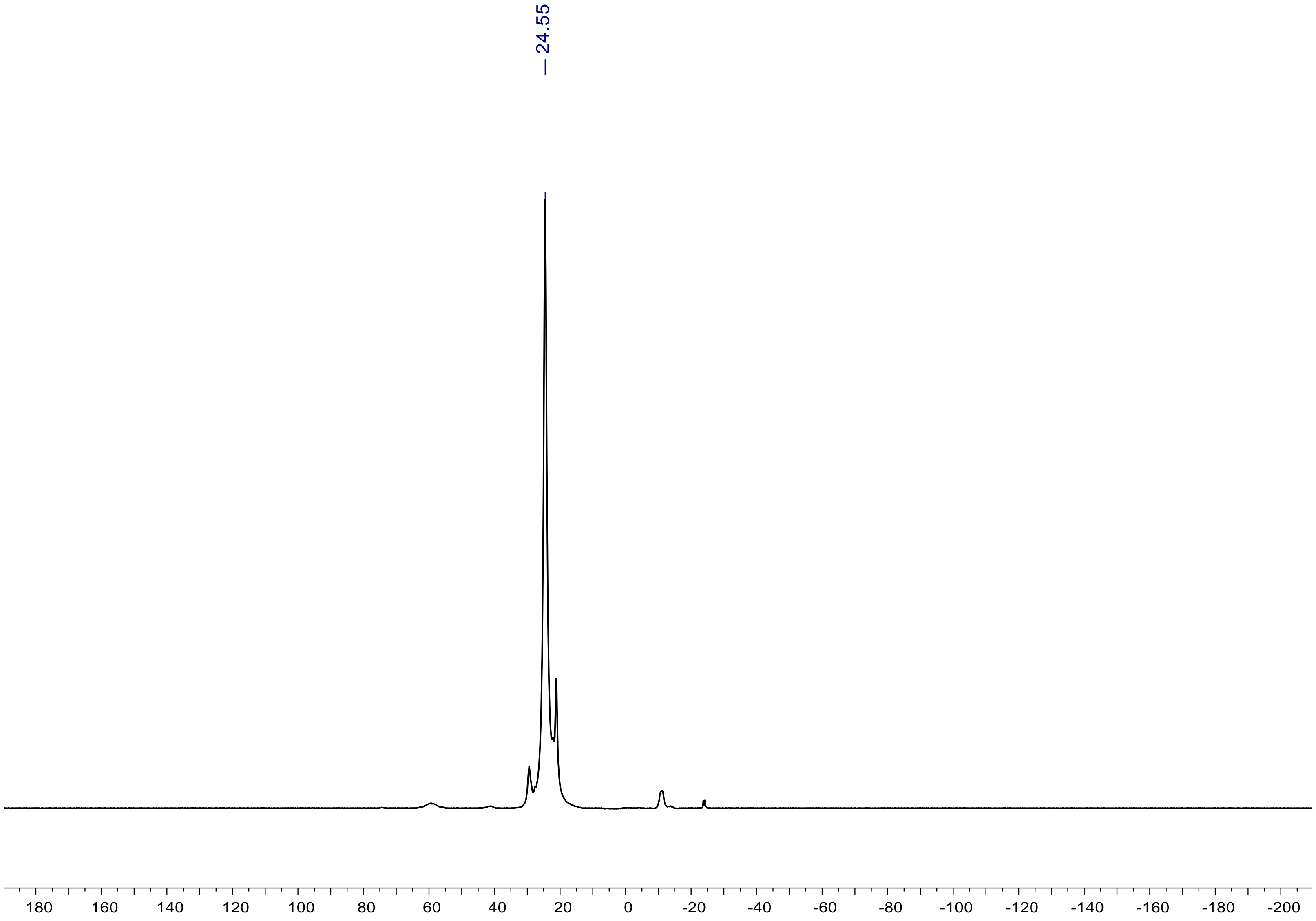

S165 ^{13}C NMR (126 MHz, $CDCl_3$, 298 K) spectrum of *N-benzyl-N-isopropyl-4,4,5,5-tetramethyl-1,3,2-dioxaborolan-2-amine* **3j**.

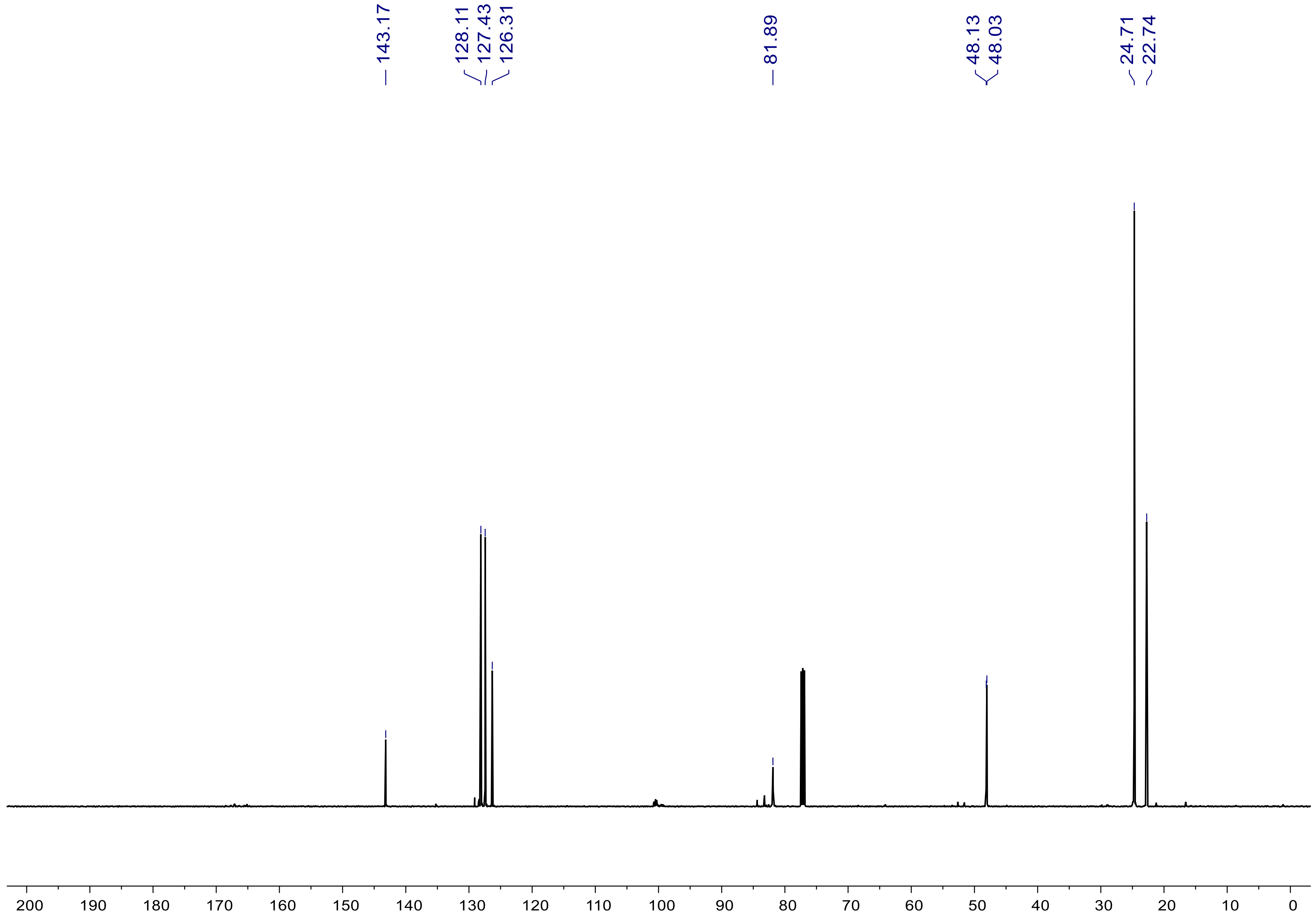

S166 ^{1}H NMR (500 MHz, $CDCl_3$, 298 K) spectrum of *N-benzyl-N-cyclopentyl-4,4,5,5-tetramethyl-1,3,2-dioxaborolan-2-amine* **3k**.

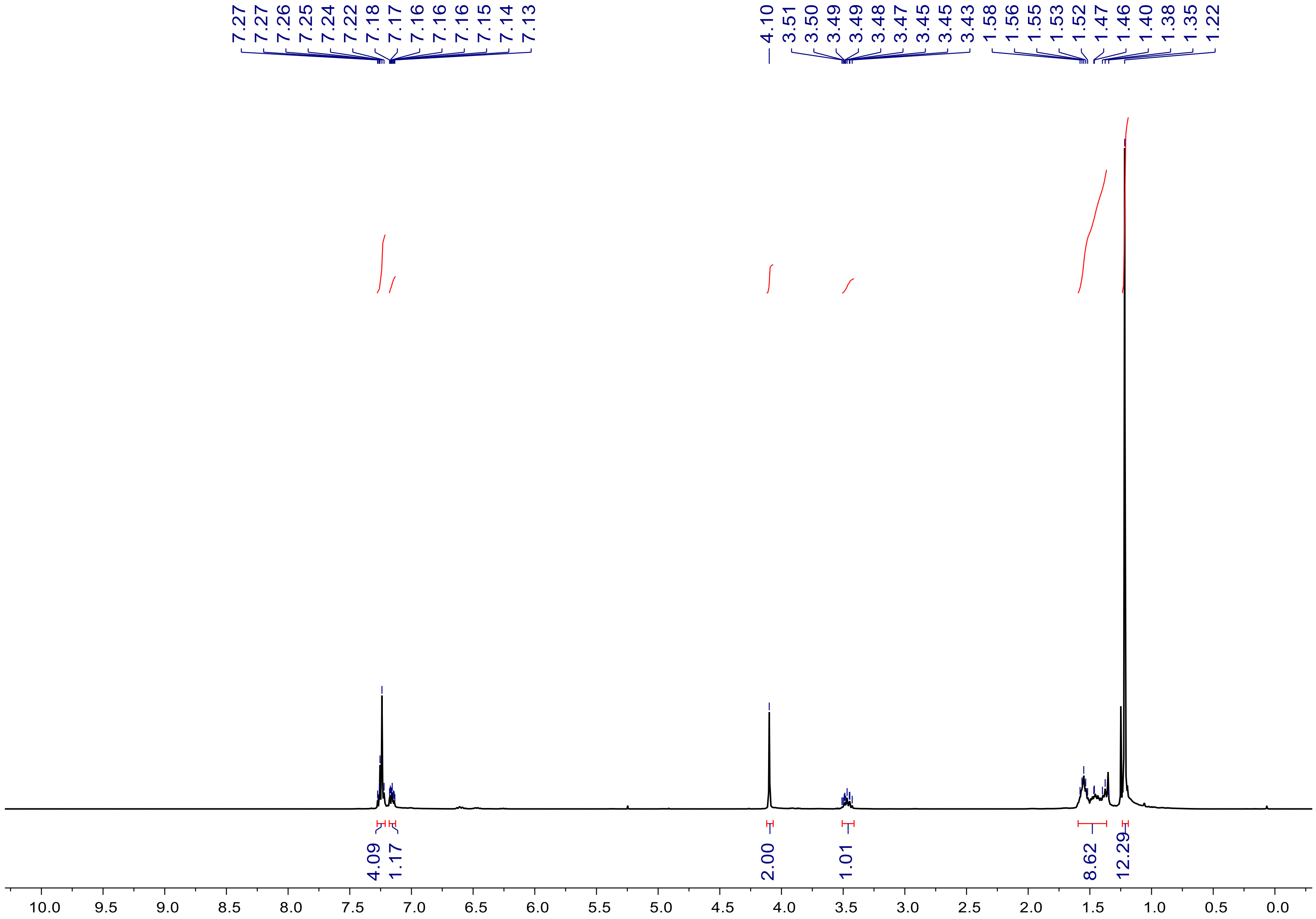

S167 ^{11}B NMR (160 MHz, $CDCl_3$, 298 K) spectrum of *N-benzyl-N-cyclopentyl-4,4,5,5-tetramethyl-1,3,2-dioxaborolan-2-amine* **3k**.

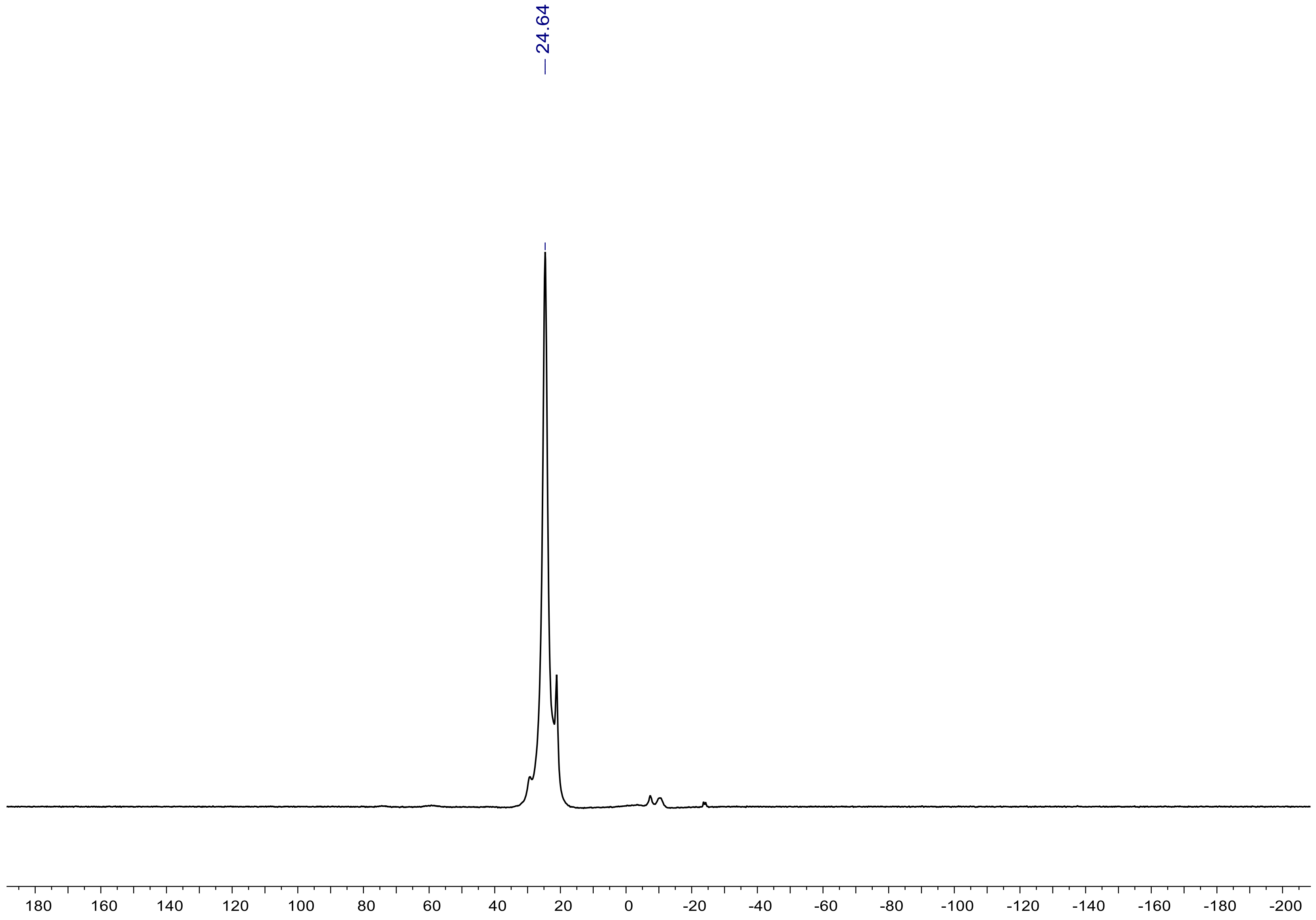

S168 ^{13}C NMR (126 MHz, $CDCl_3$, 298 K) spectrum of *N-benzyl-N-cyclopentyl-4,4,5,5-tetramethyl-1,3,2-dioxaborolan-2-amine* **3k**.

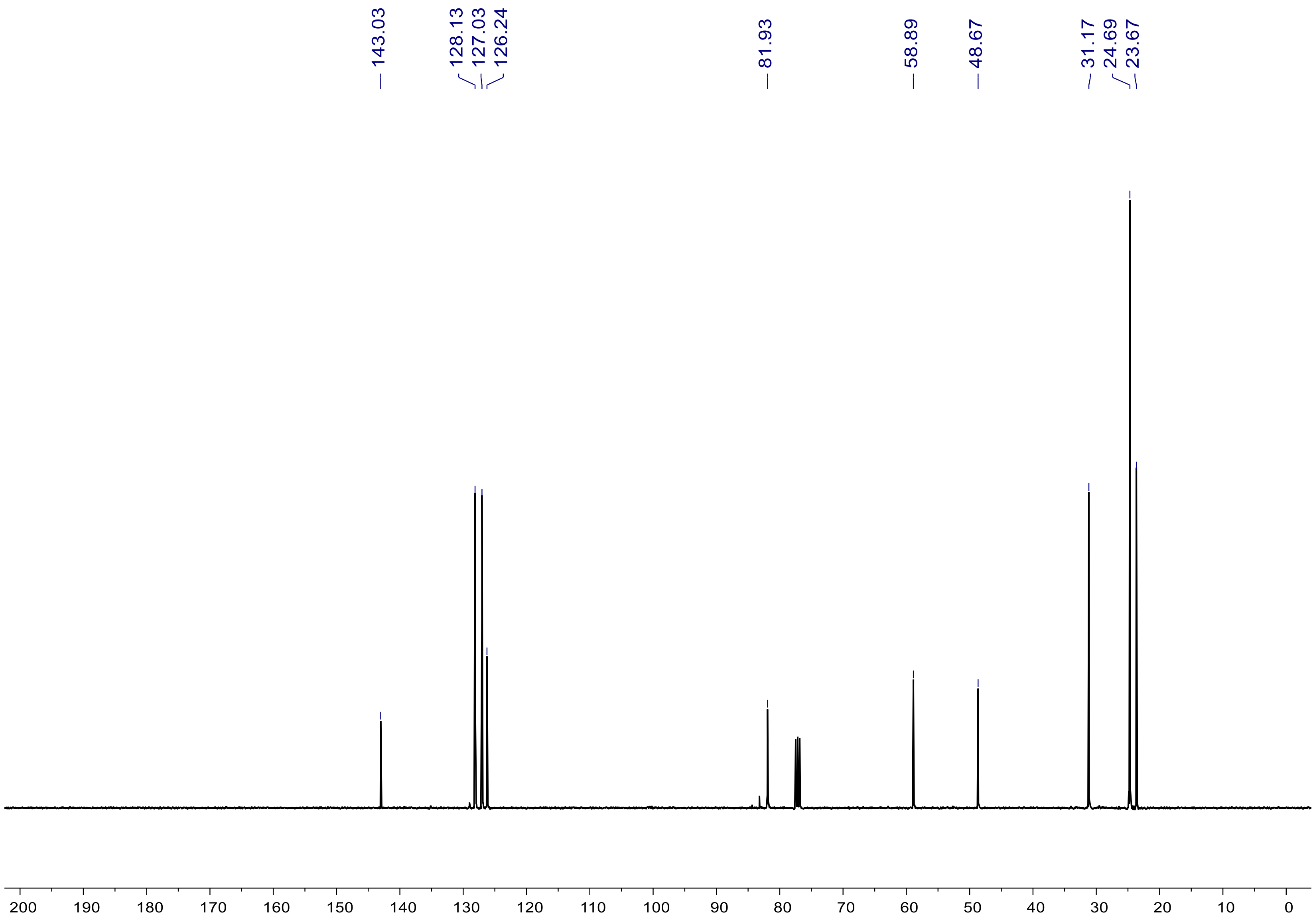

S169 ^{1}H NMR (500 MHz, $CDCl_3$, 298 K) spectrum of *N,N-dibenzyl-4,4,5,5-tetramethyl-1,3,2-dioxaborolan-2-amine* **3l**.

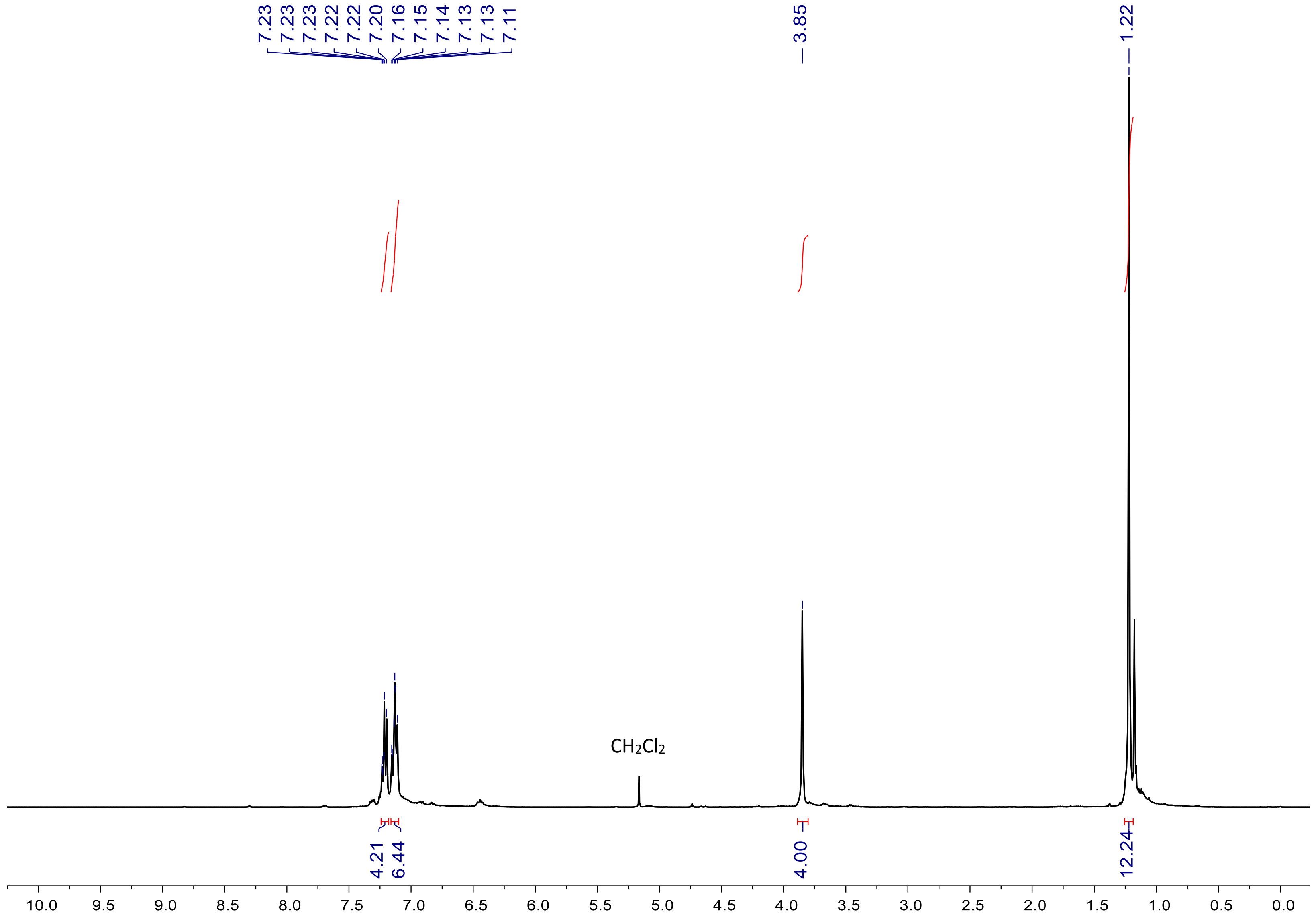

S170 ^{11}B NMR (160 MHz, $CDCl_3$, 298 K) spectrum of *N,N-dibenzyl-4,4,5,5-tetramethyl-1,3,2-dioxaborolan-2-amine* **3l**.

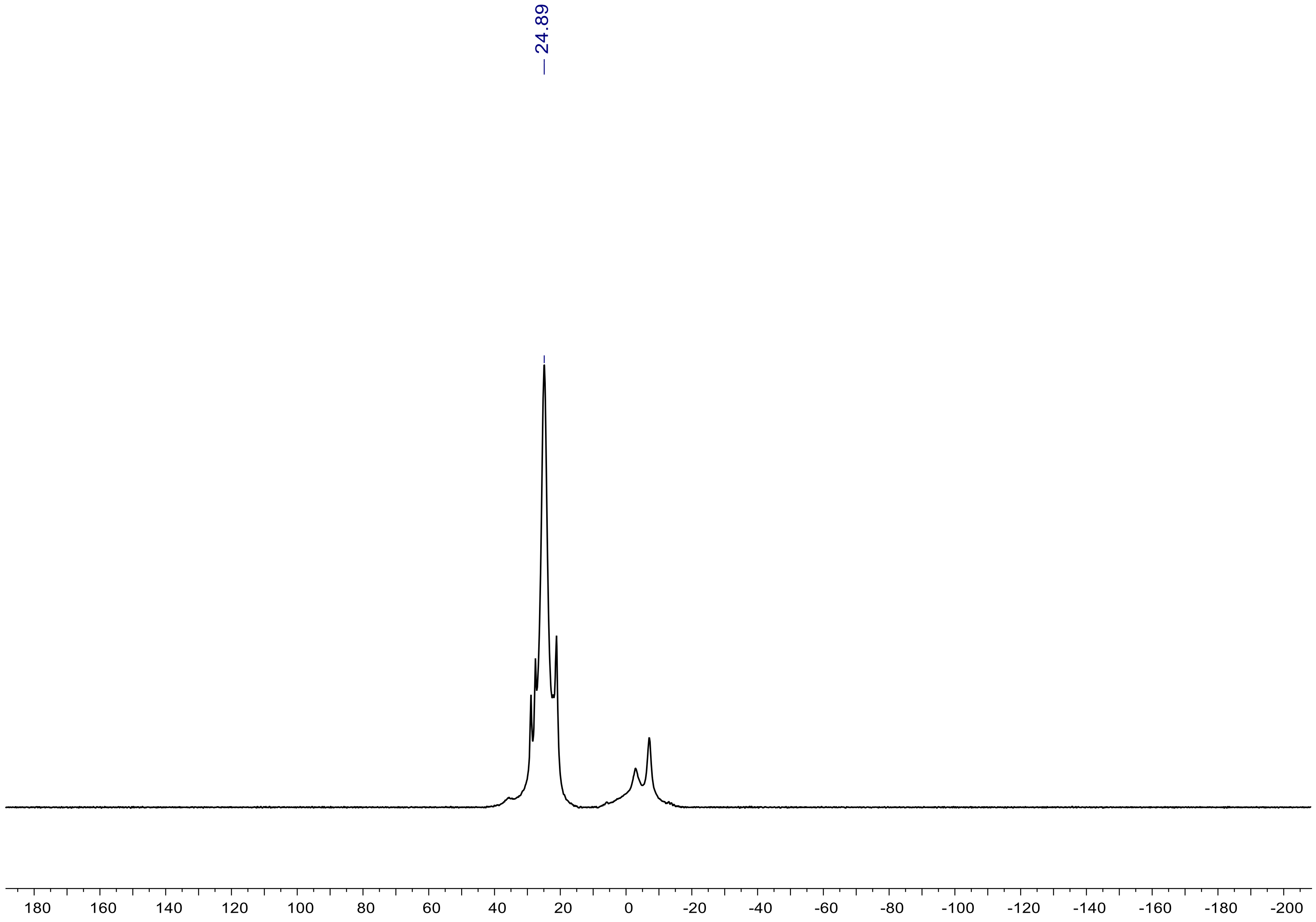

S171 ^{13}C NMR (126 MHz, $CDCl_3$, 298 K) spectrum of *N,N-dibenzyl-4,4,5,5-tetramethyl-1,3,2-dioxaborolan-2-amine* **3l**.

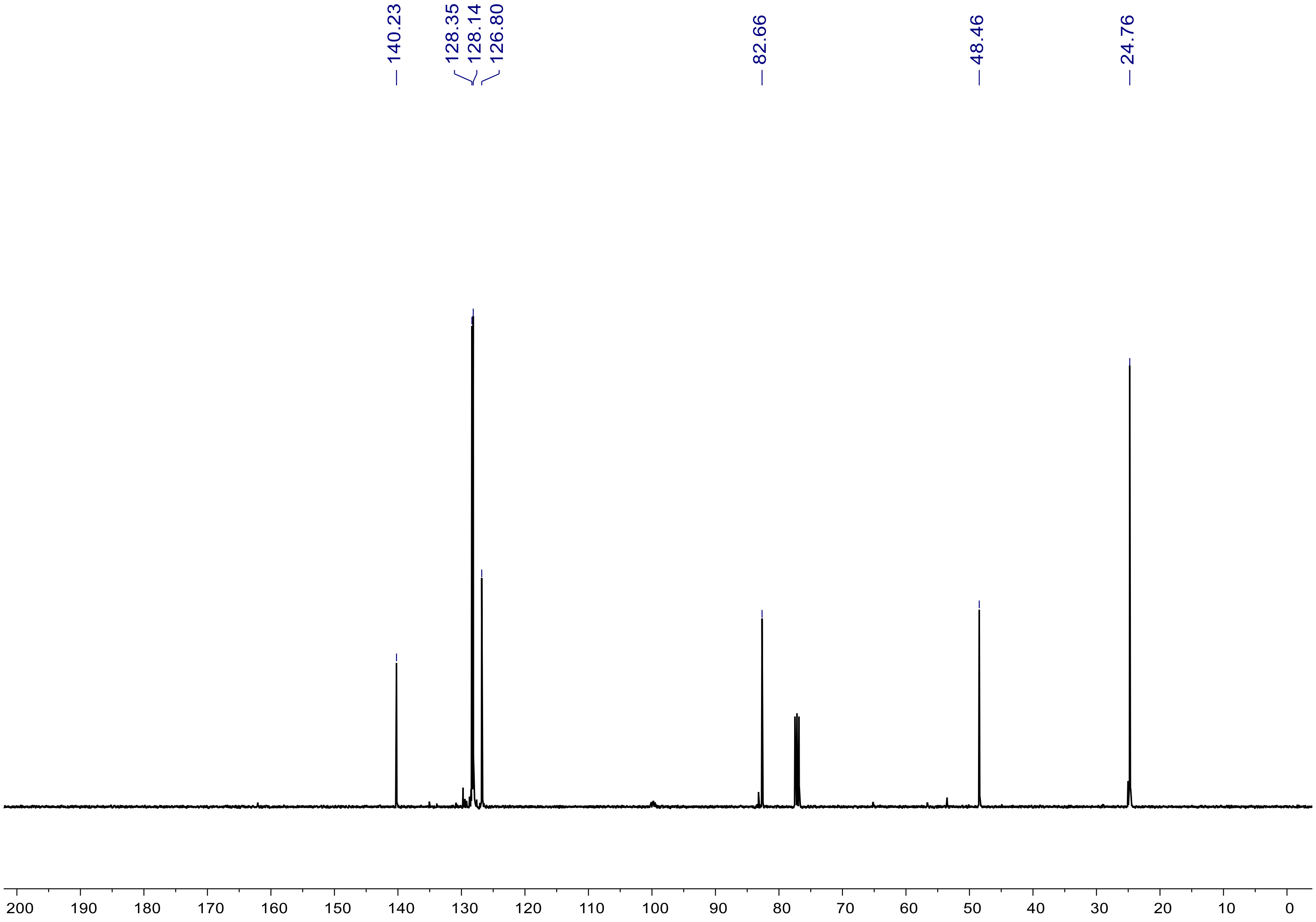

S172 ^{1}H NMR (500 MHz, $CDCl_3$, 298 K) spectrum of *N-benzyl-N-(2,6-diethylphenyl)-4,4,5,5-tetramethyl-1,3,2-dioxaborolan-2-amine* **3m**.

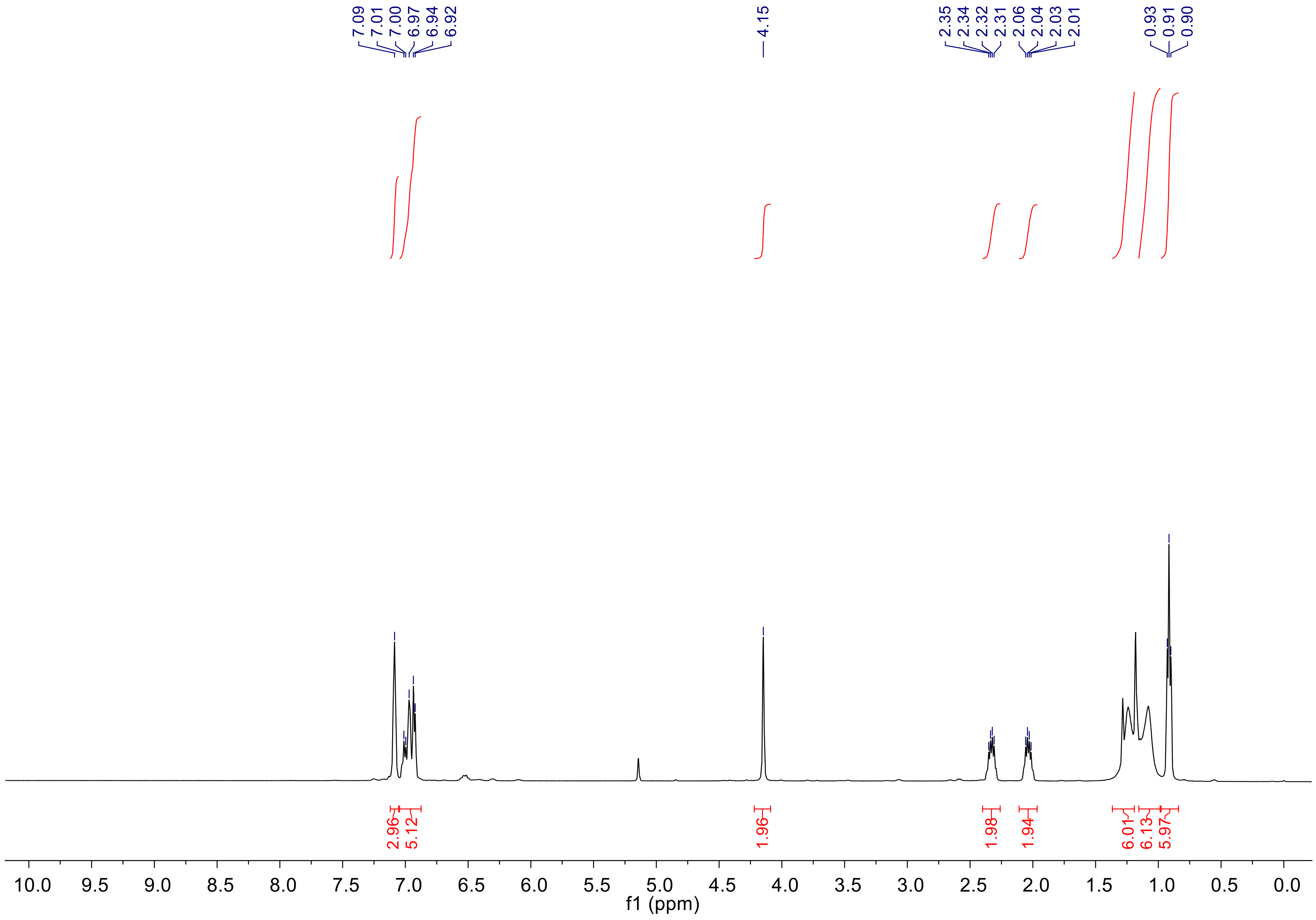

S173 ^{11}B NMR (160 MHz, $CDCl_3$, 298 K) spectrum of *N-benzyl-N-(2,6-diethylphenyl)-4,4,5,5-tetramethyl-1,3,2-dioxaborolan-2-amine* **3m**.

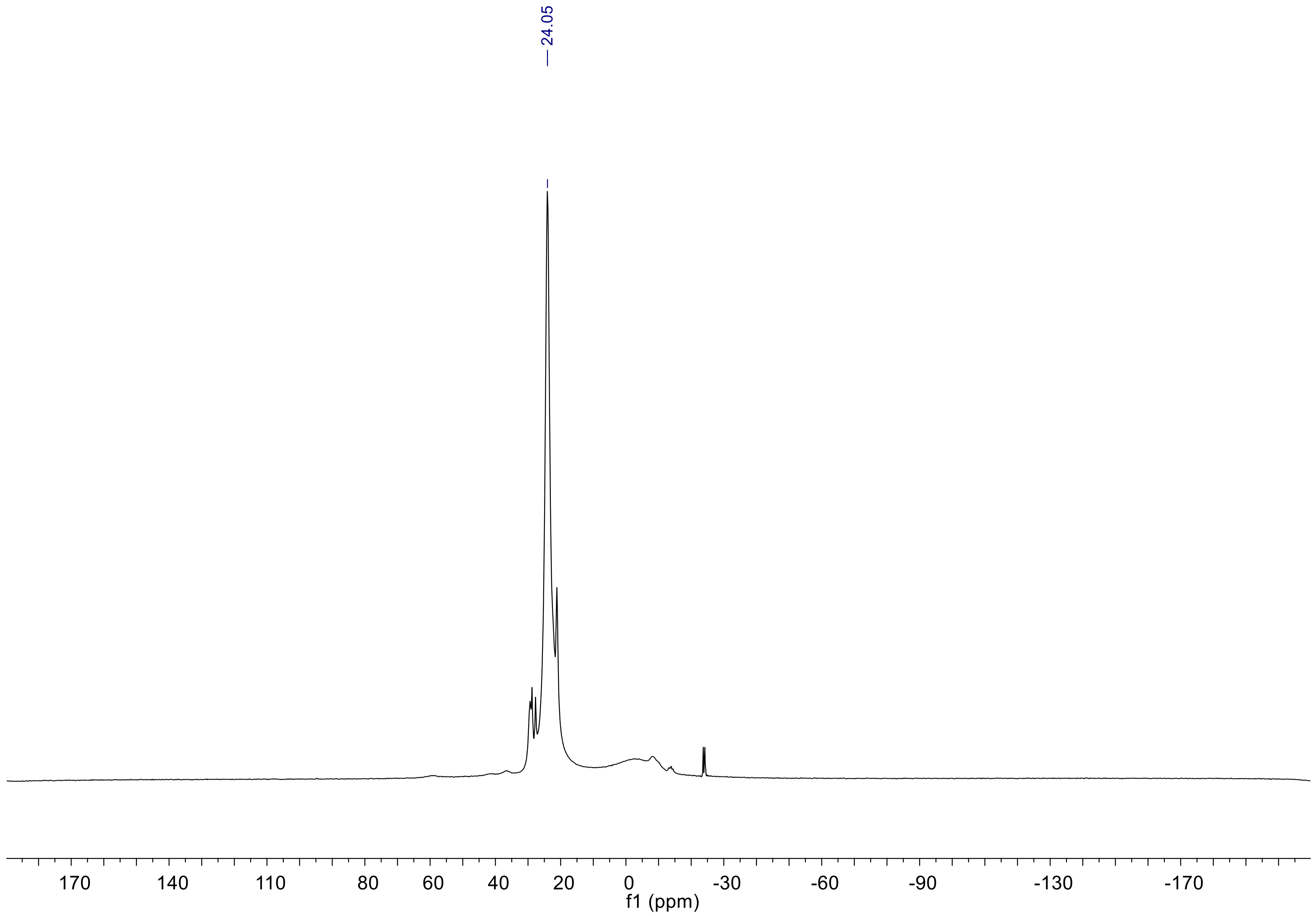

S174 ^{13}C NMR (126 MHz, $CDCl_3$, 298 K) spectrum of *N-benzyl-N-(2,6-diethylphenyl)-4,4,5,5-tetramethyl-1,3,2-dioxaborolan-2-amine* **3m**.

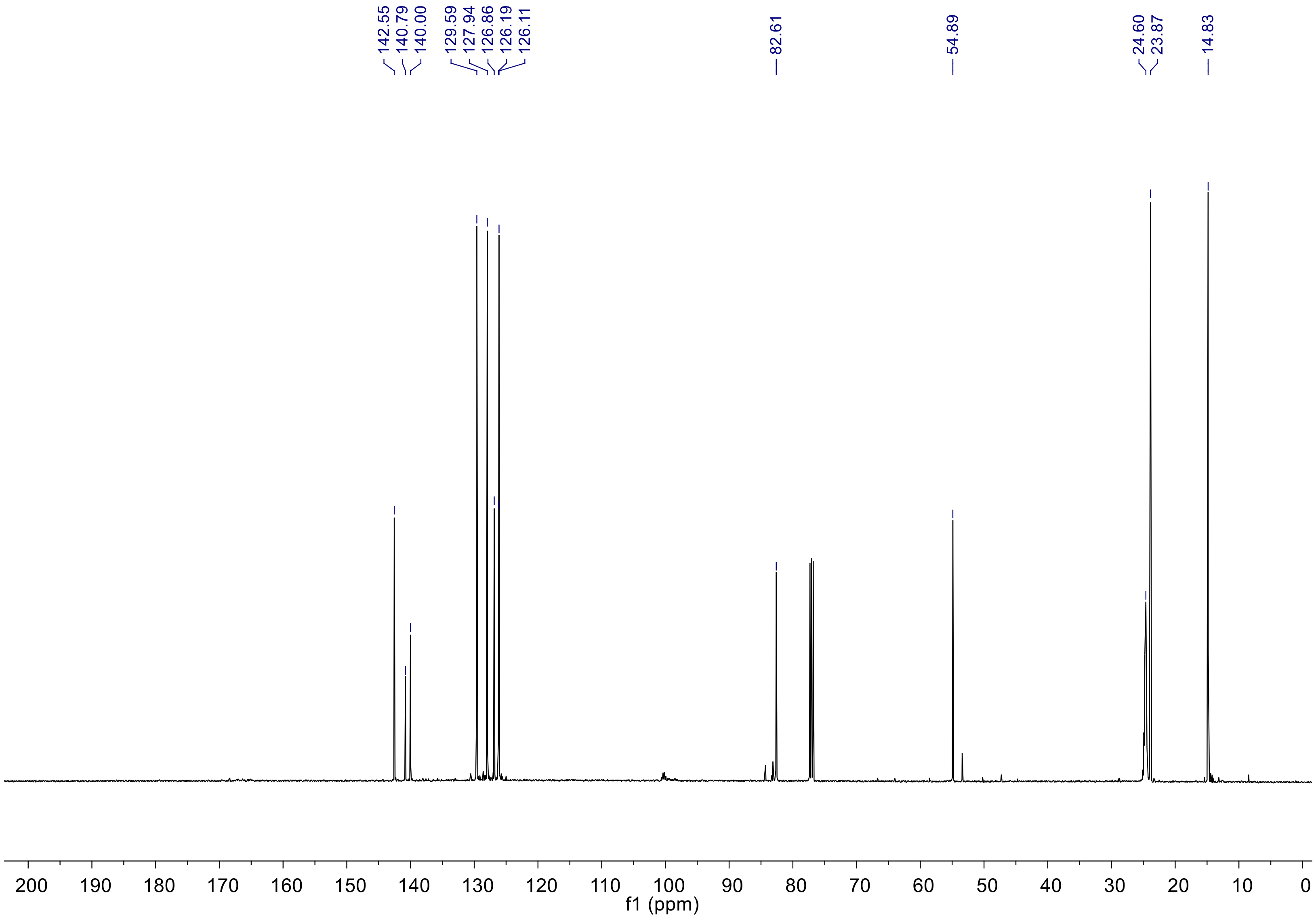

S175 ^{1}H NMR (400 MHz, $CDCl_3$, 298 K) spectrum of *N-benzyl-N-mesityl-4,4,5,5-tetramethyl-1,3,2-dioxaborolan-2-amine* **3n**.

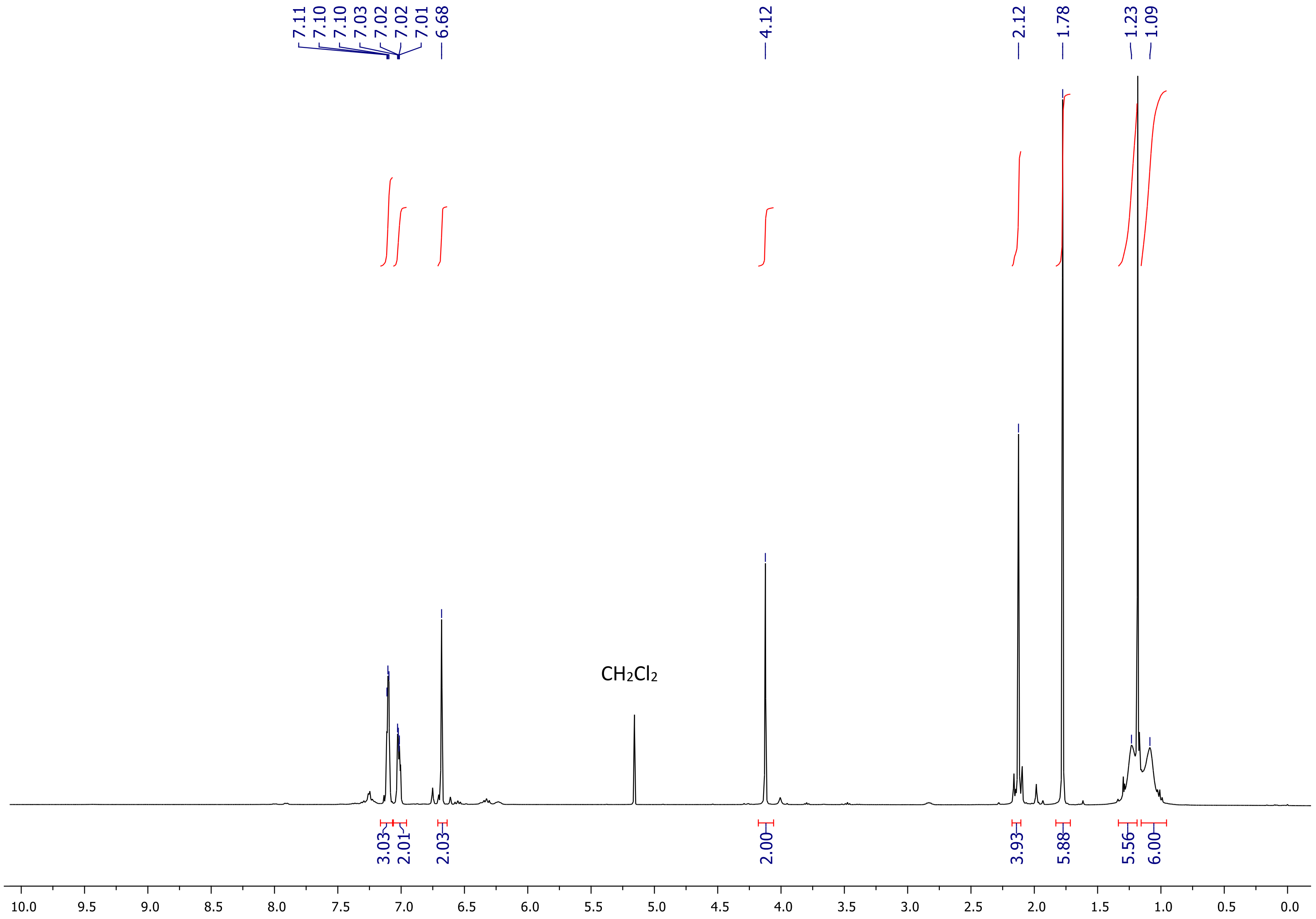

S176 ^{11}B NMR (128 MHz, $CDCl_3$, 298 K) spectrum of *N-benzyl-N-mesityl-4,4,5,5-tetramethyl-1,3,2-dioxaborolan-2-amine* **3n**.

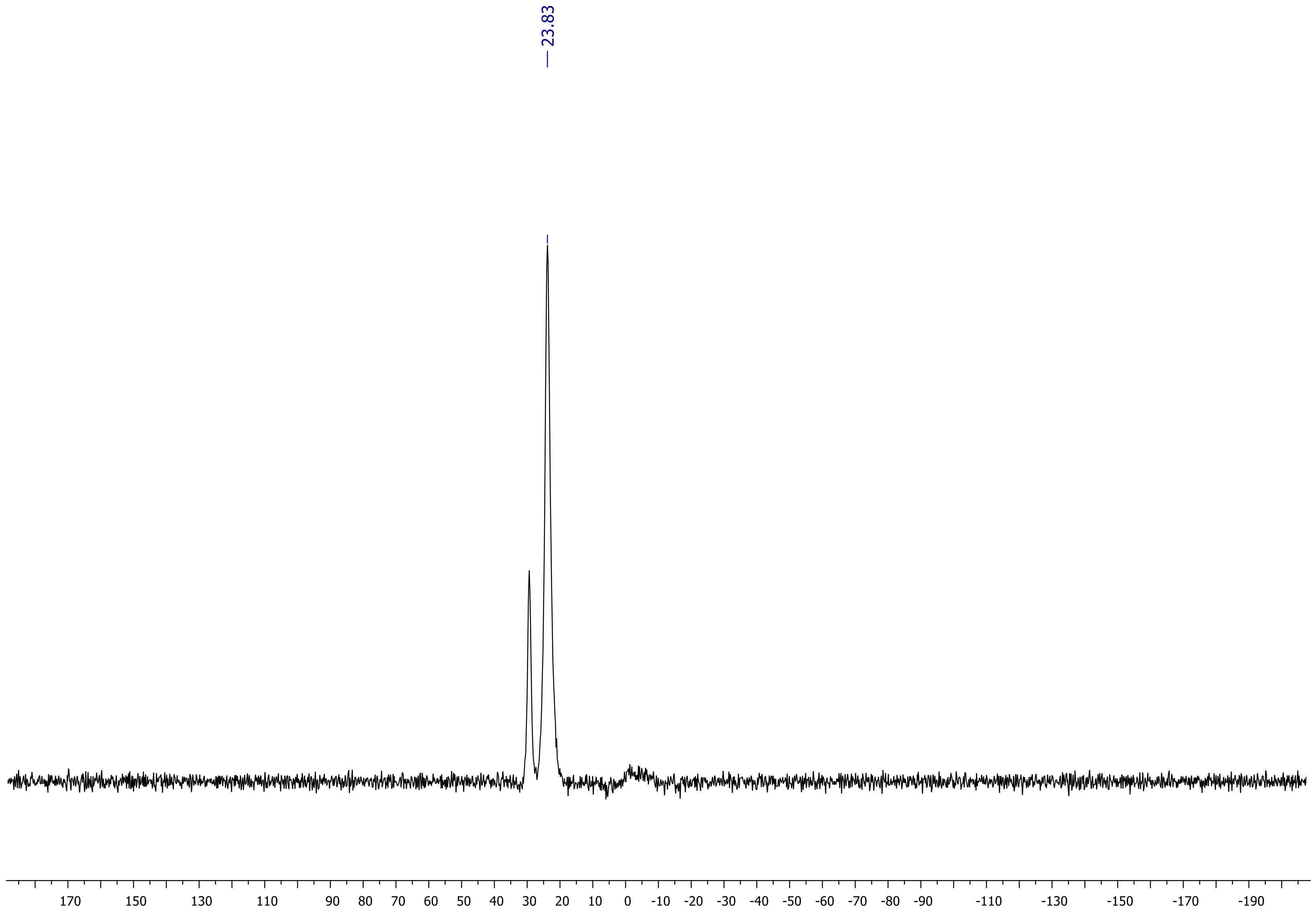

S177 ^{13}C NMR (101 MHz, $CDCl_3$, 298 K) spectrum of *N-benzyl-N-mesityl-4,4,5,5-tetramethyl-1,3,2-dioxaborolan-2-amine* **3n**.

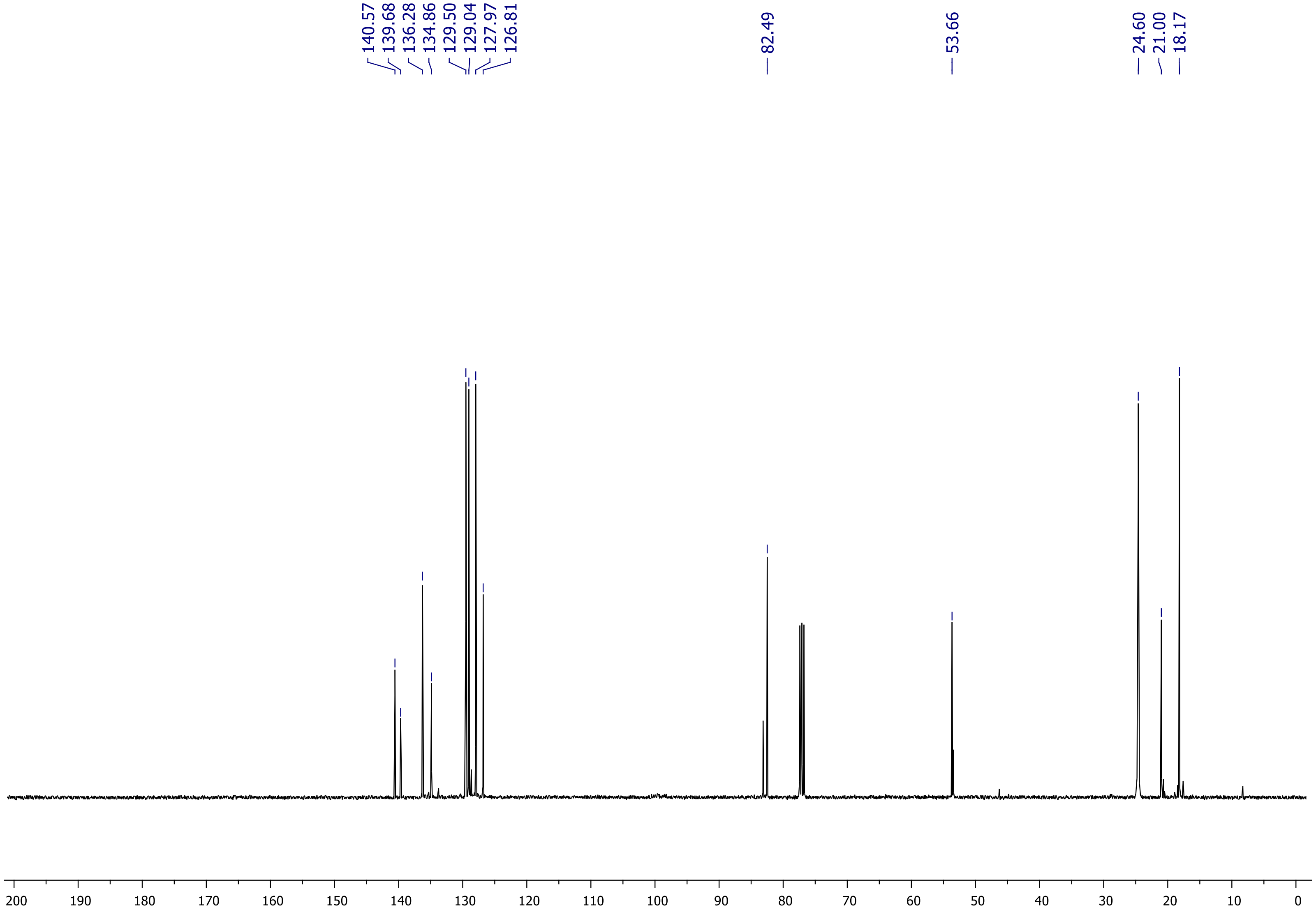

S178 ^{1}H NMR (400 MHz, $CDCl_3$, 298 K) spectrum of *N-benzyl-4,4,5,5-tetramethyl-N-(4-(trifluoromethyl)phenyl)-1,3,2-dioxaborolan-2-amine* **3o**.

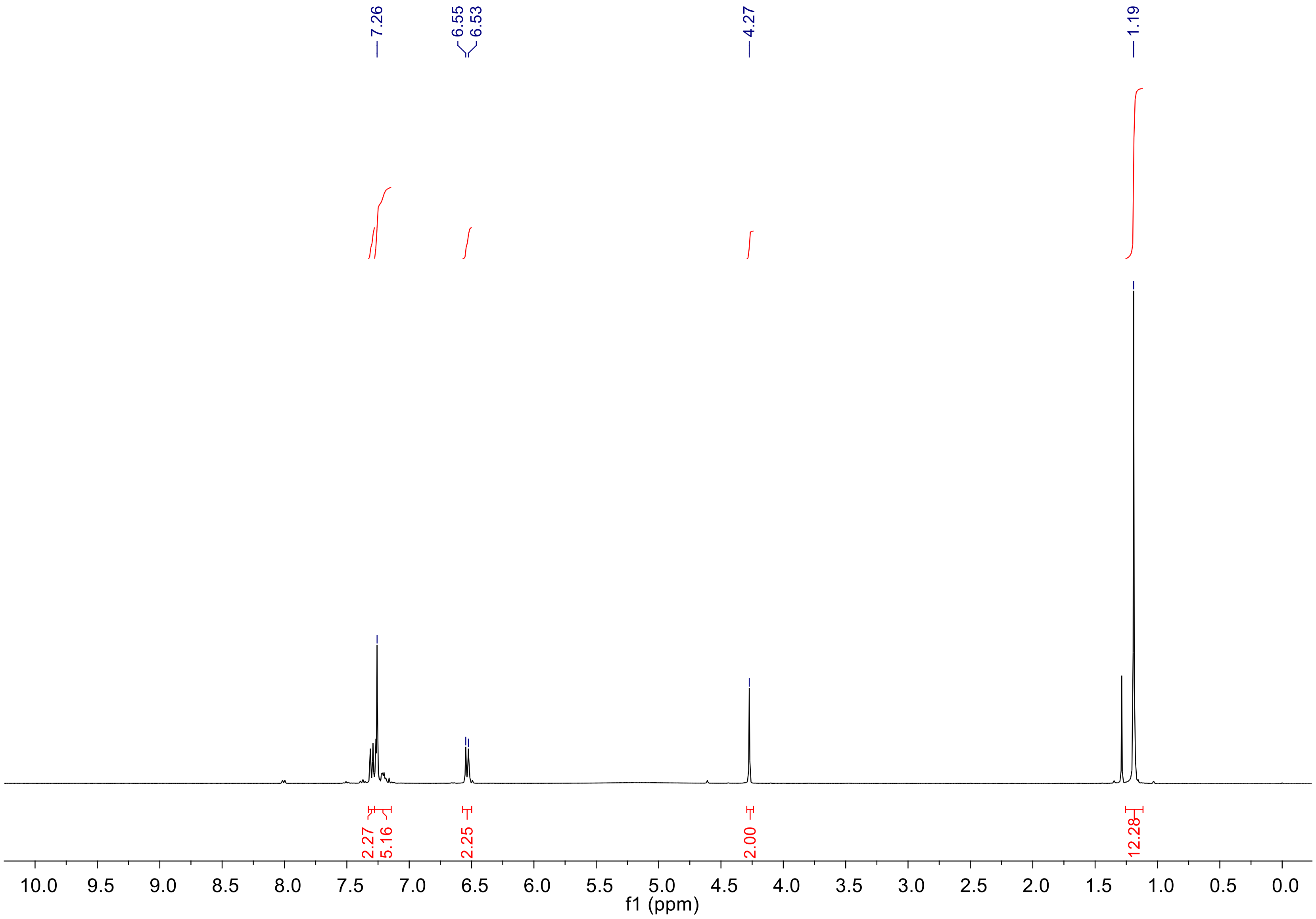

S179 ^{11}B NMR (128 MHz, $CDCl_3$, 298 K) spectrum of *N-benzyl-4,4,5,5-tetramethyl-N-(4-(trifluoromethyl)phenyl)-1,3,2-dioxaborolan-2-amine* **3o**.

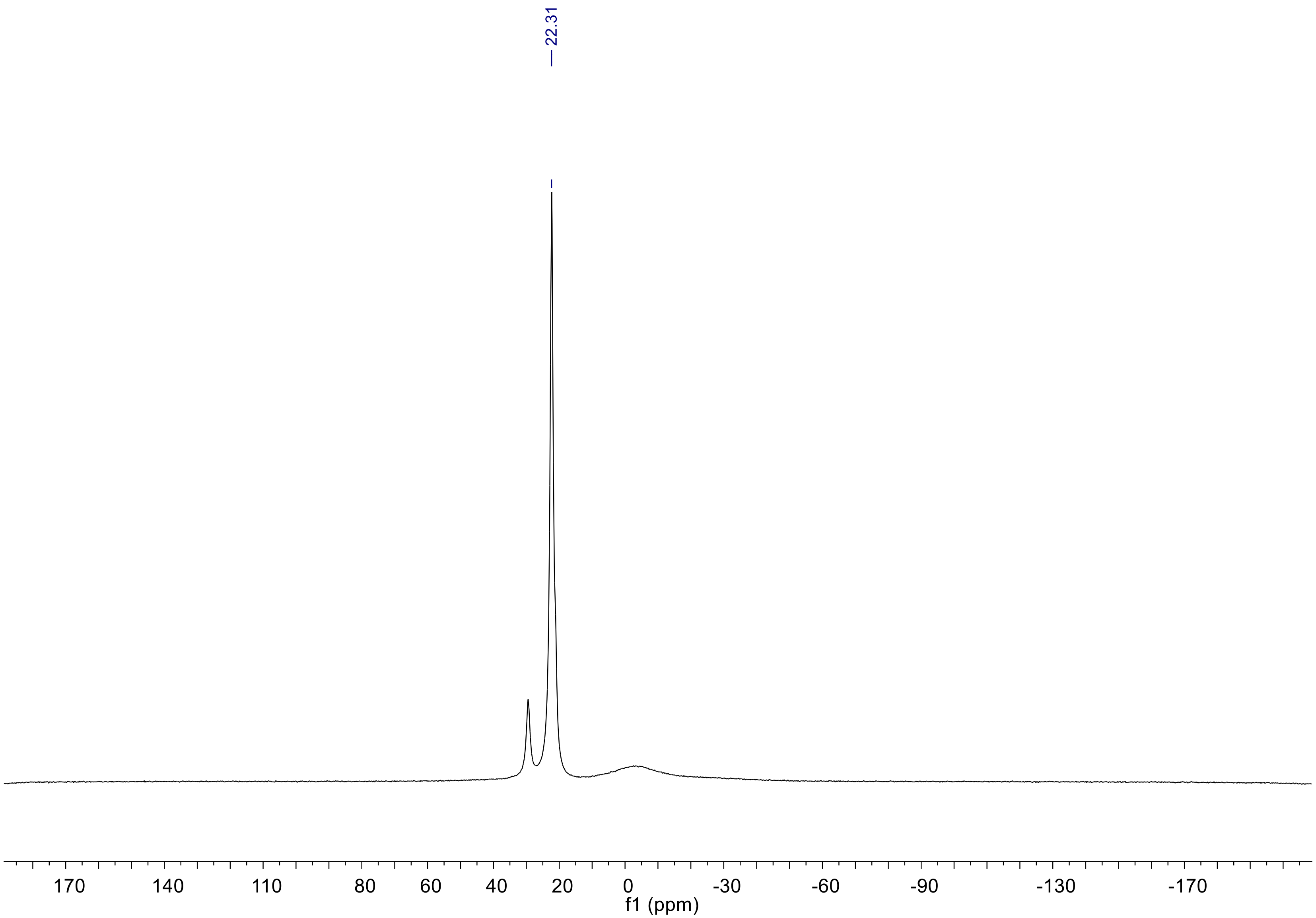

S180 ^{13}C NMR (101 MHz, $CDCl_3$, 298 K) spectrum of *N-benzyl-4,4,5,5-tetramethyl-N-(4-(trifluoromethyl)phenyl)-1,3,2-dioxaborolan-2-amine* **3o**.

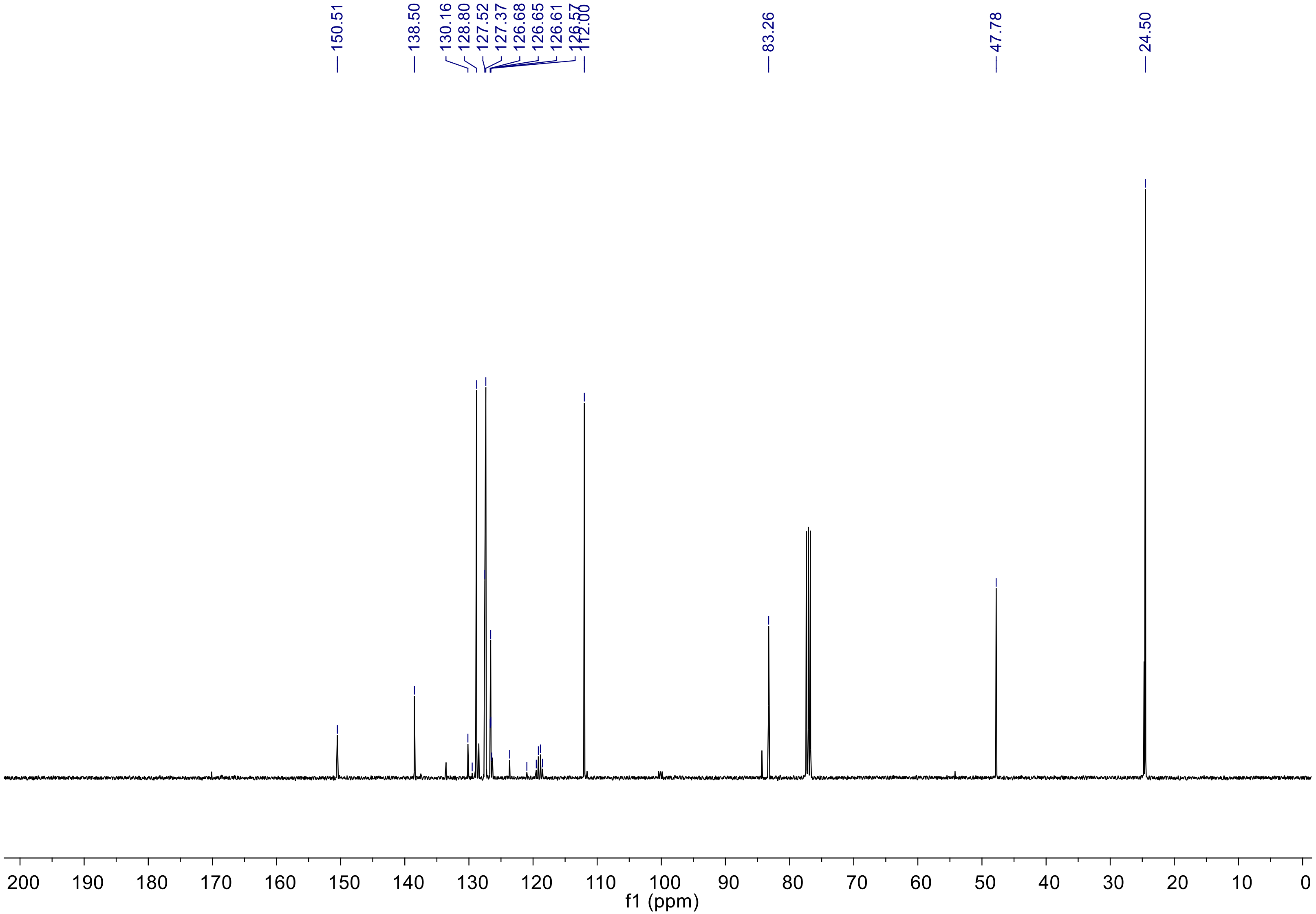

S181 ^{19}F NMR (377 MHz, $CDCl_3$, 298 K) spectrum of *N-benzyl-4,4,5,5-tetramethyl-N-(4-(trifluoromethyl)phenyl)-1,3,2-dioxaborolan-2-amine* **3o**.

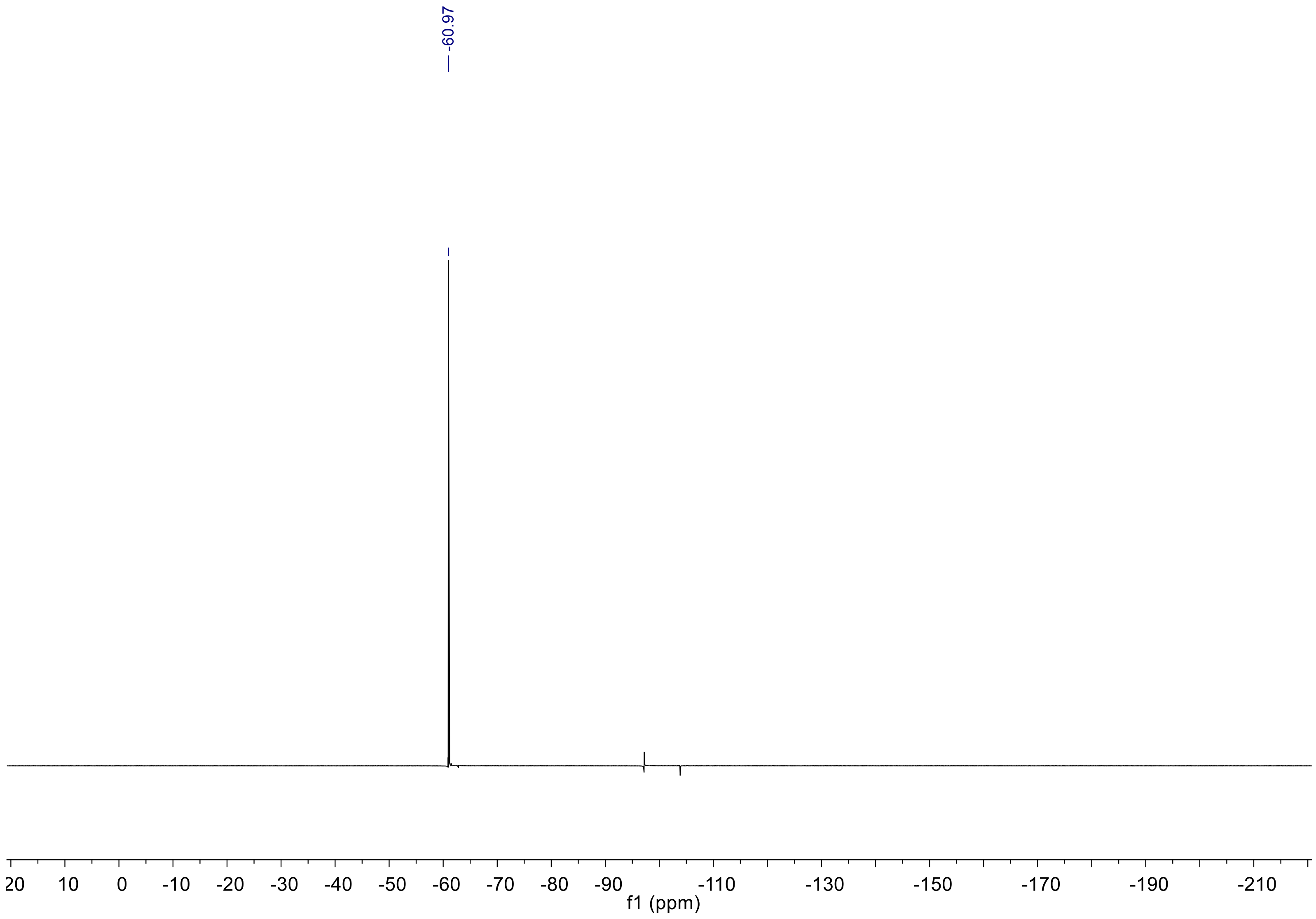

S182 ^{1}H NMR (400 MHz, $CDCl_3$, 298 K) spectrum of the hydrolysis product of *N-benzyl-N-(2-fluorophenyl)-4,4,5,5-tetramethyl-1,3,2-dioxaborolan-2-amine* **3p**.

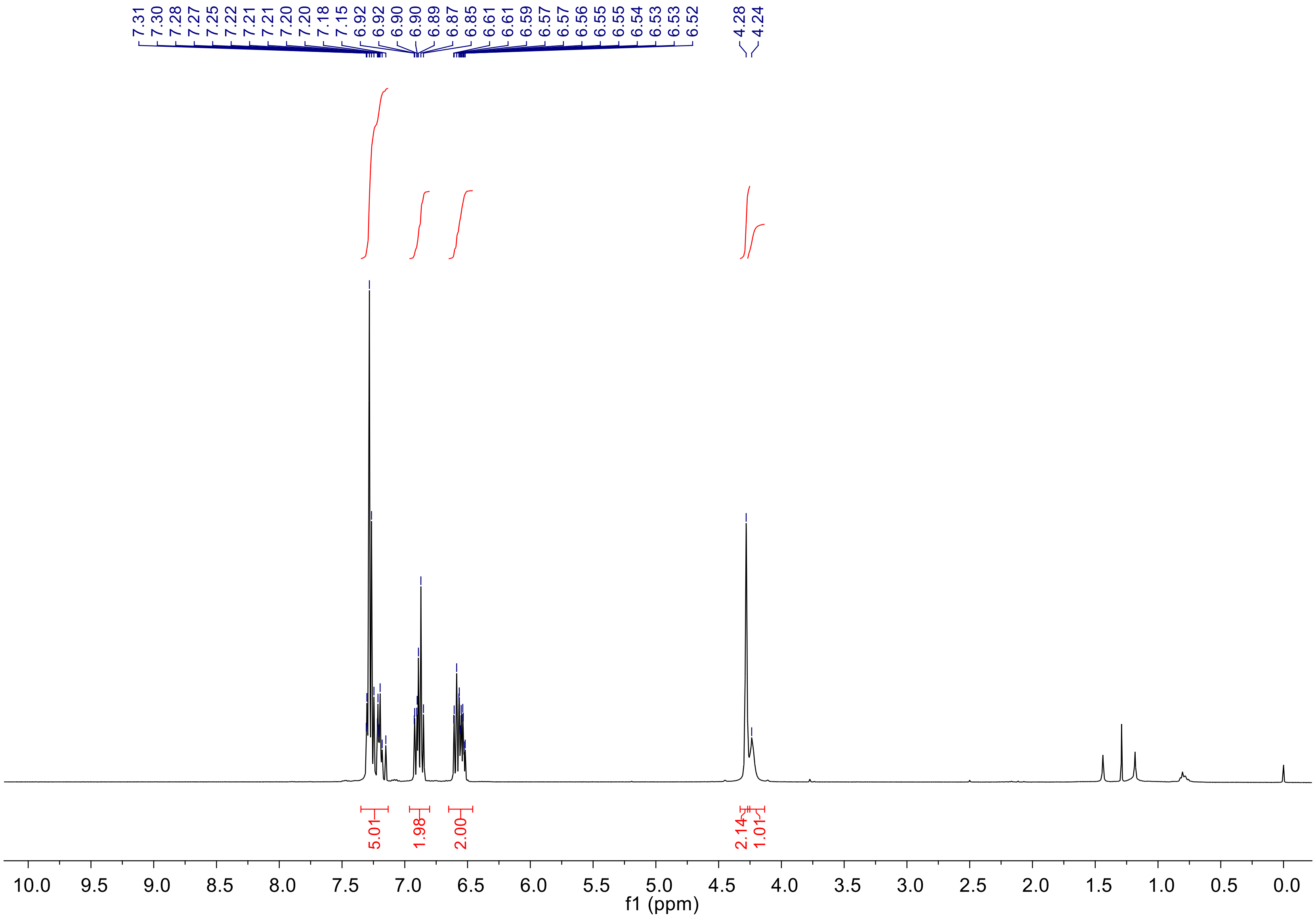

S183 ^{19}F NMR (377 MHz, $CDCl_3$, 298 K) spectrum of the hydrolysis product of *N-benzyl-N-(2-fluorophenyl)-4,4,5,5-tetramethyl-1,3,2-dioxaborolan-2-amine* **3p**.

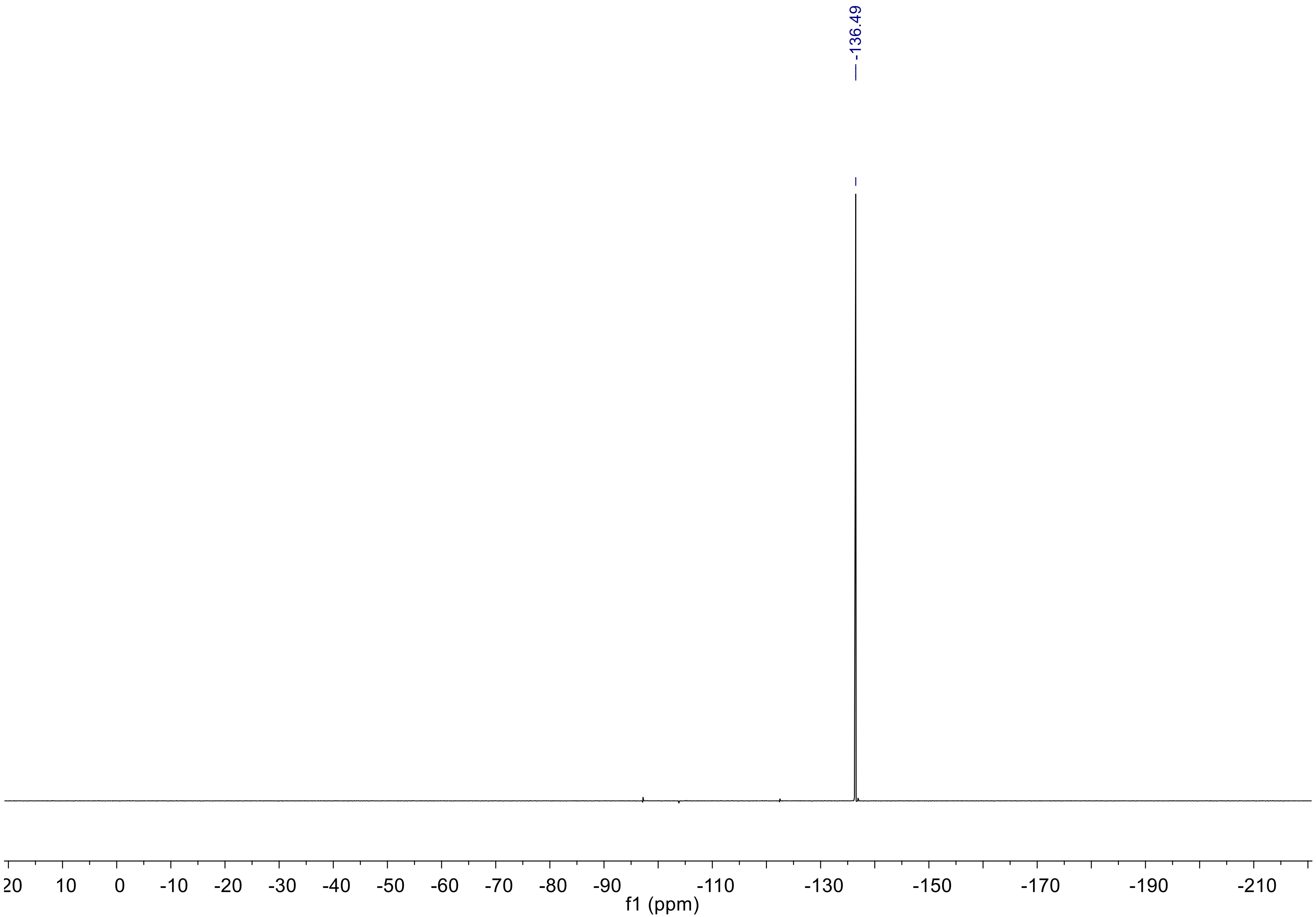

S184 ^{13}C NMR (101 MHz, $CDCl_3$, 298 K) spectrum of the hydrolysis product of *N-benzyl-N-(2-fluorophenyl)-4,4,5,5-tetramethyl-1,3,2-dioxaborolan-2-amine* **3p**.

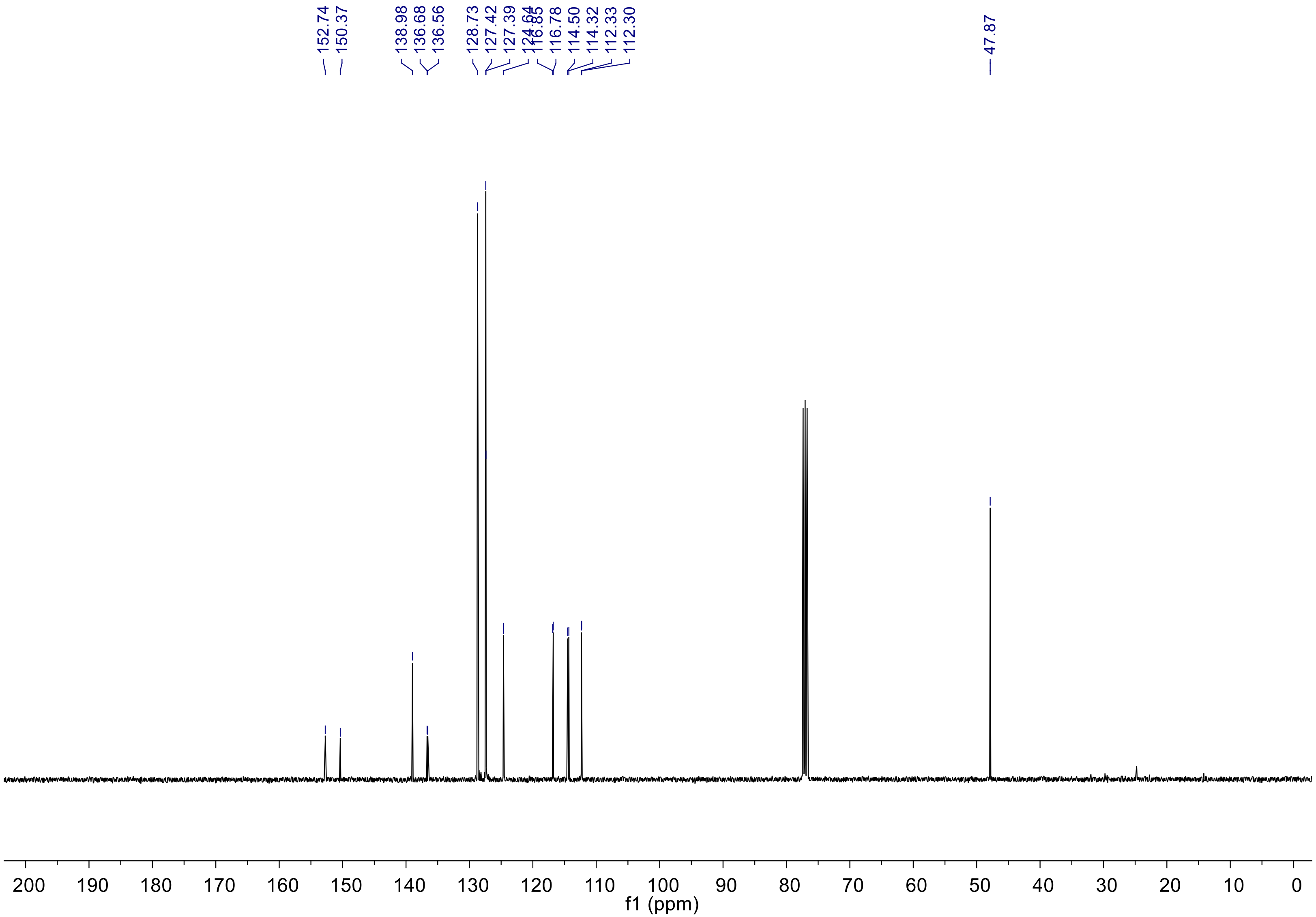

3. Crystallographic studies.

3.1 General information and refinement data.

Crystallographic studies were undertaken of a single crystal mounted in paratone and studied on an Agilent SuperNova Dual Atlas three-circle diffractometer using Cu-Kα radiation and a CCD detector. Measurements were carried out at 150(2) K with temperatures maintained using an Oxford cryostream unless otherwise stated. Data were collected and integrated and data corrected for absorption using a numerical absorption correction based on gaussian integration over a multifaceted crystal model within CrysAlisPro.[26] The structures were solved by direct methods and refined against F^2 within SHELXL-2013.[27] A summary of crystallographic data are available as ESI and the structures deposited with the Cambridge Structural Database (CCDC deposition numbers 1556722–1556723). These data can be obtained free of charge from The Cambridge Crystallographic Data Centre via www.ccdc.cam.ac.uk/data_request/cif.

Table 1. Crystallographic data for **1k** and **1p**.

Compound	**1k**	**1p**
Empirical Formula	$C_{12}H_{25}BO_2Si$	$C_{21}H_{25}BCl_2O_2$
Crystal System	Monoclinic	Monoclinic
Space Group	*P*21/*n*	*P*21/*n*
a/Å	6.5494(4)	18.5123(8)
b/Å	21.5345(13)	6.02084(19)
c/Å	10.8938(7)	19.3786(9)
α/°	90	90
β/°	94.458(6)	107.955(5)
γ/°	90	90
V/Å^3	1531.81(17)	2054.75(15)
Z	4	4
T /K	150(2)	150(2)
D_c/g.cm^{-3}	1.042	1.264
Crystal size/mm	0.236 x 0.182 x 0.095	0.488 x 0.102 x 0.090
Total data	5536	7867
Unique data	2883	4058
R_{int}	0.0446	0.0460
$R_1[F^2>2\sigma(F^2)]$	0.0685	0.0688
wR2 (all data)	0.2004	0.1984
GoF	1.044	1.050
ρ_{min}/ρ_{max}/eÅ^{-3}	-0.391/0.614	-0.926/0.852
CCDC code	1556722	1556723

3.2 Thermal ellipsoid plots.

S185 Solid-state structure of **1k** (thermal ellipsoid probability at 50%) C: black, O: red, B: yellow-green, Si: grey.

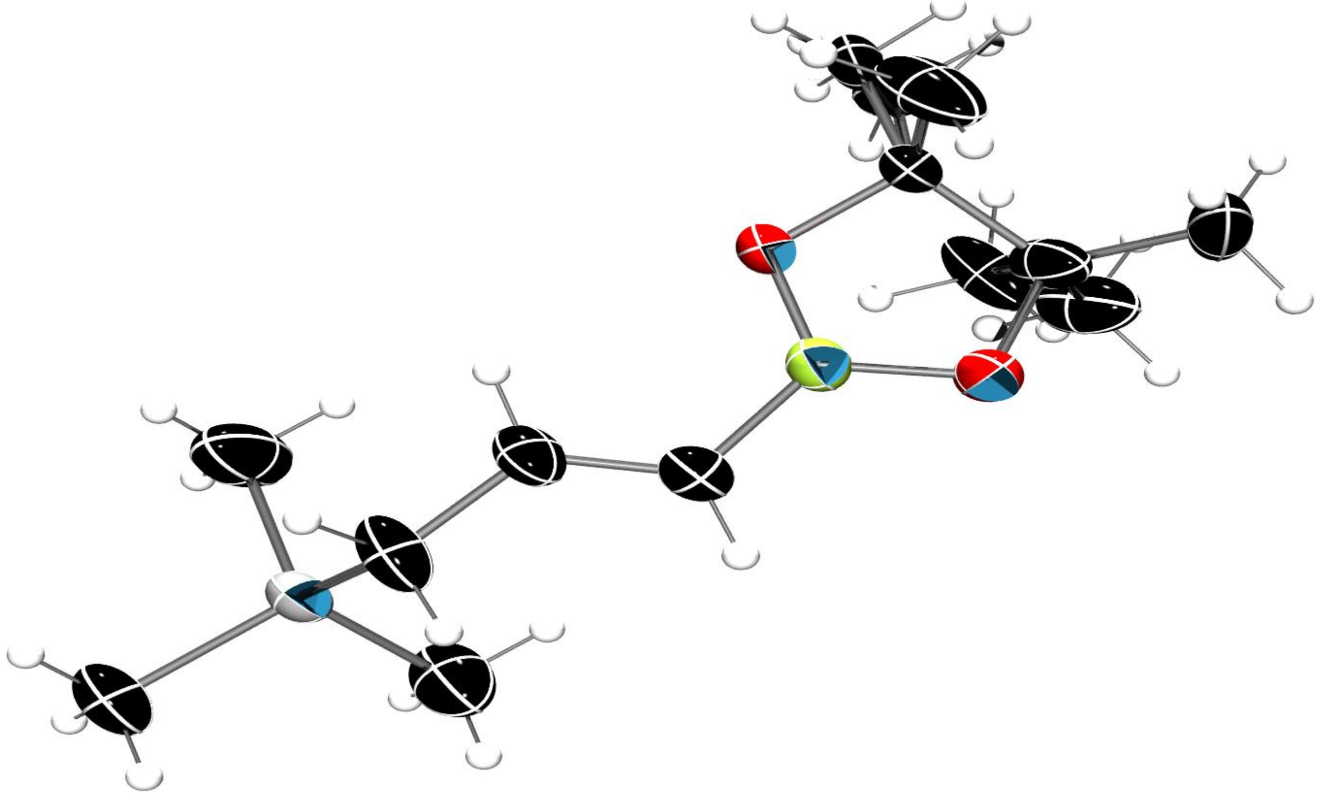

S186 Solid state-structure of **1p** (thermal ellipsoid probability at 50%) C: black, O: red, B: yellow-green. CH_2Cl_2 solvent molecule omitted for clarity.

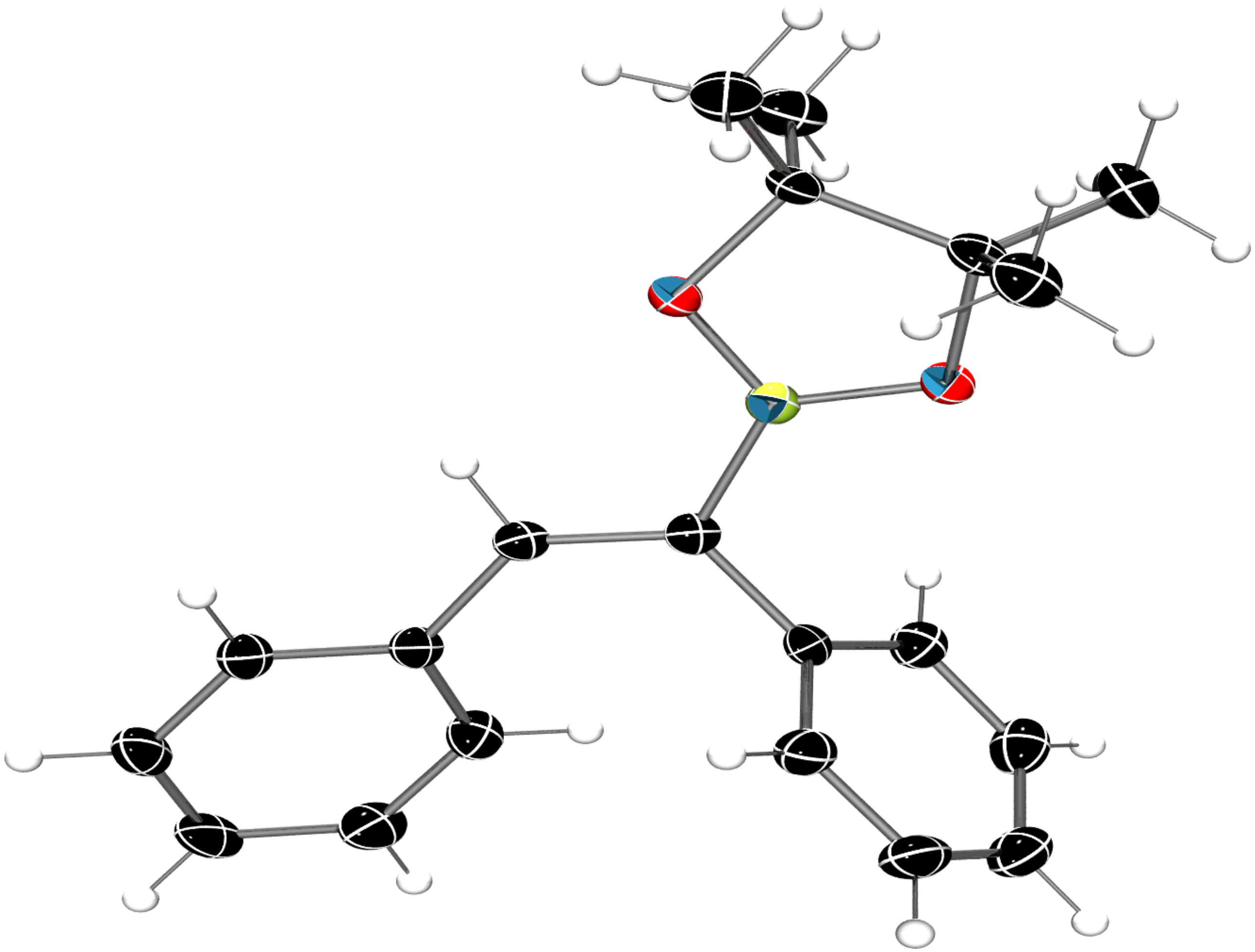

4. References

[1] J. A. Nicasio, S. Steinberg, B. Inés, M. Alcarazo, *Chem. Eur. J.* **2013**, *19*, 11016-11020.

[2] K. Lam, I. E. Markó, *Tetrahedron* **2009**, *65*, 10930-10940.

[3] M. M. Hansmann, R. L. Melen, F. Rominger, A. S. K. Hashmi, D. W. Stephan, *J. Am. Chem. Soc.* **2014**, *136*, 777-782.

[4] J. D. Moore, R. J. Byrne, P. Vedantham, D. L. Flynn, P. R. Hanson, *Org. Lett.* **2003**, *5*, 4241-4244.

[5] S. Punna, S. Meunier, M. G. Finn, *Org. Lett.* **2004**, *6*, 2777-2779.

[6] J. A. Buonomo, C. C. Aldrich, *Angew. Chem. Int. Ed.* **2015**, *54*, 13041-13044.

[7] C. Wang, K. Huang, J. Wang, H. Wang, L. Liu, W. Chang, J. Li, *Adv. Synth. Catal.* **2015**, *357*, 2795-2802.

[8] J. Bhattacharjee, M. Sachdeva, T. K. Panda, *Z. Anorg. Allg. Chem.* **2016**, *642*, 937-940.

[9] A. Hasegawa, Y. Naganawa, M. Fushimi, K. Ishihara, H. Yamamoto, *Org. Lett.* **2006**, *8*, 3175-3178.

[10] J. R. Miecznikowski, R. H. Crabtree, *Polyhedron* **2004**, *23*, 2857-2872.

[11] L. Han, P. Xing, B. Jiang, *Org. Lett.* **2014**, *16*, 3428-3431.

[12] J.-M. Huang, J.-F. Zhang, Y. Dong, W. Gong, *J. Org. Chem.* **2011**, *76*, 3511-3514.

[13] M. Okimoto, Y. Takahashi, K. Numata, Y. Nagata, G. Sasaki, *Synth. Commun.* **2005**, *35*, 1989-1995.

[14] Z.-Q. Feng, X.-L. Yang, Y.-F. Ye, L.-Y. Hao, *Bull. Korean Chem. Soc.* **2014**, *35*, 1121-1127.

[15] D.-W. Tan, H.-X. Li, M.-J. Zhang, J.-L. Yao, J.-P. Lang, *Chemcatchem* **2017**, *9*, 1113-1118.

[16] K. Shirakawa, A. Arase, M. Hoshi, *Synthesis* **2004**, *2004*, 1814-1820.

[17] M. Fleige, J. Mobus, T. vom Stein, F. Glorius, D. W. Stephan, *Chem. Commun.* **2016**, *52*, 10830-10833.

[18] A. Macé, F. Tripoteau, Q. Zhao, E. Gayon, E. Vrancken, J.-M. Campagne, B. Carboni, *Org. Lett.* **2013**, *15*, 906-909.

[19] M. K. Barman, A. Baishya, S. Nembenna, *Dalton Trans.* **2017**, *46*, 4152-4156.

[20] Y. Wu, C. Shan, Y. Sun, P. Chen, J. Ying, J. Zhu, L. Liu, Y. Zhao, *Chem. Commun.* **2016**, *52*, 13799-13802.

[21] P. Selig, W. Raven, *Org. Lett.* **2014**, *16*, 5192-5195.

[22] Z. Yang, M. Zhong, X. Ma, S. De, C. Anusha, P. Parameswaran, H. W. Roesky, *Angew. Chem. Int. Ed.* **2015**, *54*, 10225-10229.

[23] D. Mukherjee, A. Ellern, A. D. Sadow, *Chem. Sci.* **2014**, *5*, 959-964.

[24] D. Mukherjee, H. Osseili, K.-N. Truong, T. P. Spaniol, J. Okuda, *Chem. Commun.* **2017**, *53*, 3493-3496.

[25] V. Fasano, J. E. Radcliffe, M. J. Ingleson, *ACS Catal.* **2016**, *6*, 1793-1798.

[26] CrysAlisPro, Agilent Technologies, Version 1.171.37.33 (release 27-03-2014 CrysAlis171 .NET).

[27] SHELXL-2013, G.M. Sheldrick, University of Göttingen, Germany (2013).
